# Analysis of *CACTA* transposases reveals intron loss as major factor influencing their exon/intron structure in monocotyledonous and eudicotyledonous hosts

**DOI:** 10.1186/1759-8753-5-24

**Published:** 2014-09-01

**Authors:** Jan P Buchmann, Ari Löytynoja, Thomas Wicker, Alan H Schulman

**Affiliations:** 1 Institute of Biotechnology, Viikki Biocenter, University of Helsinki, PO Box 65, FIN-00014 Helsinki, Finland; 2 Institute of Plant Biology, University of Zurich, Zollikerstrasse 107, Zurich, Switzerland; 3 Biotechnology and Food Research, MTT Agrifood Research Finland, Myllytie 1, FIN-31600 Jokioinen, Finland; 4 Present address: Marie Bashir Institute for Infectious Diseases and Biosecurity, Charles Perkins Center, University of Sydney, Sydney NSW 2006, Australia

**Keywords:** Transposases, Intron loss, Molecular evolution, DNA transposons, Plants

## Abstract

**Background:**

*CACTA* elements are DNA transposons and are found in numerous organisms. Despite their low activity, several thousand copies can be identified in many genomes. *CACTA* elements transpose using a ‘cut-and-paste’ mechanism, which is facilitated by a DDE transposase. DDE transposases from *CACTA* elements contain, despite their conserved function, different exon numbers among various *CACTA* families. While earlier studies analyzed the ancestral history of the DDE transposases, no studies have examined exon loss and gain with a view of mechanisms that could drive the changes.

**Results:**

We analyzed 64 transposases from different *CACTA* families among monocotyledonous and eudicotyledonous host species. The annotation of the exon/intron boundaries showed a range from one to six exons. A robust multiple sequence alignment of the 64 transposases based on their protein sequences was created and used for phylogenetic analysis, which revealed eight different clades. We observed that the exon numbers in *CACTA* transposases are not specific for a host genome. We found that ancient *CACTA* lineages diverged before the divergence of monocotyledons and eudicotyledons. Most exon/intron boundaries were found in three distinct regions among all the transposases, grouping 63 conserved intron/exon boundaries.

**Conclusions:**

We propose a model for the ancestral *CACTA* transposase gene, which consists of four exons, that predates the divergence of the monocotyledons and eudicotyledons. Based on this model, we propose pathways of intron loss or gain to explain the observed variation in exon numbers. While intron loss appears to have prevailed, a putative case of intron gain was nevertheless observed.

## Background

*CACTA* elements are DNA transposons found in genomes across the phylogenetic spectrum, from algae [1] to vascular plants [2-6] to animals [7,8]. The first *CACTA* element described at the molecular level was *En-1* in *Zea mays*[2]; since then, they have been well documented in the grasses. Although *CACTA* elements usually do not account for the large genome sizes found in grasses, *CACTA* families nevertheless can be highly abundant. In a few cases, however, including *Tpo1* in Lolium *perenne* (ryegrass) and *Caspar* in the Triticeae, *CACTA* elements are known to have contributed considerably to the expansion of the genome size of their host [9-12]. Moreover, *CACTA*s can influence the evolution of the host genome in other ways [12]. In *Glycine max* (soybean), *CACTA* elements can affect flower color and capture host genes [13-16]. *CACTA* elements are sometimes associated with regulatory elements of genes, therefore possibly influencing gene expression [10,17]. Despite their prevalence and impact, evolutionary studies about *CACTA* elements, or DNA transposons in general, are scarce.

The *CACTA* superfamily belongs to the Class II of transposable elements, proliferating by a ‘cut and paste’ mechanism. In contrast to Class I elements, which transpose via an RNA intermediate and therefore copy the original element, *CACTA*s transpose the original element itself. *CACTA* elements constitute approximately 2 to 5% of a grass genome [16,18]. However, only few active *CACTA* elements have been identified in plants [2-6,19]. In addition, only seven putative transcribed transposases have been identified in the Triticeae [10].

A full-length *CACTA* element consists of two terminal inverted repeats (TIRs) bordering two open reading frames(ORFs), one encoding a transposase and the other, called ORF2, a protein of unknown function. The first and last 5 bp of the TIRs consist of the highly conserved CACTA and TAGTG motifs, respectively, hence the name of the element. The function of the ORF2 protein has been determined in specific *CACTA* families to support excision and transposition [20]. However, the transposase is the key transposition enzyme. It binds to the TIR during excision, creating a 3-bp target site duplication (TSD) [21]. The catalytic center of the transposase is the acidic triad known as the ‘DDD/E’ motif, which is highly conserved [22].

The presence of *CACTA* elements across the phylogenetic spectrum and the highly conserved catalytic core of their transposases indicate an ancient presence. Interestingly, the number of exons in transposases among *CACTA* transposons differs even among the grasses. Transposases in rice were found that have four exons [23], while studies in maize reported up to eleven exons for *CACTA* transposases [2,24]. In the recently sequenced grass *Brachypodium distachyon*, the exon number for transposases among *CACTA* superfamilies ranges from one to three. Therefore, the analysis of the exon/intron configuration of *CACTA* transposases offers an excellent opportunity to study the evolutionary mechanisms of intron gain and loss in DNA transposons. In addition, analyzing exon number variations in such a highly conserved and ancient gene as the *CACTA* transposase can offer a perspective on the ‘intron-early’ and ‘intron-late’ models [25,26].

The goal of this study was to analyze the differences in exon numbers in *CACTA* transposases in monocotyledonous and eudicotyledonous plants and to identify an evolutionary mechanism to explain those differences. This was accomplished using phylogenetic and comparative analyses, which required a solid and robust multiple sequence alignment (MSA). We constructed such an MSA based on protein consensus sequences of 64 transposases from *CACTA* families annotated in ten monocotyledonous and eudicotyledonous species.

Our phylogenetic analysis revealed that ancient *CACTA* lineages diverged before the divergence of the monocotyledons and eudicotyledons, supporting an intron-early model for *CACTA* transposases. The analysis of the MSA identified conserved exon/intron boundaries and putative intron gain among the transposases examined. Combining these analyses lead to a model for a putative ancient *CACTA* transposase, in which intron loss was the main mechanism shaping the exon/intron configurations of current transposases found in monocotyledonous and eudicotyledonous plants.

## Results

We analyzed 64 autonomous *CACTA* transposases from ten different monocotyledonous and eudicotyledonous species. All analyzed transposases are derived from consensus sequences from distinctive *CACTA* families. Because families of transposable elements (TEs) differ from each other based on the 80-80-80 rule, they were considered orthologous [27]. Therefore, the name of the family, for example, Calvin, will indicate the consensus sequence of the transposase and not the consensus of the whole element. We refer to the plant in which a *CACTA* family and its transposase were annotated as its host. Except for transposases identified in *B. distachyon*, we searched the PTREP [28] and Repbase [29] databases for *CACTA* families with annotated transposases (see Materials and Methods). The selection was based on two criteria: i) the annotation had to clearly state ‘transposase’, that is annotations without ORFs described as transposases were omitted because *CACTA* elements have two ORFs, the transposase and ORF2; ii) the presence of two ORFs was expected, thereby avoiding selection of annotations having a predicted transposase that spans most of a consensus sequence, such as ATENSPM10 in Repbase, where the consensus is 8,272 bp and the predicted transposase covers positions 1,201 to 7,766. We selected nine transposases from *Sorghum bicolor*, eight transposases from *Z. mays*, five transposases from *Triticum aestivum*, 13 from *Oryza sativa*, and 11 from *B.distachyon* (Additional file 1). This resulted in a total of 46 transposases from monocotyledonous hosts. For the eudicotyledonous dataset, we selected all transposases from eudicotyledonous hosts in Repbase fitting our criteria, totaling in eighteen elements: seven transposases from elements annotated in *Arabidopsis thaliana*, five from *Fragaria vesca*, three from *Vitis vinifera*, and one each from *Petunia hybrida, Malus domestica*, and *G. max* (Additional file 1).

### Annotation of exon/intron boundaries on *CACTA* transposases

For simplicity, the term ‘boundary’ will indicate exon/intron boundaries in this study. Except for transposases in *B. distachyon*, boundaries were extracted from the respective PTREP and Repbase entries (Table 1, Material and Methods). The eleven *Brachypodium distachyon* transposases were derived from consensus sequences of the autonomous families in this genome [18]. We manually annotated the transposases and boundaries by aligning to the most similar BLASTX hit within the PTREP database. Additional alignments against transcription databases from rice and *B. distachyon* did not increase the quality of the boundary predictions, because transcriptome data is scarce for *CACTA* transposases. *De novo* gene prediction did not return significant results.

**Table 1:** **Exon/intron boundaries of the 34 analyzed *CACTA *** **transposases with more than one exon.**

	**1**	**2**	**3**	**4**	**5**
EnSpm12_Fves	462 | 564^G^				
C	718 | 771^I^				
EnSpm10_Fves	826 | 846				
Joey	842 | 893^II^				
Janus	837 | 894^II^				
F	846 | 894^II^				
G	847 | 894^II^				
Norman	879 | 921^III^				
En1	879 | 925^III^				
Alfred	885 | 925^III^				
H	838 | 972				
EnSpm3_Vvin	821 | 894^II^	856 | 925^III^			
EnSpm8_Sbic	754 | 783^I^	*976 | 0*			
Storm	827 | 782^I^	*951 | 0*			
Sherman	831 | 782^I^	*954 | 0*			
J	495 | 521	750 | 885			
EnSpm2_Mdom	755 | 782^I^	886 | 893^II^			
Baldur	731 | 782^I^	837 | 895^II^			
I	834 | 782^I^	954 | 910			
Isidor	857 | 894^II^	892 | 920^III^			
Radon	841 | 894^II^	877 | 921^III^			
Rufus	851 | 894^II^	887 | 921^III^			
EnSpm13_Vvin	821 | 894^II^	856 | 925^III^			
EnSpm5_Vvin	824 | 894^II^	859 | 925^III^			
Isaac	861 | 894^II^	900 | 925^III^			
Sandro	744 | 782^I^	851 | 928^III^			
Balduin	850 | 895^II^	890 | 930^III^			
DOPPIA	843 | 894^II^	890 | 936^III^			
K	744 | 7,82I	850 | 936^III^			
Horace	712 | 711	981 | 1,054			
EnSpm4_Fves	*812 | 0*	*992 | 0*	*1,244 | 0*		
EnSpm3_Fves	*681 | 0*	770 | 781^I^	*919 | 0*		
Seamus	730 | 782^I^	833 | 892^II^	878 | 925^III^		
Dario	726 | 711	842 | 839	895 | 890^III^		
Aron	851 | 833	899 | 879^II^	1,013 | 1,060		
Korbin	510 | 567^G^	718 | 782^I^	814 | 894^II^	*853 | 0*	
Chester	520 | 563^G^	728 | 777^I^	823 | 889^II^	858 | 920^III^	
Baron	522 | 568^G^	730 | 781^I^	825 | 893^II^	861 | 925^III^	
EnSpm8_Fves	158 | 163	830 | 893^II^	*975 | 0*	*1,219 | 0*	*1,500 | 0*
ATENSPM6_Athal	802 | 809	918 | 922^III^	978 | 981	1,011 | 1,012	*1,141 | 0*

**Figure 1:**
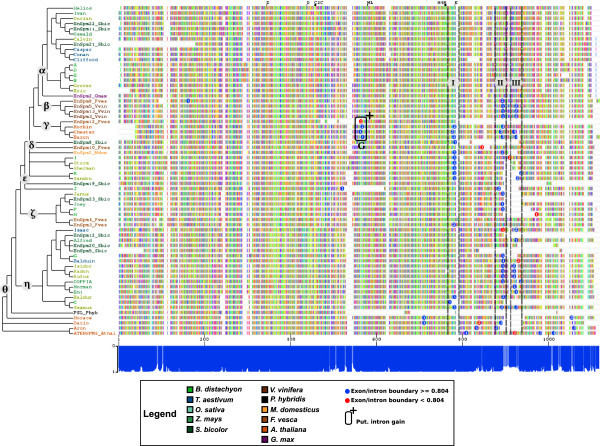
**Multiple sequence alignment based on protein sequences of the 64 analyzed *CACTA *transposases.** Colored boxes indicate amino acids, gray boxes indicate residues with a GUIDANCE score below 0.804, and white boxes indicate gaps in the multiple sequence alignment (MSA). The plot below the MSA shows GUIDANCE scores for the corresponding position in the MSA. Columns with a score below 0.804 are indicated in light blue while columns with a score of 0.804 and above in dark blue. Positions relative to the MSA and corresponding GUIDANCE score are shown between the MSA and the plot. Highly conserved DDE transposase motifs as described in [22] are depicted on top. In the phylogenetic tree, colors indicate the host as shown in the legend. Major clades are depicted α to θ. Exon/intron boundaries are depicted as blue circles if their GUIDANCE score was above 0.804 and red otherwise. The number in the boundary indicates the boundary number on the corresponding transposase. Regions I to III are indicated by dashed lines and corresponding roman capitals. Positions of putative intron gain are depicted as described in the legend.

The positions are relative to the beginning of the transcription start and given as follows:On the protein sequence | on the trimmed multiple sequence alignment (MSA). 0 and numbers in italic indicate boundaries with GUIDANCE scores below 0.804 and removed in the final MSA. Superscripts indicate Regions I to III and G cluster, respectively (Figure 1).

Our final dataset consisted of 64 transposases with 86 annotated boundaries on the 40 transposases that contained more than one exon (Table 1). Out of the 64 annotated transposases, 24 contained only one exon and therefore no boundaries. On the remaining 40 transposases, we annotated between two and six exons (Additional file 1). The length of the transposases ranged from 552 amino acids (amino acids; PSL, 1 exon) to 4,785 amino acids (EnSpm4_Fves, 4 exons), and averaged 1,163 amino acids. The six transposases Isidor, Rufus, Sandro, Radon, Ivan, and Isaac were annotated on the 3’ end of the corresponding *CACTA* consensus sequence (Additional file 1).

### Generation of a robust multiple sequence alignment using confidence scores

Our phylogenetic and comparative analyses were based on an MSA derived from the selected 64 consensus transposase protein sequences. Due to the possibly ancient origin of certain *CACTA* transposases and their generally low activity, we assumed that some parts of sequences might be more evolutionarily diverged than others. In addition, the formation of consensus sequences can introduce weak regions into an MSA. A robust MSA is therefore crucial because errors or uncertainties can influence the downstream analysis. In addition, identifying weakly aligned regions or positions in an MSA and then removing them may improve downstream phylogenetic analysis [30].

GUIDANCE is a method to infer unreliable regions in an MSA and remove the potentially erroneous signal from subsequent analyses ([31]; Materials and Methods). The final MSA was 2,516 residues long and contained five unstable regions placed between positions 120 to 186, 196 to 251, 381 to 416, 728 to 766, and in the 3’ end, starting from position 1,665 (Additional file 2). GUIDANCE scores range from 0 (low confidence) to 1 (high confidence) and are calculated for single residues as well as for whole columns. Because there is no recommended confidence score for residues and columns in an MSA, a trade-off between sensitivity and specificity is required. High sensitivity (low cutoff value) retains as many columns as possible while high specificity (high cutoff value) keeps only columns of very high confidence.

The default GUIDANCE cutoff of 0.93 removed 638 columns (approximately 25%) from the alignment, including the badly aligned regions and 34 annotated boundaries. However, GUIDANCE kept columns with only one residue, for example, most of the badly aligned 3’ end. To retain as many boundaries as possible for the analysis we applied our own trimming: we removed columns containing only residues with scores below 0.804 (keeping boundaries) and columns with only one residue (not comparable and/or bad aligned). This approach removed 1,398 columns (approximately 44%): the badly aligned regions but only 13 annotated boundaries. This final MSA was 1,118 residues long and contained 73 annotated boundaries in 64 transposases (Figure 1). Because the first boundary is also the beginning of the first intron, introns were named in the 5’ to 3’ direction and designated as subscripts to the name of the transposase, for example, the first intron and boundary of transposase Baron is described as Baron_1_. We mapped conserved DDE motifs [22] onto the MSA, which were all in positions with high confidence values (Figure 1). This MSA was used for all further analysis.

### Exon numbers in *CACTA* transposases are not specific to a host genome

**Figure 2:**
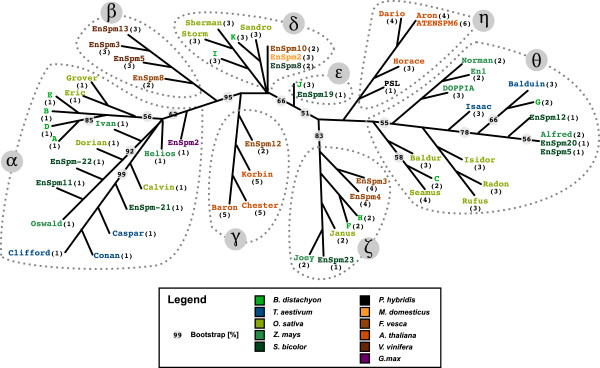
**Majority-rule based phylogram of the 64 analyzed *CACTA *transposases.** The phylogenetic tree is the same as in Figure 1. Bootstrap values represent the percentage out of 1,000 bootstrap replicates. Only bootstraps below 100% are indicated. Transposase hosts are colored as indicated in the legend. Numbers in parentheses indicate the number of exons. Clades are indicated by dashed lines and labeled α to θ.

RAxML [32] was used to calculate the phylogenetic tree (Figure 2). A maximum likelihood (ML) tree was generated based on 200 distinct, randomized, maximum parsimony trees and its robustness assessed by using 1,000 bootstrap replicates and by testing the influence of several outgroups (Additional file 3, Material and Methods). The resulting tree shows the relation between individual transposases but not their evolution over time; that is the branch lengths do not indicate the time when transposases diverged from each other but how close they are on the molecular level (Figure 2). We identified eight clades, designated α to θ (Figure 2). Crucially, the transposases grouped primarily by their exon numbers rather than by their hosts and the analysis of the clusters found no host-specific exon numbers for *CACTA* transposases (Figure 2).

### Ancient *CACTA* lineages diverged before the divergence of monocotyledons and eudicotyledons

We identified three clades in which monocotyledonous and eudicotyledonous transposases clustered together. EnSpm2_Gmax from soybean grouped in Clade α with transposases from several monocotyledonous hosts, analogous to EnSpm3_Fves and EnSpm4_Fves from strawberry in Clade ζ. Clade δ grouped transposases from strawberry, apple, and several grasses. The other clades contained only transposases from either eudicotyledonous or monocotyledonous hosts (Figure 2). Despite the long evolutionary time separating monocotyledonous and eudicotyledonous hosts, the presence of mixed clades and the close relation of clades with only monocotyledonous or eudicotyledonous hosts suggests that the *CACTA* transposase phylogeny rather than the host phylogeny is primary, that is that the main transposase branches diverged already before the divergence of monocotyledons and eudicotyledons. Indeed, a closer look at the phylogenetic tree revealed that transposases within clades tend to have the same number of exons (Figure 2).

### The majority of *CACTA* transposase boundaries are found in three regions on the MSA

To analyze the evolution of exon/intron arrangements in *CACTA* transposases, we compared the boundaries from the 33 transposases containing 73 introns that were not removed in the trimming process (Table 1, Figure 1). We identified 3 regions, labeled I to III, in the MSA, which contain 63 out of the 73 boundaries (Figure 1). Outside those regions, we identified eight boundaries inside the DDE motif, four boundaries between Regions I and II, one boundary between Regions II and III and five boundaries downstream of Region III. Most boundaries are close to each other but not in the same position on the alignment. This can be due to small errors introduced by calculating the MSA or consensus sequences. Therefore, we analyzed the distances between boundaries to identify which were shared among transposases.

We analyzed the boundaries by clustering them based on their positions on the MSA. We set the maximal distance between boundaries still considered to be in the same region to 16 residues, which is half the length of the shortest intron annotated (33 amino acids in ATENSPM_Athal_3_). Boundaries that were closer than 16 residues to each other were grouped together. No boundaries within a region were further than 16 residues apart (Tables 2, 3, Additional files 4, 5, 6). The distances between the closest boundaries of Regions I and II is 98 residues (Additional file 7), but 30 residues between Region II and III (Additional file 7). The closest boundary upstream of Region I is 60 residues away, whereas the closest boundary downstream of Region III is 36 residues away. This clustering confirmed the previously identified regions as clearly distinct. The four boundaries EnSpm10_Fves_1_, Dario_2_, Aron_1_, and ATENSPM6_Athal_1_ between Region I and II, as well as I_2_ between Region II and III could not be clustered in those Regions. We identified only one additional cluster containing four boundaries outside Regions I to III. It groups the first introns from all members of Clade γ and was therefore named Region G.

**Table 2:** Distances between exon/intron boundaries within Region I

	**Baldur**_**1**_											
Baron_2_	1	Baron_2_										
C_1_	11	10	C_1_									
Chester_2_	5	4	6	Chester_2_								
EnSpm2_Mdom_1_	0	1	11	5	EnSpm2_Mdom_1_							
EnSpm3_Fves_2_	1	0	10	4	1	EnSpm3_Fves_2_						
I_1_	0	1	11	5	0	1	I_1_					
K_1_	0	1	11	5	0	1	0	K_1_				
Korbin_2_	0	1	11	5	0	1	0	0	Korbin_2_			
Sandro_1_	0	1	11	5	0	1	0	0	0	Sandro_1_		
Seamus_1_	0	1	11	5	0	1	0	0	0	0	Seamus_1_	
Sherman_1_	0	1	11	5	0	1	0	0	0	0	0	Sherman_1_
Storm_1_	0	1	11	5	0	1	0	0	0	0	0	0

Distances between exon/intron boundaries in the MSA within Region I (depicted in Figure 1). The distances are given in residues in the alignment.

**Table 3:** Distances between exon/intron boundaries within Region III

	**Alfred**_**1**_													
Balduin_2_	5	Balduin_2_												
Baron_4_	0	5	Baron_4_											
Chester_4_	5	10	5	Chester_4_										
En1_1_	0	5	0	5	En1_1_									
EnSpm13_Vvin_2_	0	5	0	5	0	EnSpm13_Vvin_2_								
EnSpm3_Vvin_2_	0	5	0	5	0	0	EnSpm3_Vvin_2_							
EnSpm5_Vvin_2_	0	5	0	5	0	0	0	EnSpm5_Vvin_2_						
Isaac_2_	0	5	0	5	0	0	0	0	Isaac_2_					
Isidor_2_	5	10	5	0	5	5	5	5	5	Isidor_2_				
Norman_1_	4	9	4	1	4	4	4	4	4	1	Norman_1_			
Radon_2_	4	9	4	1	4	4	4	4	4	1	0	Radon_2_		
Rufus_2_	4	9	4	1	4	4	4	4	4	1	0	0	Rufus_2_	
Sandro_2_	3	2	3	8	3	3	3	3	3	8	7	7	7	Sandro_2_
Seamus_3_	0	5	0	5	0	0	0	0	0	5	4	4	4	3

Distances between exon/intron boundaries in the MSA within Region III (depicted in Figure 1). The distances are given in residues in the alignment.

Based on these analyses of distances between all boundaries, we established that Regions I to III and G in the MSA were clearly separated from each other as well as from all other boundaries. Given the distinctness of the four boundary regions, we examined if the boundaries themselves were conserved among the analyzed transposases.

### Boundaries in Regions I to III are conserved among most transposases while Region G represents putative intron gain

Due to the proximity of boundaries in Regions I to III and their clear separation from other boundaries, we established that boundaries within a region are shared between the different transposases. The clustering of boundaries within Regions I to III indicates that the boundaries are conserved among the analyzed transposases. This is supported by the phylogenetic tree, in which purely monocotyledonous or eudicotyledonous clades share boundaries (Figure 1). Boundaries in Region I are on, or close to, the position of the conserved E from the DDE motif, supporting the claim that Region I represents conserved boundaries among the transposases (Figure 1). Therefore, we considered the 63 boundaries in Regions I to III as conserved within each region. All transposases in Clade γ share their first introns with a maximum distance of five residues (Figure 1, Table 4). This is a unique cluster in the whole tree, indicating intron gain since all members of Clade γ share this intron but none of its ancestor nodes and transposases in other clades.

**Table 4:** Distances between exon/intron boundaries within Cluster G

	**Baron**_**1**_		
Chester_1_	5	Chester_1_	
EnSpm12_Fves_1_	4	1	EnSpm12_Fves_1_
Korbin_1_	1	4	3

Distances between exon/intron boundaries in the MSA within Cluster G (depicted in Figure 1). The distances are given in residues in the alignment.

### Only two boundaries from a monocotyledonous host are found outside Regions I to III

We identified 17 boundaries outside Regions I to III (Figure 1). Only J_1_ and H1 are from a monocotyledonous host, whereas the remaining 15 boundaries were annotated in transposases from eudicotyledonous hosts. Boundaries I_1_ and ATENSPM6_1,2,3_ cannot be clustered and therefore were not further characterized. The transposases Horace, Dario, and Aron have three separate boundaries which are not farther apart than six residues: Horace_1_ and Dario_1_, Daron_2_ and Aron_1_, Horace_2_ and Aron_3_. While this appears as another case of intron gain, their relation in the phylogenetic tree is not properly resolved and does not support this interpretation.

Our analysis of the boundaries identified 63 conserved boundaries and 4 cases of putative intron gain in Region G. Most conserved introns were identified in transposases from monocotyledonous hosts. In contrast, all unique boundaries except two were identified in eudicotyledonous hosts. We decided to combine the results of the phylogenetic and boundary analyses to develop a model to understand how the observed exon/intron configuration evolved.

### Defining consensus exon numbers for each phylogenetic clade

**Figure 3:**
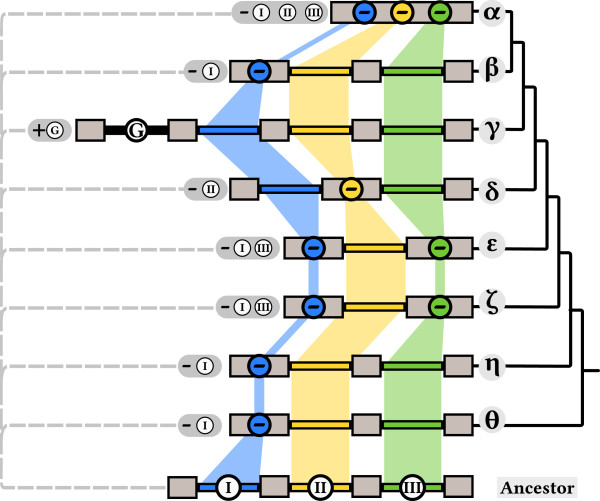
**Model for the loss and gain of introns in *CACTA *transposases.** Simplified phylogenetic tree based on the consensus exon numbers per clade as described in the text. Below the tree the putative ancestor transposase with four exons is depicted. Exons are depicted as gray rectangles with introns as colored lines. Blue, red and green depict introns conserved in Regions I to III, G indicates cluster G with the putative intron gain. Conserved introns share the same color band. Intron loss is depicted by its corresponding color and circled −, intron gain by an encircled +. Gray balloons indicate how the observed configuration arose from the putative ancestor.

A comparison of the phylogenetic tree and the conserved boundaries revealed a high consistency between clades and boundary positions. Based on the majority of exons per clade, we constructed a loose consensus to represent the exon number for transposases in the corresponding clade. For example, Clade ζ groups together seven transposases of which four, the majority, have two exons. Therefore, a representative transposase from Clade ζ has two exons and one consensus boundary. We used this approach for each clade (Figure 3). Our approach resulted in following exon numbers for representative transposases: one exon for Clade α; Clades β, δ, and θ three exons each; Clade η four exons; Clade γ five exons. Designating consensus exon numbers for each clade simplified further the analysis to develop a model for the loss and gain of boundaries in *CACTA* transposases.

### A model for loss and gain of exon/intron boundaries in *CACTA* transposases

Because it had the largest number of confirmed exons, we compared all consensus boundaries to Clade γ (Figure 3). Clade α has no annotated introns. The second, third, and fourth intron of Clade γ can be found throughout the phylogenetic tree, whereby the third intron of Clade γ is the most conserved, followed by its fourth and second intron. The fourth intron of Clade γ is found among Clades β, θ, ι, and in Isaac. The third intron is missing in the Clades EnSpm8, δ, and θ, but otherwise is found in all clades containing introns. The second intron of Clade γ is present in Clades δ, EnSpm8, and η. This comparison indicates that *CACTA* transposases were as a whole losing rather than gaining introns. However, Clades γ and ζ have introns that are not found in other clades (Figure 3), the first intron in Clade γ representing an intron gain. The unique introns in Clade ζ cannot be classified as losses or gains because the phylogenetic tree does not allow a definitive classification.We propose that the consensus transposase in Clade γ represents the most likely exon/intron configuration of an ancient transposase, containing at least four exons and three introns (Figure 3). The three boundaries correspond to those identified in Regions I to III in the MSA (Figures 1, 3). Using the putative ancestor model transposase, we can infer the emergence of the known transposases through intron loss and gain (Figure 3).

## Discussion

In sum, we analyzed 64 *CACTA* transposases from 11 monocotyledonous and eudicotyledonous hosts. Our phylogenetic analysis indicates divergence of ancient *CACTA* lineages already before the divergence of the monocotyledons and eudicotyledons. The analysis of 73 boundaries across 33 transposases with more than one exon identified 55 conserved exon/intron boundaries and allowed us to reconstruct the exon/intron configuration of a *CACTA* transposase representing the ancestral state before the divergence of monocotyledonous and eudicotyledonous plants. The model consists of at least four exons. We propose a mechanism for the evolution of the extant *CACTA* transposases in which they were shaped mainly by intron loss, although one case of putative intron gain was found.

### Potential for greater regulation of *CACTA* elements in eudicotyledons

Studies of the *P* Element in *Drosophila* and *Ac/Ds* in maize have shown that alternative splicing can regulate tissue-specific transposition of elements. For example, the *P* element retains its third intron in somatic cells, inhibiting transposition [33,34]. Should this occur with *CATCA* transposases as well, our data suggests that elements in dicotyledonous hosts have more possibilities for regulation. Interestingly, most non-clustered boundaries and the putative intron gain cluster were found in transposases from dicotyledonous hosts, whereas the majority of boundaries in Regions I to III were found in transposases from monocotyledonous hosts. The number of transposable elements in eudicotyledonous genomes is generally lower than in monocotyledonous genomes, consistent with a tighter control of transposable elements in eudicotyledonous hosts. Therefore, the large number of unique boundaries found outside Regions I to III could be associated with more control of expression of *CACTA* elements in eudicotyledons than in monocotyledons.

### Differences in intron gain and loss among TE transposases

Previously, intron gain and loss in transposases of DNA transposable elements was studied for *Mariner*-like elements in flowering plants [35]. In that study, degenerate primers were used to extract fragments of DDE transposases from 54 plant species for phylogenetic analysis. The results were consistent with vertical transmission and rapid diversification and indicated a gain of introns in grasses in a localized region of the transposase gene. This may indicate that *Mariner*-like elements generally tend to gain introns, while *CACTA* elements tend to intron loss. However, the *Mariner* fragments analyzed were mainly located within the DDE motif, where exon/intron boundaries have been predicted, whereas our data suggests that most exon/intron boundaries in DDE transposases from *CATCA* elements are downstream of that motif.

### Horizontal transfer of *CACTA* elements

We observed several transposases from distinct species grouping in the same clade such as EnSpm2_Fves in Clade α and EnSpm3_Fves and EnSpm4_Fves in Clade ζ. This raises the question of a possible horizontal mode of inheritance, which has been proposed to drive genomic variation in eukaryotic genomes and has been shown for the *Mu*-like elements in plants [36,37]. Experiments that introduced the *Ac/Ds* element from maize into *A. thaliana* and sugar beet found reduced levels of correctly spliced *Ac* transposase transcripts in those distant heterologous host species. Therefore, it has been proposed that intron loss in the transposases of DNA transposons is an adaptation to ease horizontal transfer [36]. Although the ML tree from our analysis clusters transposases from different host together, the closest relations are mainly from the same host (Additional file 3). Some exceptions are found, mostly where transposases from maize, sorghum, wheat and *B. distachyon* are found as closest neighbors. Interestingly, those close neighbors have a very similar exon/intron boundary configuration, for example, G and Balduin in Clade η, Sandro and K in Clade δ, and Oswald and EnSpm11_Sbic in Clade α. Because we did our analysis on consensus protein sequences, analysis on the DNA level as performed earlier [37] was not possible. Therefore, although horizontal transposon transfer for *CACTA* elements cannot be ruled out, our dataset does not provide support for this mechanism.

### Using several data sources increases fidelity of the annotated exon/intron boundaries

To counter the various influences of consensus sequences, we used GUIDANCE. The identification of weak regions and residues in the MSA using confidence scores improves subsequent analysis [30]. We decided to apply a threshold lower than the default, 0.804 compared to 0.93, because the boundary annotations are based on predictions and modeling approaches. Certain boundaries may have been wrongly predicted or modeled because transcription data for *CACTA* transposases is scarce. Analyses for the Triticeae have shown only seven putative transcribed transposases out of 41 identified *CACTA* elements [10]. Nevertheless, the range of annotated exons in the transposases is similar for the previously published *CACTA* transposons. OsESI1 and Hipa in rice have four exons [23], although studies in maize indicate transposases with up to eleven exons [2,24].

We used three sources to collect transposes: PTREP, Repbase, and our own models for the transposases annotated in *B. distachyon*. The majority of annotated boundaries were found in three Regions, I to III. In several cases, the boundary predictions overlapped. Annotated boundaries in Region II were derived from Repbase, our own modeling and from PTREP. This overlap strongly supports the proper annotation of an exon/intron boundary at those positions. The unique boundaries are missing such support and have, therefore, not been classified because there was not enough data to assess if they represent a putative conserved boundary or recent intron gain or loss.

An alternative explanation for the presence of conserved introns at similar positions is intron sliding or slippage. Intron sliding is defined as the shift of an exon/intron position over time during evolution, such as through nucleotide insertions before the boundary [38,39]. Calculations have shown that changes of one to 15 nucleotides may occur; shifts of one nucleotide have been observed [39]. We calculated a maximum distance of seven amino acids, which is very close to the proposed maximum of intron slippage, supporting our claim of conserved boundaries in those regions.

### High *CACTA* diversity existed already in the ancestor of monocotyledons and eudicotyledons

Our phylogenetic reconstruction clustered the transposases according to their exon number rather than by host species. This supports earlier studies, which compared intron gain and loss across several eukaryotic species and showed the evolutionary conservation of intron positions and their use as additional sources of phylogenetic information [40-42]. All clades contained a mixture of several host species, although Clade θ harbored only transposases from eudicotyledonous hosts. The monocotyledonous and eudicotyledonous hosts in all clades diverged approximately 120 to 340 million years ago [43]. This supports the existence of diversity among *CACTA* transposases already in the common ancestor of the monocotyledons and eudicotyledons.

### The ancestral *CACTA* transposase likely had four exons

The number of exons in the transposases varies between species. Our analysis of boundaries between the transposases showed that 55 out of 73 exon/intron boundaries are conserved between 2 or more transposases. This raises the question of whether the ancestral transposase, which predated the divergence of the clades that we analyzed contained one exon and later gained additional exons or instead contained several exons and then lost them over time. A third alternative is a mixture of both mechanisms, in which exons are arbitrarily gained and lost. In most transposases, we annotated between two and six exons. The conservation of the boundaries in Regions I to III across several clades indicates a loss of introns in *CACTA* transposases rather than a gain.

Boundaries in Region I have the least conservation level among the boundaries analyzed. However, these boundaries were mapped on, or close to, the E of the DDE motif. Because this motif is considered to be highly conserved and from a common origin [22], the boundaries in Region I are very likely to have been generally conserved but lost in some transposases. Nevertheless, unique introns indicate that intron gain may occur, albeit at a low frequency. The putative intron gain in Clade γ is supported by its unique occurrence, whereas the conserved boundaries are found in Regions I to III and in several clades. This is in accordance with observations of ancestral introns in plants, fungi, and animals [44].

Taking these lines of evidence into account, we propose an ancestral *CACTA* transposase configuration with at least four exons. Subsequent and differential intron loss was a major force in *CACTA* transposase evolution. Our prediction is that the ancestor *CACTA* transposase with four exons predates the divergence of monocotyledons and eudicotyledons. Given the ancestry and abundance of DDE transposases, the *CACTA* transposases appear to follow the model of ‘many introns early in eukaryotic evolution’ [38,45,46].

### Potential selection for intron gain

Against a background of general intron loss, we observed only one conspicuous case of intron gain, that of the first intron in Clade γ, where the intron is found within the entire clade. This clade contains *A. thaliana* and strawberry as hosts. Other introns were found outside Regions I to III, particularly in Clade θ, but are not present throughout an entire clade. These others are either remnants of an intron that was gained at the root of the clade, but then differentially lost in various families within the clade, or alternatively represent later insertions on the family level. Our dataset cannot resolve these alternatives. Moreover, the boundaries are based on models; a wrong prediction cannot be excluded. Due to the sparse number and weak support for introns with spotty distributions, we eliminated them from the analysis. Intron gain has been proposed to occur through the insertion of TEs and subsequent loss of TE mobility [33,47]. However, we did not identify TEs in *CACTA* transposase introns.

Interestingly, the putative gained intron in Clade γ represents the first intron, which is the one nearest the N-terminus. Studies in both eudicots and monocots suggest that first introns in particular have roles either as enhancers or in controlling the tissue specificity of expression [48-50]. Introns in *A. thaliana* have been shown to increase expression best when near the promoter [48] and to have the capacity for mediating differential expression patterns [51,52]. Therefore, intron gain at the first position in *A. thaliana* transposases may well have constituted an advantage. Although first introns have regulatory roles in monocots as well, we found no clade-wide examples of gain and retention of new transposase introns.

### Intron loss in *CACTA* transposase was reverse transcriptase -mediated

Loss of introns in the analyzed transposase genes occurred in-frame, because putative functional ORFs have been identified. Therefore, intron loss in *CACTA* transposases most likely did not influence the coding capability of the transposases. We observed only small perturbations in the alignment where introns were lost in Region I, while Regions II and III show larger disturbances at positions of intron loss. The most commonly postulated means for intron loss are by reverse transcription of spliced transcripts, by direct genomic deletion, by intron removal as a result of double strand break (DSB) repair, and by exonization.

Exonization may occur if a donor splice site is mutated so that an intron is retained in the transcript [53,54]. This would lead to a fusion of the intron with its flanking exons and therefore the shifting of an annotated boundary in the MSA. Only unique boundaries could represent an intron lost by exonization. However, unique boundaries were annotated in highly similar blocks in the MSA, indicating no gain of sequence (Figure 1). If exonization has been responsible for intron loss, it would follow that *CACTA* transposases may undergo alternative splicing, similar to the *P* element in *Drosophila* or to *Ac/Ds* in maize. [33,34,55]. Intron loss by DSB repair [56] first requires a DSB, initiated either by excision of a mobile element such as a DNA transposon or by other means. However, no mobile elements have been identified in the transposase introns, making intron loss due to DSB repair unlikely. Evidence for a DSB initiated by other means was not found, but the DSB repair model cannot be excluded. Direct genomic deletion may lead to in-frame loss of introns if small direct repeats are present at the intron ends [25,57].

Intron loss by the action of reverse transcriptase (RT) is a frequently proposed model [58-61]. The mechanism comprises reverse transcription of processed or partially processed mRNA into cDNA and subsequent integration of the cDNA into the genome by homologous recombination [44,62,63]. This mechanism can lead either to loss of all introns, as suggested for gene *EP-1α* in the zooplankton *Oikopleura longicauda*[62], or to partial loss of introns as proposed in the *catalase 3* genes in *Z. mays*[63]. A modification of the RT model has been proposed to explain the partial loss of introns, in which enzymes that recognize and degrade aberrant DNA generate fragments from the cDNA [57]. These fragments then would recombine with genomic DNA. Alternatively, selective and precise in-frame loss of introns in the *str* gene family of *Caenorhabditis briggsae* and *C. elegans* was proposed to be due to a non-homologous recombination mechanism [64].

In the *CACTA* transposases, the phylogenetically close relationship of Clade α to Clades β and γ indicates a loss of all introns (Figure 2) as the simple RT-mediation model would predict. Similarly, in several clades transposases with one exon are grouped together with transposases containing several exons (Figure 2). Therefore, loss of all introns in a *CACTA* transposase was not a unique event; it has occurred several times in different clades. Moreover, Clade α consists of eighteen transposases from all five monocotyledonous hosts and the one transposase from soybean. This indicates no species specificity exists for transposases with one exon. Moreover, intron loss due to DSB repair, intron retention, or genomic deletion would target individual elements. In contrast, in RT-mediated intron loss, the reverse transcribed transposases could undergo homologous recombination with highly similar regions such as the DDE motif that is also found in a variety of other transposases. Plants, especially grasses, are known to have high numbers of retroelements, providing the potential for RT to interact with transcripts from *CACTA* transposases [65]. Taking these strands together, it appears that RT-mediation is the most likely pathway for intron loss in *CACTA* transposases and possibly in DNA transposon transposases as a whole.

### Intron loss and gain in transposases and genes indicates transposases are ancient genomic components

Evolution of the *CACTA* transposase gene structure has parallels to that of the GDSL-lipase gene family [66]. By analysis of intron gain and loss across several land plants, it appears that the common ancestor of this gene family contained six exons. Through gain and loss of introns, different subfamilies arose, some containing unique introns. Intron loss in GDSL-lipase genes was prevalent in grasses, especially in sorghum. By contrast, in the widely distributed regulatory SnRK2 kinase family, monocots and eudicots are distinct regarding their patterns of intron retention, with the rice genes retaining more introns than those in *Arabidopsis*[67]. Most *CACTA* transposases without introns were found in sorghum, although this may merely represent sampling error. Independent loss of introns has been reported as well for the *4f-rnp* genes in *Drosophila melanogaster*[68]. The similar trajectories followed by both different gene families and the *CACTA* transposases indicates that intron gain and loss in transposases has been driven by the same evolutionary mechanisms in TEs and in genes for various cellular functions. This is consonant with the view of transposable elements as ancient genomic components and not genome ‘invaders’ [69].

## Conclusion

The presented analysis and comparison of exon/intron boundaries among 64 *CACTA* elements from monocotyledonous and eudicotyledonous hosts gives an insight into the dynamics of intron loss and gain in eukaryotic transposases in general and *CACTA* transposases in detail. Our results explain the observed variety in intron numbers among *CACTA* elements found in monocotyledonous and dicotyledonous and possibly further diverged hosts. The observed predominant loss of introns in *CACTA* transposases differs from previous studies in *Mariner*-like elements, indicating differences of intron gain and loss between DNA transposons. Our study strongly indicates a high variety among *CACTA* transposases before the divergence of monocotyledons and eudicotyledons hosts and provides a putative *CACTA* transposase configuration for the corresponding ancestor element. Our results support the view of transposable elements as genomic components and not as genome ‘invaders’. However, to fully understand intron loss and gain in *CACTA* elements, or in DNA transposon in general, reliable transcription data will be required.

## Materials and methods

### Transposase selection

Transposase sequences from *O. sativa*, *T. aestivum*, *S. bicolor, Z. mays*, *A. thaliana*, *P. hybrida*, *F. vesca*, *M. domestica,* and *V. vinifera* were extracted from Repbase and PTREP, respectively, according to criteria described in the text. *CACTA* elements are described as *EnSpm*-like elements in Repbase while DTC in PTREP. *B. distachyon CACTA* consensus sequences were taken from [18] and annotated as described in the text.

### Annotation of exon positions

For Repbase entries stored in the EMBL file format, we extracted the exon coordinates and transformed them from nucleotide positions into amino acid positions relative to the beginning of the predicted transposase protein. PTREP entries which stored protein sequences in the FASTA format were translated into DNA and aligned against the DNA consensus sequence of the corresponding *CACTA* element using dotter [70]. Despite the existence of multiple codons for each amino acid, exons could be visually recognized and annotated.

### Multiple sequence alignments and GUIDANCE

To obtain the multiple sequence alignment and confidence scores the GUIDANCE web server (http://guidance.tau.ac.il, [71]) was used with following parameters: algorithm, GUIDANCE; number of bootstrap repeats, 100; multiple sequence alignment algorithm, MAFFT; advanced alignment options, maxiterate 1000; refinement strategy, genafpair. Perl scripts were written to extract and visualize data from GUIDANCE.

### Generation of phylogenetic trees

All phylogenetic trees were calculated using RAxMLversion 7.2.8 [32]. For the meaning of the used parameter and correct calling of RAxML, we referred to the RAxML manual. The PROTGAMMALGF protein substitution model was selected using the Perl script to identify the best protein substitution model provided on the RAxML website (http://sco.h-its.org/exelixis/web/software/raxml/index.html). Construction of the ML tree was made using following parameters: -m PROTGAMMALGF, -f d, -N 200. Bootstrap analysis was carried out using following parameters: -m PROTGAMMALGF, -f d, -x 54321, -N 1000. The consensus tree was computed using following parameters: -m PROTGAMMALGF, -J MR. Testing of outgroups was performed using following parameters: -f d -m PROTGAMMALGF -N 50 -o < outgroup>. Phylogenetic trees were prepared using FigTree (http://tree.bio.ed.ac.uk/software/figtree/) and TreeGraph [72].

### Exon/intron boundary analysis

Various Perl scripts were written to analyze and visualize boundary data. All Perl programs can be obtained from the authors.

## Abbreviations

DSB: double-strand break; ML: maximum likelihood; MSA: multiple sequence alignment; ORF: open reading frame; RT: reverse transcriptase; TE: transposable element; TIR: terminal inverted repeat; TSD: target site duplication.

## Competing interests

The authors declare that they have no competing interests.

## Authors’ contributions

JPB and TW conceived the study, JPB and AL performed the analyses, AHS contributed to the interpretation; JPB, AL, TW and AHS wrote the manuscript. All authors read and approved the final manuscript.

## Authors’ information

Co-senior authors: Thomas Wicker and Alan H Schulman.

## Supplementary Material

Additional file 1 **Table summarizing the analyzed transposases.** Contains the names, length, and number of exons, host, and source for each analyzed transposase. Contains all annotated boundaries with positions on the original protein, on the trimmed MSA, its score and the residue. Click here for file

Additional file 2 **GUIDANCE results.** Contains all files to recreate the analyzed MSA and consists of three files: msa_initial.fasta, the sequence alignment derived from GUIDANCE in FASTA format; msa_residueScores.txt, GUIDANCE scores for all residues; guidance output in HTML format. Click here for file

Additional file 3 **Best maximum likelihood tree for the 57 analyzed** ***CACTA*** **transposases.** Describe s the best maximum likelihood tree out of 200 distinct, randomized, maximum parsimony trees for the 64 analyzed *CACTA* transposases. The tree has been mid-point rooted due to the lack of an available outgroup. Contains the 12 maximum likelihood trees in the Newick format which were used to check the robustness of the initial maximum likelihood tree. It can be opened using most modern phylogenetic programs. Click here for file

Additional file 4 **Distances between exon/intron boundaries within Region I.** Contains a table with distances for all exon/intron boundaries within Region I depicted in Figure 1. The distances are given as residues on the MSA. Click here for file

Additional file 5 **Distances between exon/intron boundaries within Region II.** Contains a table with distances for all exon/intron boundaries within Region II depicted in Figure 1. The distances are given as residues on the MSA. Click here for file

Additional file 6 **Distances between exon/intron boundaries within Region III.** Contains a table with distances for all exon/intron boundaries within Region III depicted in Figure 1. The distances are given as residues on the MSA. Click here for file

Additional file 7 **Distances between all analyzed exon/intron boundaries.** Contains a table with all distances between all analyzed exon/intron boundaries in the analyzed MSA. The distances are given as residues on the MSA. Click here for file
