# Supplementary material for: Analysis of CACTA transposases reveals intron loss as major factor influencing their exon/intron structure in monocotyledonous and eudicotyledonous hosts
Source: Mob DNA. 2014 Sep 1;5:24. doi: 10.1186/1759-8753-5-24 (PMC4158355; doi:10.1186/1759-8753-5-24)
Supplement: Additional file 1 — Table summarizing the analyzed transposases. Contains the names, length, and number of exons, host, and source for each analyzed transposase. Contains all annotated boundaries with positions on the original protein, on the trimmed MSA, its score and the residue. [file 1759-8753-5-24-S1.zip › Additional_File_1.pdf]

Additional File 1: The 57 *CACTA* transposases analyzed in the study.
id: name of *CACTA* transposase; exons: number of exons in the transposase; length: length of the transposases protein; host: host of the element and transposase, respectively; source: source of the analyzed element and transposase annotation; identifier: PTREP or Repbase identifier; $^{3'}$: transposase was annotated at the 3' end of the *CACTA* element.

id	exons	length[aa]	host	source	identifier
Horace	3	1076	*A. thaliana*	PTREP	PTREP187
Aron	4	1122	.	PTREP	PTREP157
Dario	4	1028	.	PTREP	PTREP174
Baron	5	1087	.	PTREP	PTREP162
Chester	5	1055	.	PTREP	PTREP168
Korbin	5	1058	.	PTREP	PTREP198
ATENSPM6	6	1158	.	Repbase	ATENSPM6
PSL	1	552	*P. hybrida*	Repbase	AF009516
EnSpm10_Fves	2	1105	*F. vesca*	Repbase	EnSpm-10_FV
EnSpm12_Fves	2	759	.	Repbase	EnSpm-12_FV
EnSpm3_Fves	4	1204	.	Repbase	EnSpm-3_FV
EnSpm4_Fves	4	4785	.	Repbase	EnSpm-4_FV
EnSpm8_Fves	6	1253	.	Repbase	EnSpm-8_FV
EnSpm2_Mdom	3	1217	*M. domestica*	Repbase	EnSpm-2_Mad
EnSpm13_Vvin	3	1131	*V. vinifera*	Repbase	EnSpm-13_VV
EnSpm3_Vvin	3	3397	.	Repbase	EnSpm-3_VV
EnSpm5_Vvin	3	1133	.	Repbase	EnSpm-5_VV
EnSpm2_Gmax	1	1064	*Glycine max*	Repbase	EnSpm-2_GM
A	1	1146	*B. distachyon*	Model	
B	1	1136	.	Model	
D	1	1146	.	Model	
E	1	1118	.	Model	
C	2	818	.	Model	
F	2	1067	.	Model	
G	2	1095	.	Model	
H	2	1038	.	Model	
I	3	1146	.	Model	
J	3	1001	.	Model	
K	3	1086	.	Model	
Calvin	1	1110	*O. sativa*	PTREP	PTREP164
Dorian	1	1114	.	PTREP	PTREP178
Eric	1	1053	.	PTREP	PTREP182
Grover	1	1095	.	PTREP	PTREP184
Janus	2	1082	.	PTREP	PTREP194
Baldur	3	1139	.	PTREP	PTREP160
Isidor$^{3'}$	3	1144	.	PTREP	PTREP190
Radon$^{3'}$	3	1139	.	PTREP	PTREP206
Rufus$^{3'}$	3	1151	.	PTREP	PTREP208
Sandro$^{3'}$	3	1069	.	PTREP	PTREP210
Sherman	3	1122	.	PTREP	PTREP213
Storm	3	1118	.	PTREP	PTREP215
Seamus	4	1138	.	PTREP	PTREP211
Caspar	1	1105	*T. aestivum*	PTREP	PTREP166
Clifford	1	1071	.	PTREP	PTREP170
Conan	1	1139	.	PTREP	PTREP172
Isaac$^{3'}$	3	1070	.	PTREP	PTREP188
Balduin	3	1159	.	PTREP	PTREP158
Helios	1	1054	*Z. mays*	PTREP	PTREP186
Ivan$^{3'}$	1	1120	.	PTREP	PTREP192
Oswald	1	1168	.	PTREP	PTREP202
Alfred	2	1147	.	PTREP	PTREP155
En1	2	1129	.	PTREP	PTREP180
Joey	2	1092	.	PTREP	PTREP196
Norman	2	1130	.	PTREP	PTREP200
DOPPIA	3	1129	.	PTREP	PTREP176
EnSpm5_Sbic	1	744	*Sorghum bicolor*	Repbase	EnSpm5_SB
EnSpm19_Sbic	1	900	.	Repbase	EnSpm-19_SBi
EnSpm20_Sbic	1	913	.	Repbase	EnSpm-20_SBi
EnSpm12_Sbic	1	846	.	Repbase	EnSpm12_SB
EnSpm23_Sbic	1	844	.	Repbase	EnSpm-23_SBi
EnSpm22_Sbic	1	1167	.	Repbase	EnSpm-22_SBi
EnSpm11_Sbic	1	1177	.	Repbase	EnSpm11_SB
EnSpm21_Sbic	1	923	.	Repbase	EnSpm-21_SBi
EnSpm8_Sbic	2	976	.	Repbase	EnSpm8_SB
