# Supplementary material for: Analysis of CACTA transposases reveals intron loss as major factor influencing their exon/intron structure in monocotyledonous and eudicotyledonous hosts
Source: Mob DNA. 2014 Sep 1;5:24. doi: 10.1186/1759-8753-5-24 (PMC4158355; doi:10.1186/1759-8753-5-24)
Supplement: Additional file 2 — GUIDANCE results. Contains all files to recreate the analyzed MSA and consists of three files: msa_initial.fasta, the sequence alignment derived from GUIDANCE in FASTA format; msa_residueScores.txt, GUIDANCE scores for all residues; guidance output in HTML format. [file 1759-8753-5-24-S2.zip › guidance.html]

# MSA color-coded by GUIDANCE scores

|  |  |  |  |  |  |  |  |  |  |  |  |  |  |  |  |  |  |  |  |  |  |  |  |  |  |  |  |  |  |  |  |  |  |  |  |  |  |  |  |  |  |  |  |  |  |  |  |  |  |  |  |  |  |  |  |  |  |  |  |  |  |  |  |  |  |  |  |  |  |  |  |  |  |  |  |  |  |  |  |  |  |  |  |  |  |  |  |  |  |  |  |  |  |  |  |  |  |  |  |  |  |  |  |  |  |  |  |  |  |  |  |  |  |  |  |  |  |  |  |  |  |  |  |  |  |  |  |  |  |  |  |  |  |  |  |  |  |  |  |  |  |  |  |  |  |  |  |  |  |  |  |  |  |  |  |  |  |  |  |  |  |  |  |  |  |  |  |  |  |  |  |  |  |  |  |  |  |  |  |  |  |  |  |  |  |  |  |  |  |  |  |  |  |  |  |  |  |  |  |  |  |  |  |  |  |  |  |  |  |  |  |  |  |  |  |  |  |  |  |  |  |  |  |  |  |  |  |  |  |  |  |  |  |  |  |  |  |  |  |  |  |  |  |  |  |  |  |  |  |  |  |  |  |  |  |  |  |  |  |  |  |  |  |  |  |  |  |  |  |  |  |  |  |  |  |  |  |  |  |  |  |  |  |  |  |  |  |  |  |  |  |  |  |  |  |  |  |  |  |  |  |  |  |  |  |  |  |  |  |  |  |  |  |  |  |  |  |  |  |  |  |  |  |  |  |  |  |  |  |  |  |  |  |  |  |  |  |  |  |  |  |  |  |  |  |  |  |  |  |  |  |  |  |  |  |  |  |  |  |  |  |  |  |  |  |  |  |  |  |  |  |  |  |  |  |  |  |  |  |  |  |  |  |  |  |  |  |  |  |  |  |  |  |  |  |  |  |  |  |  |  |  |  |  |  |  |  |  |  |  |  |  |  |  |  |  |  |  |  |  |  |  |  |  |  |  |  |  |  |  |  |  |  |  |  |  |  |  |  |  |  |  |  |  |  |  |  |  |  |  |  |  |  |  |  |  |  |  |  |  |  |  |  |  |  |  |  |  |  |  |  |  |  |  |  |  |  |  |  |  |  |  |  |  |  |  |  |  |  |  |  |  |  |  |  |  |  |  |  |  |  |  |  |  |  |  |  |  |  |  |  |  |  |  |  |  |  |  |  |  |  |  |  |  |  |  |  |  |  |  |  |  |  |  |  |  |  |  |  |  |  |  |  |  |  |  |  |  |  |  |  |  |  |  |  |  |  |  |  |  |  |  |  |  |  |  |  |  |  |  |  |  |  |  |  |  |  |  |  |  |  |  |  |  |  |  |  |  |  |  |  |  |  |  |  |  |  |  |  |  |  |  |  |  |  |  |  |  |  |  |  |  |  |  |  |  |  |  |  |  |  |  |  |  |  |  |  |  |  |  |  |  |  |  |  |  |  |  |  |  |  |  |  |  |  |  |  |  |  |  |  |  |  |  |  |  |  |  |  |  |  |  |  |  |  |  |  |  |  |  |  |  |  |  |  |  |  |  |  |  |  |  |  |  |  |  |  |  |  |  |  |  |  |  |  |  |  |  |  |  |  |  |  |  |  |  |  |  |  |  |  |  |  |  |  |  |  |  |  |  |  |  |  |  |  |  |  |  |  |  |  |  |  |  |  |  |  |  |  |  |  |  |  |  |  |  |  |  |  |  |  |  |  |  |  |  |  |  |  |  |  |  |  |  |  |  |  |  |  |  |  |  |  |  |  |  |  |  |  |  |  |  |  |  |  |  |  |  |  |  |  |  |  |  |  |  |  |  |  |  |  |  |  |  |  |  |  |  |  |  |  |  |  |  |  |  |  |  |  |  |  |  |  |  |  |  |  |  |  |  |  |  |  |  |  |  |  |  |  |  |  |  |  |  |  |  |  |  |  |  |  |  |  |  |  |  |  |  |  |  |  |  |  |  |  |  |  |  |  |  |  |  |  |  |  |  |  |  |  |  |  |  |  |  |  |  |  |  |  |  |  |  |  |  |  |  |  |  |  |  |  |  |  |  |  |  |  |  |  |  |  |  |  |  |  |  |  |  |  |  |  |  |  |  |  |  |  |  |  |  |  |  |  |  |  |  |  |  |  |  |  |  |  |  |  |  |  |  |  |  |  |  |  |  |  |  |  |  |  |  |  |  |  |  |  |  |  |  |  |  |  |  |  |  |  |  |  |  |  |  |  |  |  |  |  |  |  |  |  |  |  |  |  |  |  |  |  |  |  |  |  |  |  |  |  |  |  |  |  |  |  |  |  |  |  |  |  |  |  |  |  |  |  |  |  |  |  |  |  |  |  |  |  |  |  |  |  |  |  |  |  |  |  |  |  |  |  |  |  |  |  |  |  |  |  |  |  |  |  |  |  |  |  |  |  |  |  |  |  |  |  |  |  |  |  |  |  |  |  |  |  |  |  |  |  |  |  |  |  |  |  |  |  |  |  |  |  |  |  |  |  |  |  |  |  |  |  |  |  |  |  |  |  |  |  |  |  |  |  |  |  |  |  |  |  |  |  |  |  |  |  |  |  |  |  |  |  |  |  |  |  |  |  |  |  |  |  |  |  |  |  |  |  |  |  |  |  |  |  |  |  |  |  |  |  |  |  |  |  |  |  |  |  |  |  |  |  |  |  |  |  |  |  |  |  |  |  |  |  |  |  |  |  |  |  |  |  |  |  |  |  |  |  |  |  |  |  |  |  |  |  |  |  |  |  |  |  |  |  |  |  |  |  |  |  |  |  |  |  |  |  |  |  |  |  |  |  |  |  |  |  |  |  |  |  |  |  |  |  |  |  |  |  |  |  |  |  |  |  |  |  |  |  |  |  |  |  |  |  |  |  |  |  |  |  |  |  |  |  |  |  |  |  |  |  |  |  |  |  |  |  |  |  |  |  |  |  |  |  |  |  |  |  |  |  |  |  |  |  |  |  |  |  |  |  |  |  |  |  |  |  |  |  |  |  |  |  |  |  |  |  |  |  |  |  |  |  |  |  |  |  |  |  |  |  |  |  |  |  |  |  |  |  |  |  |  |  |  |  |  |  |  |  |  |  |  |  |  |  |  |  |  |  |  |  |  |  |  |  |  |  |  |  |  |  |  |  |  |  |  |  |  |  |  |  |  |  |  |  |  |  |  |  |  |  |  |  |  |  |  |  |  |  |  |  |  |  |  |  |  |  |  |  |  |  |  |  |  |  |  |  |  |  |  |  |  |  |  |  |  |  |  |  |  |  |  |  |  |  |  |  |  |  |  |  |  |  |  |  |  |  |  |  |  |  |  |  |  |  |  |  |  |  |  |  |  |  |  |  |  |  |  |  |  |  |  |  |  |  |  |  |  |  |  |  |  |  |  |  |  |  |  |  |  |  |  |  |  |  |  |  |  |  |  |  |  |  |  |  |  |  |  |  |  |  |  |  |  |  |  |  |  |  |  |  |  |  |  |  |  |  |  |  |  |  |  |  |  |  |  |  |  |  |  |  |  |  |  |  |  |  |  |  |  |  |  |  |  |  |  |  |  |  |  |  |  |  |  |  |  |  |  |  |  |  |  |  |  |  |  |  |  |  |  |  |  |  |  |  |  |  |  |  |  |  |  |  |  |  |  |  |  |  |  |  |  |  |  |  |  |  |  |  |  |  |  |  |  |  |  |  |  |  |  |  |  |  |  |  |  |  |  |  |  |  |  |  |  |  |  |  |  |  |  |  |  |  |  |  |  |  |  |  |  |  |  |  |  |  |  |  |  |  |  |  |  |  |  |  |  |  |  |  |  |  |  |  |  |  |  |  |  |  |  |  |  |  |  |  |  |  |  |  |  |  |  |  |  |  |  |  |  |  |  |  |  |  |  |  |  |  |  |  |  |  |  |  |  |  |  |  |  |  |  |  |  |  |  |  |  |  |  |  |  |  |  |  |  |  |  |  |  |  |  |  |  |  |  |  |  |  |  |  |  |  |  |  |  |  |  |  |  |  |  |  |  |  |  |  |  |  |  |  |  |  |  |  |  |  |  |  |  |  |  |  |  |  |  |  |  |  |  |  |  |  |  |  |  |  |  |  |  |  |  |  |  |  |  |  |  |  |  |  |  |  |  |  |  |  |  |  |  |  |  |  |  |  |  |  |  |  |  |  |  |  |  |  |  |  |  |  |  |  |  |  |  |  |  |  |  |  |  |  |  |  |  |  |  |  |  |  |  |  |  |  |  |  |  |  |  |  |  |  |  |  |  |  |  |  |  |  |  |  |  |  |  |  |  |  |  |  |  |  |  |  |  |  |  |  |  |  |  |  |  |  |  |  |  |  |  |  |  |  |  |  |  |  |  |  |  |  |  |  |  |  |  |  |  |  |  |  |  |  |  |  |  |  |  |  |  |  |  |  |  |  |  |  |  |  |  |  |  |  |  |  |  |  |  |  |  |  |  |  |  |  |  |  |  |  |  |  |  |  |  |  |  |  |  |  |  |  |  |  |  |  |  |  |  |  |  |  |  |  |  |  |  |  |  |  |  |  |  |  |  |  |  |  |  |  |  |  |  |  |  |  |  |  |  |  |  |  |  |  |  |  |  |  |  |  |  |  |  |  |  |  |  |  |  |  |  |  |  |  |  |  |  |  |  |  |  |  |  |  |  |  |  |  |  |  |  |  |  |  |  |  |  |  |  |  |  |  |  |  |  |  |  |  |  |  |  |  |  |  |  |  |  |  |  |  |  |  |  |  |  |  |  |  |  |  |  |  |  |  |  |  |  |  |  |  |  |  |  |  |  |  |  |  |  |  |  |  |  |  |  |  |  |  |  |  |  |  |  |  |  |  |  |  |  |  |  |  |  |  |  |  |  |  |  |  |  |  |  |  |  |  |  |  |  |  |  |  |  |  |  |  |  |  |  |  |  |  |  |  |  |  |  |  |  |  |  |  |  |  |  |  |  |  |  |  |  |  |  |  |  |  |  |  |  |  |  |  |  |  |  |  |  |  |  |  |  |  |  |  |  |  |  |  |  |  |  |  |  |  |  |  |  |  |  |  |  |  |  |  |  |  |  |  |  |  |  |  |  |  |  |  |  |  |  |  |  |  |  |  |  |  |  |  |  |  |  |  |  |  |  |  |  |  |  |  |  |  |  |  |  |  |  |  |  |  |  |  |  |  |  |  |  |  |  |  |  |  |  |  |  |  |  |  |  |  |  |  |  |  |  |  |  |  |  |  |  |  |  |  |  |  |  |  |  |  |  |  |  |  |  |  |  |  |  |  |  |  |  |  |  |  |  |  |  |  |  |  |  |  |  |  |  |  |  |  |  |  |  |  |  |  |  |  |  |  |  |  |  |  |  |  |  |  |  |  |  |  |  |  |  |  |  |  |  |  |  |  |  |  |  |  |  |  |  |  |  |  |  |  |  |  |  |  |  |  |  |  |  |  |  |  |  |  |  |  |  |  |  |  |  |  |  |  |  |  |  |  |  |  |  |  |  |  |  |  |  |  |  |  |  |  |  |  |  |  |  |  |  |  |  |  |  |  |  |  |  |  |  |  |  |  |  |  |  |  |  |  |  |  |  |  |  |  |  |  |  |  |  |  |  |  |  |  |  |  |  |  |  |  |  |  |  |  |  |  |  |  |  |  |  |  |  |  |  |  |  |  |  |  |  |  |  |
| --- | --- | --- | --- | --- | --- | --- | --- | --- | --- | --- | --- | --- | --- | --- | --- | --- | --- | --- | --- | --- | --- | --- | --- | --- | --- | --- | --- | --- | --- | --- | --- | --- | --- | --- | --- | --- | --- | --- | --- | --- | --- | --- | --- | --- | --- | --- | --- | --- | --- | --- | --- | --- | --- | --- | --- | --- | --- | --- | --- | --- | --- | --- | --- | --- | --- | --- | --- | --- | --- | --- | --- | --- | --- | --- | --- | --- | --- | --- | --- | --- | --- | --- | --- | --- | --- | --- | --- | --- | --- | --- | --- | --- | --- | --- | --- | --- | --- | --- | --- | --- | --- | --- | --- | --- | --- | --- | --- | --- | --- | --- | --- | --- | --- | --- | --- | --- | --- | --- | --- | --- | --- | --- | --- | --- | --- | --- | --- | --- | --- | --- | --- | --- | --- | --- | --- | --- | --- | --- | --- | --- | --- | --- | --- | --- | --- | --- | --- | --- | --- | --- | --- | --- | --- | --- | --- | --- | --- | --- | --- | --- | --- | --- | --- | --- | --- | --- | --- | --- | --- | --- | --- | --- | --- | --- | --- | --- | --- | --- | --- | --- | --- | --- | --- | --- | --- | --- | --- | --- | --- | --- | --- | --- | --- | --- | --- | --- | --- | --- | --- | --- | --- | --- | --- | --- | --- | --- | --- | --- | --- | --- | --- | --- | --- | --- | --- | --- | --- | --- | --- | --- | --- | --- | --- | --- | --- | --- | --- | --- | --- | --- | --- | --- | --- | --- | --- | --- | --- | --- | --- | --- | --- | --- | --- | --- | --- | --- | --- | --- | --- | --- | --- | --- | --- | --- | --- | --- | --- | --- | --- | --- | --- | --- | --- | --- | --- | --- | --- | --- | --- | --- | --- | --- | --- | --- | --- | --- | --- | --- | --- | --- | --- | --- | --- | --- | --- | --- | --- | --- | --- | --- | --- | --- | --- | --- | --- | --- | --- | --- | --- | --- | --- | --- | --- | --- | --- | --- | --- | --- | --- | --- | --- | --- | --- | --- | --- | --- | --- | --- | --- | --- | --- | --- | --- | --- | --- | --- | --- | --- | --- | --- | --- | --- | --- | --- | --- | --- | --- | --- | --- | --- | --- | --- | --- | --- | --- | --- | --- | --- | --- | --- | --- | --- | --- | --- | --- | --- | --- | --- | --- | --- | --- | --- | --- | --- | --- | --- | --- | --- | --- | --- | --- | --- | --- | --- | --- | --- | --- | --- | --- | --- | --- | --- | --- | --- | --- | --- | --- | --- | --- | --- | --- | --- | --- | --- | --- | --- | --- | --- | --- | --- | --- | --- | --- | --- | --- | --- | --- | --- | --- | --- | --- | --- | --- | --- | --- | --- | --- | --- | --- | --- | --- | --- | --- | --- | --- | --- | --- | --- | --- | --- | --- | --- | --- | --- | --- | --- | --- | --- | --- | --- | --- | --- | --- | --- | --- | --- | --- | --- | --- | --- | --- | --- | --- | --- | --- | --- | --- | --- | --- | --- | --- | --- | --- | --- | --- | --- | --- | --- | --- | --- | --- | --- | --- | --- | --- | --- | --- | --- | --- | --- | --- | --- | --- | --- | --- | --- | --- | --- | --- | --- | --- | --- | --- | --- | --- | --- | --- | --- | --- | --- | --- | --- | --- | --- | --- | --- | --- | --- | --- | --- | --- | --- | --- | --- | --- | --- | --- | --- | --- | --- | --- | --- | --- | --- | --- | --- | --- | --- | --- | --- | --- | --- | --- | --- | --- | --- | --- | --- | --- | --- | --- | --- | --- | --- | --- | --- | --- | --- | --- | --- | --- | --- | --- | --- | --- | --- | --- | --- | --- | --- | --- | --- | --- | --- | --- | --- | --- | --- | --- | --- | --- | --- | --- | --- | --- | --- | --- | --- | --- | --- | --- | --- | --- | --- | --- | --- | --- | --- | --- | --- | --- | --- | --- | --- | --- | --- | --- | --- | --- | --- | --- | --- | --- | --- | --- | --- | --- | --- | --- | --- | --- | --- | --- | --- | --- | --- | --- | --- | --- | --- | --- | --- | --- | --- | --- | --- | --- | --- | --- | --- | --- | --- | --- | --- | --- | --- | --- | --- | --- | --- | --- | --- | --- | --- | --- | --- | --- | --- | --- | --- | --- | --- | --- | --- | --- | --- | --- | --- | --- | --- | --- | --- | --- | --- | --- | --- | --- | --- | --- | --- | --- | --- | --- | --- | --- | --- | --- | --- | --- | --- | --- | --- | --- | --- | --- | --- | --- | --- | --- | --- | --- | --- | --- | --- | --- | --- | --- | --- | --- | --- | --- | --- | --- | --- | --- | --- | --- | --- | --- | --- | --- | --- | --- | --- | --- | --- | --- | --- | --- | --- | --- | --- | --- | --- | --- | --- | --- | --- | --- | --- | --- | --- | --- | --- | --- | --- | --- | --- | --- | --- | --- | --- | --- | --- | --- | --- | --- | --- | --- | --- | --- | --- | --- | --- | --- | --- | --- | --- | --- | --- | --- | --- | --- | --- | --- | --- | --- | --- | --- | --- | --- | --- | --- | --- | --- | --- | --- | --- | --- | --- | --- | --- | --- | --- | --- | --- | --- | --- | --- | --- | --- | --- | --- | --- | --- | --- | --- | --- | --- | --- | --- | --- | --- | --- | --- | --- | --- | --- | --- | --- | --- | --- | --- | --- | --- | --- | --- | --- | --- | --- | --- | --- | --- | --- | --- | --- | --- | --- | --- | --- | --- | --- | --- | --- | --- | --- | --- | --- | --- | --- | --- | --- | --- | --- | --- | --- | --- | --- | --- | --- | --- | --- | --- | --- | --- | --- | --- | --- | --- | --- | --- | --- | --- | --- | --- | --- | --- | --- | --- | --- | --- | --- | --- | --- | --- | --- | --- | --- | --- | --- | --- | --- | --- | --- | --- | --- | --- | --- | --- | --- | --- | --- | --- | --- | --- | --- | --- | --- | --- | --- | --- | --- | --- | --- | --- | --- | --- | --- | --- | --- | --- | --- | --- | --- | --- | --- | --- | --- | --- | --- | --- | --- | --- | --- | --- | --- | --- | --- | --- | --- | --- | --- | --- | --- | --- | --- | --- | --- | --- | --- | --- | --- | --- | --- | --- | --- | --- | --- | --- | --- | --- | --- | --- | --- | --- | --- | --- | --- | --- | --- | --- | --- | --- | --- | --- | --- | --- | --- | --- | --- | --- | --- | --- | --- | --- | --- | --- | --- | --- | --- | --- | --- | --- | --- | --- | --- | --- | --- | --- | --- | --- | --- | --- | --- | --- | --- | --- | --- | --- | --- | --- | --- | --- | --- | --- | --- | --- | --- | --- | --- | --- | --- | --- | --- | --- | --- | --- | --- | --- | --- | --- | --- | --- | --- | --- | --- | --- | --- | --- | --- | --- | --- | --- | --- | --- | --- | --- | --- | --- | --- | --- | --- | --- | --- | --- | --- | --- | --- | --- | --- | --- | --- | --- | --- | --- | --- | --- | --- | --- | --- | --- | --- | --- | --- | --- | --- | --- | --- | --- | --- | --- | --- | --- | --- | --- | --- | --- | --- | --- | --- | --- | --- | --- | --- | --- | --- | --- | --- | --- | --- | --- | --- | --- | --- | --- | --- | --- | --- | --- | --- | --- | --- | --- | --- | --- | --- | --- | --- | --- | --- | --- | --- | --- | --- | --- | --- | --- | --- | --- | --- | --- | --- | --- | --- | --- | --- | --- | --- | --- | --- | --- | --- | --- | --- | --- | --- | --- | --- | --- | --- | --- | --- | --- | --- | --- | --- | --- | --- | --- | --- | --- | --- | --- | --- | --- | --- | --- | --- | --- | --- | --- | --- | --- | --- | --- | --- | --- | --- | --- | --- | --- | --- | --- | --- | --- | --- | --- | --- | --- | --- | --- | --- | --- | --- | --- | --- | --- | --- | --- | --- | --- | --- | --- | --- | --- | --- | --- | --- | --- | --- | --- | --- | --- | --- | --- | --- | --- | --- | --- | --- | --- | --- | --- | --- | --- | --- | --- | --- | --- | --- | --- | --- | --- | --- | --- | --- | --- | --- | --- | --- | --- | --- | --- | --- | --- | --- | --- | --- | --- | --- | --- | --- | --- | --- | --- | --- | --- | --- | --- | --- | --- | --- | --- | --- | --- | --- | --- | --- | --- | --- | --- | --- | --- | --- | --- | --- | --- | --- | --- | --- | --- | --- | --- | --- | --- | --- | --- | --- | --- | --- | --- | --- | --- | --- | --- | --- | --- | --- | --- | --- | --- | --- | --- | --- | --- | --- | --- | --- | --- | --- | --- | --- | --- | --- | --- | --- | --- | --- | --- | --- | --- | --- | --- | --- | --- | --- | --- | --- | --- | --- | --- | --- | --- | --- | --- | --- | --- | --- | --- | --- | --- | --- | --- | --- | --- | --- | --- | --- | --- | --- | --- | --- | --- | --- | --- | --- | --- | --- | --- | --- | --- | --- | --- | --- | --- | --- | --- | --- | --- | --- | --- | --- | --- | --- | --- | --- | --- | --- | --- | --- | --- | --- | --- | --- | --- | --- | --- | --- | --- | --- | --- | --- | --- | --- | --- | --- | --- | --- | --- | --- | --- | --- | --- | --- | --- | --- | --- | --- | --- | --- | --- | --- | --- | --- | --- | --- | --- | --- | --- | --- | --- | --- | --- | --- | --- | --- | --- | --- | --- | --- | --- | --- | --- | --- | --- | --- | --- | --- | --- | --- | --- | --- | --- | --- | --- | --- | --- | --- | --- | --- | --- | --- | --- | --- | --- | --- | --- | --- | --- | --- | --- | --- | --- | --- | --- | --- | --- | --- | --- | --- | --- | --- | --- | --- | --- | --- | --- | --- | --- | --- | --- | --- | --- | --- | --- | --- | --- | --- | --- | --- | --- | --- | --- | --- | --- | --- | --- | --- | --- | --- | --- | --- | --- | --- | --- | --- | --- | --- | --- | --- | --- | --- | --- | --- | --- | --- | --- | --- | --- | --- | --- | --- | --- | --- | --- | --- | --- | --- | --- | --- | --- | --- | --- | --- | --- | --- | --- | --- | --- | --- | --- | --- | --- | --- | --- | --- | --- | --- | --- | --- | --- | --- | --- | --- | --- | --- | --- | --- | --- | --- | --- | --- | --- | --- | --- | --- | --- | --- | --- | --- | --- | --- | --- | --- | --- | --- | --- | --- | --- | --- | --- | --- | --- | --- | --- | --- | --- | --- | --- | --- | --- | --- | --- | --- | --- | --- | --- | --- | --- | --- | --- | --- | --- | --- | --- | --- | --- | --- | --- | --- | --- | --- | --- | --- | --- | --- | --- | --- | --- | --- | --- | --- | --- | --- | --- | --- | --- | --- | --- | --- | --- | --- | --- | --- | --- | --- | --- | --- | --- | --- | --- | --- | --- | --- | --- | --- | --- | --- | --- | --- | --- | --- | --- | --- | --- | --- | --- | --- | --- | --- | --- | --- | --- | --- | --- | --- | --- | --- | --- | --- | --- | --- | --- | --- | --- | --- | --- | --- | --- | --- | --- | --- | --- | --- | --- | --- | --- | --- | --- | --- | --- | --- | --- | --- | --- | --- | --- | --- | --- | --- | --- | --- | --- | --- | --- | --- | --- | --- | --- | --- | --- | --- | --- | --- | --- | --- | --- | --- | --- | --- | --- | --- | --- | --- | --- | --- | --- | --- | --- | --- | --- | --- | --- | --- | --- | --- | --- | --- | --- | --- | --- | --- | --- | --- | --- | --- | --- | --- | --- | --- | --- | --- | --- | --- | --- | --- | --- | --- | --- | --- | --- | --- | --- | --- | --- | --- | --- | --- | --- | --- | --- | --- | --- | --- | --- | --- | --- | --- | --- | --- | --- | --- | --- | --- | --- | --- | --- | --- | --- | --- | --- | --- | --- | --- | --- | --- | --- | --- | --- | --- | --- | --- | --- | --- | --- | --- | --- | --- | --- | --- | --- | --- | --- | --- | --- | --- | --- | --- | --- | --- | --- | --- | --- | --- | --- | --- | --- | --- | --- | --- | --- | --- | --- | --- | --- | --- | --- | --- | --- | --- | --- | --- | --- | --- | --- | --- | --- | --- | --- | --- | --- | --- | --- | --- | --- | --- | --- | --- | --- | --- | --- | --- | --- | --- | --- | --- | --- | --- | --- | --- | --- | --- | --- | --- | --- | --- | --- | --- | --- | --- | --- | --- | --- | --- | --- | --- | --- | --- | --- | --- | --- | --- | --- | --- | --- | --- | --- | --- | --- | --- | --- | --- | --- | --- | --- | --- | --- | --- | --- | --- | --- | --- | --- | --- | --- | --- | --- | --- | --- | --- | --- | --- | --- | --- | --- | --- | --- | --- | --- | --- | --- | --- | --- | --- | --- | --- | --- | --- | --- | --- | --- | --- | --- | --- | --- | --- | --- | --- | --- | --- | --- | --- | --- | --- | --- | --- | --- | --- | --- | --- | --- | --- | --- | --- | --- | --- | --- | --- | --- | --- | --- | --- | --- | --- | --- | --- | --- | --- | --- | --- | --- | --- | --- | --- | --- | --- | --- | --- | --- | --- | --- | --- | --- | --- | --- | --- | --- | --- | --- | --- | --- | --- | --- | --- | --- | --- | --- | --- | --- | --- | --- | --- | --- | --- | --- | --- | --- | --- | --- | --- | --- | --- | --- | --- | --- | --- | --- | --- | --- | --- | --- | --- | --- | --- | --- | --- | --- | --- | --- | --- | --- | --- | --- | --- | --- | --- | --- | --- | --- | --- | --- | --- | --- | --- | --- | --- | --- | --- | --- | --- | --- | --- | --- | --- | --- | --- | --- | --- | --- | --- | --- | --- | --- | --- | --- | --- | --- | --- | --- | --- | --- | --- | --- | --- | --- | --- | --- | --- | --- | --- | --- | --- | --- | --- | --- | --- | --- | --- | --- | --- | --- | --- | --- | --- | --- | --- | --- | --- | --- | --- | --- | --- | --- | --- | --- | --- | --- | --- | --- | --- | --- | --- | --- | --- | --- | --- | --- | --- | --- | --- | --- | --- | --- | --- | --- | --- | --- | --- | --- | --- | --- | --- | --- | --- | --- | --- | --- | --- | --- | --- | --- | --- | --- | --- | --- | --- | --- | --- | --- | --- | --- | --- | --- | --- | --- | --- | --- | --- | --- | --- | --- | --- | --- | --- | --- | --- | --- | --- | --- | --- | --- | --- | --- | --- | --- | --- | --- | --- | --- | --- | --- | --- | --- | --- | --- | --- | --- | --- | --- | --- | --- | --- | --- | --- | --- | --- | --- | --- | --- | --- | --- | --- | --- | --- | --- | --- | --- | --- | --- | --- | --- | --- | --- | --- | --- | --- | --- | --- | --- | --- | --- | --- | --- | --- | --- | --- | --- | --- | --- | --- | --- | --- | --- | --- | --- | --- | --- | --- | --- | --- | --- | --- | --- | --- | --- | --- | --- | --- | --- | --- | --- | --- | --- | --- | --- | --- | --- | --- | --- | --- | --- | --- | --- | --- | --- | --- | --- | --- | --- | --- | --- | --- | --- | --- | --- | --- | --- | --- | --- | --- | --- | --- | --- | --- | --- | --- | --- | --- | --- | --- | --- | --- | --- | --- | --- | --- | --- | --- | --- | --- | --- | --- | --- | --- | --- | --- | --- | --- | --- | --- | --- | --- | --- | --- | --- | --- | --- | --- | --- | --- | --- | --- | --- | --- | --- | --- | --- | --- | --- | --- | --- | --- | --- | --- | --- | --- | --- | --- | --- | --- | --- | --- | --- | --- | --- | --- | --- | --- | --- | --- | --- | --- | --- | --- | --- | --- | --- | --- | --- | --- | --- | --- | --- | --- | --- | --- | --- | --- | --- | --- | --- | --- | --- | --- | --- | --- | --- | --- | --- | --- | --- | --- | --- | --- | --- | --- | --- | --- | --- | --- | --- | --- | --- | --- | --- | --- | --- | --- | --- | --- | --- | --- | --- | --- | --- | --- | --- | --- | --- | --- | --- | --- | --- | --- | --- | --- | --- | --- | --- | --- | --- | --- | --- | --- | --- | --- | --- | --- | --- | --- | --- | --- | --- | --- | --- | --- | --- | --- | --- | --- | --- | --- | --- | --- | --- | --- | --- | --- | --- | --- | --- | --- | --- | --- | --- | --- | --- | --- | --- | --- | --- | --- | --- | --- | --- | --- | --- | --- | --- | --- | --- | --- | --- | --- | --- | --- | --- | --- | --- | --- | --- | --- | --- | --- | --- | --- | --- | --- | --- | --- | --- | --- | --- | --- | --- | --- | --- | --- | --- | --- | --- | --- | --- | --- | --- | --- | --- | --- | --- | --- | --- | --- | --- | --- | --- | --- |
|  |  | 1 |  |  |  |  |  |  |  |  | 1 | 0 |  |  |  |  |  |  |  |  | 2 | 0 |  |  |  |  |  |  |  |  | 3 | 0 |  |  |  |  |  |  |  |  | 4 | 0 |  |  |  |  |  |  |  |  | 5 | 0 |  |  |  |  |  |  |  |  | 6 | 0 |  |  |  |  |  |  |  |  | 7 | 0 |  |  |  |  |  |  |  |  | 8 | 0 |  |  |  |  |  |  |  |  | 9 | 0 |  |  |  |  |  |  |  |  | 1 | 0 | 0 |  |  |  |  |  |  |  | 1 | 1 | 0 |  |  |  |  |  |  |  | 1 | 2 | 0 |  |  |  |  |  |  |  | 1 | 3 | 0 |  |  |  |  |  |  |  | 1 | 4 | 0 |  |  |  |  |  |  |  | 1 | 5 | 0 |  |  |  |  |  |  |  | 1 | 6 | 0 |  |  |  |  |  |  |  | 1 | 7 | 0 |  |  |  |  |  |  |  | 1 | 8 | 0 |  |  |  |  |  |  |  | 1 | 9 | 0 |  |  |  |  |  |  |  | 2 | 0 | 0 |  |  |  |  |  |  |  | 2 | 1 | 0 |  |  |  |  |  |  |  | 2 | 2 | 0 |  |  |  |  |  |  |  | 2 | 3 | 0 |  |  |  |  |  |  |  | 2 | 4 | 0 |  |  |  |  |  |  |  | 2 | 5 | 0 |  |  |  |  |  |  |  | 2 | 6 | 0 |  |  |  |  |  |  |  | 2 | 7 | 0 |  |  |  |  |  |  |  | 2 | 8 | 0 |  |  |  |  |  |  |  | 2 | 9 | 0 |  |  |  |  |  |  |  | 3 | 0 | 0 |  |  |  |  |  |  |  | 3 | 1 | 0 |  |  |  |  |  |  |  | 3 | 2 | 0 |  |  |  |  |  |  |  | 3 | 3 | 0 |  |  |  |  |  |  |  | 3 | 4 | 0 |  |  |  |  |  |  |  | 3 | 5 | 0 |  |  |  |  |  |  |  | 3 | 6 | 0 |  |  |  |  |  |  |  | 3 | 7 | 0 |  |  |  |  |  |  |  | 3 | 8 | 0 |  |  |  |  |  |  |  | 3 | 9 | 0 |  |  |  |  |  |  |  | 4 | 0 | 0 |  |  |  |  |  |  |  | 4 | 1 | 0 |  |  |  |  |  |  |  | 4 | 2 | 0 |  |  |  |  |  |  |  | 4 | 3 | 0 |  |  |  |  |  |  |  | 4 | 4 | 0 |  |  |  |  |  |  |  | 4 | 5 | 0 |  |  |  |  |  |  |  | 4 | 6 | 0 |  |  |  |  |  |  |  | 4 | 7 | 0 |  |  |  |  |  |  |  | 4 | 8 | 0 |  |  |  |  |  |  |  | 4 | 9 | 0 |  |  |  |  |  |  |  | 5 | 0 | 0 |  |  |  |  |  |  |  | 5 | 1 | 0 |  |  |  |  |  |  |  | 5 | 2 | 0 |  |  |  |  |  |  |  | 5 | 3 | 0 |  |  |  |  |  |  |  | 5 | 4 | 0 |  |  |  |  |  |  |  | 5 | 5 | 0 |  |  |  |  |  |  |  | 5 | 6 | 0 |  |  |  |  |  |  |  | 5 | 7 | 0 |  |  |  |  |  |  |  | 5 | 8 | 0 |  |  |  |  |  |  |  | 5 | 9 | 0 |  |  |  |  |  |  |  | 6 | 0 | 0 |  |  |  |  |  |  |  | 6 | 1 | 0 |  |  |  |  |  |  |  | 6 | 2 | 0 |  |  |  |  |  |  |  | 6 | 3 | 0 |  |  |  |  |  |  |  | 6 | 4 | 0 |  |  |  |  |  |  |  | 6 | 5 | 0 |  |  |  |  |  |  |  | 6 | 6 | 0 |  |  |  |  |  |  |  | 6 | 7 | 0 |  |  |  |  |  |  |  | 6 | 8 | 0 |  |  |  |  |  |  |  | 6 | 9 | 0 |  |  |  |  |  |  |  | 7 | 0 | 0 |  |  |  |  |  |  |  | 7 | 1 | 0 |  |  |  |  |  |  |  | 7 | 2 | 0 |  |  |  |  |  |  |  | 7 | 3 | 0 |  |  |  |  |  |  |  | 7 | 4 | 0 |  |  |  |  |  |  |  | 7 | 5 | 0 |  |  |  |  |  |  |  | 7 | 6 | 0 |  |  |  |  |  |  |  | 7 | 7 | 0 |  |  |  |  |  |  |  | 7 | 8 | 0 |  |  |  |  |  |  |  | 7 | 9 | 0 |  |  |  |  |  |  |  | 8 | 0 | 0 |  |  |  |  |  |  |  | 8 | 1 | 0 |  |  |  |  |  |  |  | 8 | 2 | 0 |  |  |  |  |  |  |  | 8 | 3 | 0 |  |  |  |  |  |  |  | 8 | 4 | 0 |  |  |  |  |  |  |  | 8 | 5 | 0 |  |  |  |  |  |  |  | 8 | 6 | 0 |  |  |  |  |  |  |  | 8 | 7 | 0 |  |  |  |  |  |  |  | 8 | 8 | 0 |  |  |  |  |  |  |  | 8 | 9 | 0 |  |  |  |  |  |  |  | 9 | 0 | 0 |  |  |  |  |  |  |  | 9 | 1 | 0 |  |  |  |  |  |  |  | 9 | 2 | 0 |  |  |  |  |  |  |  | 9 | 3 | 0 |  |  |  |  |  |  |  | 9 | 4 | 0 |  |  |  |  |  |  |  | 9 | 5 | 0 |  |  |  |  |  |  |  | 9 | 6 | 0 |  |  |  |  |  |  |  | 9 | 7 | 0 |  |  |  |  |  |  |  | 9 | 8 | 0 |  |  |  |  |  |  |  | 9 | 9 | 0 |  |  |  |  |  |  |  | 1 | 0 | 0 | 0 |  |  |  |  |  |  | 1 | 0 | 1 | 0 |  |  |  |  |  |  | 1 | 0 | 2 | 0 |  |  |  |  |  |  | 1 | 0 | 3 | 0 |  |  |  |  |  |  | 1 | 0 | 4 | 0 |  |  |  |  |  |  | 1 | 0 | 5 | 0 |  |  |  |  |  |  | 1 | 0 | 6 | 0 |  |  |  |  |  |  | 1 | 0 | 7 | 0 |  |  |  |  |  |  | 1 | 0 | 8 | 0 |  |  |  |  |  |  | 1 | 0 | 9 | 0 |  |  |  |  |  |  | 1 | 1 | 0 | 0 |  |  |  |  |  |  | 1 | 1 | 1 | 0 |  |  |  |  |  |  | 1 | 1 | 2 | 0 |  |  |  |  |  |  | 1 | 1 | 3 | 0 |  |  |  |  |  |  | 1 | 1 | 4 | 0 |  |  |  |  |  |  | 1 | 1 | 5 | 0 |  |  |  |  |  |  | 1 | 1 | 6 | 0 |  |  |  |  |  |  | 1 | 1 | 7 | 0 |  |  |  |  |  |  | 1 | 1 | 8 | 0 |  |  |  |  |  |  | 1 | 1 | 9 | 0 |  |  |  |  |  |  | 1 | 2 | 0 | 0 |  |  |  |  |  |  | 1 | 2 | 1 | 0 |  |  |  |  |  |  | 1 | 2 | 2 | 0 |  |  |  |  |  |  | 1 | 2 | 3 | 0 |  |  |  |  |  |  | 1 | 2 | 4 | 0 |  |  |  |  |  |  | 1 | 2 | 5 | 0 |  |  |  |  |  |  | 1 | 2 | 6 | 0 |  |  |  |  |  |  | 1 | 2 | 7 | 0 |  |  |  |  |  |  | 1 | 2 | 8 | 0 |  |  |  |  |  |  | 1 | 2 | 9 | 0 |  |  |  |  |  |  | 1 | 3 | 0 | 0 |  |  |  |  |  |  | 1 | 3 | 1 | 0 |  |  |  |  |  |  | 1 | 3 | 2 | 0 |  |  |  |  |  |  | 1 | 3 | 3 | 0 |  |  |  |  |  |  | 1 | 3 | 4 | 0 |  |  |  |  |  |  | 1 | 3 | 5 | 0 |  |  |  |  |  |  | 1 | 3 | 6 | 0 |  |  |  |  |  |  | 1 | 3 | 7 | 0 |  |  |  |  |  |  | 1 | 3 | 8 | 0 |  |  |  |  |  |  | 1 | 3 | 9 | 0 |  |  |  |  |  |  | 1 | 4 | 0 | 0 |  |  |  |  |  |  | 1 | 4 | 1 | 0 |  |  |  |  |  |  | 1 | 4 | 2 | 0 |  |  |  |  |  |  | 1 | 4 | 3 | 0 |  |  |  |  |  |  | 1 | 4 | 4 | 0 |  |  |  |  |  |  | 1 | 4 | 5 | 0 |  |  |  |  |  |  | 1 | 4 | 6 | 0 |  |  |  |  |  |  | 1 | 4 | 7 | 0 |  |  |  |  |  |  | 1 | 4 | 8 | 0 |  |  |  |  |  |  | 1 | 4 | 9 | 0 |  |  |  |  |  |  | 1 | 5 | 0 | 0 |  |  |  |  |  |  | 1 | 5 | 1 | 0 |  |  |  |  |  |  | 1 | 5 | 2 | 0 |  |  |  |  |  |  | 1 | 5 | 3 | 0 |  |  |  |  |  |  | 1 | 5 | 4 | 0 |  |  |  |  |  |  | 1 | 5 | 5 | 0 |  |  |  |  |  |  | 1 | 5 | 6 | 0 |  |  |  |  |  |  | 1 | 5 | 7 | 0 |  |  |  |  |  |  | 1 | 5 | 8 | 0 |  |  |  |  |  |  | 1 | 5 | 9 | 0 |  |  |  |  |  |  | 1 | 6 | 0 | 0 |  |  |  |  |  |  | 1 | 6 | 1 | 0 |  |  |  |  |  |  | 1 | 6 | 2 | 0 |  |  |  |  |  |  | 1 | 6 | 3 | 0 |  |  |  |  |  |  | 1 | 6 | 4 | 0 |  |  |  |  |  |  | 1 | 6 | 5 | 0 |  |  |  |  |  |  | 1 | 6 | 6 | 0 |  |  |  |  |  |  | 1 | 6 | 7 | 0 |  |  |  |  |  |  | 1 | 6 | 8 | 0 |  |  |  |  |  |  | 1 | 6 | 9 | 0 |  |  |  |  |  |  | 1 | 7 | 0 | 0 |  |  |  |  |  |  | 1 | 7 | 1 | 0 |  |  |  |  |  |  | 1 | 7 | 2 | 0 |  |  |  |  |  |  | 1 | 7 | 3 | 0 |  |  |  |  |  |  | 1 | 7 | 4 | 0 |  |  |  |  |  |  | 1 | 7 | 5 | 0 |  |  |  |  |  |  | 1 | 7 | 6 | 0 |  |  |  |  |  |  | 1 | 7 | 7 | 0 |  |  |  |  |  |  | 1 | 7 | 8 | 0 |  |  |  |  |  |  | 1 | 7 | 9 | 0 |  |  |  |  |  |  | 1 | 8 | 0 | 0 |  |  |  |  |  |  | 1 | 8 | 1 | 0 |  |  |  |  |  |  | 1 | 8 | 2 | 0 |  |  |  |  |  |  | 1 | 8 | 3 | 0 |  |  |  |  |  |  | 1 | 8 | 4 | 0 |  |  |  |  |  |  | 1 | 8 | 5 | 0 |  |  |  |  |  |  | 1 | 8 | 6 | 0 |  |  |  |  |  |  | 1 | 8 | 7 | 0 |  |  |  |  |  |  | 1 | 8 | 8 | 0 |  |  |  |  |  |  | 1 | 8 | 9 | 0 |  |  |  |  |  |  | 1 | 9 | 0 | 0 |  |  |  |  |  |  | 1 | 9 | 1 | 0 |  |  |  |  |  |  | 1 | 9 | 2 | 0 |  |  |  |  |  |  | 1 | 9 | 3 | 0 |  |  |  |  |  |  | 1 | 9 | 4 | 0 |  |  |  |  |  |  | 1 | 9 | 5 | 0 |  |  |  |  |  |  | 1 | 9 | 6 | 0 |  |  |  |  |  |  | 1 | 9 | 7 | 0 |  |  |  |  |  |  | 1 | 9 | 8 | 0 |  |  |  |  |  |  | 1 | 9 | 9 | 0 |  |  |  |  |  |  | 2 | 0 | 0 | 0 |  |  |  |  |  |  | 2 | 0 | 1 | 0 |  |  |  |  |  |  | 2 | 0 | 2 | 0 |  |  |  |  |  |  | 2 | 0 | 3 | 0 |  |  |  |  |  |  | 2 | 0 | 4 | 0 |  |  |  |  |  |  | 2 | 0 | 5 | 0 |  |  |  |  |  |  | 2 | 0 | 6 | 0 |  |  |  |  |  |  | 2 | 0 | 7 | 0 |  |  |  |  |  |  | 2 | 0 | 8 | 0 |  |  |  |  |  |  | 2 | 0 | 9 | 0 |  |  |  |  |  |  | 2 | 1 | 0 | 0 |  |  |  |  |  |  | 2 | 1 | 1 | 0 |  |  |  |  |  |  | 2 | 1 | 2 | 0 |  |  |  |  |  |  | 2 | 1 | 3 | 0 |  |  |  |  |  |  | 2 | 1 | 4 | 0 |  |  |  |  |  |  | 2 | 1 | 5 | 0 |  |  |  |  |  |  | 2 | 1 | 6 | 0 |  |  |  |  |  |  | 2 | 1 | 7 | 0 |  |  |  |  |  |  | 2 | 1 | 8 | 0 |  |  |  |  |  |  | 2 | 1 | 9 | 0 |  |  |  |  |  |  | 2 | 2 | 0 | 0 |  |  |  |  |  |  | 2 | 2 | 1 | 0 |  |  |  |  |  |  | 2 | 2 | 2 | 0 |  |  |  |  |  |  | 2 | 2 | 3 | 0 |  |  |  |  |  |  | 2 | 2 | 4 | 0 |  |  |  |  |  |  | 2 | 2 | 5 | 0 |  |  |  |  |  |  | 2 | 2 | 6 | 0 |  |  |  |  |  |  | 2 | 2 | 7 | 0 |  |  |  |  |  |  | 2 | 2 | 8 | 0 |  |  |  |  |  |  | 2 | 2 | 9 | 0 |  |  |  |  |  |  | 2 | 3 | 0 | 0 |  |  |  |  |  |  | 2 | 3 | 1 | 0 |  |  |  |  |  |  | 2 | 3 | 2 | 0 |  |  |  |  |  |  | 2 | 3 | 3 | 0 |  |  |  |  |  |  | 2 | 3 | 4 | 0 |  |  |  |  |  |  | 2 | 3 | 5 | 0 |  |  |  |  |  |  | 2 | 3 | 6 | 0 |  |  |  |  |  |  | 2 | 3 | 7 | 0 |  |  |  |  |  |  | 2 | 3 | 8 | 0 |  |  |  |  |  |  | 2 | 3 | 9 | 0 |  |  |  |  |  |  | 2 | 4 | 0 | 0 |  |  |  |  |  |  | 2 | 4 | 1 | 0 |  |  |  |  |  |  | 2 | 4 | 2 | 0 |  |  |  |  |  |  | 2 | 4 | 3 | 0 |  |  |  |  |  |  | 2 | 4 | 4 | 0 |  |  |  |  |  |  | 2 | 4 | 5 | 0 |  |  |  |  |  |  | 2 | 4 | 6 | 0 |  |  |  |  |  |  | 2 | 4 | 7 | 0 |  |  |  |  |  |  | 2 | 4 | 8 | 0 |  |  |  |  |  |  | 2 | 4 | 9 | 0 |  |  |  |  |  |  | 2 | 5 | 0 | 0 |  |  |  |  |  |  | 2 | 5 | 1 | 0 |  |  |
|  | ATENSPM6\_Athal | - | - | - | - | - | - | M | A | G | N | Y | N | Y | G | G | T | G | G | F | Y | R | - | D | W | M | Y | K | R | F | D | E | V | T | G | N | L | S | A | E | Y | V | A | G | V | E | Q | F | M | T | F | A | N | S | Q | P | I | V | - | Q | S | S | R | G | K | F | H | C | P | C | S | V | C | K | N | E | K | H | I | I | S | G | - | - | - | - | R | R | V | S | S | H | L | F | S | H | G | F | M | P | D | Y | Y | V | - | - | - | W | Y | K | H | - | G | E | - | - | E | M | N | M | D | I | G | T | S | - | - | Y | T | N | R | T | Y | F | S | E | N | H | E | - | - | - | - | - | - | - | - | - | - | - | - | - | - | - | - | - | - | - | - | - | - | - | - | - | - | - | - | - | - | - | - | - | - | - | - | - | - | - | - | - | E | V | G | N | I | V | E | D | P | Y | V | D | M | V | N | D | A | F | N | F | N | V | G | Y | - | - | - | - | - | - | D | D | N | Y | R | - | - | - | - | - | - | - | - | H | D | D | S | - | - | - | - | - | - | - | Y | Q | N | V | E | E | P | V | R | - | - | - | - | - | - | - | - | - | - | - | N | H | S | N | K | F | Y | D | L | L | E | G | A | N | N | P | L | Y | - | - | - | D | G | C | R | E | G | Q | S | Q | L | S | L | A | S | R | L | M | H | N | K | A | E | Y | N | M | S | - | - | - | - | - | E | K | L | V | D | S | V | C | E | M | F | - | T | D | F | L | P | E | G | N | Q | A | T | T | S | H | Y | Q | T | E | K | L | M | R | N | L | G | L | P | Y | H | T | I | D | V | C | Q | N | N | C | M | L | F | W | K | E | - | D | E | K | E | D | Q | C | R | F | C | G | A | K | R | W | K | P | K | D | - | - | - | - | - | - | - | - | - | - | - | - | - | - | - | - | - | - | - | - | - | - | - | - | - | - | - | - | - | - | - | - | - | - | - | - | - | - | - | - | - | D | R | R | R | T | K | V | P | Y | S | R | M | W | Y | L | P | I | G | D | R | L | K | R | M | Y | Q | S | H | K | T | A | A | A | M | R | W | H | A | E | H | - | - | - | - | - | - | - | - | - | - | Q | S | - | - | - | K | E | G | E | - | - | - | - | - | M | N | H | P | S | D | A | A | E | W | R | Y | F | Q | G | L | H | P | Q | - | F | A | E | E | P | R | N | V | Y | L | G | L | C | T | D | G | F | N | P | F | G | - | M | S | R | N | H | S | L | W | P | V | I | L | T | P | Y | N | L | - | P | P | G | M | C | M | N | T | E | Y | L | F | L | T | I | L | N | S | G | P | N | H | P | R | A | S | L | - | D | V | F | L | Q | P | L | I | E | E | L | K | E | L | W | S | - | T | G | V | D | A | Y | D | V | S | L | S | Q | N | F | N | L | K | A | V | L | L | W | T | I | S | D | F | P | A | Y | S | M | L | S | G | W | T | T | H | G | K | L | S | - | - | C | P | V | C | M | E | S | T | K | S | F | Y | L | P | N | G | R | K | T | C | W | F | D | C | H | R | R | F | L | P | H | G | H | P | S | R | - | - | R | N | R | K | D | F | L | K | G | R | D | A | S | S | E | Y | P | P | E | S | L | T | - | G | E | Q | V | Y | Y | E | R | L | A | S | V | N | P | P | K | T | K | D | - | - | - | - | - | - | - | - | - | - | - | - | - | - | - | - | - | - | - | - | - | - | - | - | - | - | - | - | - | - | - | - | - | - | - | - | - | V | G | G | N | G | H | E | K | K | M | R | G | Y | - | G | K | E | H | N | W | H | K | E | S | I | L | W | E | - | L | S | Y | W | K | - | - | D | L | N | L | R | H | N | I | D | V | M | H | T | E | K | N | F | L | D | N | I | M | N | T | L | M | R | V | K | G | K | S | K | D | N | I | M | S | R | L | D | I | E | K | F | C | S | R | P | G | L | H | I | - | - | - | - | - | - | - | - | - | - | - | - | - | - | D | S | S | G | K | A | P | F | P | A | Y | T | L | T | E | E | A | K | Q | S | L | L | Q | C | V | K | Y | D | I | R | F | P | D | G | Y | S | S | D | L | A | S | C | V | D | L | D | N | G | K | F | S | G | - | M | K | S | H | D | C | H | V | F | M | E | R | L | L | P | F | I | F | A | E | L | - | - | - | - | - | - | - | L | D | R | - | N | V | H | L | A | L | S | - | - | - | - | - | - | - | - | - | - | - | - | - | - | - | - | - | - | - | - | - | - | - | G | I | G | A | F | F | R | D | L | C | S | R | T | L | Q | T | S | R | V | Q | I | L | K | Q | N | I | V | L | I | I | C | N | L | E | K | I | F | P | P | S | - | - | - | - | F | F | D | V | M | E | H | L | P | I | H | L | P | Y | E | A | E | L | G | G | P | V | Q | Y | R | W | M | Y | P | F | E | - | - | - | - | - | - | - | - | - | - | - | - | - | - | - | - | - | - | - | - | - | - | - | - | - | - | - | - | - | R | F | - | - | - | - | - | - | - | - | - | - | - | - | - | - | - | - | - | - | - | - | - | - | - | - | - | - | - | - | - | - | - | - | - | - | - | - | - | - | - | - | - | - | - | - | - | - | - | - | - | - | - | - | - | - | - | - | - | - | - | - | - | - | - | - | - | - | - | - | - | - | - | - | - | - | F | K | K | L | K | G | K | A | K | N | K | R | Y | A | A | G | S | I | V | E | S | Y | I | N | D | E | I | A | Y | - | - | - | - | F | S | E | H | Y | F | A | D | H | I | Q | - | - | - | T | K | S | R | L | T | R | F | N | E | G | E | V | P | V | Y | - | - | H | V | P | G | V | P | N | I | F | M | H | V | G | R | P | S | G | E | M | H | V | D | W | L | S | - | - | - | - | E | K | D | Y | Q | S | A | H | A | Y | V | L | R | N | C | D | Y | F | K | P | - | F | E | S | M | - | - | - | - | - | - | - | - | - | - | - | - | - | - | - | - | - | - | - | - | - | - | - | - | - | - | - | - | - | - | - | - | - | - | - | - | - | - | - | - | - | - | - | - | - | - | - | - | - | - | - | - | - | - | - | - | - | - | - | - | - | - | - | - | - | - | - | - | - | - | - | - | - | - | - | - | - | - | - | - | - | - | - | - | - | - | - | - | - | - | - | - | - | - | F | E | D | Y | L | S | A | K | Y | P | C | L | P | E | - | - | K | E | L | Y | A | R | R | A | E | E | Y | H | L | W | V | K | E | Y | V | T | Y | - | - | - | - | - | - | - | - | - | - | - | - | - | - | - | - | - | - | - | - | - | - | - | - | - | - | - | - | - | - | - | W | N | T | T | S | P | F | P | T | W | V | Q | E | I | V | Q | G | P | L | N | K | V | K | T | W | P | M | Y | F | T | R | G | Y | L | F | H | T | Q | N | H | G | A | G | - | - | - | - | R | K | T | C | N | Y | G | V | C | V | K | G | E | N | Y | A | D | S | S | D | E | - | - | - | - | - | - | - | - | A | D | F | Y | G | T | L | T | D | I | I | E | L | E | Y | - | - | - | - | - | E | G | I | V | N | L | R | I | T | L | F | K | - | C | K | W | Y | D | - | - | - | - | - | - | - | - | - | - | - | - | P | K | I | G | R | G | T | R | R | S | H | S | - | G | V | V | D | - | - | - | - | - | - | - | - | - | - | - | I | L | S | - | - | - | - | - | T | R | K | Y | - | - | N | K | Y | E | P | F | I | L | G | S | Q | A | D | Q | V | C | Y | I | P | Y | P | - | - | Y | T | K | K | P | K | N | I | - | - | - | - | - | - | - | - | - | - | - | - | W | L | N | V | L | K | V | N | P | R | G | N | I | S | G | E | Y | E | N | N | D | P | T | L | L | Q | T | E | N | D | D | D | V | L | L | T | T | I | E | D | L | V | L | E | - | - | T | P | V | A | N | L | N | P | I | I | L | D | Y | D | V | G | D | A | E | P | E | D | E | F | R | C | N | L | S | S | S | D | E | D | E | V | E | D | E | D | V | - | - | - | - | - | - | - | - | - | - | - | - | - | - | - | - | - | - | - | - | - | - | - | - | - | - | - | - | - | - | - | - | - | - | - | - | - | - | - | - | - | - | - | - | - | - | - | - | - | - | - | - | - | - | - | - | - | - | - | - | - | - | - | - | - | - | - | - | - | - | - | - | - | - | - | - | - | - | - | - | - | - | - | - | - | - | - | - | - | - | - | - | - | - | - | - | - | - | - | - | - | - | - | - | - | - | - | - | - | - | - | - | - | - | - | - | - | - | - | - | - | - | - | - | - | - | - | - | - | - | - | - | - | - | - | - | - | - | - | - | - | - | - | - | - | - | - | - | - | - | - | - | - | - | - | - | - | - | - | - | - | - | - | - | - | - | - | - | - | - | - | - | - | - | - | - | - | - | - | - | - | - | - | - | - | - | - | - | - | - | - | - | - | - | - | - | - | - | - | - | - | - | - | - | - | - | - | - | - | - | - | - | - | - | - | - | - | - | - | - | - | - | - | - | - | - | - | - | - | - | - | - | - | - | - | - | - | - | - | - | - | - | - | - | - | - | - | - | - | - | - | - | - | - | - | - | - | - | - | - | - | - | - | - | - | - | - | - | - | - | - | - | - | - | - | - | - | - | - | - | - | - | - | - | - | - | - | - | - | - | - | - | - | - | - | - | - | - | - | - | - | - | - | - | - | - | - | - | - | - | - | - | - | - | - | - | - | - | - | - | - | - | - | - | - | - | - | - | - | - | - | - | - | - | - | - | - | - | - | - | - | - | - | - | - | - | - | - | - | - | - | - | - | - | - | - | - | - | - | - | - | - | - | - | - | - | - | - | - | - | - | - | - | - | - | - | - | - | - | - | - | - | - | - | - | - | - | - | - | - | - | - | - | - | - | - | - | - | - | - | - | - | - | - | - | - | - | - | - | - | - | - | - | - | - | - | - | - | - | - | - | - | - | - | - | - | - | - | - | - | - | - | - | - | - | - | - | - | - | - | - | - | - | - | - | - | - | - | - | - | - | - | - | - | - | - | - | - | - | - | - | - | - | - | - | - | - | - | - | - | - | - | - | - | - | - | - | - | - | - | - | - | - | - | - | - | - | - | - | - | - | - | - | - | - | - | - | - | - | - | - | - | - | - | - | - | - | - | - | - | - | - | - | - | - | - | - | - | - | - | - | - | - | - | - | - | - | - | - | - | - | - | - | - | - | - | - | - | - | - | - | - | - | - | - | - | - | - | - | - | - | - | - | - | - | - | - | - | - | - | - | - | - | - | - | - | - | - | - | - | - | - | - | - | - | - | - | - | - | - | - | - | - | - | - | - | - | - | - | - | - | - | - | - | - | - | - | - | - | - | - | - | - | - | - | - | - | - | - | - | - | - | - | - | - | - | - | - | - | - | - | - | - | - | - | - | - | - | - | - | - | - | - | - | - | - | - | - | - | - | - | - | - | - | - | - | - | - | - | - | - | - | - | - | - | - | - | - | - | - | - | - | - | - | - | - | - | - | - | - | - | - | - | - | - | - | - | - | - | - | - | - | - | - | - | - | - | - | - | - | - | - | - | - | - | - | - | - | - | - | - | - | - | - | - | - | - | - | - | - | - | - | - | - | - | - | - | - | - | - | - | - | - | - | - | - | - | - | - | - | - | - | - | - | - | - | - | - | - | - | - | - | - | - | - | - | - | - | - | - | - | - | - | - | - | - | - | - | - | - | - | - | - | - | - | - | - | - | - | - | - | - | - | - | - | - | - | - | - | - | - | - | - | - | - | - |
|  | Aron | - | - | - | - | - | - | M | A | G | N | Y | N | Y | G | G | S | G | G | F | Y | R | - | D | W | M | Y | K | R | F | D | E | V | T | G | N | L | S | A | E | Y | V | A | G | V | E | E | F | M | T | F | A | N | S | Q | P | I | V | - | Q | S | C | R | G | K | F | H | C | P | C | S | V | C | K | N | E | K | H | I | I | S | G | - | - | - | - | R | R | V | S | S | H | L | F | S | Q | G | F | M | P | D | Y | Y | V | - | - | - | W | Y | K | H | - | G | E | - | - | E | L | N | M | D | I | G | T | S | - | - | Y | T | D | R | T | Y | F | S | E | N | H | E | - | - | - | - | - | - | - | - | - | - | - | - | - | - | - | - | - | - | - | - | - | - | - | - | - | - | - | - | - | - | - | - | - | - | - | - | - | - | - | - | - | E | V | G | N | V | V | E | D | P | Y | V | D | M | V | N | D | A | F | N | F | N | V | G | Y | D | D | N | V | G | H | D | D | N | Y | H | - | - | - | - | - | - | - | - | H | D | G | S | - | - | - | - | - | - | - | Y | Q | N | V | E | E | P | V | R | - | - | - | - | - | - | - | - | - | - | - | N | H | S | N | K | F | Y | D | L | L | E | G | A | N | N | P | L | Y | - | - | - | D | G | C | R | E | G | Q | S | Q | L | S | L | A | S | R | L | M | H | N | K | A | E | Y | N | M | S | - | - | - | - | - | E | K | L | V | D | S | V | C | E | M | F | - | T | D | F | L | P | E | G | N | Q | A | T | T | S | H | Y | Q | T | E | K | L | M | R | N | L | G | L | P | Y | H | T | I | D | V | C | K | N | N | C | M | L | F | W | K | E | - | D | E | K | E | D | Q | C | R | F | C | G | A | Q | R | W | K | P | K | D | - | - | - | - | - | - | - | - | - | - | - | - | - | - | - | - | - | - | - | - | - | - | - | - | - | - | - | - | - | - | - | - | - | - | - | - | - | - | - | - | - | D | R | R | R | T | K | V | P | Y | S | R | M | W | Y | L | P | I | A | D | R | L | K | R | M | Y | Q | S | H | K | T | A | A | A | M | R | W | H | A | E | H | - | - | - | - | - | - | - | - | - | - | Q | S | - | - | - | K | E | G | E | - | - | - | - | - | M | N | H | P | S | D | A | A | E | W | R | Y | F | Q | E | L | H | P | R | - | F | A | E | E | P | R | N | V | Y | L | G | L | C | T | D | G | F | N | P | F | G | - | M | S | R | N | H | S | L | W | P | V | I | L | T | P | Y | N | L | - | P | P | G | M | C | M | N | T | E | Y | L | F | L | T | I | L | N | S | G | P | N | H | P | R | A | S | L | - | D | V | F | L | Q | P | L | I | E | E | L | K | E | L | W | C | - | T | G | V | D | A | Y | D | V | S | L | S | Q | N | F | N | L | K | A | V | L | L | W | T | I | S | D | F | P | A | Y | S | M | L | S | G | W | T | T | H | G | K | L | S | - | - | C | P | V | C | M | E | S | T | K | S | F | Y | L | P | N | G | R | K | T | C | W | F | D | C | H | R | R | F | L | P | H | G | H | P | S | R | - | - | R | N | K | K | D | F | L | K | G | R | D | A | S | S | E | Y | P | P | E | S | L | T | - | G | E | Q | V | Y | Y | E | R | L | A | S | V | N | P | P | K | T | K | D | - | - | - | - | - | - | - | - | - | - | - | - | - | - | - | - | - | - | - | - | - | - | - | - | - | - | - | - | - | - | - | - | - | - | - | - | - | V | G | G | N | G | H | E | K | K | M | R | G | Y | - | G | K | E | H | N | W | H | K | E | S | I | L | W | E | - | L | S | Y | W | K | - | - | D | L | N | L | R | H | N | I | D | V | M | H | T | E | K | N | F | L | D | N | I | M | N | T | L | L | G | V | K | G | K | S | K | D | N | I | M | S | R | L | D | I | E | K | Y | C | S | R | P | G | L | H | I | - | - | - | - | - | - | - | - | - | - | - | - | - | - | D | S | T | G | K | A | P | F | P | P | Y | T | L | T | E | E | A | K | Q | S | L | F | Q | C | V | K | H | D | V | R | F | P | D | G | Y | S | S | D | L | A | S | C | V | D | L | E | N | G | K | F | S | G | - | M | K | S | H | D | C | H | V | F | M | E | R | L | L | P | F | I | F | A | E | L | - | - | - | - | - | - | - | L | D | R | - | N | V | H | L | A | L | S | G | T | I | L | T | I | F | Y | G | E | I | F | L | C | N | Y | L | T | S | T | Y | I | L | G | I | G | A | F | F | R | D | L | C | S | R | T | L | Q | T | S | R | V | Q | I | L | K | Q | N | I | V | L | I | I | C | N | L | E | K | I | F | P | P | S | - | - | - | - | F | F | D | V | M | E | H | L | P | I | H | L | P | Y | E | A | E | L | G | G | P | V | Q | Y | R | W | M | Y | P | F | E | - | - | - | - | - | - | - | - | - | - | - | - | - | - | - | - | - | - | - | - | - | - | - | - | - | - | - | - | - | R | F | - | - | - | - | - | - | - | - | - | - | - | - | - | - | - | - | - | - | - | - | - | - | - | - | - | - | - | - | - | - | - | - | - | - | - | - | - | - | - | - | - | - | - | - | - | - | - | - | - | - | - | - | - | - | - | - | - | - | - | - | - | - | - | - | - | - | - | - | - | - | - | - | - | - | F | K | K | L | K | G | K | A | K | N | K | R | Y | A | A | G | S | I | V | E | S | Y | I | N | D | E | I | A | Y | - | - | - | - | F | S | E | H | Y | F | A | D | H | I | Q | - | - | - | T | K | S | - | - | - | R | F | D | E | G | E | V | P | V | Y | - | - | H | V | P | G | V | P | N | I | F | M | Q | V | G | R | P | S | G | A | M | H | V | E | W | L | S | - | - | - | - | E | K | D | Y | Q | N | A | H | A | Y | V | L | R | N | - | - | - | - | - | - | - | - | - | - | - | - | - | - | - | - | - | - | - | - | - | - | - | - | - | - | - | - | - | - | - | - | - | - | - | - | - | - | - | - | - | - | - | - | - | - | - | - | - | - | - | - | - | - | - | - | - | - | - | - | - | - | - | - | - | - | - | - | - | - | - | - | - | - | - | - | - | - | - | - | - | - | - | - | - | - | - | - | - | - | - | - | - | - | - | - | - | - | - | - | - | - | - | - | - | - | - | - | - | - | - | - | - | - | - | - | - | - | - | - | - | - | - | - | - | - | - | - | - | - | - | - | - | - | - | - | - | - | - | - | - | - | - | - | - | - | - | - | - | - | - | - | - | - | - | - | - | - | - | - | - | - | - | - | - | - | - | - | - | - | - | - | - | - | - | - | - | - | - | - | - | - | - | - | - | - | I | V | Q | G | P | L | N | K | V | K | T | W | P | M | Y | F | T | R | G | Y | L | F | H | T | Q | T | H | G | A | G | - | - | - | - | R | K | T | C | N | Y | G | V | C | V | K | G | E | N | Y | A | D | S | S | D | E | - | - | - | - | - | - | - | - | A | D | F | Y | G | T | L | T | D | V | I | E | L | E | Y | - | - | - | - | - | E | G | I | V | N | L | R | I | T | L | F | K | - | C | K | W | Y | D | - | - | - | - | - | - | - | - | - | - | - | - | P | K | I | G | R | G | T | R | R | N | H | G | - | G | V | V | D | - | - | - | - | - | - | - | - | - | - | - | V | L | S | - | - | - | - | - | T | R | K | Y | - | - | N | K | Y | E | P | F | I | L | - | - | - | - | D | Q | V | C | Y | I | P | Y | P | - | - | Y | T | K | K | P | K | N | I | - | - | - | - | - | - | - | - | - | - | - | - | W | L | S | V | L | K | V | N | P | R | G | N | I | S | G | Q | Y | E | N | T | D | P | T | L | L | Q | T | E | D | D | E | A | V | L | Q | T | T | I | E | D | L | V | I | D | - | - | Y | P | V | A | D | V | T | P | I | I | L | D | Y | D | I | G | D | A | E | P | E | D | E | F | R | C | N | L | S | S | S | D | E | D | E | I | E | D | E | D | E | - | - | - | - | - | - | - | - | - | - | - | - | - | - | - | - | - | - | - | - | - | - | - | - | - | - | - | - | - | - | - | - | - | - | - | - | - | - | - | - | - | - | - | - | - | - | - | - | - | - | - | - | - | - | - | - | - | - | - | - | - | - | - | - | - | - | - | - | - | - | - | - | - | - | - | - | - | - | - | - | - | - | - | - | - | - | - | - | - | - | - | - | - | - | - | - | - | - | - | - | - | - | - | - | - | - | - | - | - | - | - | - | - | - | - | - | - | - | - | - | - | - | - | - | - | - | - | - | - | - | - | - | - | - | - | - | - | - | - | - | - | - | - | - | - | - | - | - | - | - | - | - | - | - | - | - | - | - | - | - | - | - | - | - | - | - | - | - | - | - | - | - | - | - | - | - | - | - | - | - | - | - | - | - | - | - | - | - | - | - | - | - | - | - | - | - | - | - | - | - | - | - | - | - | - | - | - | - | - | - | - | - | - | - | - | - | - | - | - | - | - | - | - | - | - | - | - | - | - | - | - | - | - | - | - | - | - | - | - | - | - | - | - | - | - | - | - | - | - | - | - | - | - | - | - | - | - | - | - | - | - | - | - | - | - | - | - | - | - | - | - | - | - | - | - | - | - | - | - | - | - | - | - | - | - | - | - | - | - | - | - | - | - | - | - | - | - | - | - | - | - | - | - | - | - | - | - | - | - | - | - | - | - | - | - | - | - | - | - | - | - | - | - | - | - | - | - | - | - | - | - | - | - | - | - | - | - | - | - | - | - | - | - | - | - | - | - | - | - | - | - | - | - | - | - | - | - | - | - | - | - | - | - | - | - | - | - | - | - | - | - | - | - | - | - | - | - | - | - | - | - | - | - | - | - | - | - | - | - | - | - | - | - | - | - | - | - | - | - | - | - | - | - | - | - | - | - | - | - | - | - | - | - | - | - | - | - | - | - | - | - | - | - | - | - | - | - | - | - | - | - | - | - | - | - | - | - | - | - | - | - | - | - | - | - | - | - | - | - | - | - | - | - | - | - | - | - | - | - | - | - | - | - | - | - | - | - | - | - | - | - | - | - | - | - | - | - | - | - | - | - | - | - | - | - | - | - | - | - | - | - | - | - | - | - | - | - | - | - | - | - | - | - | - | - | - | - | - | - | - | - | - | - | - | - | - | - | - | - | - | - | - | - | - | - | - | - | - | - | - | - | - | - | - | - | - | - | - | - | - | - | - | - | - | - | - | - | - | - | - | - | - | - | - | - | - | - | - | - | - | - | - | - | - | - | - | - | - | - | - | - | - | - | - | - | - | - | - | - | - | - | - | - | - | - | - | - | - | - | - | - | - | - | - | - | - | - | - | - | - | - | - | - | - | - | - | - | - | - | - | - | - | - | - | - | - | - | - | - | - | - | - | - | - | - | - | - | - | - | - | - | - | - | - | - | - | - | - | - | - | - | - | - | - | - | - | - | - | - | - | - | - | - | - | - | - | - | - | - | - | - | - | - | - | - | - | - | - | - | - | - | - | - | - | - | - | - | - | - | - | - | - | - | - | - | - | - | - | - | - | - | - | - | - | - | - | - | - | - | - | - | - | - | - | - | - | - | - | - | - | - | - | - | - | - | - | - | - | - | - | - | - | - | - | - | - | - | - | - | - | - | - | - | - | - | - | - | - | - | - | - | - | - | - | - | - | - | - | - | - | - | - | - | - | - | - | - | - | - | - | - | - | - | - | - | - | - | - | - | - | - | - | - | - | - | - | - | - | - | - | - | - | - | - | - | - |
|  | Dario | - | - | - | - | - | - | M | A | G | N | Y | N | Y | P | S | S | G | G | F | N | R | - | D | W | M | Y | K | R | F | D | E | M | T | G | N | L | S | A | E | Y | V | A | G | V | E | E | F | L | T | F | A | N | S | Q | P | I | V | - | Q | S | C | R | G | K | F | H | C | P | C | A | V | C | K | N | K | K | H | I | V | S | G | - | - | - | - | R | K | V | S | S | H | L | F | S | Q | G | F | M | P | D | Y | Y | V | - | - | - | W | Y | M | H | - | G | E | - | - | D | F | N | M | N | V | G | T | S | N | Y | V | I | D | S | T | Y | L | R | E | N | Y | E | S | V | G | N | V | V | E | D | - | - | - | - | - | - | - | - | - | - | - | - | - | - | - | - | - | - | - | - | - | - | - | - | - | - | P | Y | V | - | - | - | - | D | V | G | N | V | V | E | D | P | Y | V | D | M | V | N | D | A | F | R | Y | N | V | G | F | - | - | - | - | - | - | D | D | N | Y | H | - | - | - | - | - | - | - | - | Q | D | G | T | - | - | - | - | - | - | - | N | Q | N | V | E | E | P | V | H | - | - | - | - | - | - | - | - | - | - | - | N | H | S | K | K | F | Y | D | L | L | E | G | A | Q | N | P | L | Y | - | - | - | D | G | C | R | Q | G | Q | S | Q | L | S | L | A | A | R | V | M | Q | N | K | A | D | H | N | M | S | - | - | - | - | - | E | R | C | V | D | S | V | Y | Q | M | L | - | T | D | F | L | P | E | G | N | Q | A | T | D | S | H | Y | K | T | E | K | L | M | C | N | L | G | L | P | Y | Y | T | I | D | V | C | I | N | N | C | M | I | F | W | K | E | - | D | E | K | E | D | K | C | R | F | C | S | A | Q | R | W | K | P | M | D | D | - | - | - | - | - | - | - | - | - | - | - | - | - | - | - | - | - | - | - | - | - | - | - | - | - | - | - | - | - | - | - | - | - | - | - | - | - | - | - | - | Y | R | R | R | T | K | V | P | Y | S | R | M | R | Y | L | P | I | G | D | R | L | K | R | M | Y | Q | S | H | K | T | A | A | A | M | R | W | H | A | E | H | - | - | - | - | - | - | - | - | - | - | Q | S | - | - | - | K | E | E | E | - | - | - | - | - | M | N | H | P | S | D | A | A | E | W | R | Y | F | Q | E | L | H | P | M | - | F | A | E | E | P | R | N | V | Y | L | G | L | C | T | D | G | F | N | P | F | G | - | M | S | R | N | H | S | L | W | P | V | I | L | T | P | Y | N | L | - | P | P | G | M | C | M | N | I | E | Y | L | F | L | T | I | L | N | S | G | P | N | H | P | R | G | S | L | - | D | V | F | L | Q | P | L | I | E | E | L | K | E | L | W | S | - | T | G | I | D | A | Y | D | V | S | L | N | Q | N | F | N | L | K | A | V | L | L | W | T | I | S | D | F | P | A | Y | S | M | L | S | G | W | T | T | H | G | K | L | S | - | - | C | P | I | C | M | E | S | T | N | S | F | Y | L | P | N | G | R | K | T | C | W | F | D | C | H | R | R | F | L | S | H | G | H | P | L | R | - | - | K | N | K | K | D | F | R | K | G | K | D | A | S | T | E | Y | P | P | E | S | L | T | - | G | E | Q | I | Y | Y | E | R | L | S | G | V | N | P | P | R | T | K | D | - | - | - | - | - | - | - | - | - | - | - | - | - | - | - | - | - | - | - | - | - | - | - | - | - | - | - | - | - | - | - | - | - | - | - | - | - | V | G | G | N | G | H | E | K | K | M | P | G | Y | - | G | K | E | H | N | W | H | K | E | S | I | L | W | E | - | L | P | Y | W | K | - | - | D | L | N | L | R | H | C | I | D | V | M | H | T | E | K | N | F | L | D | N | I | M | N | T | L | M | S | V | K | G | K | S | K | D | N | I | M | A | R | M | D | I | E | R | F | C | S | R | P | D | L | H | I | - | - | - | - | - | - | - | - | - | - | - | - | - | - | D | S | K | R | K | A | P | F | P | A | Y | T | L | T | N | E | A | K | M | S | L | L | Q | C | V | K | H | A | I | K | F | P | D | G | Y | S | S | D | L | S | S | C | V | D | M | E | N | G | K | L | S | G | - | M | K | S | H | D | C | H | V | F | M | E | R | L | L | P | F | I | F | A | E | L | - | - | - | - | - | - | - | L | D | R | - | N | V | H | L | A | L | S | - | - | - | - | - | - | - | - | - | - | - | - | - | - | - | - | - | - | - | - | - | - | - | G | V | G | A | F | F | R | D | L | C | S | R | T | L | Q | K | S | H | V | Q | I | L | K | Q | N | I | V | L | I | I | C | N | L | E | K | I | F | P | P | S | - | - | - | - | F | F | D | V | M | E | H | L | P | I | H | L | P | Y | E | A | E | L | G | G | P | V | Q | Y | R | W | M | Y | P | F | E | - | - | - | - | - | - | - | - | - | - | - | - | - | - | - | - | - | - | - | - | - | - | - | - | - | - | - | - | - | R | F | - | - | - | - | - | - | - | - | - | - | - | - | - | - | - | - | - | - | - | - | - | - | - | - | - | - | - | - | - | - | - | - | - | - | - | - | - | - | - | - | - | - | - | - | - | - | - | - | - | - | - | - | - | - | - | - | - | - | - | - | - | - | - | - | - | - | - | - | - | - | - | - | - | - | F | K | K | L | K | G | K | A | K | N | K | R | Y | A | A | G | S | I | V | E | S | Y | I | N | D | E | I | S | Y | - | - | - | - | F | S | E | H | Y | F | A | D | D | I | Q | - | - | - | T | K | S | - | - | - | R | F | N | E | G | E | V | P | V | Y | - | - | H | V | P | G | V | P | T | I | F | S | S | V | G | R | P | S | G | E | I | R | E | V | W | L | S | - | - | - | - | E | E | D | Y | Q | C | A | H | G | Y | V | I | R | N | C | D | Y | F | Q | S | - | - | - | - | - | - | - | - | - | - | - | - | - | - | - | - | - | - | - | - | - | - | - | - | - | - | - | - | - | - | - | - | - | - | - | - | - | - | - | - | - | - | - | - | - | - | - | - | - | - | - | - | - | - | - | - | - | - | - | - | - | - | - | - | - | - | - | - | - | - | - | - | - | - | - | - | - | - | - | - | - | - | - | - | - | - | - | - | - | - | - | - | - | - | - | - | - | - | - | - | - | - | - | - | - | - | - | - | - | - | - | - | - | - | - | - | - | - | - | - | - | - | - | - | - | - | - | - | - | - | - | - | - | - | - | - | - | - | - | - | - | - | - | - | - | - | - | - | - | - | - | - | - | - | - | - | - | - | - | - | - | - | - | - | - | - | - | - | - | - | - | - | - | - | - | - | - | - | - | - | - | - | M | D | L | C | T | K | S | K | H | G | Q | C | I | L | Q | E | A | I | F | F | H | T | Q | S | H | G | A | G | - | - | - | - | R | K | T | C | N | Y | G | V | C | V | K | G | E | N | Y | T | D | A | S | D | A | - | - | - | - | - | - | - | - | A | D | F | Y | G | N | L | T | D | I | I | E | L | E | Y | - | - | - | - | - | E | G | V | V | S | L | K | I | T | L | F | K | - | C | S | W | Y | D | - | - | - | - | - | - | - | - | - | - | - | - | P | K | L | G | R | G | T | R | R | S | N | S | - | G | V | V | D | - | - | - | - | - | - | - | - | - | - | - | V | L | S | - | - | - | - | - | S | R | K | Y | - | - | N | K | Y | E | P | F | I | L | G | T | Y | G | I | Y | I | W | S | F | S | F | S | S | S | Y | T | N | I | - | - | - | - | - | - | - | - | - | - | - | - | - | - | - | - | - | - | - | - | - | - | - | - | - | - | - | - | - | - | - | - | - | - | - | - | - | - | - | - | - | - | - | - | - | - | - | - | - | - | - | - | - | - | - | - | - | - | - | - | - | - | - | - | - | - | - | - | - | - | - | - | - | - | - | - | - | - | - | - | - | - | - | - | - | - | - | - | - | - | - | - | - | - | - | - | - | - | - | - | - | - | - | - | - | - | - | - | - | - | - | - | - | - | - | - | - | - | - | - | - | - | - | - | - | - | - | - | - | - | - | - | - | - | - | - | - | - | - | - | - | - | - | - | - | - | - | - | - | - | - | - | - | - | - | - | - | - | - | - | - | - | - | - | - | - | - | - | - | - | - | - | - | - | - | - | - | - | - | - | - | - | - | - | - | - | - | - | - | - | - | - | - | - | - | - | - | - | - | - | - | - | - | - | - | - | - | - | - | - | - | - | - | - | - | - | - | - | - | - | - | - | - | - | - | - | - | - | - | - | - | - | - | - | - | - | - | - | - | - | - | - | - | - | - | - | - | - | - | - | - | - | - | - | - | - | - | - | - | - | - | - | - | - | - | - | - | - | - | - | - | - | - | - | - | - | - | - | - | - | - | - | - | - | - | - | - | - | - | - | - | - | - | - | - | - | - | - | - | - | - | - | - | - | - | - | - | - | - | - | - | - | - | - | - | - | - | - | - | - | - | - | - | - | - | - | - | - | - | - | - | - | - | - | - | - | - | - | - | - | - | - | - | - | - | - | - | - | - | - | - | - | - | - | - | - | - | - | - | - | - | - | - | - | - | - | - | - | - | - | - | - | - | - | - | - | - | - | - | - | - | - | - | - | - | - | - | - | - | - | - | - | - | - | - | - | - | - | - | - | - | - | - | - | - | - | - | - | - | - | - | - | - | - | - | - | - | - | - | - | - | - | - | - | - | - | - | - | - | - | - | - | - | - | - | - | - | - | - | - | - | - | - | - | - | - | - | - | - | - | - | - | - | - | - | - | - | - | - | - | - | - | - | - | - | - | - | - | - | - | - | - | - | - | - | - | - | - | - | - | - | - | - | - | - | - | - | - | - | - | - | - | - | - | - | - | - | - | - | - | - | - | - | - | - | - | - | - | - | - | - | - | - | - | - | - | - | - | - | - | - | - | - | - | - | - | - | - | - | - | - | - | - | - | - | - | - | - | - | - | - | - | - | - | - | - | - | - | - | - | - | - | - | - | - | - | - | - | - | - | - | - | - | - | - | - | - | - | - | - | - | - | - | - | - | - | - | - | - | - | - | - | - | - | - | - | - | - | - | - | - | - | - | - | - | - | - | - | - | - | - | - | - | - | - | - | - | - | - | - | - | - | - | - | - | - | - | - | - | - | - | - | - | - | - | - | - | - | - | - | - | - | - | - | - | - | - | - | - | - | - | - | - | - | - | - | - | - | - | - | - | - | - | - | - | - | - | - | - | - | - | - | - | - | - | - | - | - | - | - | - | - | - | - | - | - | - | - | - | - | - | - | - | - | - | - | - | - | - | - | - | - | - | - | - | - | - | - | - | - | - | - | - | - | - | - | - | - | - | - | - | - | - | - | - | - | - | - | - | - | - | - | - | - | - | - | - | - | - | - | - | - | - | - | - | - | - | - | - | - | - | - | - | - | - | - | - | - | - | - | - | - | - | - | - | - | - | - | - | - | - | - | - | - | - | - | - | - | - | - | - | - | - | - | - | - | - | - | - | - | - | - | - | - | - | - | - | - | - | - | - | - | - | - | - | - | - | - | - | - | - | - | - | - | - | - | - | - | - | - | - | - | - | - | - | - | - | - | - | - | - | - | - | - | - | - | - | - | - | - | - | - | - | - | - | - | - | - | - | - | - | - | - | - | - | - | - | - | - | - | - | - | - | - | - | - | - | - | - | - | - | - | - | - | - | - | - | - | - | - | - | - | - | - | - | - | - | - | - | - | - | - | - | - | - | - | - | - | - |
|  | Horace | - | - | - | - | - | - | - | - | M | S | W | G | G | E | H | E | I | Y | T | L | R | - | S | W | M | Y | N | H | R | D | Q | A | T | G | E | V | T | R | E | Y | Y | D | G | L | R | G | F | M | Y | Q | A | T | N | E | P | F | A | - | R | E | - | N | G | T | I | F | C | P | C | R | K | C | L | N | E | K | Y | L | E | - | F | - | - | - | - | N | V | V | K | K | H | L | Y | N | R | G | F | M | P | N | Y | Y | V | - | - | - | W | L | R | H | - | G | E | - | - | G | C | R | D | S | A | G | T | S | N | T | N | Y | G | V | E | Q | P | S | N | F | N | N | D | A | N | Y | G | Q | Q | V | P | - | - | - | - | - | - | - | - | - | - | - | - | - | - | - | - | - | - | - | - | - | - | - | - | - | D | Q | F | - | - | - | - | G | Y | G | H | N | Q | E | N | R | F | H | D | M | V | T | D | A | F | H | E | T | I | A | S | F | - | - | - | - | - | - | - | - | - | - | - | - | - | - | - | - | - | - | - | - | - | - | - | - | - | - | - | - | - | P | E | N | I | S | E | E | P | N | - | - | - | - | - | - | - | - | - | - | - | V | D | A | Q | H | F | Y | D | M | L | D | A | A | N | Q | P | I | Y | - | - | - | E | G | C | R | E | G | H | S | K | L | S | L | A | S | R | M | M | T | I | K | A | D | N | N | L | S | - | - | - | - | - | E | N | C | M | D | S | W | A | E | L | I | - | K | E | Y | L | P | P | D | N | I | S | A | E | S | Y | Y | E | I | Q | K | L | V | S | S | L | G | L | P | S | E | M | I | D | V | C | I | D | Y | C | M | I | F | W | G | D | - | D | V | N | L | Q | E | C | R | F | C | G | K | P | R | Y | Q | T | - | - | - | - | - | - | - | - | - | - | - | - | - | - | - | - | - | - | - | - | - | - | - | - | - | - | - | - | - | - | - | - | - | - | - | - | - | - | - | - | - | - | - | T | G | G | R | T | R | V | P | Y | K | R | M | W | Y | L | P | I | T | D | R | L | K | R | L | Y | Q | S | E | R | T | A | A | K | M | R | W | H | A | Q | H | - | - | - | - | - | - | - | - | - | - | T | T | - | - | - | A | D | G | E | - | - | - | - | - | I | T | H | P | S | D | A | K | A | W | K | H | F | Q | T | V | Y | P | D | - | F | A | N | E | C | R | N | V | Y | L | G | L | C | T | D | G | F | S | P | F | G | L | S | G | R | Q | Y | S | L | W | P | V | I | L | T | P | Y | N | L | - | P | P | D | M | C | M | Q | S | E | F | L | F | L | S | I | L | V | P | G | P | K | H | P | K | R | A | L | - | D | V | F | L | Q | P | L | I | H | E | L | K | K | L | W | Y | - | E | G | V | H | T | F | D | Y | S | S | K | Q | N | F | N | M | R | A | V | L | M | W | T | I | S | D | F | P | A | Y | G | M | L | S | G | W | T | T | H | G | R | L | S | - | - | C | P | Y | C | M | G | R | T | D | A | F | Q | L | K | K | G | R | K | S | C | W | F | D | C | H | R | R | F | L | P | L | H | H | P | Y | R | - | - | R | N | K | K | F | F | R | K | N | K | V | V | R | L | P | - | P | P | S | Y | V | S | - | G | T | D | L | F | - | E | Q | I | D | Y | Y | G | A | Q | E | T | C | K | - | - | - | - | - | - | - | - | - | - | - | - | R | G | G | - | - | - | - | - | - | - | - | - | - | - | - | - | - | - | - | - | - | - | - | - | - | - | N | W | H | T | T | A | N | M | P | D | E | Y | - | G | S | T | H | N | W | H | K | Q | S | I | F | W | Q | - | L | P | Y | W | K | - | - | D | L | L | L | R | H | N | L | D | V | M | H | I | E | K | N | F | F | D | N | F | M | N | T | L | L | N | V | Q | G | K | T | K | D | S | L | K | S | R | L | D | L | A | E | I | C | S | R | P | E | L | H | V | - | - | - | - | - | - | - | - | - | - | - | - | - | - | T | R | D | G | K | L | P | V | S | K | F | R | L | S | N | E | A | K | K | A | L | F | E | W | V | V | A | E | V | K | F | P | D | G | Y | V | S | K | F | S | R | C | V | E | - | Q | G | Q | K | F | S | G | - | M | K | S | H | D | C | H | V | F | M | Q | R | L | L | P | F | V | L | V | E | L | - | - | - | - | - | - | - | L | P | A | - | N | I | H | E | A | I | A | - | - | - | - | - | - | - | - | - | - | - | - | - | - | - | - | - | - | - | - | - | - | - | G | I | G | V | F | F | K | D | L | C | T | R | T | L | T | T | D | T | I | E | Q | L | D | K | N | I | P | V | L | L | C | N | L | E | K | L | F | P | P | A | - | - | - | - | F | F | D | V | M | E | H | L | P | I | H | L | P | H | E | A | A | L | G | G | P | V | Q | F | R | W | M | Y | P | F | E | - | - | - | - | - | - | - | - | - | - | - | - | - | - | - | - | - | - | - | - | - | - | - | - | - | - | - | - | - | R | F | - | - | - | - | - | - | - | - | - | - | - | - | - | - | - | - | - | - | - | - | - | - | - | - | - | - | - | - | - | - | - | - | - | - | - | - | - | - | - | - | - | - | - | - | - | - | - | - | - | - | - | - | - | - | - | - | - | - | - | - | - | - | - | - | - | - | - | - | - | - | - | - | - | - | M | K | S | L | K | G | K | A | K | N | L | A | R | V | E | G | S | I | V | A | G | S | L | T | E | E | T | S | H | - | - | - | - | F | T | S | Y | Y | F | S | P | S | V | R | - | - | T | K | K | T | R | P | R | R | Y | D | D | G | G | V | A | P | T | - | Y | N | V | P | D | V | P | D | T | F | A | Q | I | G | R | L | A | G | K | L | K | E | V | W | W | D | - | - | - | D | H | A | L | S | R | A | A | H | N | Y | I | L | R | N | V | D | Y | I | Q | K | - | F | E | R | - | - | - | - | - | - | - | - | - | - | - | - | - | - | - | - | - | - | - | - | - | - | - | - | - | - | - | - | - | - | - | - | - | - | - | - | - | - | - | - | - | - | - | - | - | - | - | - | - | - | - | - | - | - | - | - | - | - | - | - | - | - | - | - | - | - | - | - | - | - | - | - | - | - | - | - | - | - | - | - | - | - | - | - | - | - | - | - | - | - | - | - | - | - | - | - | - | - | - | - | - | - | - | - | - | - | - | - | - | - | - | - | - | - | - | - | - | - | - | - | - | - | - | - | - | - | - | - | - | - | - | - | - | - | - | - | - | - | - | - | - | - | - | - | - | - | - | - | - | - | - | - | - | - | - | - | - | - | - | - | - | - | - | - | - | - | - | - | - | - | - | - | - | - | - | - | - | - | - | - | - | - | S | K | I | T | T | A | L | M | Y | F | T | R | G | Y | T | F | H | T | Y | P | Y | G | S | R | - | - | - | - | R | A | T | A | N | Y | G | I | H | V | E | G | E | - | - | - | - | - | - | - | - | - | - | - | - | - | - | - | - | T | E | F | Y | G | I | L | Q | Q | I | L | E | V | E | Y | - | - | - | - | - | P | G | L | L | N | L | K | C | V | L | F | K | - | C | D | W | F | D | - | - | - | - | - | - | - | - | - | - | - | - | P | V | I | G | R | G | V | R | V | N | N | L | - | G | V | V | D | - | - | - | - | - | - | - | - | - | - | - | V | N | A | - | - | - | - | - | N | K | T | Y | - | - | A | K | F | E | P | F | I | L | A | S | Q | A | G | Q | V | S | F | L | S | Y | P | - | - | R | V | R | S | R | R | E | - | - | - | - | - | V | - | - | - | - | - | - | - | W | L | S | V | I | K | V | N | P | R | G | R | I | V | G | L | V | - | - | - | D | D | V | V | M | Q | Q | E | - | - | - | - | - | - | - | - | - | - | - | - | S | I | R | E | - | V | S | I | P | D | I | T | T | E | D | V | I | H | I | D | L | Q | N | R | E | L | E | D | I | T | H | D | G | S | E | E | E | E | Q | - | - | - | - | - | - | - | - | - | - | - | - | - | - | - | - | - | - | - | - | - | - | - | - | - | - | - | - | - | - | - | - | - | - | - | - | - | - | - | - | - | - | - | - | - | - | - | - | - | - | - | - | - | - | - | - | - | - | - | - | - | - | - | - | - | - | - | - | - | - | - | - | - | - | - | - | - | - | - | - | - | - | - | - | - | - | - | - | - | - | - | - | - | - | - | - | - | - | - | - | - | - | - | - | - | - | - | - | - | - | - | - | - | - | - | - | - | - | - | - | - | - | - | - | - | - | - | - | - | - | - | - | - | - | - | - | - | - | - | - | - | - | - | - | - | - | - | - | - | - | - | - | - | - | - | - | - | - | - | - | - | - | - | - | - | - | - | - | - | - | - | - | - | - | - | - | - | - | - | - | - | - | - | - | - | - | - | - | - | - | - | - | - | - | - | - | - | - | - | - | - | - | - | - | - | - | - | - | - | - | - | - | - | - | - | - | - | - | - | - | - | - | - | - | - | - | - | - | - | - | - | - | - | - | - | - | - | - | - | - | - | - | - | - | - | - | - | - | - | - | - | - | - | - | - | - | - | - | - | - | - | - | - | - | - | - | - | - | - | - | - | - | - | - | - | - | - | - | - | - | - | - | - | - | - | - | - | - | - | - | - | - | - | - | - | - | - | - | - | - | - | - | - | - | - | - | - | - | - | - | - | - | - | - | - | - | - | - | - | - | - | - | - | - | - | - | - | - | - | - | - | - | - | - | - | - | - | - | - | - | - | - | - | - | - | - | - | - | - | - | - | - | - | - | - | - | - | - | - | - | - | - | - | - | - | - | - | - | - | - | - | - | - | - | - | - | - | - | - | - | - | - | - | - | - | - | - | - | - | - | - | - | - | - | - | - | - | - | - | - | - | - | - | - | - | - | - | - | - | - | - | - | - | - | - | - | - | - | - | - | - | - | - | - | - | - | - | - | - | - | - | - | - | - | - | - | - | - | - | - | - | - | - | - | - | - | - | - | - | - | - | - | - | - | - | - | - | - | - | - | - | - | - | - | - | - | - | - | - | - | - | - | - | - | - | - | - | - | - | - | - | - | - | - | - | - | - | - | - | - | - | - | - | - | - | - | - | - | - | - | - | - | - | - | - | - | - | - | - | - | - | - | - | - | - | - | - | - | - | - | - | - | - | - | - | - | - | - | - | - | - | - | - | - | - | - | - | - | - | - | - | - | - | - | - | - | - | - | - | - | - | - | - | - | - | - | - | - | - | - | - | - | - | - | - | - | - | - | - | - | - | - | - | - | - | - | - | - | - | - | - | - | - | - | - | - | - | - | - | - | - | - | - | - | - | - | - | - | - | - | - | - | - | - | - | - | - | - | - | - | - | - | - | - | - | - | - | - | - | - | - | - | - | - | - | - | - | - | - | - | - | - | - | - | - | - | - | - | - | - | - | - | - | - | - | - | - | - | - | - | - | - | - | - | - | - | - | - | - | - | - | - | - | - | - | - | - | - | - | - | - | - | - | - | - | - | - | - | - | - | - | - | - | - | - | - | - | - | - | - | - | - | - | - | - | - | - | - | - | - | - | - | - | - | - | - | - | - | - | - | - | - | - | - | - | - | - | - | - | - | - | - | - | - | - | - | - | - | - | - | - | - | - | - | - | - | - | - | - | - | - | - | - | - | - | - | - | - | - | - | - | - | - | - | - | - | - | - | - | - | - | - | - | - | - | - | - | - | - | - | - | - | - | - | - | - | - | - | - | - | - | - | - | - | - | - | - | - | - | - | - | - | - | - |
|  | PSL\_Phyb | - | - | - | - | - | - | - | - | - | - | - | - | - | - | - | M | E | N | L | D | R | - | G | W | M | Y | E | R | L | D | G | - | R | G | G | Y | N | S | N | F | I | T | G | V | D | N | F | L | K | F | A | C | S | Q | Q | N | R | - | M | S | - | G | C | N | V | R | C | P | C | K | I | C | K | N | I | K | Y | W | D | - | V | - | - | - | - | E | T | V | R | L | H | L | Y | Q | S | G | F | V | E | N | Y | F | V | - | - | - | W | V | Y | Q | - | G | E | - | - | K | A | M | I | S | A | L | S | S | R | S | N | F | D | - | - | - | - | - | - | - | - | - | - | - | - | - | - | - | - | - | - | - | - | - | - | - | - | - | - | - | - | - | - | - | - | - | - | - | - | - | - | - | - | - | - | - | G | G | S | - | - | - | - | Q | S | E | L | G | Y | E | N | P | Y | R | Q | M | V | L | D | I | A | G | P | N | F | G | P | N | I | E | Q | G | S | S | W | Q | S | D | - | - | - | - | - | - | - | - | S | N | V | E | P | E | S | S | H | H | F | E | S | P | M | E | E | E | P | N | - | - | - | - | - | - | - | - | - | - | - | P | E | S | Q | R | F | Y | D | L | L | H | A | A | D | A | E | L | Y | - | - | - | P | G | S | - | - | S | L | S | Q | L | A | V | V | S | R | M | L | S | I | K | M | E | N | T | L | S | - | - | - | - | - | Q | R | G | Y | N | Q | M | M | Q | L | L | - | K | E | A | L | P | E | D | N | K | V | L | D | S | Y | Y | Q | T | K | K | L | V | Q | S | L | G | L | P | V | E | K | I | D | C | C | N | S | G | C | M | L | Y | W | G | D | - | D | E | D | L | T | S | C | K | F | C | G | H | D | R | Y | K | R | R | V | G | - | - | - | - | - | - | - | - | - | - | - | - | - | - | - | - | - | - | - | - | - | - | - | - | - | - | - | - | - | - | - | - | - | - | - | - | - | - | - | - | T | R | K | R | K | S | V | P | Y | K | K | M | Y | Y | F | P | L | I | P | R | L | Q | R | L | Y | A | S | H | A | T | A | S | D | M | R | W | H | H | E | H | - | - | - | - | - | - | - | - | - | - | I | Q | - | - | - | E | E | G | V | - | - | - | - | - | M | R | H | P | S | D | S | E | A | W | K | H | F | N | E | T | H | S | F | - | F | A | N | E | P | R | N | I | R | L | G | L | C | T | D | G | F | Q | P | F | G | Q | S | G | R | K | Y | S | S | W | P | V | I | L | T | P | Y | N | L | - | P | P | W | M | C | M | K | E | A | Y | M | F | L | T | I | I | V | P | G | P | N | N | P | K | Q | K | I | - | D | V | Y | L | Q | P | L | I | K | E | L | T | L | L | W | E | - | T | G | V | E | A | F | D | I | S | K | K | Q | N | F | Q | L | R | A | A | L | M | W | T | I | S | D | F | P | A | Y | S | M | L | S | G | W | S | T | A | G | N | K | A | - | - | C | P | Y | C | M | E | E | A | Q | S | F | R | L | R | H | G | R | K | T | S | W | F | D | S | H | R | M | F | L | D | Q | N | H | P | F | R | - | - | R | D | R | K | N | F | L | K | G | Q | T | V | R | M | P | - | P | P | P | L | R | T | - | G | E | E | I | L | - | N | Q | I | I | E | L | G | - | - | - | - | - | - | - | - | - | - | - | - | - | - | - | - | - | - | - | - | - | - | - | - | - | - | - | - | - | - | - | - | - | - | - | - | - | - | - | - | - | - | - | - | - | - | - | - | - | - | - | - | - | - | - | - | - | - | - | - | - | - | - | - | - | - | - | - | - | - | - | - | - | - | - | - | - | - | - | - | - | - | - | - | - | - | - | - | - | - | - | - | - | - | - | - | - | - | - | - | - | - | - | - | - | - | - | - | - | - | - | - | - | - | - | - | - | - | - | - | - | - | - | - | - | - | - | - | - | - | - | - | - | - | - | - | - | - | - | - | - | - | - | - | - | - | - | - | - | - | - | - | - | - | - | - | - | - | - | - | - | - | - | - | - | - | - | - | - | - | - | - | - | - | - | - | - | - | - | - | - | - | - | - | L | R | K | V | T | E | L | D | A | E | E | V | N | G | - | - | - | - | - | - | - | - | - | - | - | - | - | - | - | - | - | - | - | - | - | - | - | - | - | - | - | - | - | - | - | - | - | - | - | - | - | - | - | - | - | - | - | - | - | - | - | - | - | - | - | - | - | - | - | - | - | - | - | - | - | - | - | - | - | - | - | - | - | - | - | - | - | - | - | - | - | - | - | - | - | - | - | - | - | - | - | - | - | - | - | - | - | - | - | - | - | - | - | - | - | - | - | - | - | - | - | - | - | - | - | - | - | - | - | - | - | - | - | - | - | - | - | - | - | - | - | - | - | - | - | - | - | - | - | - | - | - | - | - | - | - | - | - | - | - | - | - | - | - | - | - | - | - | - | - | - | - | - | - | - | - | - | - | - | - | - | R | I | - | - | - | - | - | - | - | - | - | - | - | - | - | - | - | - | - | - | - | - | - | - | - | - | - | - | - | - | - | - | - | - | - | - | - | - | - | - | - | - | - | - | - | - | - | - | - | - | - | - | - | - | - | - | - | - | - | - | - | - | - | - | - | - | - | - | - | - | - | - | - | - | - | - | - | - | - | - | - | - | - | - | - | - | - | - | - | - | - | - | - | - | - | - | - | - | - | - | - | - | - | - | - | - | - | - | - | - | - | - | - | - | - | - | - | - | - | - | - | - | - | - | - | - | - | - | - | - | - | - | - | - | - | - | - | - | - | - | - | - | - | - | - | - | - | - | - | - | - | - | - | - | - | - | - | - | - | - | - | - | - | - | - | - | - | - | - | - | - | - | - | - | - | - | - | - | - | - | - | - | - | - | - | - | - | - | - | - | - | - | - | - | - | - | - | - | - | - | - | - | - | - | - | - | - | - | - | - | - | - | - | - | - | - | - | - | - | - | - | - | - | - | - | - | - | - | - | - | - | - | - | - | - | - | - | - | - | - | - | - | - | - | - | - | - | - | - | - | - | - | - | - | - | - | - | - | - | - | - | - | - | - | - | - | - | - | - | - | - | - | - | - | - | - | - | - | - | - | - | - | - | - | - | - | - | - | - | - | - | - | - | - | - | - | - | - | - | - | - | - | - | - | - | - | - | - | - | - | - | - | - | - | - | - | - | - | - | - | - | - | - | - | - | - | - | - | - | - | - | - | - | - | - | - | - | - | - | - | - | - | - | - | - | - | - | - | - | - | - | - | - | - | - | - | - | - | - | - | - | - | - | - | - | - | - | - | - | - | - | - | - | - | - | - | - | - | - | - | - | - | - | - | - | - | - | - | - | - | - | - | - | - | - | - | - | - | - | - | - | - | - | - | - | - | - | - | - | - | - | - | - | - | - | - | - | - | - | - | - | - | - | - | - | - | - | - | - | - | - | - | - | - | - | - | - | - | - | - | - | - | - | - | - | - | - | - | - | - | - | - | - | - | - | - | - | - | - | - | - | - | - | - | - | - | C | R | S | - | - | - | - | - | - | - | - | - | - | - | - | - | - | - | - | - | - | - | - | - | - | - | - | - | - | - | - | - | - | - | - | - | - | - | - | - | - | - | - | - | - | - | - | - | - | - | - | - | - | - | - | - | - | - | - | - | - | - | - | - | - | - | - | - | - | - | - | - | - | - | - | - | - | - | - | - | - | - | - | - | - | - | - | - | - | - | - | - | - | - | - | - | - | - | - | - | - | - | - | - | - | - | - | - | - | - | - | - | - | - | - | - | - | - | - | - | - | - | - | - | - | - | - | - | - | - | - | - | - | - | - | - | - | - | - | - | - | - | - | - | - | - | - | - | - | - | - | - | - | - | - | - | - | - | - | - | - | - | - | - | - | - | - | - | - | - | - | - | - | - | - | - | - | - | - | - | - | - | - | - | - | - | - | - | - | - | - | - | - | - | - | - | - | - | - | - | - | - | - | - | - | - | - | - | - | - | - | - | - | - | - | - | - | - | - | - | - | - | - | - | - | - | - | - | - | - | - | - | - | - | - | - | - | - | - | - | - | - | - | - | - | - | - | - | - | - | - | - | - | - | - | - | - | - | - | - | - | - | - | - | - | - | - | - | - | - | - | - | - | - | - | - | - | - | - | - | - | - | - | - | - | - | - | - | - | - | - | - | - | - | - | - | - | - | - | - | - | - | - | - | - | - | - | - | - | - | - | - | - | - | - | - | - | - | - | - | - | - | - | - | - | - | - | - | - | - | - | - | - | - | - | - | - | - | - | - | - | - | - | - | - | - | - | - | - | - | - | - | - | - | - | - | - | - | - | - | - | - | - | - | - | - | - | - | - | - | - | - | - | - | - | - | - | - | - | - | - | - | - | - | - | - | - | - | - | - | - | - | - | - | - | - | - | - | - | - | - | - | - | - | - | - | - | - | - | - | - | - | - | - | - | - | - | - | - | - | - | - | - | - | - | - | - | - | - | - | - | - | - | - | - | - | - | - | - | - | - | - | - | - | - | - | - | - | - | - | - | - | - | - | - | - | - | - | - | - | - | - | - | - | - | - | - | - | - | - | - | - | - | - | - | - | - | - | - | - | - | - | - | - | - | - | - | - | - | - | - | - | - | - | - | - | - | - | - | - | - | - | - | - | - | - | - | - | - | - | - | - | - | - | - | - | - | - | - | - | - | - | - | - | - | - | - | - | - | - | - | - | - | - | - | - | - | - | - | - | - | - | - | - | - | - | - | - | - | - | - | - | - | - | - | - | - | - | - | - | - | - | - | - | - | - | - | - | - | - | - | - | - | - | - | - | - | - | - | - | - | - | - | - | - | - | - | - | - | - | - | - | - | - | - | - | - | - | - | - | - | - | - | - | - | - | - | - | - | - | - | - | - | - | - | - | - | - | - | - | - | - | - | - | - | - | - | - | - | - | - | - | - | - | - | - | - | - | - | - | - | - | - | - | - | - | - | - | - | - | - | - | - | - | - | - | - | - | - | - | - | - | - | - | - | - | - | - | - | - | - | - | - | - | - | - | - | - | - | - | - | - | - | - | - | - | - | - | - | - | - | - | - | - | - | - | - | - | - | - | - | - | - | - | - | - | - | - | - | - | - | - | - | - | - | - | - | - | - | - | - | - | - | - | - | - | - | - | - | - | - | - | - | - | - | - | - | - | - | - | - | - | - | - | - | - | - | - | - | - | - | - | - | - | - | - | - | - | - | - | - | - | - | - | - | - | - | - | - | - | - | - | - | - | - | - | - | - | - | - | - | - | - | - | - | - | - | - | - | - | - | - | - | - | - | - | - | - | - | - | - | - | - | - | - | - | - | - | - | - | - | - | - | - | - | - | - | - | - | - | - | - | - | - | - | - | - | - | - | - | - | - | - | - | - | - | - | - | - | - | - | - | - | - | - | - | - | - | - | - | - | - | - | - | - | - | - | - | - | - | - | - | - | - | - | - | - | - | - | - | - | - | - | - | - | - | - | - | - | - | - | - | - | - | - | - | - | - | - | - | - | - | - | - | - | - | - | - | - | - | - | - | - | - | - | - | - | - | - | - | - | - | - | - | - | - | - | - | - | - | - | - | - | - | - | - | - | - | - | - | - | - | - | - | - | - | - | - | - | - | - | - | - | - | - | - | - | - | - | - | - | - | - | - | - | - | - | - | - | - | - | - | - | - | - | - | - | - | - | - | - | - | - | - | - |
|  | Alfred | - | - | - | - | - | - | - | - | - | - | - | - | - | - | - | - | - | M | E | D | R | - | E | W | M | Y | T | G | R | R | G | - | R | N | D | V | T | T | E | W | I | R | K | T | D | D | F | V | E | R | A | Y | G | E | A | A | - | - | K | G | - | A | S | L | V | P | C | P | C | S | K | C | A | N | R | K | R | K | P | - | K | - | - | - | - | K | A | M | V | E | H | I | W | K | N | G | F | T | P | G | Y | T | R | - | - | - | W | I | F | H | - | G | E | - | - | A | H | R | T | R | E | E | V | L | R | Q | R | - | - | - | - | - | - | - | - | - | - | - | - | - | - | - | - | - | - | - | - | - | - | - | - | - | - | - | - | - | - | - | - | - | - | - | - | - | - | - | - | - | - | - | - | - | V | E | D | - | - | - | - | - | - | - | Y | D | A | D | A | G | V | A | D | M | L | N | D | Y | Q | E | A | Q | Y | T | G | - | - | - | - | - | - | - | - | - | - | - | - | - | - | - | - | - | - | - | - | - | - | - | - | - | - | - | - | - | - | G | C | - | M | D | D | E | P | E | - | - | - | - | - | - | - | - | - | - | - | P | T | A | K | A | F | Y | D | M | F | D | A | A | Q | K | P | L | H | - | - | - | G | Q | T | - | - | K | V | S | Q | L | D | A | I | G | R | V | M | A | F | K | S | Q | Y | S | M | S | - | - | - | - | - | R | D | A | F | D | G | L | L | T | V | I | - | G | S | L | L | P | D | D | H | V | L | P | K | S | M | Y | E | A | Q | K | L | L | R | A | L | K | M | T | Y | E | Q | I | H | A | C | P | K | G | C | V | L | F | R | K | E | - | Y | A | E | A | K | Y | C | P | K | C | K | S | S | R | F | M | E | V | D | S | G | D | G | - | - | - | - | - | - | - | - | - | - | - | - | - | - | - | - | - | - | - | - | - | - | - | - | - | - | - | - | - | - | - | - | - | - | - | - | - | Q | K | R | Q | L | D | I | P | L | T | I | L | R | H | L | P | F | I | P | R | I | Q | R | L | Y | M | T | E | E | S | A | K | Q | M | T | W | H | K | N | G | - | - | - | - | - | - | - | - | - | - | K | R | - | - | Y | N | P | D | K | - | - | - | - | - | M | V | H | A | S | D | G | E | A | W | K | H | F | D | A | I | H | R | E | - | K | A | E | E | A | R | N | V | R | V | A | L | A | T | D | G | F | N | P | Y | G | M | S | A | A | P | Y | T | C | W | P | V | F | V | I | P | I | N | L | - | P | P | G | V | C | F | Q | R | Q | N | I | F | V | S | L | I | I | P | G | - | - | H | P | G | N | K | M | - | G | V | Y | M | E | P | L | I | D | E | L | V | R | A | W | E | - | E | G | V | W | T | Y | D | R | A | T | K | T | N | F | R | M | H | V | W | Y | Q | Y | S | M | H | D | L | P | A | Y | G | L | F | C | A | W | C | V | H | G | K | F | P | - | - | C | P | V | C | K | E | A | L | R | F | I | W | L | K | K | G | G | K | Y | S | S | F | D | K | H | R | Q | F | L | P | P | D | H | P | F | R | - | - | L | D | I | K | N | F | T | K | G | V | V | V | T | D | R | - | P | P | A | T | M | T | - | G | A | E | I | R | - | Q | Q | I | D | G | L | V | - | A | N | - | - | - | - | - | - | - | - | - | - | - | - | - | - | - | - | - | - | - | - | - | - | - | - | - | - | - | - | - | - | - | - | - | - | - | - | - | - | - | - | - | - | - | - | - | T | E | G | G | F | V | G | Y | - | G | E | Q | H | M | W | T | H | K | S | G | L | T | R | - | L | P | Y | Y | D | - | - | D | L | L | L | P | H | N | I | D | V | M | H | T | E | K | N | V | A | E | A | L | W | A | T | I | M | D | I | P | D | K | S | K | D | N | V | K | A | R | V | D | L | A | A | L | C | D | R | P | N | Q | E | M | K | P | P | S | G | - | - | - | - | - | - | - | - | - | G | K | T | W | R | R | P | K | A | D | F | V | L | S | R | A | Q | R | K | E | V | L | Q | W | I | - | K | M | L | M | F | P | D | G | Y | A | A | N | L | S | R | G | V | N | L | S | T | M | R | V | L | G | - | M | K | S | H | D | F | H | I | W | I | E | R | I | L | P | A | M | V | R | G | Y | - | - | - | - | - | - | - | V | P | E | - | H | V | W | L | A | L | S | - | - | - | - | - | - | - | - | - | - | - | - | - | - | - | - | - | - | - | - | - | - | - | E | L | S | Y | F | F | R | Q | L | C | A | K | E | L | S | R | T | V | V | A | D | L | E | R | L | A | P | V | L | L | C | K | L | E | K | I | F | P | P | G | - | - | - | - | F | F | N | P | M | Q | H | L | I | L | H | L | P | Y | E | A | R | M | G | G | P | V | Q | G | R | W | C | Y | P | I | E | - | - | - | - | - | - | - | - | - | - | - | - | - | - | - | - | - | - | - | - | - | - | - | - | - | - | - | - | - | R | C | - | - | - | - | - | - | - | - | - | - | - | - | - | - | - | - | - | - | - | - | - | - | - | - | - | - | - | - | - | - | - | - | - | - | - | - | - | - | - | - | - | - | - | - | - | - | - | - | - | - | - | - | - | - | - | - | - | - | - | - | - | - | - | - | - | - | - | - | - | - | - | - | - | - | L | K | T | I | R | K | K | C | R | N | K | C | K | I | E | A | S | I | A | E | A | Y | I | L | E | E | V | S | N | - | - | - | - | F | T | T | T | Y | Y | G | D | K | L | P | - | - | S | V | H | N | P | P | P | R | Y | N | D | G | D | N | E | - | - | - | - | - | - | - | S | N | L | S | I | F | R | G | Q | L | G | S | A | S | G | S | T | T | K | T | L | T | - | - | - | - | H | E | E | W | R | H | I | M | L | Y | V | L | T | N | L | E | E | V | T | P | - | Y | M | E | Q | F | L | H | E | F | W | R | R | S | R | - | - | - | - | - | - | - | - | - | - | - | - | - | - | - | - | - | - | - | - | - | - | - | - | - | - | - | - | - | - | - | - | - | - | - | - | - | - | - | - | - | - | - | - | - | - | - | - | - | - | - | - | - | - | - | - | - | - | - | - | - | - | - | - | - | - | - | - | - | - | - | - | - | - | - | - | - | - | - | - | - | - | - | - | D | P | T | P | Q | E | Y | D | T | L | L | R | K | G | - | - | - | - | - | A | R | N | G | L | P | D | F | I | S | W | F | K | R | K | V | G | Q | - | - | - | - | - | - | - | - | - | - | - | - | - | - | - | - | - | - | - | - | - | - | - | - | - | - | - | - | - | - | - | - | - | R | D | P | S | M | S | A | E | L | R | Q | V | A | N | G | F | A | Y | R | V | K | K | Y | S | R | Y | D | V | N | G | Y | R | F | R | T | T | N | Y | D | K | S | - | R | P | N | R | K | T | T | C | S | G | V | F | T | P | G | L | D | D | - | - | - | - | - | - | - | - | - | - | - | - | - | - | V | D | Y | F | G | R | I | E | E | I | Y | E | L | N | F | - | - | - | - | - | Y | G | S | K | P | L | T | P | V | I | F | K | - | C | H | W | F | D | - | - | - | - | - | - | - | - | - | - | - | - | P | Q | V | T | - | - | R | R | T | H | S | N | L | G | I | V | E | - | - | - | - | - | - | - | - | - | - | - | I | R | Q | - | - | - | - | - | D | S | T | L | - | - | P | G | D | D | V | Y | I | V | A | Q | Q | A | T | Q | V | Y | Y | L | P | Y | A | - | - | C | Q | T | K | E | H | L | - | K | G | - | - | - | - | - | - | - | - | - | - | W | D | V | V | Y | K | V | S | P | H | G | R | L | P | V | - | P | - | - | - | N | D | E | D | Y | N | - | - | - | - | - | L | D | P | D | T | Y | D | G | E | F | F | Q | E | - | D | G | L | - | - | - | - | - | E | G | R | F | E | I | D | L | T | E | A | I | G | M | D | V | D | I | E | M | V | V | D | E | E | D | D | E | - | - | - | - | - | - | - | - | - | - | - | - | - | - | - | - | - | - | - | - | - | - | - | - | - | - | - | - | - | - | - | - | - | - | - | - | - | - | - | - | - | - | - | - | - | - | - | - | - | - | - | - | - | - | - | - | - | - | - | - | - | - | - | - | - | - | - | - | - | - | - | - | - | - | - | - | - | - | - | - | - | - | - | - | - | - | - | - | - | - | - | - | - | - | - | - | - | - | - | - | - | - | - | - | - | - | - | - | - | - | - | - | - | - | - | - | - | - | - | - | - | - | - | - | - | - | - | - | - | - | - | - | - | - | - | - | - | - | - | - | - | - | - | - | - | - | - | - | - | - | - | - | - | - | - | - | - | - | - | - | - | - | - | - | - | - | - | - | - | - | - | - | - | - | - | - | - | - | - | - | - | - | - | - | - | - | - | - | - | - | - | - | - | - | - | - | - | - | - | - | - | - | - | - | - | - | - | - | - | - | - | - | - | - | - | - | - | - | - | - | - | - | - | - | - | - | - | - | - | - | - | - | - | - | - | - | - | - | - | - | - | - | - | - | - | - | - | - | - | - | - | - | - | - | - | - | - | - | - | - | - | - | - | - | - | - | - | - | - | - | - | - | - | - | - | - | - | - | - | - | - | - | - | - | - | - | - | - | - | - | - | - | - | - | - | - | - | - | - | - | - | - | - | - | - | - | - | - | - | - | - | - | - | - | - | - | - | - | - | - | - | - | - | - | - | - | - | - | - | - | - | - | - | - | - | - | - | - | - | - | - | - | - | - | - | - | - | - | - | - | - | - | - | - | - | - | - | - | - | - | - | - | - | - | - | - | - | - | - | - | - | - | - | - | - | - | - | - | - | - | - | - | - | - | - | - | - | - | - | - | - | - | - | - | - | - | - | - | - | - | - | - | - | - | - | - | - | - | - | - | - | - | - | - | - | - | - | - | - | - | - | - | - | - | - | - | - | - | - | - | - | - | - | - | - | - | - | - | - | - | - | - | - | - | - | - | - | - | - | - | - | - | - | - | - | - | - | - | - | - | - | - | - | - | - | - | - | - | - | - | - | - | - | - | - | - | - | - | - | - | - | - | - | - | - | - | - | - | - | - | - | - | - | - | - | - | - | - | - | - | - | - | - | V | Q | N | D | N | D | L | V | I | L | E | G | N | D | I | N | D | E | L | A | S | S | D | G | V | E | F | E | M | V | D | S | D | D | E | S | Y | D | P | A | N | P | D | T | Y | E | D | Y | F | - | - | - | - | - | - | - | - | - | - | - | - | - | - | - | - | - | - | - | - | - | - | - | - | - | - | - | - | - | - | - | - | - | - | - | - | - | - | - | - | - | - | - | - | - | - | - | - | - | - | - | - | - | - | - | - | - | - | - | - | - | - | - | - | - | - | - | - | - | - | - | - | - | - | - | - | - | - | - | - | - | - | - | - | - | - | - | - | - | - | - | - | - | - | - | - | - | - | - | - | - | - | - | - | - | - | - | - | - | - | - | - | - | - | - | - | - | - | - | - | - | - | - | - | - | - | - | - | - | - | - | - | - | - | - | - | - | - | - | - | - | - | - | - | - | - | - | - | - | - | - | - | - | - | - | - | - | - | - | - | - | - | - | - | - | - | - | - | - | - | - | - | - | - | - | - | - | - | - | - | - | - | - | - | - | - | - | - | - | - | - | - | - | - | - | - | - | - | - | - | - | - | - | - | - | - | - | - | - | - | - | - | - | - | - | - | - | - | - | - | - | - | - | - | - | - | - | - | - | - | - | - | - | - | - | - | - | - | - | - |
|  | EnSpm12\_Sbic | - | - | - | - | - | - | - | - | - | - | - | - | - | - | - | - | - | M | D | D | R | - | E | W | M | Y | S | G | R | S | S | - | F | G | E | W | T | D | E | W | I | K | K | T | D | A | F | L | E | T | A | F | R | L | A | - | - | - | K | G | - | A | R | K | I | W | C | P | C | S | S | C | E | N | G | L | R | Q | T | - | K | - | - | - | - | T | D | M | G | K | H | L | C | K | Y | G | F | M | P | G | Y | I | R | - | - | - | W | T | L | H | - | G | E | - | - | P | D | R | M | R | D | E | V | V | R | Q | R | - | - | - | - | - | - | - | - | - | - | - | - | - | - | - | - | - | - | - | - | - | - | - | - | - | - | - | - | - | - | - | - | - | - | - | - | - | - | - | - | - | - | - | - | - | V | A | E | - | - | - | - | - | - | - | F | E | D | E | G | G | V | A | D | M | I | D | D | F | H | D | G | N | F | I | E | - | - | - | - | - | - | - | - | - | - | - | - | - | - | - | - | - | - | - | - | - | - | - | - | - | - | - | - | - | - | G | R | S | E | D | E | E | P | E | - | - | - | - | - | - | - | - | - | - | - | A | T | A | K | A | Y | Y | D | M | L | E | A | A | Q | K | P | L | H | - | - | - | E | Q | T | - | - | N | V | S | Q | L | D | A | I | G | R | L | I | D | L | K | S | Q | L | N | M | S | - | - | - | - | - | R | A | G | F | D | V | M | L | T | V | F | - | G | S | I | L | P | K | G | H | I | L | P | K | N | M | Y | E | S | Q | K | L | L | R | A | L | K | M | P | Y | E | P | I | H | A | C | E | H | G | C | I | L | F | R | E | E | - | H | A | E | A | T | H | C | P | K | C | G | S | S | R | F | V | E | Q | H | H | G | D | G | - | - | - | - | - | - | - | - | - | - | - | - | - | - | - | - | - | - | - | - | - | - | - | - | - | - | - | - | - | - | - | - | - | - | - | - | - | R | I | E | Q | L | S | I | P | V | K | I | L | R | Y | L | P | V | I | P | R | L | Q | R | L | Y | M | T | E | E | S | A | K | Q | M | T | W | H | K | H | G | - | - | - | - | - | - | - | - | - | - | R | R | - | - | Y | N | P | E | K | - | - | - | - | - | M | V | H | P | S | D | G | E | A | W | T | H | F | D | S | I | H | H | E | - | K | A | L | E | A | R | N | V | R | V | A | F | A | T | D | G | F | N | P | Y | G | M | M | A | A | P | Y | T | C | W | P | V | F | V | I | P | L | N | L | - | P | P | G | V | C | F | Q | A | Q | N | I | F | L | S | L | I | I | P | G | - | - | H | P | G | N | N | M | - | G | V | Y | M | A | P | L | I | D | E | L | I | K | S | W | D | - | E | G | V | L | T | F | D | R | A | T | K | T | N | F | T | M | H | V | W | Y | H | Y | S | L | H | D | F | L | A | Y | G | I | F | C | G | W | C | V | H | G | K | F | P | - | - | C | P | T | C | K | T | N | V | Q | F | I | W | L | K | K | G | G | K | Y | S | S | F | D | K | H | R | Q | F | L | P | I | E | H | P | F | R | - | - | Q | D | I | K | N | F | T | K | G | V | V | V | T | D | P | - | P | P | E | M | M | T | - | G | V | E | V | R | - | A | Q | I | D | A | L | E | - | V | S | - | - | - | - | - | - | - | - | - | - | - | - | - | - | - | - | - | - | - | - | - | - | - | - | - | - | - | - | - | - | - | - | - | - | - | - | - | - | - | - | - | - | - | - | - | E | G | G | G | F | V | G | Y | - | G | H | E | H | A | W | T | H | K | S | G | L | E | R | - | L | P | Y | Y | Q | - | - | D | L | L | L | P | H | N | I | D | V | M | H | T | E | K | N | I | A | E | A | L | W | G | T | I | M | D | I | K | E | K | S | K | D | N | V | K | A | R | I | D | L | A | A | I | C | D | R | P | N | Q | E | M | R | P | P | R | A | - | - | - | - | - | - | - | - | - | G | K | T | W | R | R | P | P | A | D | Y | M | L | T | R | P | Q | R | R | E | V | L | E | W | F | - | Q | N | L | M | F | P | D | G | Y | A | A | N | L | R | R | G | V | N | L | S | T | M | R | I | N | G | - | L | K | S | H | D | Y | H | I | W | I | E | R | L | L | P | V | M | V | R | G | Y | - | - | - | - | - | - | - | V | P | D | - | H | V | W | Q | V | L | A | - | - | - | - | - | - | - | - | - | - | - | - | - | - | - | - | - | - | - | - | - | - | - | E | L | S | N | F | F | R | Q | L | C | A | K | E | L | S | R | S | V | V | K | D | L | E | K | M | A | P | L | L | L | C | K | L | E | K | V | F | P | P | G | - | - | - | - | F | F | N | P | M | Q | H | L | I | L | H | L | P | Y | E | A | R | M | G | G | P | V | Q | F | R | W | C | Y | F | V | E | - | - | - | - | - | - | - | - | - | - | - | - | - | - | - | - | - | - | - | - | - | - | - | - | - | - | - | - | - | R | T | - | - | - | - | - | - | - | - | - | - | - | - | - | - | - | - | - | - | - | - | - | - | - | - | - | - | - | - | - | - | - | - | - | - | - | - | - | - | - | - | - | - | - | - | - | - | - | - | - | - | - | - | - | - | - | - | - | - | - | - | - | - | - | - | - | - | - | - | - | - | - | - | - | - | L | K | V | V | R | K | K | C | R | N | K | C | K | I | E | A | S | I | A | E | A | S | I | L | E | E | V | S | N | - | - | - | - | F | T | T | K | Y | Y | G | D | N | L | P | - | - | S | V | H | N | R | Q | L | R | Y | N | V | G | E | N | E | - | - | - | - | - | - | - | S | N | L | S | L | F | R | G | Q | L | G | S | A | S | D | W | T | Q | K | T | L | R | - | - | - | - | H | D | E | W | R | I | I | M | F | Y | V | L | S | N | L | T | E | V | Q | P | - | Y | I | G | - | - | - | - | - | - | - | - | - | - | - | - | - | - | - | - | - | - | - | - | - | - | - | - | - | - | - | - | - | - | - | - | - | - | - | - | - | - | - | - | - | - | - | - | - | - | - | - | - | - | - | - | - | - | - | - | - | - | - | - | - | - | - | - | - | - | - | - | - | - | - | - | - | - | - | - | - | - | - | - | - | - | - | - | - | - | - | - | - | - | - | - | - | - | - | - | - | - | - | - | - | - | - | - | - | - | - | - | - | - | - | - | - | - | - | - | - | - | - | - | - | - | - | - | - | - | - | - | - | - | - | - | - | - | - | - | - | - | - | - | - | - | - | - | - | - | - | - | - | - | - | - | - | - | - | - | - | - | - | - | - | - | - | - | - | - | - | - | - | - | - | - | - | - | - | - | - | - | - | - | - | - | - | - | - | - | - | - | - | - | - | - | - | - | - | - | - | - | - | - | - | - | - | - | - | - | - | - | - | - | - | - | - | - | - | - | - | - | - | - | - | - | - | - | - | - | - | - | - | - | - | - | - | - | - | - | - | - | - | - | - | - | - | - | - | - | - | - | - | - | - | - | - | - | - | - | - | - | - | - | - | - | - | - | - | - | - | - | - | - | - | - | - | - | - | - | - | - | - | - | - | - | - | - | - | - | - | - | - | - | - | - | - | - | - | - | - | - | - | - | - | - | - | - | - | - | - | - | - | - | - | - | - | - | - | - | - | - | - | - | - | - | - | - | - | - | - | - | - | - | - | - | - | - | - | - | - | - | - | - | - | - | - | - | - | - | - | - | - | - | - | - | - | - | - | - | - | - | - | - | - | - | - | - | - | - | - | - | - | - | - | - | - | - | - | - | - | - | - | - | - | - | - | - | - | - | - | - | - | - | - | - | - | - | - | - | - | - | - | - | - | - | - | - | - | - | - | - | - | - | - | - | - | - | - | - | - | - | - | - | - | - | - | - | - | - | - | - | - | - | - | - | - | - | - | - | - | - | - | - | - | - | - | - | - | - | - | - | - | - | - | - | - | - | - | - | - | - | - | - | - | - | - | - | - | - | - | - | - | - | - | - | - | - | - | - | - | - | - | - | - | - | - | - | - | - | - | - | - | - | - | - | - | - | - | - | - | - | - | - | - | - | - | - | - | - | - | - | - | - | - | - | - | - | - | - | - | - | - | - | - | - | - | - | - | - | - | - | - | - | - | - | - | - | - | - | - | - | - | - | - | - | - | - | - | - | - | - | - | - | - | - | - | - | - | - | - | - | - | - | - | - | - | - | - | - | - | - | - | - | - | - | - | - | - | - | - | - | - | - | - | - | - | - | - | - | - | - | - | - | - | - | - | - | - | - | - | - | - | - | - | - | - | - | - | - | - | - | - | - | - | - | - | - | - | - | - | - | - | - | - | - | - | - | - | - | - | - | - | - | - | - | - | - | - | - | - | - | - | - | - | - | - | - | - | - | - | - | - | - | - | - | - | - | - | - | - | - | - | - | - | - | - | - | - | - | - | - | - | - | - | - | - | - | - | - | - | - | - | - | - | - | - | - | - | - | - | - | - | - | - | - | - | - | - | - | - | - | - | - | - | - | - | - | - | - | - | - | - | - | - | - | - | - | - | - | - | - | - | - | - | - | - | - | - | - | - | - | - | - | - | - | - | - | - | - | - | - | - | - | - | - | - | - | - | - | - | - | - | - | - | - | - | - | - | - | - | - | - | - | - | - | - | - | - | - | - | - | - | - | - | - | - | - | - | - | - | - | - | - | - | - | - | - | - | - | - | - | - | - | - | - | - | - | - | - | - | - | - | - | - | - | - | - | - | - | - | - | - | - | - | - | - | - | - | - | - | - | - | - | - | - | - | - | - | - | - | - | - | - | - | - | - | - | - | - | - | - | - | - | - | - | - | - | - | - | - | - | - | - | - | - | - | - | - | - | - | - | - | - | - | - | - | - | - | - | - | - | - | - | - | - | - | - | - | - | - | - | - | - | - | - | - | - | - | - | - | - | - | - | - | - | - | - | - | - | - | - | - | - | - | - | - | - | - | - | - | - | - | - | - | - | - | - | - | - | - | - | - | - | - | - | - | - | - | - | - | - | - | - | - | - | - | - | - | - | - | - | - | - | - | - | - | - | - | - | - | - | - | - | - | - | - | - | - | - | - | - | - | - | - | - | - | - | - | - | - | - | - | - | - | - | - | - | - | - | - | - | - | - | - | - | - | - | - | - | - | - | - | - | - | - | - | - | - | - | - | - | - | - | - | - | - | - | - | - | - | - | - | - | - | - | - | - | - | - | - | - | - | - | - | - | - | - | - | - | - | - | - | - | - | - | - | - | - | - | - | - | - | - | - | - | - | - | - | - | - | - | - | - | - | - | - | - | - | - | - | - | - | - | - | - | - | - | - | - | - | - | - | - | - | - | - | - | - | - | - | - | - | - | - | - | - | - | - | - | - | - | - | - | - | - | - | - | - | - | - | - | - | - | - | - | - | - | - | - | - | - | - | - | - | - | - | - | - | - | - | - | - | - | - | - | - | - | - | - | - | - | - | - | - | - | - | - | - | - | - | - | - | - | - | - | - | - | - | - | - | - | - | - | - | - | - | - | - | - | - | - | - | - | - | - | - | - | - | - | - | - | - | - | - | - | - | - | - | - | - | - | - | - | - | - | - | - | - | - | - | - | - | - | - | - | - | - | - | - | - | - | - | - | - | - | - | - | - | - | - | - | - | - | - | - | - | - | - | - | - | - | - | - | - | - | - | - | - | - | - | - | - | - | - | - | - | - | - | - | - | - | - | - | - | - | - | - | - | - | - |
|  | EnSpm5\_Sbic | - | - | - | - | - | - | - | - | - | - | - | - | - | - | - | - | - | - | - | - | - | - | - | - | - | - | - | - | - | - | - | - | - | - | - | - | - | - | - | - | - | - | - | - | - | - | - | - | - | - | - | - | - | - | - | - | - | - | - | - | - | - | - | - | - | - | - | - | - | - | - | - | - | - | - | - | - | - | - | - | - | - | - | - | - | - | - | M | G | K | H | L | X | X | X | G | X | T | P | G | Y | X | R | - | - | - | W | V | Y | H | - | G | E | - | - | A | H | R | I | R | E | E | V | V | R | P | R | - | - | - | - | - | - | - | - | - | - | - | - | - | - | - | - | - | - | - | - | - | - | - | - | - | - | - | - | - | - | - | - | - | - | - | - | - | - | - | - | - | - | - | - | - | L | E | P | - | - | - | - | - | - | - | F | D | D | D | A | G | V | A | D | M | L | D | D | I | H | Q | A | Q | F | A | E | - | - | - | - | - | - | - | - | - | - | - | - | - | - | - | - | - | - | - | - | - | - | - | - | - | - | - | - | - | - | G | R | - | X | E | E | X | M | E | - | - | - | - | - | - | - | - | - | - | - | A | N | V | E | A | F | Y | X | M | L | E | S | A | X | K | P | L | H | - | - | - | G | H | T | - | - | D | I | S | Q | L | D | X | I | G | R | X | X | G | L | K | X | E | L | N | L | S | - | - | - | - | - | R | E | G | F | D | K | M | L | T | V | X | - | X | T | X | L | P | K | D | H | V | L | P | X | N | L | Y | E | S | X | K | L | L | R | A | L | K | M | P | Y | X | X | I | H | X | C | P | K | G | C | V | L | F | R | K | E | - | H | E | D | A | K | Y | C | P | K | C | G | A | S | R | Y | L | E | A | X | S | G | D | G | - | - | - | - | - | - | - | - | - | - | - | - | - | - | - | - | - | - | - | - | - | - | - | - | - | - | - | - | - | - | - | - | - | - | - | - | - | Q | K | E | Q | L | A | I | P | A | R | V | L | R | H | L | P | F | V | P | R | I | Q | R | L | F | M | T | E | E | T | A | K | Q | M | T | W | H | K | D | G | - | - | - | - | - | - | - | - | - | - | K | R | - | - | Y | H | P | D | K | - | - | - | - | - | M | V | H | P | A | D | G | E | A | W | A | H | F | D | D | R | H | X | E | - | K | A | D | E | A | R | N | V | R | V | A | L | A | T | D | G | F | N | P | Y | G | M | M | S | S | P | Y | T | C | W | P | V | F | A | I | P | L | N | L | - | P | P | G | V | A | F | R | R | Q | N | V | F | L | S | L | I | I | P | G | - | - | H | P | G | S | N | M | - | G | V | F | M | E | P | V | I | D | E | L | I | G | A | W | N | - | E | G | V | W | T | Y | D | R | A | T | R | S | S | F | K | M | H | V | W | Y | Q | Y | S | I | H | D | F | L | A | Y | G | L | F | C | A | W | C | V | H | G | K | F | P | - | - | C | P | V | C | K | E | A | V | R | F | I | W | L | K | K | G | G | K | Y | S | S | F | D | Q | H | R | Q | F | L | P | A | G | H | P | F | R | - | - | R | D | V | K | N | F | R | K | G | V | V | V | T | D | P | - | K | P | H | M | K | T | - | G | A | E | V | R | - | A | Q | I | D | A | L | V | - | P | X | - | - | - | - | - | - | - | - | - | - | - | - | - | - | - | - | - | - | - | - | - | - | - | - | - | - | - | - | - | - | - | - | - | - | - | - | - | - | - | - | - | - | - | - | - | E | G | G | G | F | V | G | Y | - | G | E | Q | H | M | W | T | H | K | S | G | L | T | R | - | L | P | Y | Y | E | - | - | D | L | L | L | P | H | N | I | D | V | M | H | T | E | K | N | V | A | E | A | L | W | A | T | L | M | D | T | - | E | K | S | K | D | N | P | K | A | R | V | D | L | A | T | L | C | D | R | P | K | Q | E | M | R | P | P | A | G | R | - | - | - | - | - | - | - | - | N | K | X | W | K | R | P | K | A | D | F | V | L | K | A | E | Q | R | R | E | V | L | Q | W | I | - | K | T | L | M | F | P | D | G | Y | A | A | N | X | S | R | G | V | N | L | T | T | L | R | V | N | G | - | M | K | S | H | D | Y | H | V | W | I | E | R | L | L | P | A | M | V | R | G | Y | - | - | - | - | - | - | - | V | P | E | - | R | V | W | I | V | L | A | - | - | - | - | - | - | - | - | - | - | - | - | - | - | - | - | - | - | - | - | - | - | - | E | L | S | Y | F | F | R | Q | L | C | A | K | E | V | S | R | T | V | A | I | D | L | E | K | A | A | P | V | L | L | C | K | L | E | K | I | F | P | P | G | - | - | - | - | F | F | L | P | M | Q | H | L | I | V | H | L | P | Y | E | A | R | L | G | G | P | V | Q | G | R | W | C | Y | T | V | E | - | - | - | - | - | - | - | - | - | - | - | - | - | - | - | - | - | - | - | - | - | - | - | - | - | - | - | - | - | R | A | - | - | - | - | - | - | - | - | - | - | - | - | - | - | - | - | - | - | - | - | - | - | - | - | - | - | - | - | - | - | - | - | - | - | - | - | - | - | - | - | - | - | - | - | - | - | - | - | - | - | - | - | - | - | - | - | - | - | - | - | - | - | - | - | - | - | - | - | - | - | - | - | - | - | L | K | T | V | R | K | K | C | R | N | K | A | R | I | E | A | S | V | A | G | A | F | V | L | E | E | V | A | N | - | - | - | - | F | T | H | C | T | T | R | R | I | F | R | A | C | T | T | H | - | - | - | - | - | - | - | - | - | - | - | - | - | - | - | - | - | - | - | - | - | - | - | - | - | - | - | - | - | - | - | - | - | - | - | - | - | - | - | - | - | - | - | - | - | - | - | - | - | - | - | - | - | - | - | - | - | - | - | - | - | - | - | - | - | - | - | - | - | - | - | - | - | - | - | - | - | - | - | - | - | - | - | - | - | - | - | - | - | - | - | - | - | - | - | - | - | - | - | - | - | - | - | - | - | - | - | - | - | - | - | - | - | - | - | - | - | - | - | - | - | - | - | - | - | - | - | - | - | - | - | - | - | - | - | - | - | - | - | - | - | - | - | - | - | - | - | - | - | - | - | - | - | - | - | - | - | - | - | - | - | - | - | - | - | - | - | - | - | - | - | - | - | - | - | - | - | - | - | - | - | - | - | - | - | - | - | - | - | - | - | - | - | - | - | - | - | - | - | - | - | - | - | - | - | - | - | - | - | - | - | - | - | - | - | - | - | - | - | - | - | - | - | - | - | - | - | - | - | - | - | - | - | - | - | - | - | - | - | - | - | - | - | - | - | - | - | - | - | - | - | - | - | - | - | - | - | - | - | - | - | - | - | - | - | - | - | - | - | - | - | - | - | - | - | - | - | - | - | - | - | - | - | - | - | - | - | - | - | - | - | - | - | - | - | - | - | - | - | - | - | - | - | - | - | - | - | - | - | - | - | - | - | - | - | - | - | - | - | - | - | - | - | - | - | - | - | - | - | - | - | - | - | - | - | - | - | - | - | - | - | - | - | - | - | - | - | - | - | - | - | - | - | - | - | - | - | - | P | L | V | T | M | S | T | K | M | N | R | P | - | - | - | - | - | - | - | - | - | - | - | - | - | - | - | - | - | - | - | - | - | - | - | - | - | - | - | - | - | - | - | - | - | - | - | - | - | - | - | - | - | - | - | - | - | - | - | - | - | - | - | - | - | - | - | - | - | - | - | - | - | - | - | - | - | - | - | - | - | - | - | - | - | - | - | - | - | - | - | - | - | - | - | - | - | - | - | - | - | - | - | - | - | - | - | - | - | - | - | - | - | - | - | - | - | - | - | - | - | - | - | - | - | - | - | - | - | - | - | - | - | - | - | - | - | - | - | - | - | - | - | - | - | - | - | - | - | - | - | - | - | - | - | - | - | - | - | - | - | - | - | - | - | - | - | - | - | - | - | - | - | - | - | - | - | - | - | - | - | - | - | - | - | - | - | - | - | - | - | - | - | - | - | - | - | - | - | - | - | - | - | - | - | - | - | - | - | - | - | - | - | - | - | - | - | - | - | - | - | - | - | - | - | - | - | - | - | - | - | - | - | - | - | - | - | - | - | - | - | - | - | - | - | - | - | - | - | - | - | - | - | - | - | - | - | - | - | - | - | - | - | - | - | - | - | - | - | - | - | - | - | - | - | - | - | - | - | - | - | - | - | - | - | - | - | - | - | - | - | - | - | - | - | - | - | - | - | - | - | - | - | - | - | - | - | - | - | - | - | - | - | - | - | - | - | - | - | - | - | - | - | - | - | - | - | - | - | - | - | - | - | - | - | - | - | - | - | - | - | - | - | - | - | - | - | - | - | - | - | - | - | - | - | - | - | - | - | - | - | - | - | - | - | - | - | - | - | - | - | - | - | - | - | - | - | - | - | - | - | - | - | - | - | - | - | - | - | - | - | - | - | - | - | - | - | - | - | - | - | - | - | - | - | - | - | - | - | - | - | - | - | - | - | - | - | - | - | - | - | - | - | - | - | - | - | - | - | - | - | - | - | - | - | - | - | - | - | - | - | - | - | - | - | - | - | - | - | - | - | - | - | - | - | - | - | - | - | - | - | - | - | - | - | - | - | - | - | - | - | - | - | - | - | - | - | - | - | - | - | - | - | - | - | - | - | - | - | - | - | - | - | - | - | - | - | - | - | - | - | - | - | - | - | - | - | - | - | - | - | - | - | - | - | - | - | - | - | - | - | - | - | - | - | - | - | - | - | - | - | - | - | - | - | - | - | - | - | - | - | - | - | - | - | - | - | - | - | - | - | - | - | - | - | - | - | - | - | - | - | - | - | - | - | - | - | - | - | - | - | - | - | - | - | - | - | - | - | - | - | - | - | - | - | - | - | - | - | - | - | - | - | - | - | - | - | - | - | - | - | - | - | - | - | - | - | - | - | - | - | - | - | - | - | - | - | - | - | - | - | - | - | - | - | - | - | - | - | - | - | - | - | - | - | - | - | - | - | - | - | - | - | - | - | - | - | - | - | - | - | - | - | - | - | - | - | - | - | - | - | - | - | - | - | - | - | - | - | - | - | - | - | - | - | - | - | - | - | - | - | - | - | - | - | - | - | - | - | - | - | - | - | - | - | - | - | - | - | - | - | - | - | - | - | - | - | - | - | - | - | - | - | - | - | - | - | - | - | - | - | - | - | - | - | - | - | - | - | - | - | - | - | - | - | - | - | - | - | - | - | - | - | - | - | - | - | - | - | - | - | - | - | - | - | - | - | - | - | - | - | - | - | - | - | - | - | - | - | - | - | - | - | - | - | - | - | - | - | - | - | - | - | - | - | - | - | - | - | - | - | - | - | - | - | - | - | - | - | - | - | - | - | - | - | - | - | - | - | - | - | - | - | - | - | - | - | - | - | - | - | - | - | - | - | - | - | - | - | - | - | - | - | - | - | - | - | - | - | - | - | - | - | - | - | - | - | - | - | - | - | - | - | - | - | - | - | - | - | - | - | - | - | - | - | - | - | - | - | - | - | - | - | - | - | - | - | - | - | - | - | - | - | - | - | - | - | - | - | - | - | - | - | - | - | - | - | - | - | - | - | - | - | - | - | - | - | - | - | - | - | - | - | - | - | - | - | - | - | - | - | - | - | - | - | - | - | - | - | - | - | - | - | - | - | - | - | - | - | - | - | - | - | - | - | - | - | - | - | - | - | - | - | - | - | - | - | - |
|  | EnSpm20\_Sbic | - | M | H | F | V | L | V | L | L | S | H | A | C | V | C | Q | R | M | E | N | R | - | E | W | M | Y | T | G | F | A | C | - | - | - | - | R | T | T | E | W | V | Q | K | S | M | A | F | L | E | H | A | F | G | E | A | A | - | - | K | G | - | S | R | R | M | P | C | P | C | S | K | C | K | N | M | S | R | F | E | - | K | - | - | - | - | S | E | M | W | K | H | I | F | K | Y | G | F | V | E | N | Y | T | R | - | - | - | W | I | Y | H | - | G | E | - | - | A | S | R | I | R | E | E | V | V | R | E | R | - | - | - | - | - | - | - | - | - | - | - | - | - | - | - | - | - | - | - | - | - | - | - | - | - | - | - | - | - | - | - | - | - | - | - | - | - | - | - | - | - | - | - | - | - | L | E | E | - | - | - | - | - | - | - | Y | D | C | D | G | G | V | P | D | M | M | E | D | A | H | D | A | Y | F | G | E | - | - | - | - | - | - | - | - | - | - | - | - | - | - | - | - | - | - | - | - | - | - | - | - | - | - | - | - | - | - | - | - | G | M | E | D | E | P | E | - | - | - | - | - | - | - | - | - | - | - | E | S | A | K | A | F | Y | E | M | L | K | S | A | Q | K | P | L | H | - | - | - | D | H | T | - | - | R | V | S | Q | L | D | A | L | G | R | L | M | G | M | K | S | Q | F | S | M | S | - | - | - | - | - | R | E | H | F | D | F | T | L | T | I | I | - | G | T | L | L | P | E | G | H | I | L | P | K | N | T | Y | E | S | K | K | L | L | R | A | L | K | M | P | Y | E | Q | I | H | A | C | K | E | G | C | V | L | F | R | G | H | - | L | K | E | A | T | H | C | P | K | C | N | S | S | R | Y | V | E | V | N | S | S | G | G | - | - | - | - | - | - | - | - | - | - | - | - | - | - | - | - | - | - | - | - | - | - | - | - | - | - | - | - | - | - | - | - | - | - | - | - | - | S | L | Q | Q | S | R | I | P | H | K | V | L | R | Y | L | P | F | I | P | R | I | Q | R | L | F | M | M | E | E | S | A | K | Q | M | T | W | H | K | N | G | - | - | - | - | - | - | - | - | - | - | K | R | - | - | Y | N | P | D | K | - | - | - | - | - | M | V | H | P | S | D | G | E | A | W | R | H | F | D | G | K | H | P | G | - | K | A | M | E | A | R | N | V | R | V | A | L | A | T | D | G | F | N | P | Y | G | M | M | A | V | P | Y | T | C | W | P | V | F | V | I | P | L | N | L | P | P | P | G | V | M | F | Q | P | K | N | I | F | L | S | L | I | I | P | G | - | - | H | P | G | N | N | M | - | G | V | F | M | Q | P | L | W | D | E | L | V | H | A | W | E | - | H | G | V | L | T | F | D | R | A | T | K | R | N | F | I | M | H | V | W | Y | Q | Y | S | M | H | D | F | L | A | Y | G | I | F | C | A | W | C | V | H | G | K | F | P | - | - | C | P | I | C | K | T | A | V | R | F | T | W | L | K | S | G | G | K | Y | S | S | F | D | Q | H | R | Q | F | L | P | P | N | H | P | F | R | - | - | R | D | X | K | H | F | R | K | G | V | Q | V | R | D | X | - | V | P | H | M | M | S | - | P | A | E | V | R | - | A | E | I | E | G | L | K | V | D | E | - | - | - | - | - | - | - | - | - | - | - | - | - | - | - | - | - | - | - | - | - | - | - | - | - | - | - | - | - | - | - | - | - | - | - | - | - | - | - | - | - | - | - | - | - | V | N | G | G | F | I | G | Y | - | A | D | E | H | M | W | T | H | T | S | G | L | T | K | - | L | P | Y | F | D | - | - | D | L | L | L | P | H | N | I | D | V | M | H | T | E | K | N | V | A | E | A | L | W | A | T | L | M | D | I | Q | E | K | S | K | D | N | V | K | A | R | L | D | L | E | T | L | C | D | R | P | N | L | V | M | K | P | P | A | A | - | - | - | - | - | - | - | - | - | G | K | R | W | K | R | T | P | A | D | Y | I | L | K | R | E | H | R | K | E | V | L | Q | W | M | - | K | T | L | L | F | P | D | G | Y | A | A | N | L | R | R | G | V | N | L | S | T | L | R | V | N | G | - | M | K | S | H | D | F | H | I | W | I | E | R | L | L | P | A | M | T | R | G | Y | - | - | - | - | - | - | - | L | P | E | - | P | V | W | R | V | L | A | - | - | - | - | - | - | - | - | - | - | - | - | - | - | - | - | - | - | - | - | - | - | - | E | L | S | Y | F | F | R | Q | L | C | A | K | E | L | S | R | D | V | V | R | D | L | E | S | V | A | P | V | L | L | C | K | L | E | M | I | F | P | P | G | - | - | - | - | F | F | L | P | M | Q | H | L | I | L | H | L | P | Y | E | A | K | M | G | G | P | V | Q | A | R | W | C | Y | P | I | E | - | - | - | - | - | - | - | - | - | - | - | - | - | - | - | - | - | - | - | - | - | - | - | - | - | - | - | - | - | R | C | - | - | - | - | - | - | - | - | - | - | - | - | - | - | - | - | - | - | - | - | - | - | - | - | - | - | - | - | - | - | - | - | - | - | - | - | - | - | - | - | - | - | - | - | - | - | - | - | - | - | - | - | - | - | - | - | - | - | - | - | - | - | - | - | - | - | - | - | - | - | - | - | - | - | L | K | V | M | R | K | K | C | R | N | K | A | R | I | E | A | S | I | A | E | A | S | I | L | E | E | V | S | N | - | - | - | - | F | T | T | N | Y | Y | T | D | N | L | P | - | - | S | V | H | N | P | L | S | R | Y | N | D | N | E | S | S | - | - | - | - | - | - | - | S | N | L | S | L | F | K | G | Q | L | G | R | A | S | A | G | T | P | K | T | L | C | - | - | - | - | Y | L | E | W | R | K | I | M | L | Y | L | L | L | N | L | D | E | V | H | E | - | Y | Q | R | Q | F | I | H | E | T | W | T | K | D | W | - | - | - | - | - | - | - | - | - | - | - | - | - | - | - | - | - | - | - | - | - | - | - | - | - | - | - | - | - | - | - | - | - | - | - | - | - | - | - | - | - | - | - | - | - | - | - | - | - | - | - | - | - | - | - | - | - | - | - | - | - | - | - | - | - | - | - | - | - | - | - | - | - | - | - | - | - | - | - | - | - | - | - | - | E | P | S | A | Q | E | E | D | H | L | L | K | N | N | - | - | - | - | - | - | - | - | - | S | P | D | F | I | S | W | F | S | T | K | V | S | S | - | - | - | - | - | - | - | - | - | - | - | - | - | - | - | - | - | - | - | - | - | - | - | - | - | - | - | - | - | - | - | - | - | - | - | - | - | - | - | - | - | - | - | - | - | - | - | - | - | - | - | - | - | - | - | - | - | - | - | - | - | - | - | - | - | - | - | - | - | - | - | - | - | - | - | - | - | - | - | - | - | - | - | - | - | - | - | - | - | - | - | - | - | - | - | - | - | - | - | - | - | - | - | - | - | - | - | - | - | - | - | - | - | - | - | - | - | - | - | - | - | - | - | - | - | - | - | - | - | - | - | N | L | P | C | L | I | F | L | H | S | T | L | X | V | - | - | - | - | - | - | - | - | - | - | - | - | - | - | - | - | - | - | - | - | - | - | - | - | - | - | - | - | - | - | - | - | - | - | - | - | - | - | - | - | - | - | - | - | - | - | - | - | - | - | - | - | - | - | - | - | - | - | - | - | - | - | - | - | - | - | - | - | - | - | - | - | - | - | - | - | - | - | - | - | - | - | - | - | - | - | - | - | - | - | - | - | - | - | - | - | - | - | - | - | - | - | - | - | - | - | - | - | - | - | - | - | - | - | - | - | - | - | - | - | - | - | - | - | - | - | - | - | - | - | - | - | - | - | - | - | - | - | - | - | - | - | - | - | - | - | - | - | - | - | - | - | - | - | - | - | - | - | - | - | - | - | - | - | - | - | - | - | - | - | - | - | - | - | - | - | - | - | - | - | - | - | - | - | - | - | - | - | - | - | - | - | - | - | - | - | - | - | - | - | - | - | - | - | - | - | - | - | - | - | - | - | - | - | - | - | - | - | - | - | - | - | - | - | - | - | - | - | - | - | - | - | - | - | - | - | - | - | - | - | - | - | - | - | - | - | - | - | - | - | - | - | - | - | - | - | - | - | - | - | - | - | - | - | - | - | - | - | - | - | - | - | - | - | - | - | - | - | - | - | - | - | - | - | - | - | - | - | - | - | - | - | - | - | - | - | - | - | - | - | - | - | - | - | - | - | - | - | - | - | - | - | - | - | - | - | - | - | - | - | - | - | - | - | - | - | - | - | - | - | - | - | - | - | - | - | - | - | - | - | - | - | - | - | - | - | - | - | - | - | - | - | - | - | - | - | - | - | - | - | - | - | - | - | - | - | - | - | - | - | - | - | - | - | - | - | - | - | - | - | - | - | - | - | - | - | - | - | - | - | - | - | - | - | - | - | - | - | - | - | - | - | - | - | - | - | - | - | - | - | - | - | - | - | - | - | - | - | - | - | - | - | - | - | - | - | - | - | - | - | - | - | - | - | - | - | - | - | - | - | - | - | - | - | - | - | - | - | - | - | - | - | - | - | - | - | - | - | - | - | - | - | - | - | - | - | - | - | - | - | - | - | - | - | - | - | - | - | - | - | - | - | - | - | - | - | - | - | - | - | - | - | - | - | - | - | - | - | - | - | - | - | - | - | - | - | - | - | - | - | - | - | - | - | - | - | - | - | - | - | - | - | - | - | - | - | - | - | - | - | - | - | - | - | - | - | - | - | - | - | - | - | - | - | - | - | - | - | - | - | - | - | - | - | - | - | - | - | - | - | - | - | - | - | - | - | - | - | - | - | - | - | - | - | - | - | - | - | - | - | - | - | - | - | - | - | - | - | - | - | - | - | - | - | - | - | - | - | - | - | - | - | - | - | - | - | - | - | - | - | - | - | - | - | - | - | - | - | - | - | - | - | - | - | - | - | - | - | - | - | - | - | - | - | - | - | - | - | - | - | - | - | - | - | - | - | - | - | - | - | - | - | - | - | - | - | - | - | - | - | - | - | - | - | - | - | - | - | - | - | - | - | - | - | - | - | - | - | - | - | - | - | - | - | - | - | - | - | - | - | - | - | - | - | - | - | - | - | - | - | - | - | - | - | - | - | - | - | - | - | - | - | - | - | - | - | - | - | - | - | - | - | - | - | - | - | - | - | - | - | - | - | - | - | - | - | - | - | - | - | - | - | - | - | - | - | - | - | - | - | - | - | - | - | - | - | - | - | - | - | - | - | - | - | - | - | - | - | - | - | - | - | - | - | - | - | - | - | - | - | - | - | - | - | - | - | - | - | - | - | - | - | - | - | - | - | - | - | - | - | - | - | - | - | - | - | - | - | - | - | - | - | - | - | - | - | - | - | - | - | - | - | - | - | - | - | - | - | - | - | - | - | - | - | - | - | - | - | - | - | - | - | - | - | - | - | - | - | - | - | - | - | - | - | - | - | - | - | - | - | - | - | - | - | - | - | - | - | - | - | - | - | - | - | - | - | - | - | - | - | - | - | - | - | - | - | - | - | - | - | - | - | - | - | - | - | - | - | - | - | - | - | - | - | - | - | - | - | - | - | - | - | - | - | - | - | - | - | - | - | - | - | - | - | - | - | - | - | - | - | - | - | - | - | - | - | - | - | - | - | - | - | - | - | - | - | - | - | - | - | - | - | - | - | - | - | - | - | - | - | - | - | - | - | - | - | - | - | - | - |
|  | Balduin | - | - | - | - | - | - | - | - | - | - | - | - | - | - | - | - | - | M | S | D | R | - | A | W | M | Y | T | G | H | P | S | - | Q | K | D | M | T | D | E | W | F | I | K | T | K | E | F | V | E | S | A | F | S | N | G | - | - | - | - | - | - | Q | E | K | T | W | C | P | C | S | R | C | E | N | - | K | K | K | T | - | E | - | - | - | - | A | V | M | G | K | H | L | Q | M | S | G | F | T | P | D | Y | T | V | - | - | - | W | T | F | H | - | G | E | S | - | A | Q | R | N | R | V | E | V | V | R | R | R | - | - | - | - | - | - | - | - | - | - | - | - | - | - | - | - | - | - | - | - | - | - | - | - | - | - | - | - | - | - | - | - | - | - | - | - | - | - | - | - | - | - | - | - | - | T | D | E | - | - | - | - | - | - | - | - | - | Y | G | T | G | I | E | D | M | V | Q | D | F | D | D | A | R | D | - | - | - | - | - | - | - | - | - | - | - | - | - | - | - | - | - | - | - | - | - | - | - | - | - | - | - | - | - | - | - | - | - | - | - | S | D | D | E | M | E | - | - | - | - | - | - | - | - | - | - | - | E | S | A | K | A | F | Y | E | M | L | E | S | S | K | R | P | L | H | - | - | - | E | H | T | - | - | E | L | C | Q | L | D | A | I | A | Q | V | M | A | L | K | A | Q | F | N | L | G | - | - | - | - | - | R | E | C | Y | D | A | M | M | T | L | F | - | G | R | F | L | P | K | G | H | V | M | P | A | N | L | Y | Q | S | D | K | I | L | R | A | L | K | M | P | Y | E | K | I | H | A | C | E | K | G | C | A | L | F | R | N | E | - | Y | A | D | L | N | Y | C | P | I | C | K | S | S | R | Y | V | V | V | D | N | G | M | G | - | - | - | - | - | - | - | - | - | - | - | - | - | - | - | - | - | - | - | - | - | - | - | - | - | - | - | - | - | - | - | - | - | - | - | - | - | E | K | R | Q | T | K | I | P | V | S | V | L | R | Y | M | P | I | V | P | R | L | Q | R | L | F | M | V | E | E | T | A | R | Q | M | T | W | H | K | F | G | - | - | - | - | - | - | - | - | - | - | K | R | - | - | T | E | L | D | A | D | G | N | L | M | M | V | H | T | S | D | G | D | A | W | K | D | F | D | G | L | H | E | D | - | K | A | A | D | P | R | N | P | R | V | G | I | S | T | D | G | F | N | P | F | G | M | T | A | T | Q | Y | S | C | W | P | V | F | V | I | P | L | N | L | - | P | P | G | Q | I | M | Q | R | K | N | I | F | L | T | L | I | I | P | G | P | N | Y | P | G | K | N | M | - | N | V | Y | M | Q | P | L | K | D | E | L | E | E | A | W | D | - | N | G | V | K | T | Y | D | A | A | R | K | E | N | F | K | M | H | V | W | Y | M | Y | S | M | H | D | L | P | A | Y | A | L | F | V | G | W | C | V | H | G | R | F | P | - | - | C | P | Q | C | K | A | A | L | E | F | H | W | L | Q | A | G | R | K | Y | S | C | F | D | L | H | R | Q | F | L | D | P | D | H | Q | F | R | - | - | K | D | K | K | N | F | I | K | G | K | V | V | K | N | S | - | A | P | P | A | L | T | - | G | Q | Q | I | L | - | D | Q | L | N | A | L | E | P | D | P | E | - | - | - | - | - | - | - | - | - | - | - | - | - | - | - | - | - | - | - | - | - | - | - | - | - | - | - | - | - | - | - | - | - | - | - | - | - | - | - | - | - | - | - | - | R | P | G | Y | F | K | G | Y | - | N | S | K | H | A | W | T | H | K | P | C | F | W | D | - | L | P | Y | F | K | - | - | D | L | L | L | P | H | N | I | D | M | M | H | T | E | K | N | I | A | E | A | L | F | G | T | L | F | G | I | D | G | K | S | K | D | N | T | K | A | R | V | D | Q | E | T | L | C | D | R | P | L | Q | N | M | R | E | P | K | G | - | - | - | - | - | - | - | - | - | K | Q | N | W | S | K | P | K | A | W | F | N | L | G | R | P | A | M | R | E | I | L | L | W | V | Q | K | Q | L | M | F | P | D | G | Y | A | A | N | L | K | R | G | A | S | L | E | K | L | K | I | F | G | - | L | K | S | H | D | W | H | I | W | L | E | R | V | M | P | V | M | L | R | G | F | - | - | - | - | - | - | - | I | P | E | - | D | E | W | L | V | L | A | - | - | - | - | - | - | - | - | - | - | - | - | - | - | - | - | - | - | - | - | - | - | - | E | L | S | Y | F | F | R | V | L | C | A | K | E | L | S | P | G | V | V | E | D | M | E | E | L | A | P | E | L | F | C | K | L | E | K | I | F | P | P | G | - | - | - | - | F | F | N | P | M | Q | H | L | I | L | H | L | P | T | E | A | R | L | G | G | P | V | Q | N | R | W | C | Y | A | T | E | - | - | - | - | - | - | - | - | - | - | - | - | - | - | - | - | - | - | - | - | - | - | - | - | - | - | - | - | - | R | M | - | - | - | - | - | - | - | - | - | - | - | - | - | - | - | - | - | - | - | - | - | - | - | - | - | - | - | - | - | - | - | - | - | - | - | - | - | - | - | - | - | - | - | - | - | - | - | - | - | - | - | - | - | - | - | - | - | - | - | - | - | - | - | - | - | - | - | - | - | - | - | - | - | - | Q | K | T | L | R | G | K | C | K | N | K | R | R | I | E | A | S | M | A | E | A | F | I | T | E | E | A | A | N | - | - | - | - | F | V | T | A | H | Y | E | A | K | N | H | - | - | H | L | H | N | P | K | P | R | Y | N | V | G | D | P | K | K | G | - | - | - | - | G | S | N | L | S | L | F | K | G | K | L | A | P | A | G | V | S | Q | P | I | S | L | D | - | - | - | - | I | E | E | W | R | T | I | S | L | Y | I | F | N | N | L | I | E | V | R | P | - | Y | I | D | R | Y | V | V | K | F | S | Y | G | A | V | I | - | - | - | - | - | - | - | - | - | - | - | - | - | - | - | - | - | - | - | - | - | - | - | - | - | - | - | - | - | - | - | - | - | - | - | - | - | - | - | - | - | - | - | - | - | - | - | - | - | - | - | - | - | - | - | - | - | - | - | - | - | - | - | - | - | - | - | - | - | - | - | - | - | - | - | - | - | - | - | - | - | - | - | E | N | D | S | V | E | E | Y | E | L | L | A | K | T | - | - | - | - | - | - | G | G | G | Y | P | G | F | I | S | W | F | K | - | - | - | - | - | - | - | - | - | - | - | - | - | - | - | - | - | - | - | - | - | - | - | - | - | - | - | - | - | - | - | - | - | - | - | - | - | - | - | Q | T | S | M | D | A | E | L | R | Q | V | A | N | G | F | D | Y | K | V | R | S | F | E | K | Y | D | I | N | G | Y | R | F | R | T | F | G | K | E | L | S | - | M | P | D | R | K | S | K | N | F | G | V | S | A | I | G | E | G | G | - | - | - | - | - | - | - | - | - | - | - | - | - | - | I | E | Y | Y | G | R | V | E | A | I | Y | E | L | H | F | - | - | - | - | - | Y | G | E | N | P | P | N | V | V | V | F | K | - | C | Y | W | F | E | - | - | - | - | - | - | - | - | - | - | - | - | P | N | K | T | - | - | R | R | T | H | E | H | V | G | L | V | E | - | - | - | - | - | - | - | - | - | - | - | I | N | Q | - | - | - | - | - | N | T | H | L | - | - | V | V | P | D | V | Y | I | M | A | Q | Q | A | T | Q | V | F | Y | L | P | W | A | - | - | C | Q | T | D | E | N | L | - | E | G | - | - | - | - | - | - | - | - | - | - | W | Y | V | V | Y | E | V | P | P | H | V | R | P | P | P | - | P | - | - | - | N | E | E | D | Y | E | P | H | - | - | - | I | N | P | D | T | Y | D | G | E | F | F | Q | E | - | S | R | L | S | K | N | R | F | K | N | R | Y | T | S | S | Q | N | M | E | V | D | S | D | N | E | S | Y | F | I | P | E | A | E | Q | E | E | L | E | L | E | E | - | - | - | - | - | - | - | - | - | - | - | - | - | - | - | - | - | - | - | - | - | - | - | - | - | - | - | - | - | - | - | - | - | - | - | - | - | - | - | - | - | - | - | - | - | - | - | - | - | - | - | - | - | - | - | - | - | - | - | - | - | - | - | - | - | - | - | - | - | - | - | - | - | - | - | - | - | - | - | - | - | - | - | - | - | - | - | - | - | - | - | - | - | - | - | - | - | - | - | - | - | - | - | - | - | - | - | - | - | - | - | - | - | - | - | - | - | - | - | - | - | - | - | - | - | - | - | - | - | - | - | - | - | - | - | - | - | - | - | - | - | - | - | - | - | - | - | - | - | - | - | - | - | - | - | - | - | - | - | - | - | - | - | - | - | - | - | - | - | - | - | - | - | - | - | - | - | - | - | - | - | - | - | - | - | - | - | - | - | - | - | - | - | - | - | - | - | - | - | - | - | - | - | - | - | - | - | - | - | - | - | - | - | - | - | - | - | - | - | - | - | - | - | - | - | - | - | - | - | - | - | - | - | - | - | - | - | - | - | - | - | - | - | - | - | - | - | - | - | - | - | - | - | - | - | - | - | - | - | - | - | - | - | - | - | - | - | - | - | - | - | - | - | - | - | - | - | - | - | - | - | - | - | - | - | - | - | - | - | - | - | - | - | - | - | - | - | - | - | - | - | - | - | - | - | - | - | - | - | - | - | - | - | - | - | - | - | - | - | - | - | - | - | - | - | - | - | - | - | - | - | - | - | - | - | - | - | - | - | - | - | - | - | - | - | - | - | - | - | - | - | - | - | - | - | - | - | - | - | - | - | - | - | - | - | - | - | - | - | - | - | - | - | - | - | - | - | - | - | - | - | - | - | - | - | - | - | - | - | - | - | - | - | - | - | - | - | - | - | - | - | - | - | - | - | - | - | - | - | - | - | - | - | - | - | - | - | - | - | - | - | - | - | - | - | - | - | - | - | - | - | - | - | - | - | - | - | - | - | - | - | - | - | - | - | - | - | - | - | - | - | - | - | - | - | - | - | - | - | - | - | - | - | - | - | - | - | - | - | - | - | - | - | - | - | - | - | - | - | - | - | - | - | - | - | - | - | - | - | - | - | - | - | - | - | - | - | - | V | T | D | A | D | D | L | S | M | L | D | R | L | Q | E | G | L | S | H | V | D | A | V | E | P | D | E | P | V | I | H | Y | D | M | C | D | S | D | D | D | Y | A | F | I | D | D | T | Y | R | D | Y | - | - | - | - | - | - | - | - | - | - | - | - | - | - | - | - | - | - | - | - | - | - | - | - | - | - | - | - | - | - | - | - | - | - | - | - | - | - | - | - | - | - | - | - | - | - | - | - | - | - | - | - | - | - | - | - | - | - | - | - | - | - | - | - | - | - | - | - | - | - | - | - | - | - | - | - | - | - | - | - | - | - | - | - | - | - | - | - | - | - | - | - | - | - | - | - | - | - | - | - | - | - | - | - | - | - | - | - | - | - | - | - | - | - | - | - | - | - | - | - | - | - | - | - | - | - | - | - | - | - | - | - | - | - | - | - | - | - | - | - | - | - | - | - | - | - | - | - | - | - | - | - | - | - | - | - | - | - | - | - | - | - | - | - | - | - | - | - | - | - | - | - | - | - | - | - | - | - | - | - | - | - | - | - | - | - | - | - | - | - | - | - | - | - | - | - | - | - | - | - | - | - | - | - | - | - | - | - | - | - | - | - | - | - | - | - | - | - | - | - | - | - | - | - | - | - | - | - | - | - | - | - | - | - | - | - | - | - |
|  | G | - | - | - | - | - | - | - | - | - | - | - | - | - | - | - | - | - | M | A | D | R | - | R | W | M | Y | S | G | R | R | S | - | R | T | D | R | D | D | D | W | M | K | N | T | A | D | F | L | E | V | A | F | A | R | G | - | - | - | - | - | - | E | E | R | A | W | C | P | C | S | R | C | N | N | K | T | R | H | T | - | K | - | - | - | - | D | E | M | G | N | H | L | L | L | Y | G | Y | T | P | N | Y | T | R | - | - | - | W | G | Y | H | - | G | E | Q | - | E | D | R | G | R | E | E | V | V | R | R | R | - | - | - | - | - | - | - | - | - | - | - | - | - | - | - | - | - | - | - | - | - | - | - | - | - | - | - | - | - | - | - | - | - | - | - | - | - | - | - | - | - | - | - | - | - | T | D | D | - | - | - | - | - | - | - | - | - | Y | D | T | G | F | Q | D | M | L | D | D | L | E | E | A | R | V | P | - | - | - | - | - | - | - | - | - | - | - | - | - | - | - | - | - | - | - | - | - | - | - | - | - | - | - | - | - | - | - | - | - | - | T | E | E | E | P | E | - | - | - | - | - | - | - | - | - | - | - | A | T | S | K | A | Y | L | D | M | L | A | E | A | S | T | D | L | H | - | - | - | A | H | T | - | - | K | V | N | K | L | D | A | V | A | R | I | M | A | V | K | T | Q | F | N | L | S | - | - | - | - | - | R | E | W | F | D | V | M | M | A | V | I | - | G | S | L | L | P | E | G | H | L | L | P | K | S | M | Y | E | A | N | K | M | L | S | V | L | K | L | P | A | Q | C | L | H | S | C | K | N | G | C | T | V | F | D | R | E | - | Y | A | D | A | K | H | C | P | K | C | N | F | S | R | Y | V | E | V | D | H | G | K | G | - | - | - | - | - | - | - | - | - | - | - | - | - | - | - | - | - | - | - | - | - | - | - | - | - | - | - | - | - | - | - | - | - | - | - | - | - | - | L | V | Q | S | K | I | P | H | K | V | L | R | M | L | P | F | S | E | R | I | G | R | M | M | L | S | D | V | P | G | K | A | V | T | H | H | K | L | G | - | - | - | - | - | - | - | - | - | - | K | K | - | - | Y | K | Y | D | K | D | H | R | P | M | L | T | H | P | S | D | G | E | A | W | K | D | F | D | R | K | Y | P | E | - | K | A | A | E | P | R | N | V | R | V | A | I | A | T | D | G | F | N | P | F | G | M | T | S | A | P | Y | S | C | W | P | V | F | V | I | P | L | N | L | - | P | P | G | M | I | M | Q | R | K | Y | M | F | L | A | L | I | I | P | G | P | E | Y | P | G | K | N | I | - | N | V | Y | M | R | P | L | M | E | E | L | K | R | G | W | E | - | E | G | I | M | V | Y | D | K | A | T | K | T | N | F | R | M | H | I | W | F | Q | F | S | I | H | D | H | P | G | G | S | I | L | S | G | K | G | L | Q | G | K | F | M | - | - | C | P | H | C | G | T | D | L | E | F | Y | R | L | A | H | G | R | K | Y | C | C | F | D | L | H | R | R | F | L | P | P | N | H | P | F | R | - | - | Q | D | K | K | N | F | R | K | G | V | V | V | E | E | G | - | P | P | R | Y | R | T | - | G | S | E | L | L | - | A | E | L | D | A | L | V | P | D | P | T | - | - | - | - | - | - | - | - | - | - | - | - | - | - | - | - | - | - | - | - | - | - | - | - | - | - | - | - | - | - | - | - | - | - | - | - | - | - | - | - | - | - | - | - | Q | P | D | K | F | L | G | Y | - | G | D | T | H | N | W | T | H | K | S | C | F | W | E | - | L | P | Y | F | K | - | - | D | I | I | L | P | H | C | I | D | V | M | H | T | E | K | N | V | A | E | A | L | F | N | T | I | L | N | I | I | D | K | T | K | D | N | P | K | A | R | K | D | Q | E | E | L | C | D | R | P | Q | L | S | I | K | K | V | P | - | - | - | - | - | - | - | - | - | - | G | R | S | D | I | R | P | K | A | P | F | A | L | D | R | D | Q | K | M | E | V | L | L | W | F | - | K | E | L | K | F | P | D | G | Y | A | A | N | L | K | R | G | V | N | L | G | T | M | R | I | N | G | - | L | K | S | H | D | Y | H | I | W | L | E | R | L | L | P | V | M | V | R | G | Y | - | - | - | - | - | - | - | V | S | E | - | D | I | W | R | V | L | A | - | - | - | - | - | - | - | - | - | - | - | - | - | - | - | - | - | - | - | - | - | - | - | E | L | S | N | F | F | R | R | L | C | A | K | E | L | S | P | T | L | L | E | E | M | E | K | Q | A | P | V | L | L | C | K | L | E | R | I | F | P | P | G | - | - | - | - | M | F | V | T | M | Q | H | L | I | I | H | L | P | Y | E | A | R | M | W | G | P | V | Q | H | R | W | M | Y | A | V | E | - | - | - | - | - | - | - | - | - | - | - | - | - | - | - | - | - | - | - | - | - | - | - | - | - | - | - | - | - | R | L | - | - | - | - | - | - | - | - | - | - | - | - | - | - | - | - | - | - | - | - | - | - | - | - | - | - | - | - | - | - | - | - | - | - | - | - | - | - | - | - | - | - | - | - | - | - | - | - | - | - | - | - | - | - | - | - | - | - | - | - | - | - | - | - | - | - | - | - | - | - | - | - | - | - | M | K | T | L | R | A | K | V | K | N | K | A | K | V | E | A | C | I | W | E | A | S | R | L | E | E | V | S | N | - | - | - | - | A | T | T | S | Y | Y | S | S | D | I | A | - | - | T | L | H | N | P | V | S | R | Y | N | L | G | N | P | E | D | E | - | - | - | - | - | S | K | L | S | L | F | R | G | Q | L | G | T | S | K | A | E | K | T | V | T | L | S | - | - | - | - | Y | Q | E | W | R | S | L | T | L | Y | I | L | N | N | L | D | E | V | Q | P | - | Y | I | K | A | - | - | - | - | - | - | - | - | - | - | - | - | - | - | - | - | - | - | - | - | - | - | - | - | - | - | - | - | - | - | - | - | - | - | - | - | - | - | - | - | - | - | - | - | - | - | - | - | - | - | - | - | - | - | - | - | - | - | - | - | - | - | - | - | - | - | - | - | - | - | - | - | - | - | - | - | - | - | - | - | - | - | - | - | - | - | - | - | - | - | - | - | - | - | - | - | - | - | - | - | - | - | - | - | - | - | - | - | - | - | - | - | - | - | - | - | - | - | - | - | - | - | - | - | - | - | - | - | - | - | - | - | - | - | - | - | - | - | - | - | - | - | - | - | - | - | - | - | - | - | - | - | - | - | - | - | - | - | - | - | - | - | - | N | N | D | A | S | M | S | S | Q | L | R | Q | I | A | N | G | F | D | V | K | V | G | S | F | K | S | Y | D | V | N | G | Y | R | F | H | T | T | G | Y | E | L | S | - | R | P | N | R | T | S | T | N | S | G | V | F | V | E | C | G | G | D | - | - | - | - | - | - | - | - | - | - | - | - | - | - | - | D | F | Y | G | R | V | E | E | I | Y | E | L | Q | Y | - | - | - | - | - | R | G | P | K | P | P | K | V | V | V | F | K | - | C | R | W | I | D | - | - | - | - | - | - | - | - | - | - | - | - | P | K | H | V | - | - | N | K | D | D | T | I | - | G | L | V | E | - | - | - | - | - | - | - | - | - | - | - | V | K | R | - | - | - | - | - | D | S | V | F | - | - | E | C | E | D | V | Y | I | V | A | Q | Q | A | T | Q | V | Y | F | L | P | Y | P | - | - | C | T | K | V | A | R | L | - | Q | G | - | - | - | - | - | - | - | - | - | - | L | D | V | V | Y | H | V | Q | P | H | G | K | L | P | E | - | P | - | - | - | N | D | Q | D | Y | L | P | Q | - | - | - | - | - | - | R | E | P | G | D | E | F | F | Q | E | - | A | G | G | D | T | S | D | F | V | V | R | Y | T | D | S | P | G | T | E | V | D | N | D | G | E | G | D | E | V | - | - | - | - | - | - | - | - | - | - | - | - | - | - | - | - | - | - | - | - | - | - | - | - | - | - | - | - | - | - | - | - | - | - | - | - | - | - | - | - | - | - | - | - | - | - | - | - | - | - | - | - | - | - | - | - | - | - | - | - | - | - | - | - | - | - | - | - | - | - | - | - | - | - | - | - | - | - | - | - | - | - | - | - | - | - | - | - | - | - | - | - | - | - | - | - | - | - | - | - | - | - | - | - | - | - | - | - | - | - | - | - | - | - | - | - | - | - | - | - | - | - | - | - | - | - | - | - | - | - | - | - | - | - | - | - | - | - | - | - | - | - | - | - | - | - | - | - | - | - | - | - | - | - | - | - | - | - | - | - | - | - | - | - | - | - | - | - | - | - | - | - | - | - | - | - | - | - | - | - | - | - | - | - | - | - | - | - | - | - | - | - | - | - | - | - | - | - | - | - | - | - | - | - | - | - | - | - | - | - | - | - | - | - | - | - | - | - | - | - | - | - | - | - | - | - | - | - | - | - | - | - | - | - | - | - | - | - | - | - | - | - | - | - | - | - | - | - | - | - | - | - | - | - | - | - | - | - | - | - | - | - | - | - | - | - | - | - | - | - | - | - | - | - | - | - | - | - | - | - | - | - | - | - | - | - | - | - | - | - | - | - | - | - | - | - | - | - | - | - | - | - | - | - | - | - | - | - | - | - | - | - | - | - | - | - | - | - | - | - | - | - | - | - | - | - | - | - | - | - | - | - | - | - | - | - | - | - | - | - | - | - | - | - | - | - | - | - | - | - | - | - | - | - | - | - | - | - | - | - | - | - | - | - | - | - | - | - | - | - | - | - | - | - | - | - | - | - | - | - | - | - | - | - | - | - | - | - | - | - | - | - | - | - | - | - | - | - | - | - | - | - | - | - | - | - | - | - | - | - | - | - | - | - | - | - | - | - | - | - | - | - | - | - | - | - | - | - | - | - | - | - | - | - | - | - | - | - | - | - | - | - | - | - | - | - | - | - | - | - | - | - | - | - | - | - | - | - | - | - | - | - | - | - | - | - | - | - | - | - | - | - | - | - | - | - | - | - | - | - | - | - | - | - | - | - | - | - | - | - | - | - | - | - | - | - | - | - | - | - | - | - | - | - | - | - | - | - | - | - | - | - | - | - | - | - | - | - | - | - | - | - | - | - | - | - | D | G | D | G | D | D | D | D | E | E | Q | A | N | V | D | S | D | V | E | S | D | L | D | A | A | L | L | V | D | P | D | D | E | D | Y | N | - | - | - | - | - | - | - | - | - | - | - | - | - | - | - | - | - | - | - | - | - | - | - | - | - | - | - | - | - | - | - | - | - | - | - | - | - | - | - | - | - | - | - | - | - | - | - | - | - | - | - | - | - | - | - | - | - | - | - | - | - | - | - | - | - | - | - | - | - | - | - | - | - | - | - | - | - | - | - | - | - | - | - | - | - | - | - | - | - | - | - | - | - | - | - | - | - | - | - | - | - | - | - | - | - | - | - | - | - | - | - | - | - | - | - | - | - | - | - | - | - | - | - | - | - | - | - | - | - | - | - | - | - | - | - | - | - | - | - | - | - | - | - | - | - | - | - | - | - | - | - | - | - | - | - | - | - | - | - | - | - | - | - | - | - | - | - | - | - | - | - | - | - | - | - | - | - | - | - | - | - | - | - | - | - | - | - | - | - | - | - | - | - | - | - | - | - | - | - | - | - | - | - | - | - | - | - | - | - | - | - | - | - | - | - | - | - | - | - | - | - | - | - | - | - | P | H | A | H | T | - | - | - | - | - | - | - |
|  | Isidor | - | - | - | - | - | - | - | - | - | - | - | - | - | - | - | - | M | E | E | D | R | - | S | W | M | Y | N | G | R | R | K | - | R | N | Q | V | S | A | D | W | I | L | K | T | T | D | F | L | D | R | A | F | S | R | D | - | - | - | T | S | - | A | D | G | V | M | C | P | F | V | T | C | E | H | T | K | T | Q | A | - | H | T | K | Q | W | D | T | M | M | K | H | L | F | K | H | G | F | R | P | E | Y | T | V | - | - | - | W | V | Y | H | - | G | E | S | D | S | D | P | T | R | D | D | V | L | R | Q | R | - | - | - | - | - | - | - | - | - | - | - | - | - | - | - | - | - | - | - | - | - | - | - | - | - | - | - | - | - | - | - | - | - | - | - | - | - | - | - | - | - | - | - | - | - | T | F | D | - | - | - | - | E | D | G | D | A | E | V | H | R | M | D | D | M | V | D | D | V | R | D | A | H | I | S | V | - | - | - | - | - | - | - | - | - | - | - | - | - | - | - | - | - | - | - | - | - | - | - | - | - | - | - | - | - | - | - | - | - | E | E | E | E | P | E | - | - | - | - | - | - | - | - | - | - | - | P | I | A | R | A | F | Y | E | M | L | T | A | S | N | Q | S | L | H | - | - | - | A | H | T | - | - | E | V | S | Q | L | D | A | I | T | R | L | L | A | V | K | S | Q | F | S | I | S | - | - | - | - | - | I | A | G | F | D | A | L | L | N | V | F | - | G | A | L | L | P | Q | G | H | K | L | P | L | N | L | Y | E | A | K | K | F | L | S | A | L | T | M | P | Y | E | K | I | D | V | C | P | K | Y | C | M | L | F | R | E | E | - | N | S | E | K | T | H | C | D | K | C | G | E | S | R | Y | V | E | V | Q | N | S | Y | G | - | - | - | - | - | - | - | - | - | - | - | - | - | - | - | - | - | - | - | - | - | - | - | - | - | - | - | - | - | - | - | - | - | - | - | - | - | E | K | K | Q | L | N | I | P | M | K | V | L | R | Y | I | P | F | I | P | R | L | Q | R | L | Y | M | S | E | P | Q | A | K | Q | M | T | W | H | K | Y | G | - | - | - | - | - | - | - | - | - | - | H | R | - | - | Y | H | P | N | K | - | - | - | - | - | I | V | H | T | A | D | A | E | A | W | K | Q | F | D | R | D | F | S | E | - | F | A | S | D | A | R | N | V | R | I | A | I | A | T | D | G | F | N | P | F | G | M | G | A | A | S | Y | T | C | W | P | V | F | V | I | P | L | N | L | - | P | P | G | V | C | M | Q | K | H | N | M | F | L | S | L | I | I | P | G | P | D | Y | P | G | K | K | I | - | S | M | Y | M | E | P | L | V | D | D | L | L | H | A | W | E | - | H | G | V | Q | T | Y | D | R | A | T | K | Q | N | F | N | M | R | V | S | Y | L | F | S | F | H | D | L | P | A | Y | G | I | F | C | G | W | C | V | H | G | K | M | P | - | - | C | P | V | C | M | E | V | L | K | G | R | R | L | K | F | G | G | K | Y | S | F | F | D | C | H | R | Q | F | L | P | H | G | H | I | F | R | - | - | N | D | P | N | S | F | L | A | N | T | T | V | T | T | E | - | P | P | H | R | F | K | - | T | E | E | V | H | - | V | R | L | Q | R | L | Q | P | A | A | - | - | - | - | - | - | - | - | - | - | - | - | - | - | - | - | - | - | - | - | - | - | - | - | - | - | - | - | - | - | - | - | - | - | - | - | - | - | - | - | - | - | - | - | - | N | G | E | G | F | E | G | Y | - | G | E | D | H | N | W | T | H | I | P | G | L | W | R | - | L | P | Y | F | H | - | - | K | L | V | L | P | H | N | I | D | V | M | H | N | E | K | N | V | A | E | A | I | F | N | T | C | F | D | I | P | E | K | T | K | D | N | V | K | A | R | L | D | Q | A | I | L | C | N | R | P | D | L | N | M | I | R | R | Q | T | - | - | - | - | - | - | - | - | - | S | G | Q | W | L | K | P | R | A | D | F | C | L | N | R | T | Q | K | K | E | I | L | E | W | F | - | Q | T | L | K | F | P | D | G | Y | G | S | N | L | R | R | G | V | N | F | K | K | M | R | I | N | G | - | L | K | S | H | D | Y | H | I | M | M | E | R | L | L | P | V | M | F | R | G | Y | - | - | - | - | - | - | - | F | Q | P | - | Y | L | W | E | V | I | A | - | - | - | - | - | - | - | - | - | - | - | - | - | - | - | - | - | - | - | - | - | - | - | E | L | S | F | F | Y | R | K | L | C | A | K | E | V | D | P | I | E | L | E | S | M | E | L | Q | V | P | V | L | L | C | K | L | E | M | I | F | P | S | G | - | - | - | - | F | F | N | P | M | Q | H | L | I | L | H | L | P | Y | E | A | R | M | G | G | P | V | Q | Y | R | W | M | Y | P | G | E | - | - | - | - | - | - | - | - | - | - | - | - | - | - | - | - | - | - | - | - | - | - | - | - | - | - | - | - | - | R | D | - | - | - | - | - | - | - | - | - | - | - | - | - | - | - | - | - | - | - | - | - | - | - | - | - | - | - | - | - | - | - | - | - | - | - | - | - | - | - | - | - | - | - | - | - | - | - | - | - | - | - | - | - | - | - | - | - | - | - | - | - | - | - | - | - | - | - | - | - | - | - | - | - | - | Q | K | D | L | K | S | K | V | K | N | R | A | R | V | E | A | S | I | A | E | A | Y | I | L | D | E | I | A | N | - | - | - | - | F | T | T | I | Y | F | A | D | Q | V | Y | - | - | T | V | H | N | P | V | P | R | Y | N | V | T | V | E | S | R | E | - | - | - | - | - | C | S | L | S | L | F | S | I | K | G | D | S | T | S | R | G | V | T | R | H | L | T | - | - | - | - | E | V | E | W | E | A | A | M | L | Y | V | L | T | N | L | P | E | V | D | D | - | Y | V | G | K | F | L | H | E | E | W | S | R | R | G | - | - | - | - | - | - | - | - | - | - | - | - | - | - | - | - | - | - | - | - | - | - | - | - | - | - | - | - | - | - | - | - | - | - | - | - | - | - | - | - | - | - | - | - | - | - | - | - | - | - | - | - | - | - | - | - | - | - | - | - | - | - | - | - | - | - | - | - | - | - | - | - | - | - | - | - | - | - | - | - | - | - | - | - | E | P | T | R | Q | Q | K | E | N | L | L | R | N | G | - | - | - | - | - | A | R | N | G | C | P | N | F | V | T | W | F | L | - | - | - | - | - | - | - | - | - | - | - | - | - | - | - | - | - | - | - | - | - | - | - | - | - | - | - | - | - | - | - | - | - | - | - | - | - | - | - | D | M | N | M | N | D | D | L | K | Q | V | A | K | G | C | H | T | R | V | K | T | Y | S | I | Y | D | V | N | G | Y | R | F | R | T | N | K | Y | E | K | E | - | R | P | N | A | T | T | I | I | V | G | L | V | T | I | G | Q | G | E | N | S | D | I | - | - | - | - | - | - | - | - | - | - | T | D | Y | Y | G | Y | I | K | E | I | I | E | L | S | F | - | - | - | - | - | H | G | D | S | E | L | T | L | V | L | F | N | - | C | H | W | F | D | - | - | - | - | - | - | - | - | - | - | - | - | P | A | Q | T | - | - | R | Y | T | P | Q | Y | - | G | L | V | E | - | - | - | - | - | - | - | - | - | - | - | V | A | H | - | - | - | - | - | S | S | T | L | - | - | A | V | Y | E | P | F | V | V | A | H | Q | A | T | Q | V | Y | Y | I | P | Y | P | - | - | C | K | S | V | P | A | L | - | I | D | - | - | - | - | - | - | - | - | - | - | W | W | V | V | Y | K | V | Q | P | T | G | K | I | A | A | - | P | - | - | - | V | D | K | D | Y | D | - | - | - | - | - | F | L | P | N | N | D | N | V | H | Y | F | Q | E | - | D | G | L | - | - | - | - | - | Q | G | T | F | V | V | D | L | M | E | G | L | H | N | V | S | E | - | - | A | T | T | R | D | A | E | G | - | - | - | - | - | - | - | - | - | - | - | - | - | - | - | - | - | - | - | - | - | - | - | - | - | - | - | - | - | - | - | - | - | - | - | - | - | - | - | - | - | - | - | - | - | - | - | - | - | - | - | - | - | - | - | - | - | - | - | - | - | - | - | - | - | - | - | - | - | - | - | - | - | - | - | - | - | - | - | - | - | - | - | - | - | - | - | - | - | - | - | - | - | - | - | - | - | - | - | - | - | - | - | - | - | - | - | - | - | - | - | - | - | - | - | - | - | - | - | - | - | - | - | - | - | - | - | - | - | - | - | - | - | - | - | - | - | - | - | - | - | - | - | - | - | - | - | - | - | - | - | - | - | - | - | - | - | - | - | - | - | - | - | - | - | - | - | - | - | - | - | - | - | - | - | - | - | - | - | - | - | - | - | - | - | - | - | - | - | - | - | - | - | - | - | - | - | - | - | - | - | - | - | - | - | - | - | - | - | - | - | - | - | - | - | - | - | - | - | - | - | - | - | - | - | - | - | - | - | - | - | - | - | - | - | - | - | - | - | - | - | - | - | - | - | - | - | - | - | - | - | - | - | - | - | - | - | - | - | - | - | - | - | - | - | - | - | - | - | - | - | - | - | - | - | - | - | - | - | - | - | - | - | - | - | - | - | - | - | - | - | - | - | - | - | - | - | - | - | - | - | - | - | - | - | - | - | - | - | - | - | - | - | - | - | - | - | - | - | - | - | - | - | - | - | - | - | - | - | - | - | - | - | - | - | - | - | - | - | - | - | - | - | - | - | - | - | - | - | - | - | - | - | - | - | - | - | - | - | - | - | - | - | - | - | - | - | - | - | - | - | - | - | - | - | - | - | - | - | - | - | - | - | - | - | - | - | - | - | - | - | - | - | - | - | - | - | - | - | - | - | - | - | - | - | - | - | - | - | - | - | - | - | - | - | - | - | - | - | - | - | - | - | - | - | - | - | - | - | - | - | - | - | - | - | - | - | - | - | - | - | - | - | - | - | - | - | - | - | - | - | - | - | - | - | - | - | - | - | - | - | - | - | - | - | - | - | - | - | - | - | - | - | - | - | - | - | - | - | - | - | - | - | - | - | - | - | - | - | - | - | - | - | - | - | - | - | - | - | - | - | - | - | - | I | V | N | D | K | D | L | Q | L | L | N | G | S | N | V | L | Q | Q | D | E | S | D | S | S | E | F | D | E | - | - | - | - | - | - | - | - | D | E | E | E | G | P | - | - | - | - | - | I | I | D | E | Y | - | - | - | - | - | - | - | - | - | - | - | - | - | - | - | - | - | - | - | - | - | - | - | - | - | - | - | - | - | - | - | - | - | - | - | - | - | - | - | - | - | - | - | - | - | - | - | - | - | - | - | - | - | - | - | - | - | - | - | - | - | - | - | - | - | - | - | - | - | - | - | - | - | - | - | - | - | - | - | - | - | - | - | - | - | - | - | - | - | - | - | - | - | - | - | - | - | - | - | - | - | - | - | - | - | - | - | - | - | - | - | - | - | - | - | - | - | - | - | - | - | - | - | - | - | - | - | - | - | - | - | - | - | - | - | - | - | - | - | - | - | - | - | - | - | - | - | - | - | - | - | - | - | - | - | - | - | - | - | - | - | - | - | - | - | - | - | - | - | - | - | - | - | - | - | - | - | - | - | - | - | - | - | - | - | - | - | - | - | - | - | - | - | - | - | - | - | - | - | - | - | - | - | - | - | - | - | - | - | - | - | - | - | - | - | - | - | - | - | - | - | - | - | - | - | F | - | - | - | - | - | - | - | - | - | - | - |
|  | Radon | - | - | - | - | - | - | - | - | - | - | - | - | - | - | - | - | M | L | V | D | R | - | N | W | M | Y | K | G | H | R | N | - | R | G | E | V | T | A | E | W | I | L | K | T | T | E | F | L | D | M | A | F | S | R | D | - | - | - | T | R | - | C | A | D | V | L | C | P | C | N | Y | C | N | N | T | R | P | Q | T | - | R | - | - | - | - | D | V | M | M | K | H | L | C | K | Y | G | F | R | P | E | Y | T | V | - | - | - | W | V | Y | H | - | G | E | - | - | T | H | P | T | C | E | E | V | V | C | E | S | - | - | - | - | - | - | - | - | - | - | - | - | - | - | - | - | - | - | - | - | - | - | - | - | - | - | - | - | - | - | - | - | - | - | - | - | - | - | - | - | - | - | - | - | - | T | E | D | - | - | - | - | E | D | R | V | V | D | I | N | R | M | D | D | M | V | D | D | I | R | D | A | Y | V | S | V | - | - | - | - | - | - | - | - | - | - | - | - | - | - | - | - | - | - | - | - | - | - | - | - | - | - | - | - | - | - | - | - | - | E | E | E | D | P | E | - | - | - | - | - | - | - | - | - | - | - | P | T | A | Q | S | F | F | E | M | L | S | A | A | T | K | P | L | H | - | - | - | E | H | T | - | - | Q | V | S | Q | L | D | A | T | T | R | L | L | A | V | K | S | Q | Y | A | I | S | - | - | - | - | - | I | A | G | F | D | A | L | L | N | V | V | - | C | A | L | L | P | Q | G | H | K | L | P | P | N | L | Y | E | A | K | K | F | L | S | A | L | N | M | P | Y | E | K | I | D | A | C | P | K | N | C | M | L | F | R | K | E | - | N | E | H | K | T | H | C | D | K | C | G | E | S | R | Y | V | E | V | E | N | S | K | G | - | - | - | - | - | - | - | - | - | - | - | - | - | - | - | - | - | - | - | - | - | - | - | - | - | - | - | - | - | - | - | - | - | - | - | - | - | Q | K | R | Q | L | T | V | A | R | K | V | L | R | Y | L | P | I | I | P | R | L | Q | R | L | Y | M | S | E | S | Q | A | K | Q | M | T | W | H | K | T | G | - | - | - | - | - | - | - | - | - | - | H | R | - | - | Y | H | P | D | K | - | - | - | - | - | I | V | H | P | A | D | G | E | A | W | K | K | F | D | R | D | F | P | A | - | F | A | M | E | M | R | N | V | R | V | A | I | A | T | D | G | F | C | P | F | G | M | G | A | S | S | Y | T | C | W | P | M | F | V | I | P | L | N | L | - | P | P | G | I | C | M | R | Q | H | N | M | F | L | S | L | I | I | P | G | P | N | Y | P | G | K | D | I | - | S | V | F | M | E | P | L | V | D | E | L | I | H | A | W | Q | - | Q | G | V | L | T | Y | D | R | A | T | K | T | N | F | I | M | R | V | S | F | L | Y | S | I | H | D | L | P | A | Y | G | I | F | R | G | W | C | V | H | G | K | M | S | - | - | C | P | I | C | M | E | V | L | Q | G | K | R | L | K | Y | G | G | K | Y | S | F | F | D | C | H | R | Q | F | L | P | S | D | H | S | F | R | - | - | T | D | S | N | S | F | M | A | N | T | T | V | S | T | E | - | P | P | R | R | L | S | - | G | G | E | I | K | - | A | Q | L | D | S | L | V | L | A | P | - | - | - | - | - | - | - | - | - | - | - | - | - | - | - | - | - | - | - | - | - | - | - | - | - | - | - | - | - | - | - | - | - | - | - | - | - | - | - | - | - | - | - | - | - | N | G | N | G | F | V | D | Y | - | G | K | S | H | N | W | T | H | R | S | S | L | W | R | - | L | P | Y | F | H | - | - | M | L | V | L | P | H | N | I | D | V | M | H | N | E | K | N | V | A | E | A | I | F | N | T | C | F | D | I | P | D | K | T | K | D | N | V | K | A | R | L | D | Q | A | A | L | C | N | H | P | E | L | N | L | V | Q | K | Q | P | - | - | - | - | - | - | - | - | - | S | G | Q | W | V | K | P | R | A | D | F | C | L | N | R | T | Q | K | K | E | I | L | E | W | F | - | R | T | L | K | F | P | D | G | Y | G | S | N | L | R | R | G | V | N | L | K | N | M | R | I | N | G | - | L | K | S | H | D | Y | H | I | M | M | E | R | L | F | P | V | M | F | R | G | Y | - | - | - | - | - | - | - | L | P | T | - | H | V | W | E | V | L | A | - | - | - | - | - | - | - | - | - | - | - | - | - | - | - | - | - | - | - | - | - | - | - | E | V | S | F | F | Y | R | K | L | C | A | K | Q | F | D | P | I | E | M | E | N | M | E | S | Q | I | V | V | L | V | C | K | L | E | K | I | F | P | P | G | - | - | - | - | F | F | N | P | M | Q | H | L | - | - | - | - | - | - | - | - | - | M | S | G | P | V | Q | F | R | W | N | Y | P | V | E | - | - | - | - | - | - | - | - | - | - | - | - | - | - | - | - | - | - | - | - | - | - | - | - | - | - | - | - | - | R | G | - | - | - | - | - | - | - | - | - | - | - | - | - | - | - | - | - | - | - | - | - | - | - | - | - | - | - | - | - | - | - | - | - | - | - | - | - | - | - | - | - | - | - | - | - | - | - | - | - | - | - | - | - | - | - | - | - | - | - | - | - | - | - | - | - | - | - | - | - | - | - | - | - | - | Q | K | Y | L | K | S | K | V | R | N | R | A | R | V | E | A | S | I | A | E | A | Y | I | L | D | E | I | S | N | - | - | - | - | F | T | S | I | Y | F | A | D | Q | V | R | - | - | T | I | H | N | P | V | P | R | Y | N | V | A | - | Q | S | T | D | - | - | - | - | - | C | S | L | S | L | F | S | I | K | G | D | S | T | S | R | G | V | P | R | H | L | T | - | - | - | - | A | E | E | W | D | A | T | M | L | Y | V | L | T | N | L | T | E | V | D | D | - | Y | I | R | K | F | I | D | E | E | W | T | S | R | G | - | - | - | - | - | - | - | - | - | - | - | - | - | - | - | - | - | - | - | - | - | - | - | - | - | - | - | - | - | - | - | - | - | - | - | - | - | - | - | - | - | - | - | - | - | - | - | - | - | - | - | - | - | - | - | - | - | - | - | - | - | - | - | - | - | - | - | - | - | - | - | - | - | - | - | - | - | - | - | - | - | - | - | - | V | P | T | R | Q | Q | Q | E | N | I | L | Q | N | G | - | - | - | - | - | A | G | R | D | H | P | N | F | V | S | C | M | M | - | - | - | - | - | - | - | - | - | - | - | - | - | - | - | - | - | - | - | - | - | - | - | - | - | - | - | - | - | - | - | - | - | - | - | - | - | - | - | D | E | T | M | S | D | D | L | K | Q | I | A | R | G | C | H | T | R | V | L | L | Y | N | I | Y | D | V | N | G | Y | R | F | R | T | H | K | Y | E | Q | E | - | R | P | N | A | T | T | I | N | S | G | L | V | T | I | G | Q | G | E | N | D | E | M | - | - | - | - | - | - | - | - | - | - | T | E | Y | Y | G | Y | I | K | E | I | I | E | I | S | F | - | - | - | - | - | D | G | T | K | P | L | T | L | V | L | F | N | - | C | H | W | F | D | - | - | - | - | - | - | - | - | - | - | - | - | P | S | Q | V | - | - | R | Y | S | P | R | Y | - | G | L | V | E | - | - | - | - | - | - | - | - | - | - | - | V | A | H | - | - | - | - | - | A | S | I | L | - | - | P | K | F | E | P | F | V | I | A | H | Q | A | T | Q | V | Y | Y | M | P | Y | P | - | - | C | K | S | V | Q | D | L | - | T | N | - | - | - | - | - | - | - | - | - | - | W | W | V | V | Y | K | V | Q | P | I | G | R | L | E | V | - | P | - | - | - | T | D | Q | D | Y | D | - | - | - | - | - | F | V | P | N | T | D | V | V | H | Y | F | Q | E | - | D | G | L | - | - | - | - | - | L | G | S | F | V | I | D | L | G | L | N | F | D | N | N | S | E | E | A | A | S | S | R | D | T | E | D | - | - | - | - | - | - | - | - | - | - | - | - | - | - | - | - | - | - | - | - | - | - | - | - | - | - | - | - | - | - | - | - | - | - | - | - | - | - | - | - | - | - | - | - | - | - | - | - | - | - | - | - | - | - | - | - | - | - | - | - | - | - | - | - | - | - | - | - | - | - | - | - | - | - | - | - | - | - | - | - | - | - | - | - | - | - | - | - | - | - | - | - | - | - | - | - | - | - | - | - | - | - | - | - | - | - | - | - | - | - | - | - | - | - | - | - | - | - | - | - | - | - | - | - | - | - | - | - | - | - | - | - | - | - | - | - | - | - | - | - | - | - | - | - | - | - | - | - | - | - | - | - | - | - | - | - | - | - | - | - | - | - | - | - | - | - | - | - | - | - | - | - | - | - | - | - | - | - | - | - | - | - | - | - | - | - | - | - | - | - | - | - | - | - | - | - | - | - | - | - | - | - | - | - | - | - | - | - | - | - | - | - | - | - | - | - | - | - | - | - | - | - | - | - | - | - | - | - | - | - | - | - | - | - | - | - | - | - | - | - | - | - | - | - | - | - | - | - | - | - | - | - | - | - | - | - | - | - | - | - | - | - | - | - | - | - | - | - | - | - | - | - | - | - | - | - | - | - | - | - | - | - | - | - | - | - | - | - | - | - | - | - | - | - | - | - | - | - | - | - | - | - | - | - | - | - | - | - | - | - | - | - | - | - | - | - | - | - | - | - | - | - | - | - | - | - | - | - | - | - | - | - | - | - | - | - | - | - | - | - | - | - | - | - | - | - | - | - | - | - | - | - | - | - | - | - | - | - | - | - | - | - | - | - | - | - | - | - | - | - | - | - | - | - | - | - | - | - | - | - | - | - | - | - | - | - | - | - | - | - | - | - | - | - | - | - | - | - | - | - | - | - | - | - | - | - | - | - | - | - | - | - | - | - | - | - | - | - | - | - | - | - | - | - | - | - | - | - | - | - | - | - | - | - | - | - | - | - | - | - | - | - | - | - | - | - | - | - | - | - | - | - | - | - | - | - | - | - | - | - | - | - | - | - | - | - | - | - | - | - | - | - | - | - | - | - | - | - | - | - | - | - | - | - | - | - | - | - | - | - | - | - | - | - | - | - | - | - | - | - | - | - | - | - | I | C | N | G | K | D | L | E | L | L | N | G | S | S | I | - | - | Q | D | V | N | Y | D | - | - | D | D | E | S | Y | Y | A | D | S | S | N | E | D | D | E | Y | P | T | N | R | D | I | L | Y | D | E | Y | - | - | - | - | - | - | - | - | - | - | - | - | - | - | - | - | - | - | - | - | - | - | - | - | - | - | - | - | - | - | - | - | - | - | - | - | - | - | - | - | - | - | - | - | - | - | - | - | - | - | - | - | - | - | - | - | - | - | - | - | - | - | - | - | - | - | - | - | - | - | - | - | - | - | - | - | - | - | - | - | - | - | - | - | - | - | - | - | - | - | - | - | - | - | - | - | - | - | - | - | - | - | - | - | - | - | - | - | - | - | - | - | - | - | - | - | - | - | - | - | - | - | - | - | - | - | - | - | - | - | - | - | - | - | - | - | - | - | - | - | - | - | - | - | - | - | - | - | - | - | - | - | - | - | - | - | - | - | - | - | - | - | - | - | - | - | - | - | - | - | - | - | - | - | - | - | - | - | - | - | - | - | - | - | - | - | - | - | - | - | - | - | - | - | - | - | - | - | - | - | - | - | - | - | - | - | - | - | - | - | - | - | - | - | - | - | - | - | - | - | - | - | - | - | - | F | - | - | - | - | - | - | - | - | - | - | - |
|  | Rufus | - | - | - | - | - | - | - | - | - | - | - | - | - | - | - | - | M | E | T | D | R | - | S | W | M | Y | K | G | R | R | K | - | R | G | E | V | T | A | E | W | I | S | K | T | T | E | F | L | D | K | A | F | S | R | E | - | - | - | T | G | - | R | A | G | V | L | C | P | C | N | Y | C | N | N | T | R | P | Q | T | - | R | - | - | - | - | D | M | M | M | K | H | L | C | K | F | G | F | R | P | A | Y | T | V | - | - | - | W | V | Y | H | - | G | E | - | - | T | Y | P | A | R | E | S | D | I | C | Q | S | - | - | - | - | - | - | - | - | - | - | - | - | - | - | - | - | - | - | - | - | - | - | - | - | - | - | - | - | - | - | - | - | - | - | - | - | - | - | - | - | - | - | - | - | - | T | E | D | - | - | - | - | E | D | R | V | A | E | I | D | R | M | D | E | M | I | D | D | V | H | D | A | Y | V | S | V | - | - | - | - | - | - | - | - | - | - | - | - | - | - | - | - | - | - | - | - | - | - | - | - | - | - | - | - | - | - | - | - | - | E | E | E | D | P | E | - | - | - | - | - | - | - | - | - | - | - | P | T | A | Q | A | F | F | Q | M | L | S | A | A | T | Q | P | L | H | - | - | - | E | H | T | - | - | Q | V | S | Q | L | D | A | I | T | R | L | L | A | V | K | S | Q | Y | A | I | S | - | - | - | - | - | I | A | G | F | D | A | L | L | N | V | I | - | C | A | L | L | P | Q | G | H | K | L | P | A | N | L | Y | E | A | K | K | V | L | S | A | L | N | M | P | Y | E | K | I | D | A | C | P | K | N | C | M | L | F | R | K | E | - | N | T | Q | K | T | H | C | D | N | C | G | E | S | R | Y | V | E | V | E | D | S | N | G | - | - | - | - | - | - | - | - | - | - | - | - | - | - | - | - | - | - | - | - | - | - | - | - | - | - | - | - | - | - | - | - | - | - | - | - | - | H | K | K | Q | L | T | V | A | K | K | V | L | R | Y | L | P | F | I | P | R | I | Q | R | L | Y | M | S | E | S | Q | A | K | Q | M | T | W | H | K | N | G | - | - | - | - | - | - | - | - | - | - | H | R | - | - | Y | H | T | D | K | - | - | - | - | - | I | V | H | P | A | D | G | E | A | W | K | K | F | D | R | D | F | T | G | - | F | S | M | E | A | R | N | V | R | I | A | I | A | T | D | G | F | C | P | F | G | M | G | S | S | S | Y | T | C | W | P | M | F | V | I | P | L | N | L | - | P | P | G | V | C | M | R | Q | H | N | M | F | L | T | L | I | I | P | G | P | N | Y | P | G | K | H | I | - | S | V | F | M | E | P | L | V | D | E | L | I | H | A | W | Q | - | Q | G | V | L | T | Y | D | R | A | T | K | T | N | F | I | M | R | V | S | F | L | Y | S | I | H | D | L | P | A | Y | G | I | F | S | G | W | C | V | H | G | K | M | P | - | - | C | P | T | C | M | E | A | L | Q | G | K | R | L | K | F | G | G | K | Y | S | F | F | D | C | H | R | Q | F | L | P | I | D | H | S | F | R | - | - | S | D | S | N | S | F | L | S | N | T | T | V | S | N | E | - | P | P | L | R | L | S | - | G | Q | E | I | R | - | A | R | L | D | N | L | V | P | A | A | - | - | - | - | - | - | - | - | - | - | - | - | - | - | - | - | - | - | - | - | - | - | - | - | - | - | - | - | - | - | - | - | - | - | - | - | - | - | - | - | - | - | - | - | - | N | G | D | K | F | V | G | Y | - | G | K | D | H | H | W | T | H | I | S | S | L | W | R | - | L | P | Y | F | H | - | - | K | L | V | L | P | H | N | I | D | V | M | H | N | E | K | N | V | A | E | A | I | F | N | T | C | F | D | I | P | D | Q | T | K | D | N | V | K | A | R | L | D | Q | A | A | L | C | N | R | P | E | L | N | L | V | Q | K | H | A | - | - | - | - | - | - | - | - | - | S | S | Q | W | V | K | P | R | A | D | F | C | L | N | R | A | Q | K | K | E | V | L | E | W | F | - | Q | T | L | K | F | P | D | G | Y | G | S | N | L | R | R | G | V | N | L | K | T | M | R | I | N | G | - | L | K | S | H | D | Y | H | I | M | M | E | R | I | F | P | V | M | F | R | G | Y | - | - | - | - | - | - | - | L | P | T | - | H | V | W | E | V | L | A | - | - | - | - | - | - | - | - | - | - | - | - | - | - | - | - | - | - | - | - | - | - | - | E | V | S | F | F | Y | R | K | L | C | A | K | Q | F | D | P | I | E | M | A | N | M | Q | S | Q | V | I | V | L | V | C | K | L | E | K | I | F | P | P | G | - | - | - | - | F | F | N | P | M | Q | H | L | M | I | H | L | P | Y | E | A | S | M | G | G | P | V | Q | F | R | W | N | Y | P | I | E | - | - | - | - | - | - | - | - | - | - | - | - | - | - | - | - | - | - | - | - | - | - | - | - | - | - | - | - | - | R | G | - | - | - | - | - | - | - | - | - | - | - | - | - | - | - | - | - | - | - | - | - | - | - | - | - | - | - | - | - | - | - | - | - | - | - | - | - | - | - | - | - | - | - | - | - | - | - | - | - | - | - | - | - | - | - | - | - | - | - | - | - | - | - | - | - | - | - | - | - | - | - | - | - | - | Q | K | Y | L | K | S | K | V | R | N | R | A | R | V | E | A | S | I | A | E | A | Y | I | L | D | E | I | S | N | - | - | - | - | F | T | S | I | Y | F | A | D | Q | V | Q | - | - | T | I | H | N | P | V | P | R | Y | N | V | A | T | Q | S | I | D | - | - | - | - | - | C | S | L | N | L | F | S | I | R | G | D | S | T | S | R | G | V | P | R | H | L | T | - | - | - | - | R | E | E | W | N | A | A | M | L | Y | V | L | T | N | L | T | E | V | D | D | - | Y | I | G | K | F | I | D | E | E | W | T | R | R | G | - | - | - | - | - | - | - | - | - | - | - | - | - | - | - | - | - | - | - | - | - | - | - | - | - | - | - | - | - | - | - | - | - | - | - | - | - | - | - | - | - | - | - | - | - | - | - | - | - | - | - | - | - | - | - | - | - | - | - | - | - | - | - | - | - | - | - | - | - | - | - | - | - | - | - | - | - | - | - | - | - | - | - | - | V | P | T | R | Q | Q | Q | E | N | I | L | R | N | G | - | - | - | - | - | A | G | R | D | R | P | N | F | V | S | C | M | L | - | - | - | - | - | - | - | - | - | - | - | - | - | - | - | - | - | - | - | - | - | - | - | - | - | - | - | - | - | - | - | - | - | - | - | - | - | - | - | D | E | T | M | S | D | D | L | R | Q | V | A | K | G | C | H | T | R | V | L | Q | Y | N | M | Y | D | V | N | G | Y | R | F | R | T | H | K | Y | E | H | E | - | R | P | N | A | T | T | I | N | S | G | L | V | T | I | G | Q | G | E | N | S | D | T | - | - | - | - | - | - | - | - | - | - | T | E | Y | Y | G | Y | I | K | E | I | V | E | I | S | F | - | - | - | - | - | D | G | R | K | P | L | T | L | V | L | F | N | - | C | H | W | F | D | - | - | - | - | - | - | - | - | - | - | - | - | P | S | K | V | - | - | R | Y | T | P | R | Y | - | G | L | V | E | - | - | - | - | - | - | - | - | - | - | - | V | A | H | - | - | - | - | - | A | S | I | L | - | - | P | K | F | E | P | F | V | L | S | H | Q | A | T | Q | V | Y | Y | M | P | Y | P | - | - | C | K | S | V | Q | D | L | - | T | N | - | - | - | - | - | - | - | - | - | - | W | W | V | V | Y | K | V | Q | P | I | G | R | L | Q | V | - | P | - | - | - | N | D | Q | D | Y | N | - | - | - | - | - | S | V | P | N | S | N | V | V | H | Y | F | Q | E | - | D | G | L | - | - | - | - | - | S | G | S | F | V | I | D | L | G | Q | E | L | N | N | D | P | E | Q | V | A | I | S | R | D | T | E | D | - | - | - | - | - | - | - | - | - | - | - | - | - | - | - | - | - | - | - | - | - | - | - | - | - | - | - | - | - | - | - | - | - | - | - | - | - | - | - | - | - | - | - | - | - | - | - | - | - | - | - | - | - | - | - | - | - | - | - | - | - | - | - | - | - | - | - | - | - | - | - | - | - | - | - | - | - | - | - | - | - | - | - | - | - | - | - | - | - | - | - | - | - | - | - | - | - | - | - | - | - | - | - | - | - | - | - | - | - | - | - | - | - | - | - | - | - | - | - | - | - | - | - | - | - | - | - | - | - | - | - | - | - | - | - | - | - | - | - | - | - | - | - | - | - | - | - | - | - | - | - | - | - | - | - | - | - | - | - | - | - | - | - | - | - | - | - | - | - | - | - | - | - | - | - | - | - | - | - | - | - | - | - | - | - | - | - | - | - | - | - | - | - | - | - | - | - | - | - | - | - | - | - | - | - | - | - | - | - | - | - | - | - | - | - | - | - | - | - | - | - | - | - | - | - | - | - | - | - | - | - | - | - | - | - | - | - | - | - | - | - | - | - | - | - | - | - | - | - | - | - | - | - | - | - | - | - | - | - | - | - | - | - | - | - | - | - | - | - | - | - | - | - | - | - | - | - | - | - | - | - | - | - | - | - | - | - | - | - | - | - | - | - | - | - | - | - | - | - | - | - | - | - | - | - | - | - | - | - | - | - | - | - | - | - | - | - | - | - | - | - | - | - | - | - | - | - | - | - | - | - | - | - | - | - | - | - | - | - | - | - | - | - | - | - | - | - | - | - | - | - | - | - | - | - | - | - | - | - | - | - | - | - | - | - | - | - | - | - | - | - | - | - | - | - | - | - | - | - | - | - | - | - | - | - | - | - | - | - | - | - | - | - | - | - | - | - | - | - | - | - | - | - | - | - | - | - | - | - | - | - | - | - | - | - | - | - | - | - | - | - | - | - | - | - | - | - | - | - | - | - | - | - | - | - | - | - | - | - | - | - | - | - | - | - | - | - | - | - | - | - | - | - | - | - | - | - | - | - | - | - | - | - | - | - | - | - | - | - | - | - | - | - | - | - | - | - | - | - | - | - | - | - | - | - | - | - | - | - | - | - | - | - | - | - | - | - | - | - | - | - | - | - | - | I | C | N | D | K | D | L | K | L | L | N | G | S | S | I | - | - | Q | D | E | D | D | D | D | E | D | D | E | S | Y | C | T | S | S | S | A | E | D | D | E | V | P | T | S | R | D | I | L | F | D | E | Y | - | - | - | - | - | - | - | - | - | - | - | - | - | - | - | - | - | - | - | - | - | - | - | - | - | - | - | - | - | - | - | - | - | - | - | - | - | - | - | - | - | - | - | - | - | - | - | - | - | - | - | - | - | - | - | - | - | - | - | - | - | - | - | - | - | - | - | - | - | - | - | - | - | - | - | - | - | - | - | - | - | - | - | - | - | - | - | - | - | - | - | - | - | - | - | - | - | - | - | - | - | - | - | - | - | - | - | - | - | - | - | - | - | - | - | - | - | - | - | - | - | - | - | - | - | - | - | - | - | - | - | - | - | - | - | - | - | - | - | - | - | - | - | - | - | - | - | - | - | - | - | - | - | - | - | - | - | - | - | - | - | - | - | - | - | - | - | - | - | - | - | - | - | - | - | - | - | - | - | - | - | - | - | - | - | - | - | - | - | - | - | - | - | - | - | - | - | - | - | - | - | - | - | - | - | - | - | - | - | - | - | - | - | - | - | - | - | - | - | - | - | - | - | - | - | F | - | - | - | - | - | - | - | - | - | - | - |
|  | Isaac | - | - | - | - | - | - | - | - | - | - | - | - | - | - | - | - | - | M | A | D | N | - | Q | W | M | Y | S | G | F | I | R | - | R | N | R | V | T | S | E | W | I | A | K | T | D | V | Y | L | K | E | I | F | R | R | P | M | - | - | - | - | - | R | I | I | P | P | C | P | C | A | R | C | A | R | R | H | R | R | N | - | Q | - | - | - | - | T | D | M | S | E | H | L | R | T | H | G | Y | M | P | N | F | D | M | - | - | - | P | P | I | N | I | A | E | - | - | Q | D | H | G | R | E | E | V | M | R | Q | R | - | - | - | - | - | - | - | - | - | - | - | - | - | - | - | - | - | - | - | - | - | - | - | - | - | - | - | - | - | - | - | - | - | - | - | - | - | - | - | - | - | - | - | - | - | I | N | R | - | - | - | - | - | - | - | - | Y | E | D | D | G | V | R | D | M | L | M | H | V | I | V | A | D | S | A | N | A | T | P | - | - | - | - | - | - | - | - | - | - | - | - | - | - | - | - | - | - | - | - | - | - | - | - | - | - | - | S | E | N | E | P | E | D | P | E | - | - | - | - | - | - | - | - | - | - | - | A | T | A | K | A | F | L | E | V | L | A | S | S | K | K | P | L | Y | - | - | - | A | G | A | - | - | K | I | S | Q | L | D | A | I | S | Q | L | I | A | V | K | A | E | Y | G | C | S | - | - | - | - | - | Q | K | C | F | E | A | F | L | G | V | W | - | A | N | S | L | P | E | G | H | E | L | P | K | S | M | Y | D | T | K | K | I | M | K | A | L | S | M | D | Y | E | K | I | D | V | C | P | K | N | C | F | L | F | R | H | E | - | Y | A | D | D | K | Y | C | R | K | C | G | S | S | W | Y | I | E | V | V | G | E | D | G | - | - | - | - | - | - | - | - | - | - | - | - | - | - | - | - | - | - | - | - | - | - | - | - | - | - | - | - | - | - | - | - | - | - | - | - | - | E | K | K | Q | L | T | I | P | V | K | V | L | R | Y | L | D | F | I | K | R | L | Q | R | L | F | I | T | K | E | S | A | K | M | M | K | W | H | K | E | G | - | - | - | - | - | - | - | - | - | - | I | R | - | - | Y | N | P | K | K | - | - | - | - | - | I | I | H | P | S | G | G | E | A | W | K | S | F | D | E | E | Y | P | E | - | E | A | A | E | A | G | N | V | R | I | A | I | S | G | E | G | L | N | P | Y | G | M | S | S | N | P | Y | S | C | W | P | V | F | V | I | P | L | N | L | - | P | P | G | A | L | M | Q | R | K | T | M | F | L | S | L | I | I | P | G | P | D | Y | P | G | K | Q | L | - | G | V | F | M | Q | P | L | V | D | A | L | H | H | S | W | H | - | F | P | R | L | T | Y | D | R | D | L | Q | R | N | F | L | M | K | V | W | L | H | Y | C | M | H | D | F | H | G | Y | A | L | F | C | G | W | C | T | S | G | K | M | P | - | - | C | P | V | C | M | Q | A | L | R | M | I | W | L | S | K | G | G | K | Y | V | A | F | D | L | H | R | Q | F | L | P | P | D | H | P | D | R | - | - | E | D | K | K | N | F | M | K | G | R | V | V | H | E | V | T | E | I | P | T | F | S | - | G | A | D | V | L | - | A | Q | L | K | A | L | K | P | K | V | K | G | K | - | - | - | - | - | - | - | - | - | - | - | - | - | - | - | - | - | - | - | - | - | - | - | - | - | - | - | - | - | - | - | - | - | - | - | - | - | - | - | G | K | A | K | A | K | G | F | E | G | Y | - | G | E | T | H | N | W | T | H | I | T | P | F | S | E | - | L | P | Y | F | K | - | - | D | L | K | L | P | Y | N | I | D | V | M | H | T | E | K | N | V | A | E | S | L | - | S | T | R | S | S | T | F | L | I | R | E | G | Y | V | K | A | R | A | D | Q | Q | R | I | C | D | R | P | R | L | N | M | K | P | P | T | G | - | - | - | - | - | - | - | - | G | R | K | N | W | F | K | P | D | A | D | F | V | L | K | P | P | E | K | K | E | V | L | I | W | L | K | Q | I | L | K | F | T | D | G | Y | A | S | N | I | S | K | G | V | N | L | S | T | G | K | V | T | G | - | V | K | S | H | D | Y | H | V | W | I | E | R | I | M | P | V | M | V | R | G | Y | - | - | - | - | - | - | - | V | P | E | - | H | V | W | R | V | L | A | - | - | - | - | - | - | - | - | - | - | - | - | - | - | - | - | - | - | - | - | - | - | - | E | L | S | H | F | F | C | M | L | C | A | K | E | V | S | K | E | V | I | E | K | L | H | K | K | A | P | E | L | I | V | K | L | E | K | I | F | P | P | G | - | - | - | - | F | F | T | P | M | T | H | L | I | L | H | L | A | N | E | V | L | L | G | G | P | V | Q | N | R | W | Q | Y | G | P | E | - | - | - | - | - | - | - | - | - | - | - | - | - | - | - | - | - | - | - | - | - | - | - | - | - | - | - | - | - | R | Q | - | - | - | - | - | - | - | - | - | - | - | - | - | - | - | - | - | - | - | - | - | - | - | - | - | - | - | - | - | - | - | - | - | - | - | - | - | - | - | - | - | - | - | - | - | - | - | - | - | - | - | - | - | - | - | - | - | - | - | - | - | - | - | - | - | - | - | - | - | - | - | - | - | - | N | K | H | L | R | R | K | C | G | N | K | A | K | I | K | A | S | I | A | E | A | V | I | L | E | E | V | S | D | - | - | - | - | L | R | T | S | Y | Y | P | D | H | V | P | - | - | H | L | H | N | K | V | P | R | Y | N | I | E | E | P | K | Y | Q | - | - | - | - | - | P | R | L | D | L | F | N | A | Q | G | R | R | A | G | A | S | K | S | Y | N | M | P | - | - | - | - | R | Q | E | W | E | H | L | M | F | Y | I | L | H | N | I | K | E | V | E | E | V | W | M | S | D | F | V | Q | E | E | W | T | G | V | N | - | - | - | - | - | - | - | - | - | - | - | - | - | - | - | - | - | - | - | - | - | - | - | - | - | - | - | - | - | - | - | - | - | - | - | - | - | - | - | - | - | - | - | - | - | - | - | - | - | - | - | - | - | - | - | - | - | - | - | - | - | - | - | - | - | - | - | - | - | - | - | - | - | - | - | - | - | - | - | - | - | - | - | - | P | P | T | E | A | E | A | L | T | L | L | R | H | G | - | - | - | - | - | - | N | P | G | R | K | N | F | V | A | W | F | M | D | K | G | K | D | - | - | - | - | - | - | - | - | - | - | - | - | - | - | - | - | - | - | - | - | - | - | - | - | - | - | - | - | - | - | - | - | - | L | N | I | S | M | D | D | E | L | R | W | V | S | M | G | F | D | P | A | V | M | T | C | K | K | Y | D | V | N | G | Y | R | F | H | T | E | E | H | Q | N | S | - | R | P | D | P | K | T | I | N | T | R | V | Y | T | P | G | Q | N | S | - | - | - | - | - | - | - | - | - | - | - | - | - | - | V | D | Y | Y | G | R | V | Q | N | I | Y | E | V | K | F | - | - | - | - | R | Q | G | R | E | T | L | S | L | P | V | F | K | - | C | R | W | F | D | - | - | - | - | - | - | - | - | - | - | - | - | P | R | E | G | - | V | K | H | T | P | S | I | - | G | L | V | E | - | - | - | - | - | - | - | - | - | - | - | V | K | P | - | - | - | - | - | S | T | V | Y | - | - | A | G | A | D | L | F | I | A | A | T | Q | A | T | Q | V | Y | Y | L | P | Y | P | - | - | C | Q | K | - | V | Y | L | - | K | G | - | - | - | - | - | - | - | - | - | - | W | E | V | V | F | K | V | S | P | H | G | K | L | R | T | Q | T | - | - | - | M | M | I | I | T | T | - | - | - | - | - | - | - | - | - | - | - | - | - | - | - | - | - | - | - | - | - | - | - | - | - | - | - | - | - | - | - | - | - | - | - | - | - | - | - | - | - | - | - | - | - | - | - | - | - | - | - | - | - | - | - | - | - | - | - | - | - | - | - | - | - | - | - | - | - | - | - | - | - | - | - | - | - | - | - | - | - | - | - | - | - | - | - | - | - | - | - | - | - | - | - | - | - | - | - | - | - | - | - | - | - | - | - | - | - | - | - | - | - | - | - | - | - | - | - | - | - | - | - | - | - | - | - | - | - | - | - | - | - | - | - | - | - | - | - | - | - | - | - | - | - | - | - | - | - | - | - | - | - | - | - | - | - | - | - | - | - | - | - | - | - | - | - | - | - | - | - | - | - | - | - | - | - | - | - | - | - | - | - | - | - | - | - | - | - | - | - | - | - | - | - | - | - | - | - | - | - | - | - | - | - | - | - | - | - | - | - | - | - | - | - | - | - | - | - | - | - | - | - | - | - | - | - | - | - | - | - | - | - | - | - | - | - | - | - | - | - | - | - | - | - | - | - | - | - | - | - | - | - | - | - | - | - | - | - | - | - | - | - | - | - | - | - | - | - | - | - | - | - | - | - | - | - | - | - | - | - | - | - | - | - | - | - | - | - | - | - | - | - | - | - | - | - | - | - | - | - | - | - | - | - | - | - | - | - | - | - | - | - | - | - | - | - | - | - | - | - | - | - | - | - | - | - | - | - | - | - | - | - | - | - | - | - | - | - | - | - | - | - | - | - | - | - | - | - | - | - | - | - | - | - | - | - | - | - | - | - | - | - | - | - | - | - | - | - | - | - | - | - | - | - | - | - | - | - | - | - | - | - | - | - | - | - | - | - | - | - | - | - | - | - | - | - | - | - | - | - | - | - | - | - | - | - | - | - | - | - | - | - | - | - | - | - | - | - | - | - | - | - | - | - | - | - | - | - | - | - | - | - | - | - | - | - | - | - | - | - | - | - | - | - | - | - | - | - | - | - | - | - | - | - | - | - | - | - | - | - | - | - | - | - | - | - | - | - | - | - | - | - | - | - | - | - | - | - | - | - | - | - | - | - | - | - | - | - | - | - | - | - | - | - | - | - | - | - | - | - | - | - | - | - | - | - | - | - | - | - | - | - | - | - | - | - | - | - | - | - | - | - | - | - | - | - | - | - | - | - | - | - | - | - | - | - | - | - | - | - | - | - | - | - | - | - | - | - | - | - | - | - | - | - | - | - | - | - | - | - | - | - | - | - | - | - | - | - | - | - | - | - | - | - | - | - | - | - | - | - | - | - | - | - | - | - | - | - | - | - | - | - | - | - | - | - | - | - | - | - | - | - | - | - | - | - | - | - | - | - | - | - | - | - | - | - | - | - | - | - | - | - | - | - | - | - | - | - | - | - | - | - | - | - | - | - | - | - | - | - | - | - | - | - | - | - | - | - | - | - | - | - | - | - | - | - | - | - | - | - | - | - | - | - | - | - | - | - | - | - | - | - | - | - | - | - | - | - | - | - | - | - | - | - | - | - | - | - | - | - | - | - | - | - | - | - | - | - | - | - | - | - | - | - | - | - | - | - | - | - | - | - | - | - | - | - | - | - | - | - | - | - | - | - | - | - | - | - | - | - | - | - | - | - | - | - | - | - | - | - | - | - | - | - | - | - | - | - | - | - | - | - | - | - | - | - | - | - | - | - | - | - | - | - | - | - | - | - | - | - | - | - | - | - | - | - | - | - | - | - | - | - | - | - | - | - | - | - | - | - | - | - | - | - | - | - | - | - | - | - | - | - | - | - | - | - | - | - | - | - | - | - | - | - | - | - | - | - | - | - | - | - | - | - | - | - | - | - | - | - | - | - | - | - | - | - | - | - | - | - | - | - | - | - | - | - |
|  | Baldur | - | - | - | - | - | - | - | - | - | - | - | - | - | - | - | - | - | M | T | S | R | - | E | W | M | Y | S | G | W | T | R | - | G | K | A | P | T | N | E | W | I | D | N | T | T | Q | F | L | N | R | A | F | S | M | Q | E | V | - | V | K | - | D | G | T | I | K | C | P | C | A | Q | C | R | N | Y | F | R | H | K | - | R | - | - | - | - | D | T | I | E | L | H | L | C | K | Y | G | Y | K | E | N | Y | G | I | - | - | - | W | T | S | H | - | G | E | - | - | R | P | V | I | N | D | N | D | P | G | P | S | - | - | - | - | - | - | - | - | - | - | - | - | - | - | - | - | - | - | - | - | - | - | - | - | - | - | - | - | - | - | - | - | - | - | - | - | - | - | - | - | - | - | - | - | - | L | I | D | - | - | - | - | H | E | G | F | G | E | S | D | R | M | D | N | M | L | V | D | L | A | S | A | Q | P | P | E | - | - | - | - | - | - | - | - | - | - | - | - | - | - | - | - | - | - | - | - | - | - | - | - | - | - | - | - | - | - | - | - | - | S | S | E | E | P | A | - | - | - | - | - | - | - | - | - | - | - | H | Y | A | K | A | F | Y | R | M | V | A | S | A | D | E | L | I | H | - | - | - | E | N | T | - | - | T | H | S | C | L | S | A | V | A | R | L | L | A | M | K | S | Q | Y | N | M | S | - | - | - | - | - | V | A | H | Y | D | D | V | L | G | I | I | - | H | E | F | L | P | P | E | S | K | L | A | K | D | F | Y | R | S | K | K | L | L | E | G | L | G | M | P | Y | V | K | I | D | V | C | Y | N | N | C | M | L | Y | Y | K | E | - | D | E | H | K | E | K | C | D | F | C | G | T | S | R | Y | - | - | - | - | - | - | - | - | - | - | - | - | - | - | - | - | - | - | - | - | - | - | - | - | - | - | - | - | - | - | - | - | - | - | - | - | - | - | - | - | - | - | - | - | - | E | N | G | Q | N | K | T | P | R | K | V | L | R | Y | L | P | I | K | D | R | L | Q | R | L | Y | A | H | E | E | I | A | R | L | L | Q | S | H | S | R | S | - | - | - | - | - | - | - | - | - | - | - | - | - | - | - | Q | S | G | N | - | - | - | - | - | M | V | H | P | C | D | G | E | A | W | Q | Q | F | D | E | D | F | Q | D | - | F | A | Q | D | P | R | N | V | R | L | A | L | A | T | D | G | F | T | P | Y | S | L | G | A | A | P | Y | S | C | W | P | V | F | I | T | P | L | N | F | - | P | P | G | V | C | M | R | P | E | Y | T | F | L | T | L | V | I | P | G | P | E | H | P | G | K | K | L | - | S | V | L | M | Q | P | L | V | D | E | L | L | K | L | W | E | - | - | G | V | E | T | W | D | A | S | R | K | Q | N | F | T | M | R | A | I | F | L | W | S | I | H | D | F | P | A | Y | G | I | F | A | G | W | S | T | H | G | R | L | A | - | - | C | P | I | C | M | G | D | S | Q | S | F | Q | L | R | N | G | R | K | P | C | W | F | D | C | H | R | R | F | L | P | N | E | H | E | F | R | - | - | T | Q | L | N | A | F | R | K | N | T | F | M | L | D | E | - | P | P | R | I | L | T | - | G | E | E | I | K | - | E | E | M | Y | A | C | V | D | D | T | - | - | - | - | - | - | - | - | - | - | - | - | - | - | - | - | - | - | - | - | - | - | - | - | - | - | - | - | - | - | - | - | - | - | - | - | - | - | - | - | - | - | - | - | - | - | - | - | - | - | E | N | F | - | G | K | T | H | H | W | T | H | I | S | C | F | W | Q | - | L | P | Y | F | D | - | - | K | L | K | L | R | H | N | I | D | L | M | H | N | E | K | N | V | A | E | S | I | W | N | T | C | F | D | I | Q | D | K | T | K | D | N | V | K | A | R | K | D | L | A | E | I | C | S | R | P | L | L | Q | L | V | S | K | G | - | - | - | - | - | - | - | - | - | - | N | G | K | W | H | K | P | R | A | S | F | C | I | D | R | N | D | K | T | T | I | L | K | W | F | - | Q | E | L | K | F | P | D | G | Y | A | A | N | I | R | R | G | V | N | L | L | Q | R | K | I | F | G | - | L | K | S | H | D | Y | H | V | F | M | E | R | L | L | P | V | A | F | R | G | F | - | - | - | - | - | - | - | I | P | E | - | S | V | W | K | C | L | A | - | - | - | - | - | - | - | - | - | - | - | - | - | - | - | - | - | - | - | - | - | - | - | E | L | S | F | F | Y | R | Q | L | C | A | K | E | L | N | K | D | T | I | R | S | L | E | E | N | V | A | V | L | I | C | K | L | E | K | I | F | P | P | G | - | - | - | - | F | F | N | P | M | Q | H | L | I | I | H | L | P | Y | E | A | R | L | G | G | P | V | Q | F | R | W | N | Y | P | Y | E | - | - | - | - | - | - | - | - | - | - | - | - | - | - | - | - | - | - | - | - | - | - | - | - | - | - | - | - | - | R | F | - | - | - | - | - | - | - | - | - | - | - | - | - | - | - | - | - | - | - | - | - | - | - | - | - | - | - | - | - | - | - | - | - | - | - | - | - | - | - | - | - | - | - | - | - | - | - | - | - | - | - | - | - | - | - | - | - | - | - | - | - | - | - | - | - | - | - | - | - | - | - | - | - | - | I | Q | K | L | R | K | K | V | R | N | K | A | R | V | E | G | S | I | V | E | A | Y | L | V | E | E | T | S | N | - | - | - | - | F | L | S | L | Y | F | N | P | K | V | R | - | - | S | A | R | N | K | M | H | R | Y | D | D | G | S | S | Q | F | D | - | - | - | S | T | C | N | L | E | I | F | Q | Y | P | G | R | C | I | S | P | R | G | C | R | A | L | T | - | - | - | - | I | E | Q | Y | K | A | A | T | L | Y | I | L | T | N | T | P | E | M | D | D | - | F | F | K | Y | - | - | - | - | - | - | - | - | - | - | - | - | - | - | - | - | - | - | - | - | - | - | - | - | - | - | - | - | - | - | - | - | - | - | - | - | - | - | - | - | - | - | - | - | - | - | - | - | - | - | - | - | - | - | - | - | - | - | - | - | - | - | - | - | - | - | - | - | - | - | - | - | - | - | - | - | - | - | - | - | - | - | - | - | - | - | - | - | - | - | - | - | - | - | Y | D | S | N | H | L | R | H | C | L | S | F | N | - | - | - | - | L | Q | S | T | K | T | N | T | N | I | C | H | L | - | - | - | Q | C | M | K | - | - | - | - | - | - | - | - | - | - | - | - | - | - | - | - | - | - | - | - | - | - | - | - | - | - | - | - | - | - | - | - | - | - | S | S | S | I | H | N | A | L | R | Q | M | S | Y | G | F | R | S | T | V | S | T | Y | G | V | Y | D | I | N | G | Y | R | F | R | S | E | K | Y | E | S | K | - | K | S | G | L | T | T | T | N | S | G | V | C | V | S | C | V | G | E | N | N | D | V | - | - | - | - | - | - | - | - | - | - | L | E | Y | Y | G | I | I | K | D | I | L | K | I | S | W | - | - | - | - | - | E | G | S | M | N | L | E | L | V | L | F | D | - | C | Y | W | F | D | - | - | - | - | - | - | - | - | - | - | - | - | P | T | S | T | G | V | R | R | T | D | N | L | - | G | L | V | E | - | - | - | - | - | - | - | - | - | - | - | I | K | H | - | - | - | - | - | T | S | R | L | - | - | S | T | F | E | P | F | V | M | A | S | Q | V | T | Q | V | Y | Y | L | P | Y | P | - | - | C | T | T | R | R | D | L | - | S | E | - | - | - | - | - | - | - | - | - | - | W | W | V | V | H | H | V | N | P | R | G | Y | V | Q | K | V | E | - | - | - | N | T | N | N | D | S | N | P | - | - | - | N | D | D | Q | T | Q | D | V | S | F | Y | Q | E | - | D | G | L | - | - | - | - | - | E | G | T | F | I | I | D | L | G | A | D | L | G | N | M | T | P | P | I | L | D | E | - | - | - | - | - | - | - | - | - | - | - | - | - | - | - | - | - | - | - | - | - | - | - | - | - | - | - | - | - | - | - | - | - | - | - | - | - | - | - | - | - | - | - | - | - | - | - | - | - | - | - | - | - | - | - | - | - | - | - | - | - | - | - | - | - | - | - | - | - | - | - | - | - | - | - | - | - | - | - | - | - | - | - | - | - | - | - | - | - | - | - | - | - | - | - | - | - | - | - | - | - | - | - | - | - | - | - | - | - | - | - | - | - | - | - | - | - | - | - | - | - | - | - | - | - | - | - | - | - | - | - | - | - | - | - | - | - | - | - | - | - | - | - | - | - | - | - | - | - | - | - | - | - | - | - | - | - | - | - | - | - | - | - | - | - | - | - | - | - | - | - | - | - | - | - | - | - | - | - | - | - | - | - | - | - | - | - | - | - | - | - | - | - | - | - | - | - | - | - | - | - | - | - | - | - | - | - | - | - | - | - | - | - | - | - | - | - | - | - | - | - | - | - | - | - | - | - | - | - | - | - | - | - | - | - | - | - | - | - | - | - | - | - | - | - | - | - | - | - | - | - | - | - | - | - | - | - | - | - | - | - | - | - | - | - | - | - | - | - | - | - | - | - | - | - | - | - | - | - | - | - | - | - | - | - | - | - | - | - | - | - | - | - | - | - | - | - | - | - | - | - | - | - | - | - | - | - | - | - | - | - | - | - | - | - | - | - | - | - | - | - | - | - | - | - | - | - | - | - | - | - | - | - | - | - | - | - | - | - | - | - | - | - | - | - | - | - | - | - | - | - | - | - | - | - | - | - | - | - | - | - | - | - | - | - | - | - | - | - | - | - | - | - | - | - | - | - | - | - | - | - | - | - | - | - | - | - | - | - | - | - | - | - | - | - | - | - | - | - | - | - | - | - | - | - | - | - | - | - | - | - | - | - | - | - | - | - | - | - | - | - | - | - | - | - | - | - | - | - | - | - | - | - | - | - | - | - | - | - | - | - | - | - | - | - | - | - | - | - | - | - | - | - | - | - | - | - | - | - | - | - | - | - | - | - | - | - | - | - | - | - | - | - | - | - | - | - | - | - | - | - | - | - | - | - | - | - | - | - | - | - | - | - | - | - | - | - | - | - | - | - | - | - | - | - | - | - | - | - | I | T | N | A | K | D | L | E | F | L | E | K | Q | T | Q | A | E | D | N | Y | D | E | D | D | N | E | E | E | E | E | E | E | E | E | E | E | E | E | E | E | E | E | E | N | D | E | E | D | E | G | T | Q | - | - | - | - | - | - | - | - | - | - | - | - | - | - | - | - | - | - | - | - | - | - | - | - | - | - | - | - | - | - | - | - | - | - | - | - | - | - | - | - | - | - | - | - | - | - | - | - | - | - | - | - | - | - | - | - | - | - | - | - | - | - | - | - | - | - | - | - | - | - | - | - | - | - | - | - | - | - | - | - | - | - | - | - | - | - | - | - | - | - | - | - | - | - | - | - | - | - | - | - | - | - | - | - | - | - | - | - | - | - | - | - | - | - | - | - | - | - | - | - | - | - | - | - | - | - | - | - | - | - | - | - | - | - | - | - | - | - | - | - | - | - | - | - | - | - | - | - | - | - | - | - | - | - | - | - | - | - | - | - | - | - | - | - | - | - | - | - | - | - | - | - | - | - | - | - | - | - | - | - | - | - | - | - | - | - | - | - | - | - | - | - | - | - | - | - | - | - | - | - | - | - | - | - | - | - | - | - | - | - | - | - | - | - | - | - | - | - | - | - | - | - | - | - | - | P | P | - | - | - | A | Y | D | P | N | D | F |
|  | Seamus | - | - | - | - | - | - | - | - | - | - | - | - | - | - | - | - | - | M | A | E | R | - | N | W | M | Y | N | G | W | H | Y | - | G | S | A | P | S | D | E | W | I | E | K | T | N | M | F | L | D | H | A | F | S | I | P | D | L | - | V | Q | - | D | G | S | I | K | C | P | C | G | R | C | R | N | Y | M | R | H | T | - | R | - | - | - | - | D | I | I | E | A | H | L | C | K | F | G | F | K | E | N | Y | E | T | - | - | - | W | T | A | H | - | G | E | - | - | E | H | I | R | N | N | E | P | V | S | P | - | - | - | - | - | - | - | - | - | - | - | - | - | - | - | - | - | - | - | - | - | - | - | - | - | - | - | - | - | - | - | - | - | - | - | - | - | - | - | - | - | - | - | - | - | - | L | A | H | - | - | - | - | E | E | G | F | D | Q | P | D | R | M | D | E | M | L | L | D | I | G | A | V | Q | P | P | G | - | - | - | - | - | - | - | - | - | - | - | - | - | - | - | - | - | - | - | - | - | - | - | - | - | - | - | - | - | - | - | - | - | I | D | E | E | P | T | - | - | - | - | - | - | - | - | - | - | - | S | S | A | S | A | F | Y | R | M | V | D | G | A | N | E | S | V | H | - | - | - | D | N | T | - | - | M | H | T | S | L | S | A | I | A | R | L | L | A | L | K | S | E | Y | N | M | S | - | - | - | - | - | I | A | H | Y | D | D | T | L | Q | L | I | - | H | E | L | L | P | P | D | S | K | L | A | E | D | F | Y | H | S | K | K | L | L | Q | G | L | G | M | T | Y | I | K | I | D | V | C | Y | N | N | C | M | L | Y | Y | K | E | - | N | E | H | K | D | K | C | D | F | Y | N | T | P | R | F | - | - | - | - | - | - | - | - | - | - | - | - | - | - | - | - | - | - | - | - | - | - | - | - | - | - | - | - | - | - | - | - | - | - | - | - | - | - | - | - | - | - | - | - | - | E | D | G | Q | N | K | V | P | R | K | V | L | R | Y | L | P | I | T | D | R | L | Q | R | L | Y | A | H | E | E | T | A | K | A | M | Q | S | H | K | Q | S | - | - | - | - | - | - | - | - | - | - | - | - | - | - | - | T | C | G | K | - | - | - | - | - | L | V | H | P | C | D | G | E | A | W | Q | Q | F | D | I | D | F | P | T | - | F | G | R | D | A | R | N | V | R | L | A | I | S | T | D | G | F | T | P | F | N | I | G | A | A | P | Y | S | C | W | P | V | F | V | S | P | L | N | L | - | P | P | G | V | L | L | K | P | E | Y | I | F | L | S | L | V | I | S | G | P | E | H | P | G | K | K | L | - | S | I | L | M | Q | P | L | V | D | D | L | F | K | L | W | E | - | - | G | V | E | T | W | D | A | S | R | K | E | R | F | N | M | R | A | A | F | L | W | S | V | H | D | C | P | A | Y | G | N | F | A | A | W | S | T | H | G | I | F | G | - | - | C | P | T | C | L | C | D | T | Q | A | F | R | L | R | N | G | G | K | A | C | W | F | D | C | H | R | R | F | L | P | R | D | H | E | F | R | - | - | F | Q | A | N | A | F | R | K | N | T | V | V | L | D | E | - | A | P | R | I | L | K | - | G | E | E | V | R | - | D | Q | M | Y | A | H | V | A | D | R | - | - | - | - | - | - | - | - | - | - | - | - | - | - | - | - | - | - | - | - | - | - | - | - | - | - | - | - | - | - | - | - | - | - | - | - | - | - | - | - | - | - | - | - | - | - | - | - | - | - | N | N | Y | - | G | K | T | H | N | W | T | H | V | N | C | F | W | Q | - | L | P | Y | F | H | - | - | K | L | L | L | P | H | N | I | D | V | M | H | N | E | K | N | V | A | E | A | I | W | N | T | C | F | D | I | P | D | K | T | K | D | N | V | K | A | R | Q | D | L | V | D | I | C | N | R | P | S | L | H | L | E | L | K | G | - | - | - | - | - | - | - | - | - | - | N | G | K | W | H | K | P | R | A | A | F | C | I | D | K | N | D | K | T | I | I | L | K | W | F | - | Q | D | L | K | F | P | D | G | Y | A | A | N | I | R | R | G | V | N | L | L | R | R | R | I | F | G | - | L | K | S | H | D | Y | H | I | F | I | E | R | L | L | P | V | A | F | R | G | F | - | - | - | - | - | - | - | L | P | E | - | N | I | W | V | C | L | A | - | - | - | - | - | - | - | - | - | - | - | - | - | - | - | - | - | - | - | - | - | - | - | E | L | S | F | F | Y | R | Q | L | C | A | K | E | L | S | K | D | V | V | H | S | L | E | Q | N | V | A | V | L | L | C | K | L | E | K | I | F | P | P | G | - | - | - | - | F | F | N | L | M | Q | H | L | I | V | H | L | P | R | E | A | H | L | G | G | P | V | L | A | R | W | M | Y | P | Y | E | - | - | - | - | - | - | - | - | - | - | - | - | - | - | - | - | - | - | - | - | - | - | - | - | - | - | - | - | - | R | Y | - | - | - | - | - | - | - | - | - | - | - | - | - | - | - | - | - | - | - | - | - | - | - | - | - | - | - | - | - | - | - | - | - | - | - | - | - | - | - | - | - | - | - | - | - | - | - | - | - | - | - | - | - | - | - | - | - | - | - | - | - | - | - | - | - | - | - | - | - | - | - | - | - | - | I | G | R | L | R | K | K | V | R | N | K | A | R | V | E | G | S | I | V | E | A | Y | L | V | E | E | N | T | N | - | - | - | - | F | L | S | L | Y | F | S | H | N | V | R | - | - | S | T | R | N | K | I | P | R | Y | D | D | G | S | S | T | F | E | - | - | - | S | S | C | N | L | Q | I | F | Q | Y | P | G | R | C | F | N | R | R | T | C | R | D | L | T | - | - | - | - | V | E | E | Y | K | A | A | F | L | Y | I | L | T | N | I | S | E | M | D | T | - | L | - | - | Q | F | D | K | E | Q | W | K | S | R | I | - | - | - | - | - | - | - | - | - | - | - | - | - | - | - | - | - | - | - | - | - | - | - | - | - | - | - | - | - | - | - | - | - | - | - | - | - | - | - | - | - | - | - | - | - | - | - | - | - | - | - | - | - | - | - | - | - | - | - | - | - | - | - | - | - | - | - | - | - | - | - | - | - | - | - | - | - | - | - | - | - | - | - | - | A | P | S | D | K | Q | R | H | D | L | R | L | N | G | W | K | Y | Y | R | G | T | K | H | G | P | N | F | F | D | W | F | K | K | Q | C | M | A | - | - | - | - | - | - | - | - | - | - | - | - | - | - | - | - | - | - | - | - | - | - | - | - | - | - | - | - | - | - | - | - | - | - | N | S | S | I | D | S | T | L | R | Q | I | S | Y | G | F | P | R | R | I | T | T | Y | G | C | Y | D | V | N | G | Y | R | F | R | S | E | K | Y | E | S | A | - | K | S | G | L | S | T | V | N | T | G | V | C | V | S | C | V | D | D | D | N | N | V | - | - | - | - | - | - | - | - | - | - | I | D | Y | F | G | V | I | E | D | I | I | K | I | T | W | - | - | - | - | - | E | G | S | M | N | L | E | L | V | L | F | D | - | C | R | W | F | D | - | - | - | - | - | - | - | - | - | - | - | - | P | T | N | S | G | V | R | R | T | E | N | L | - | G | L | V | E | - | - | - | - | - | - | - | - | - | - | - | V | N | H | - | - | - | - | - | T | S | R | L | - | - | S | N | F | E | P | F | V | L | A | S | Q | V | T | Q | V | Y | Y | L | P | Y | P | - | - | C | D | S | R | P | D | L | - | K | D | - | - | - | - | - | - | - | - | - | - | W | W | V | V | H | N | V | T | P | H | G | W | L | P | - | - | P | - | - | - | S | T | T | N | E | S | A | P | - | - | - | P | N | D | Q | P | Q | I | D | T | F | Y | Q | E | - | E | G | L | - | - | - | - | - | E | G | H | F | V | I | D | L | G | D | D | I | E | I | A | T | T | Y | I | S | D | E | - | - | - | - | - | - | - | - | - | - | - | - | - | - | - | - | - | - | - | - | - | - | - | - | - | - | - | - | - | - | - | - | - | - | - | - | - | - | - | - | - | - | - | - | - | - | - | - | - | - | - | - | - | - | - | - | - | - | - | - | - | - | - | - | - | - | - | - | - | - | - | - | - | - | - | - | - | - | - | - | - | - | - | - | - | - | - | - | - | - | - | - | - | - | - | - | - | - | - | - | - | - | - | - | - | - | - | - | - | - | - | - | - | - | - | - | - | - | - | - | - | - | - | - | - | - | - | - | - | - | - | - | - | - | - | - | - | - | - | - | - | - | - | - | - | - | - | - | - | - | - | - | - | - | - | - | - | - | - | - | - | - | - | - | - | - | - | - | - | - | - | - | - | - | - | - | - | - | - | - | - | - | - | - | - | - | - | - | - | - | - | - | - | - | - | - | - | - | - | - | - | - | - | - | - | - | - | - | - | - | - | - | - | - | - | - | - | - | - | - | - | - | - | - | - | - | - | - | - | - | - | - | - | - | - | - | - | - | - | - | - | - | - | - | - | - | - | - | - | - | - | - | - | - | - | - | - | - | - | - | - | - | - | - | - | - | - | - | - | - | - | - | - | - | - | - | - | - | - | - | - | - | - | - | - | - | - | - | - | - | - | - | - | - | - | - | - | - | - | - | - | - | - | - | - | - | - | - | - | - | - | - | - | - | - | - | - | - | - | - | - | - | - | - | - | - | - | - | - | - | - | - | - | - | - | - | - | - | - | - | - | - | - | - | - | - | - | - | - | - | - | - | - | - | - | - | - | - | - | - | - | - | - | - | - | - | - | - | - | - | - | - | - | - | - | - | - | - | - | - | - | - | - | - | - | - | - | - | - | - | - | - | - | - | - | - | - | - | - | - | - | - | - | - | - | - | - | - | - | - | - | - | - | - | - | - | - | - | - | - | - | - | - | - | - | - | - | - | - | - | - | - | - | - | - | - | - | - | - | - | - | - | - | - | - | - | - | - | - | - | - | - | - | - | - | - | - | - | - | - | - | - | - | - | - | - | - | - | - | - | - | - | - | - | - | - | - | - | - | - | - | - | - | - | - | - | - | - | - | - | - | - | - | - | - | - | - | - | - | - | - | - | - | - | - | - | - | - | - | I | T | N | E | K | D | L | D | Y | L | A | K | C | N | S | P | T | Q | G | D | E | E | L | D | D | E | E | D | D | E | E | D | E | E | E | A | E | P | E | A | I | - | - | - | - | - | - | - | - | - | - | - | - | - | - | - | - | - | - | - | - | - | - | - | - | - | - | - | - | - | - | - | - | - | - | - | - | - | - | - | - | - | - | - | - | - | - | - | - | - | - | - | - | - | - | - | - | - | - | - | - | - | - | - | - | - | - | - | - | - | - | - | - | - | - | - | - | - | - | - | - | - | - | - | - | - | - | - | - | - | - | - | - | - | - | - | - | - | - | - | - | - | - | - | - | - | - | - | - | - | - | - | - | - | - | - | - | - | - | - | - | - | - | - | - | - | - | - | - | - | - | - | - | - | - | - | - | - | - | - | - | - | - | - | - | - | - | - | - | - | - | - | - | - | - | - | - | - | - | - | - | - | - | - | - | - | - | - | - | - | - | - | - | - | - | - | - | - | - | - | - | - | - | - | - | - | - | - | - | - | - | - | - | - | - | - | - | - | - | - | - | - | - | - | - | - | - | - | - | - | - | - | - | - | - | - | - | - | - | - | - | - | - | - | - | - | - | - | - | - | - | - | - | - | - | - | - | - | - | - | - | - | I | Y | D | P | E | D | F |
|  | C | - | - | - | - | - | - | - | - | - | - | - | - | - | - | - | - | - | M | E | D | R | - | E | W | M | Y | S | G | W | S | R | - | - | R | S | I | S | T | E | W | V | E | Q | T | K | L | F | L | D | R | A | F | S | L | P | S | L | - | V | E | - | N | G | T | I | N | S | P | C | M | K | C | R | N | D | Y | Q | H | I | - | R | - | - | - | - | V | D | I | E | L | H | L | A | K | C | R | F | M | E | N | Y | T | T | - | - | - | W | S | A | H | - | G | E | - | - | T | P | I | V | N | D | E | N | D | V | D | M | - | - | - | - | - | - | - | - | - | - | - | - | - | - | - | - | - | - | - | - | - | - | - | - | - | - | - | - | - | - | - | - | - | - | - | - | - | - | - | - | - | - | - | - | - | - | - | - | - | - | - | - | G | Y | A | N | D | V | V | D | R | M | N | D | M | L | D | D | L | A | A | E | H | T | P | H | I | - | - | - | - | - | - | - | - | - | - | - | - | - | - | - | - | - | - | - | - | - | - | - | - | - | - | - | - | - | - | - | - | V | N | E | E | P | T | - | - | - | - | - | - | - | - | - | - | - | P | Y | A | K | A | F | Y | R | M | L | D | T | V | D | D | K | V | H | - | - | - | A | N | T | - | - | Q | H | T | S | L | S | T | V | A | R | M | L | D | L | N | R | R | Y | N | M | S | - | - | - | - | - | T | A | H | Y | D | D | T | L | K | L | I | - | H | E | L | L | P | P | D | Y | N | L | V | E | D | F | Y | H | S | K | K | L | L | E | G | L | G | M | P | Y | V | K | I | D | V | C | Y | N | N | C | M | L | Y | Y | K | E | - | N | E | H | K | D | I | C | D | F | C | K | T | P | R | Y | - | - | - | - | - | - | - | - | - | - | - | - | - | - | - | - | - | - | - | - | - | - | - | - | - | - | - | - | - | - | - | - | - | - | - | - | - | - | - | - | - | - | - | - | - | E | N | D | I | N | K | V | P | R | K | I | L | R | Y | L | P | I | T | D | R | L | Q | R | L | Y | A | H | V | E | V | A | K | L | M | K | S | H | K | Q | S | - | - | - | - | - | - | - | - | - | - | - | - | - | - | - | T | L | G | K | - | - | - | - | - | M | V | H | P | C | D | G | E | A | W | K | K | F | D | E | D | F | E | V | - | F | A | S | D | P | R | S | V | R | L | A | I | A | T | D | G | F | T | P | F | N | L | T | A | A | S | Y | S | C | W | L | V | F | V | A | P | L | N | L | - | P | P | G | - | S | M | K | K | E | Y | V | F | L | S | M | V | I | S | G | P | E | D | P | G | K | K | L | S | S | V | L | M | Q | P | L | V | D | E | L | M | K | L | W | V | - | - | G | V | D | T | W | D | A | S | V | K | K | K | F | T | M | K | A | A | Y | L | W | S | I | H | D | F | P | A | Y | G | N | F | S | G | W | S | T | H | G | R | L | A | - | - | C | P | I | C | F | S | D | T | K | A | F | R | L | K | A | G | G | K | A | C | W | F | D | C | H | R | R | Y | L | P | E | N | H | V | F | R | - | - | N | Q | E | N | A | F | K | K | A | T | T | I | H | D | I | - | A | P | R | P | L | T | - | G | A | E | V | E | - | H | Q | M | N | R | L | V | G | D | K | - | - | - | - | - | - | - | - | - | - | - | - | - | - | - | - | - | - | - | - | - | - | - | - | - | - | - | - | - | - | - | - | - | - | - | - | - | - | - | - | - | - | - | - | - | - | - | - | - | - | K | G | Y | - | N | S | T | Q | N | W | S | H | A | S | C | F | W | Q | - | L | P | Y | V | N | - | - | K | L | L | L | R | H | N | I | D | V | M | H | N | E | K | N | V | V | E | A | I | W | N | T | C | F | D | V | T | D | K | S | K | D | N | W | K | A | M | L | D | L | Q | Q | I | C | H | R | P | A | L | H | L | S | D | P | T | N | - | - | - | - | - | - | - | - | - | N | G | K | W | E | K | P | R | A | K | F | C | I | K | K | D | D | K | A | I | V | L | E | W | F | - | Q | K | L | K | F | P | D | V | Y | A | A | N | I | K | C | G | V | N | L | K | K | S | K | I | F | G | - | L | K | S | H | D | F | H | I | F | I | E | C | L | L | P | V | A | F | R | G | F | - | - | - | - | - | - | - | L | P | E | - | D | I | W | I | C | L | A | - | - | - | - | - | - | - | - | - | - | - | - | - | - | - | - | - | - | - | - | - | - | - | E | L | S | H | F | Y | R | Q | L | C | A | K | E | L | S | Q | D | I | V | H | S | L | E | Q | N | V | A | A | L | V | C | K | L | E | K | I | F | P | P | G | - | - | - | - | F | F | N | P | M | Q | H | L | I | V | H | L | P | Y | E | V | R | L | G | G | - | - | - | - | - | - | - | - | - | - | - | - | - | - | - | - | - | - | - | - | - | - | - | - | - | - | - | - | - | - | - | - | - | - | - | - | - | - | - | - | - | - | - | - | - | - | - | - | - | - | - | - | - | - | - | - | - | - | - | - | - | - | - | - | - | - | - | - | - | - | - | - | - | - | - | - | - | - | - | - | - | - | - | - | - | - | - | - | - | - | - | - | - | - | - | - | - | - | - | - | - | - | - | - | - | - | - | - | - | - | - | - | - | - | - | - | - | - | - | - | L | K | K | V | R | N | K | A | R | V | E | A | S | I | V | E | A | Y | L | V | E | E | A | T | L | - | - | - | - | F | L | S | L | Y | F | K | A | G | V | R | - | - | S | A | R | N | K | T | P | R | Y | D | E | N | G | P | S | N | D | - | - | - | S | S | C | T | I | D | I | F | Q | Y | Q | G | R | Y | T | N | P | Q | G | S | R | I | L | K | - | - | - | - | P | D | E | Y | K | A | A | A | L | Y | I | L | T | N | I | P | E | M | D | E | - | F | F | V | - | - | - | - | - | - | - | - | - | - | - | - | - | - | - | - | - | - | - | - | - | - | - | - | - | - | - | - | - | - | - | - | - | - | - | - | - | - | - | - | - | - | - | - | - | - | - | - | - | - | - | - | - | - | - | - | - | - | - | - | - | - | - | - | - | - | - | - | - | - | - | - | - | - | - | - | - | - | - | - | - | - | - | - | - | - | - | - | - | - | - | - | - | - | - | - | - | - | - | - | - | - | - | - | - | - | - | - | - | - | - | - | - | - | - | - | - | - | - | - | - | - | - | - | - | - | - | - | - | - | - | - | - | - | - | - | - | - | - | - | - | - | - | - | - | - | - | - | - | - | - | - | - | - | - | - | - | - | - | - | - | - | - | - | - | - | - | - | - | - | - | - | - | - | - | - | - | - | - | - | - | - | - | - | - | - | - | - | - | - | - | - | - | - | - | - | - | - | - | - | - | - | - | - | - | - | - | - | - | - | - | - | - | - | - | - | - | - | - | - | - | - | - | - | - | - | - | - | - | - | - | - | - | - | - | - | - | - | - | - | - | - | - | - | - | - | - | - | - | - | - | - | - | - | - | - | - | - | - | - | - | - | - | - | - | - | - | - | - | - | - | - | - | - | - | - | - | - | - | - | - | - | - | - | - | - | - | - | - | - | - | - | - | - | - | - | - | - | - | - | - | - | - | - | - | - | - | - | - | - | - | - | - | - | - | - | - | - | - | - | - | - | - | - | - | - | - | - | - | - | - | - | - | - | - | - | - | - | - | - | - | - | - | - | - | - | - | - | - | - | - | - | - | - | - | - | - | - | - | - | - | - | - | - | - | - | - | - | - | - | - | - | - | - | - | - | - | - | - | - | - | - | - | - | - | - | - | - | - | - | - | - | - | - | - | - | - | - | - | - | - | - | - | - | - | - | - | - | - | - | - | - | - | - | - | - | - | - | - | - | - | - | - | - | - | - | - | - | - | - | - | - | - | - | - | - | - | - | - | - | - | - | - | - | - | - | - | - | - | - | - | - | - | - | - | - | - | - | - | - | - | - | - | - | - | - | - | - | - | - | - | - | - | - | - | - | - | - | - | - | - | - | - | - | - | - | - | - | - | - | - | - | - | - | - | - | - | - | - | - | - | - | - | - | - | - | - | - | - | - | - | - | - | - | - | - | - | - | - | - | - | - | - | - | - | - | - | - | - | - | - | - | - | - | - | - | - | - | - | - | - | - | - | - | - | - | - | - | - | - | - | - | - | - | - | - | - | - | - | - | - | - | - | - | - | - | - | - | - | - | - | - | - | - | - | - | - | - | - | - | - | - | - | - | - | - | - | - | - | - | - | - | - | - | - | - | - | - | - | - | - | - | - | - | - | - | - | - | - | - | - | - | - | - | - | - | - | - | - | - | - | - | - | - | - | - | - | - | - | - | - | - | - | - | - | - | - | - | - | - | - | - | - | - | - | - | - | - | - | - | - | - | - | - | - | - | - | - | - | - | - | - | - | - | - | - | - | - | - | - | - | - | - | - | - | - | - | - | - | - | - | - | - | - | - | - | - | - | - | - | - | - | - | - | - | - | - | - | - | - | - | - | - | - | - | - | - | - | - | - | - | - | - | - | - | - | - | - | - | - | - | - | - | - | - | - | - | - | - | - | - | - | - | - | - | - | - | - | - | - | - | - | - | - | - | - | - | - | - | - | - | - | - | - | - | - | - | - | - | - | - | - | - | - | - | - | - | - | - | - | - | - | - | - | - | - | - | - | - | - | - | - | - | - | - | - | - | - | - | - | - | - | - | - | - | - | - | - | - | - | - | - | - | - | - | - | - | - | - | - | - | - | - | - | - | - | - | - | - | - | - | - | - | - | - | - | - | - | - | - | - | - | - | - | - | - | - | - | - | - | - | - | - | - | - | - | - | - | - | - | - | - | - | - | - | - | - | - | - | - | - | - | - | - | - | - | - | - | - | - | - | - | - | - | - | - | - | - | - | - | - | - | - | - | - | - | - | - | - | - | - | - | - | - | - | - | - | - | - | - | - | - | - | - | - | - | - | - | - | - | - | - | - | - | - | - | - | - | - | - | - | - | - | - | - | - | - | - | - | - | - | - | - | - | - | - | - | - | - | - | - | - | - | - | - | - | - | - | - | - | - | - | - | - | - | - | - | - | - | - | - | - | - | - | - | - | - | - | - | - | - | - | - | - | - | - | - | - | - | - | - | - | - | - | - | - | - | - | - | - | - | - | - | - | - | - | - | - | - | - | - | - | - | - | - | - | - | - | - | - | - | - | - | - | - | - | - | - | - | - | - | - | - | - | - | - | - | - | - | - | - | - | - | - | - | - | - | - | - | - | - | - | - | - | - | - | - | - | - | - | - | - | - | - | - | - | - | - | - | - | - | - | - | - | - | - | - | - | - | - | - | - | - | - | - | - | - | - | - | - | - | - | - | - | - | - | - | - | - | - | - | - | - | - | - | - | - | - | - | - | - | - | - | - | - | - | - | - | - | - | - | - | - | - | - | - | - | - | - | - | - | - | - | - | - | - | - | - | - | - | - | - | - | - | - | - | - | - | - | - | - | - | - | - | - | - | - | - | - | - | - | - | - | - | - | - | - | - | - | - | - | - | - | - | - | - | - | - | - | - | - | - | - | - | - | - | - | - | - | - | - | - | - | - | - | - | - | - | - | - | - | - | - | - | - | - | - | - | - | - | - | - | - | - | - | - | - | - | - | - | - | - | - | - | - | - | - | - | - | - | - | - | - | - | - | - | - | - | - | - | - | - | - | - | - | - | - | - | - | - | - | - | - | - | - | - | - |
|  | DOPPIA | - | - | - | - | - | - | - | - | - | - | - | - | - | - | - | - | M | D | G | D | R | - | R | V | M | Y | D | G | W | R | K | - | D | G | A | H | S | N | A | W | M | G | V | T | K | A | F | L | E | H | A | F | K | D | A | T | - | - | - | - | - | G | R | L | A | K | C | P | C | N | R | C | E | N | K | W | P | Q | K | - | K | - | - | - | - | E | E | M | E | K | H | L | C | K | S | G | F | M | P | N | Y | L | V | - | - | - | W | Y | Q | H | - | G | E | - | - | S | I | R | H | V | D | A | E | - | - | - | - | - | - | - | - | - | - | - | - | - | - | - | - | - | - | - | - | - | - | - | - | - | - | - | - | - | - | - | - | - | - | - | - | - | - | - | - | - | - | - | - | - | - | - | - | - | - | - | - | - | - | - | - | V | E | L | D | D | D | H | D | R | M | D | D | M | L | H | D | L | G | R | E | V | E | M | N | A | E | - | - | - | - | - | - | - | - | - | - | - | - | - | - | - | - | - | - | - | - | - | - | - | - | - | - | - | - | - | - | - | E | A | G | Q | L | P | - | - | - | - | - | - | - | - | - | - | - | R | D | A | Q | E | F | F | R | L | L | A | A | G | E | E | R | L | H | - | - | - | E | H | T | - | - | P | M | S | V | L | G | T | L | T | R | L | M | A | I | K | S | K | H | N | I | S | - | - | - | - | - | N | S | A | Y | N | D | I | V | Q | L | M | - | G | E | V | L | P | E | N | H | K | L | P | K | N | M | Y | F | A | K | K | M | L | A | G | L | G | M | T | Y | E | K | I | D | V | C | P | N | S | C | M | L | F | F | E | E | - | D | D | K | L | D | R | C | K | H | C | E | A | S | R | Y | V | E | V | T | N | D | E | G | - | - | - | - | - | - | - | - | - | - | - | - | - | - | - | - | - | - | - | - | - | - | - | - | - | - | - | - | - | - | - | - | - | - | - | - | - | E | L | V | V | T | K | V | A | A | K | Q | L | R | R | L | P | I | I | P | R | L | S | R | L | F | L | N | K | E | I | A | L | H | M | T | W | P | K | N | G | - | - | - | - | - | - | - | - | - | - | V | R | L | V | T | D | P | D | I | - | - | - | - | - | M | V | H | P | S | D | G | D | A | W | K | A | F | D | E | F | D | P | E | - | F | A | N | D | P | R | S | V | R | L | G | L | S | T | D | G | F | T | P | F | N | T | S | A | S | P | Y | S | C | W | P | V | F | I | V | P | Y | N | L | - | P | P | E | L | V | N | K | E | E | F | M | F | L | A | L | V | I | P | G | P | E | H | P | G | P | K | L | - | N | M | F | V | R | P | L | I | E | E | L | K | Q | L | W | - | - | R | G | V | K | A | Y | D | S | H | T | E | K | E | F | T | M | R | A | A | Y | L | W | S | V | H | D | L | L | A | Y | G | D | W | S | G | W | C | V | H | G | R | L | C | - | - | C | P | I | C | M | N | D | T | D | A | F | R | L | K | H | G | G | K | V | S | F | F | D | A | H | R | R | W | T | P | F | K | H | D | F | R | - | - | N | S | L | T | A | F | R | G | G | A | K | I | R | N | G | - | P | P | K | R | Q | T | - | A | P | Q | I | M | - | A | W | H | A | C | L | K | Q | G | - | - | - | - | - | - | - | - | - | - | - | - | - | - | - | - | - | - | - | - | - | - | - | - | - | - | - | - | - | - | - | - | - | - | - | - | - | - | - | - | - | - | - | - | - | - | E | N | D | R | F | Q | G | Y | - | G | E | D | H | N | W | T | H | I | S | S | I | W | E | - | L | P | Y | A | K | - | - | A | L | I | M | P | H | N | I | D | L | M | H | Q | E | R | N | V | A | E | S | I | I | S | T | C | F | D | V | T | D | K | T | K | D | N | M | K | A | R | K | D | M | A | E | I | C | K | R | P | M | L | E | L | K | V | S | D | - | - | - | - | - | - | - | - | - | - | K | G | H | E | S | R | P | R | A | D | Y | C | L | K | P | D | E | R | K | E | I | F | K | W | L | - | K | N | L | K | F | P | D | R | Y | A | A | N | L | K | R | A | V | N | L | K | T | G | K | L | I | G | - | L | K | S | H | D | Y | H | I | I | M | E | R | L | M | P | V | M | F | R | G | Y | - | - | - | - | - | - | - | F | K | D | - | E | L | W | S | I | F | A | - | - | - | - | - | - | - | - | - | - | - | - | - | - | - | - | - | - | - | - | - | - | - | E | L | S | Y | F | Y | R | E | V | C | A | K | T | V | S | K | R | L | M | Q | K | F | E | K | E | I | P | I | L | I | C | K | F | E | K | V | F | P | P | G | - | - | - | - | F | F | N | V | M | Q | H | L | I | V | H | L | P | Y | E | A | L | V | G | G | P | V | Q | F | R | W | M | Y | P | I | E | - | - | - | - | - | - | - | - | - | - | - | - | - | - | - | - | - | - | - | - | - | - | - | - | - | - | - | - | - | R | A | - | - | - | - | - | - | - | - | - | - | - | - | - | - | - | - | - | - | - | - | - | - | - | - | - | - | - | - | - | - | - | - | - | - | - | - | - | - | - | - | - | - | - | - | - | - | - | - | - | - | - | - | - | - | - | - | - | - | - | - | - | - | - | - | - | - | - | - | - | - | - | - | - | - | L | K | K | L | R | A | S | V | R | N | K | A | R | V | E | G | C | I | A | E | A | F | A | L | K | E | I | S | Q | - | - | - | - | F | S | T | R | Y | F | A | - | R | A | N | - | - | N | V | F | A | P | S | V | R | L | H | V | D | N | E | S | P | Q | - | - | - | - | - | S | T | L | Q | I | F | A | N | P | G | K | A | V | G | K | G | S | V | R | H | I | E | - | - | - | - | A | S | D | L | N | T | L | M | L | Y | M | Y | S | N | I | D | S | T | Q | E | - | A | F | D | M | F | D | E | E | C | W | K | S | T | S | - | - | - | - | - | - | - | - | - | - | - | - | - | - | - | - | - | - | - | - | - | - | - | - | - | - | - | - | - | - | - | - | - | - | - | - | - | - | - | - | - | - | - | - | - | - | - | - | - | - | - | - | - | - | - | - | - | - | - | - | - | - | - | - | - | - | - | - | - | - | - | - | - | - | - | - | - | - | - | - | - | - | - | - | K | P | T | A | I | Q | L | E | N | L | R | R | D | G | - | - | - | - | - | - | L | K | G | G | P | N | F | V | Q | W | F | R | N | Y | - | - | - | - | - | - | - | - | - | - | - | - | - | - | - | - | - | - | - | - | - | - | - | - | - | - | - | - | - | - | - | - | - | - | - | - | - | - | V | S | N | C | R | S | L | I | Q | M | A | H | T | - | S | V | S | V | R | N | Y | T | R | Y | D | V | N | G | Y | R | F | R | T | A | K | L | E | K | S | - | R | P | L | A | A | T | T | N | S | G | V | L | A | S | S | Y | V | D | D | D | K | V | - | - | - | - | - | - | - | - | - | - | V | D | Y | Y | G | V | L | Q | N | I | V | E | L | I | F | - | - | - | - | - | D | G | P | K | E | L | K | V | V | F | F | E | - | C | D | W | F | D | - | - | - | - | - | - | - | - | - | - | - | - | A | H | S | G | - | - | T | R | V | D | K | Y | - | G | N | V | E | - | - | - | - | - | - | - | - | - | - | - | V | K | H | - | - | - | - | - | S | S | R | I | L | - | S | S | M | S | D | V | V | L | A | N | Q | A | K | Q | V | Y | Y | L | P | Y | P | - | - | H | P | S | L | K | A | - | - | - | - | - | - | - | - | - | - | - | - | - | - | W | W | V | A | I | K | V | N | P | Q | V | V | A | P | - | - | - | - | - | - | E | S | A | D | Y | V | - | - | - | - | - | S | T | S | R | D | N | D | D | A | V | F | Q | P | - | E | A | A | S | N | Q | D | I | A | H | R | F | H | V | T | N | G | E | G | L | E | N | L | C | C | N | S | S | D | L | I | E | E | P | R | S | K | R | K | R | P | V | - | - | - | - | - | - | - | - | - | - | - | - | - | - | - | - | - | - | - | - | - | - | - | - | - | - | - | - | - | - | - | - | - | - | - | - | - | - | - | - | - | - | - | - | - | - | - | - | - | - | - | - | - | - | - | - | - | - | - | - | - | - | - | - | - | - | - | - | - | - | - | - | - | - | - | - | - | - | - | - | - | - | - | - | - | - | - | - | - | - | - | - | - | - | - | - | - | - | - | - | - | - | - | - | - | - | - | - | - | - | - | - | - | - | - | - | - | - | - | - | - | - | - | - | - | - | - | - | - | - | - | - | - | - | - | - | - | - | - | - | - | - | - | - | - | - | - | - | - | - | - | - | - | - | - | - | - | - | - | - | - | - | - | - | - | - | - | - | - | - | - | - | - | - | - | - | - | - | - | - | - | - | - | - | - | - | - | - | - | - | - | - | - | - | - | - | - | - | - | - | - | - | - | - | - | - | - | - | - | - | - | - | - | - | - | - | - | - | - | - | - | - | - | - | - | - | - | - | - | - | - | - | - | - | - | - | - | - | - | - | - | - | - | - | - | - | - | - | - | - | - | - | - | - | - | - | - | - | - | - | - | - | - | - | - | - | - | - | - | - | - | - | - | - | - | - | - | - | - | - | - | - | - | - | - | - | - | - | - | - | - | - | - | - | - | - | - | - | - | - | - | - | - | - | - | - | - | - | - | - | - | - | - | - | - | - | - | - | - | - | - | - | - | - | - | - | - | - | - | - | - | - | - | - | - | - | - | - | - | - | - | - | - | - | - | - | - | - | - | - | - | - | - | - | - | - | - | - | - | - | - | - | - | - | - | - | - | - | - | - | - | - | - | - | - | - | - | - | - | - | - | - | - | - | - | - | - | - | - | - | - | - | - | - | - | - | - | - | - | - | - | - | - | - | - | - | - | - | - | - | - | - | - | - | - | - | - | - | - | - | - | - | - | - | - | - | - | - | - | - | - | - | - | - | - | - | - | - | - | - | - | - | - | - | - | - | - | - | - | - | - | - | - | - | - | - | - | - | - | - | - | - | - | - | - | - | - | - | - | - | - | - | - | - | - | - | - | - | - | - | - | - | - | - | - | - | - | - | - | - | - | - | V | V | R | R | S | V | R | I | Q | R | N | Q | E | R | M | N | K | Q | R | A | E | E | A | S | S | D | A | D | D | F | - | - | - | - | - | - | - | - | - | - | - | - | - | - | - | - | - | - | - | - | - | - | - | - | - | - | - | - | - | - | - | - | - | - | - | - | - | - | - | - | - | - | - | - | - | - | - | - | - | - | - | - | - | - | - | - | - | - | - | - | - | - | - | - | - | - | - | - | - | - | - | - | - | - | - | - | - | - | - | - | - | - | - | - | - | - | - | - | - | - | - | - | - | - | - | - | - | - | - | - | - | - | - | - | - | - | - | - | - | - | - | - | - | - | - | - | - | - | - | - | - | - | - | - | - | - | - | - | - | - | - | - | - | - | - | - | - | - | - | - | - | - | - | - | - | - | - | - | - | - | - | - | - | - | - | - | - | - | - | - | - | - | - | - | - | - | - | - | - | - | - | - | - | - | - | - | - | - | - | - | - | - | - | - | - | - | - | - | - | - | - | - | - | - | - | - | - | - | - | - | - | - | - | - | - | - | - | - | - | - | - | - | - | - | - | - | - | - | - | - | - | - | - | - | - | - | - | - | - | - | - | - | - | - | - | - | - | - | - | - | - | - | - | - | - | - | - | - | - | - | - | - | - | - | - | - | - | - | - | - | - | - | - | - |
|  | En1 | - | - | - | - | - | - | - | - | - | - | - | - | - | - | - | - | M | S | D | D | R | R | R | A | M | Y | D | G | F | D | S | V | T | H | G | H | S | D | A | W | L | R | V | A | D | E | F | V | A | L | A | F | V | G | D | - | - | - | - | - | - | A | R | L | A | R | C | P | C | I | K | C | R | N | L | V | R | L | K | - | K | - | - | - | - | V | E | L | S | Y | H | I | F | K | H | G | F | M | P | N | Y | L | V | - | - | - | W | H | E | H | - | G | E | - | - | V | D | H | T | I | E | - | - | - | - | - | - | - | - | - | - | - | - | - | - | - | - | - | - | - | - | - | - | - | - | - | - | - | - | - | - | - | - | - | - | - | - | - | - | - | - | - | - | - | - | - | - | - | - | - | - | - | - | - | - | - | - | - | - | S | D | G | D | Q | D | I | D | R | M | E | E | M | L | D | D | I | R | N | E | Y | P | D | L | Q | - | - | - | - | - | - | - | - | - | - | - | - | - | - | - | - | - | - | - | - | - | - | - | - | - | - | - | - | - | - | - | - | N | N | Q | A | F | P | - | - | - | - | - | - | - | - | - | - | - | E | D | V | R | E | F | Y | K | L | L | E | A | S | E | A | K | V | H | - | - | - | E | G | T | - | - | N | V | S | V | L | Q | V | V | T | R | L | M | A | M | K | S | K | Y | T | F | S | - | - | - | - | - | N | K | C | Y | N | D | I | V | K | L | I | - | I | D | I | S | P | P | N | H | N | M | P | K | D | L | Y | H | C | K | K | L | V | A | G | L | G | M | N | Y | Q | K | I | D | A | C | E | D | N | C | M | L | F | W | K | E | - | H | E | N | T | T | H | C | I | H | C | S | K | S | R | Y | A | V | V | L | D | E | D | G | - | - | - | - | - | - | - | - | - | - | - | - | - | - | - | - | - | - | - | - | - | - | - | - | - | - | - | - | - | - | - | - | - | - | - | - | - | N | E | V | T | T | K | V | P | I | K | Q | L | R | Y | M | P | I | T | P | R | L | K | R | L | F | L | N | Q | E | T | A | K | Q | M | R | W | H | K | E | G | - | - | - | - | - | - | - | - | - | - | D | R | Q | G | Q | D | P | D | V | - | - | - | - | - | M | V | H | P | S | D | G | E | A | W | Q | A | L | D | R | F | D | P | E | - | F | A | R | D | P | R | S | V | R | L | G | L | S | T | D | G | F | T | P | Y | S | N | N | S | T | S | Y | S | C | W | P | V | F | M | M | P | Y | N | L | - | P | P | N | K | C | M | K | E | E | V | M | F | L | A | L | I | V | P | G | P | K | D | P | V | T | K | I | - | N | V | F | M | E | P | L | I | E | E | L | K | M | L | W | - | - | Q | G | V | E | A | Y | D | S | H | L | K | C | C | F | T | L | R | A | A | Y | L | W | S | I | H | D | L | L | A | Y | G | I | F | S | G | W | C | V | H | G | I | L | R | - | - | C | P | I | C | M | G | D | S | Q | A | Y | R | L | E | H | G | K | K | E | T | F | F | D | V | H | R | R | L | L | P | Y | N | H | P | F | R | - | - | K | D | T | K | S | F | R | K | G | K | R | V | R | D | G | - | P | P | K | R | Q | T | - | G | E | N | I | M | - | R | Q | H | R | D | L | K | P | G | V | - | - | - | - | - | - | - | - | - | - | - | - | - | - | - | - | - | - | - | - | - | - | - | - | - | - | - | - | - | - | - | - | - | - | - | - | - | - | - | - | - | - | - | - | - | - | G | G | R | F | Q | G | Y | - | G | K | E | H | N | W | T | H | I | S | F | I | W | E | - | L | P | Y | T | K | - | - | A | L | L | L | P | H | N | I | D | L | M | H | Q | E | R | N | V | A | E | S | I | I | S | M | C | F | D | F | T | G | Q | T | K | D | N | M | N | A | R | R | D | L | A | E | L | C | D | R | P | H | L | E | L | R | K | N | P | - | - | - | - | - | - | - | - | - | - | S | G | S | E | S | R | P | Q | A | P | Y | C | L | K | R | Q | E | R | E | E | I | F | Q | W | L | - | K | K | L | R | F | P | D | R | Y | A | A | N | I | K | R | A | V | N | L | D | T | G | K | L | V | G | - | L | K | S | H | D | Y | H | I | L | I | E | R | L | V | P | V | M | F | R | G | Y | - | - | - | - | - | - | - | F | S | P | - | D | V | W | K | I | F | A | - | - | - | - | - | - | - | - | - | - | - | - | - | - | - | - | - | - | - | - | - | - | - | E | L | S | Y | F | Y | K | Q | I | C | A | K | E | I | S | K | K | L | M | L | R | F | E | K | E | I | V | V | L | V | C | K | M | E | K | V | F | P | P | G | - | - | - | - | F | F | N | C | M | Q | H | L | L | V | H | L | P | W | E | A | L | V | G | G | P | A | Q | F | R | W | M | Y | S | Q | E | - | - | - | - | - | - | - | - | - | - | - | - | - | - | - | - | - | - | - | - | - | - | - | - | - | - | - | - | - | R | E | - | - | - | - | - | - | - | - | - | - | - | - | - | - | - | - | - | - | - | - | - | - | - | - | - | - | - | - | - | - | - | - | - | - | - | - | - | - | - | - | - | - | - | - | - | - | - | - | - | - | - | - | - | - | - | - | - | - | - | - | - | - | - | - | - | - | - | - | - | - | - | - | - | - | L | K | K | L | R | G | M | V | R | N | K | A | R | V | E | G | C | I | A | E | A | F | A | A | R | E | I | T | L | - | - | - | - | F | S | S | K | Y | F | S | - | D | T | N | - | - | N | V | N | A | Q | T | T | R | Y | H | V | A | E | Q | A | P | I | - | - | - | - | - | T | D | L | S | A | F | K | W | D | G | K | G | V | G | A | Y | T | S | H | L | V | G | - | - | - | - | T | I | E | R | N | K | T | L | L | F | L | Y | V | N | M | P | E | L | H | P | - | Y | F | Q | I | F | D | - | S | I | Y | K | P | N | K | - | - | - | - | - | - | - | - | - | - | - | - | - | - | - | - | - | - | - | - | - | - | - | - | - | - | - | - | - | - | - | - | - | - | - | - | - | - | - | - | - | - | - | - | - | - | - | - | - | - | - | - | - | - | - | - | - | - | - | - | - | - | - | - | - | - | - | - | - | - | - | - | - | - | - | - | - | - | - | - | - | - | - | - | Q | L | T | Q | K | Q | L | D | D | L | R | L | K | G | - | - | - | - | - | - | L | H | G | G | P | S | F | V | Q | W | F | H | E | H | - | - | - | - | - | - | - | - | - | - | - | - | - | - | - | - | - | - | - | - | - | - | - | - | - | - | - | - | - | - | - | - | - | - | - | - | - | - | V | I | S | S | K | D | L | L | Q | I | S | H | G | - | Q | L | T | A | R | K | F | D | R | Y | D | I | N | G | Y | R | F | R | T | A | K | L | E | A | S | - | R | P | L | A | A | T | T | N | S | G | V | V | A | S | S | Y | S | D | D | G | Q | L | - | - | - | - | - | - | - | - | - | - | E | D | Y | Y | G | I | V | Q | D | I | T | E | Y | T | F | - | - | - | - | - | G | G | H | K | P | L | R | L | V | T | F | D | - | C | I | W | F | D | - | - | - | - | - | - | - | - | - | - | - | - | P | Q | V | G | - | - | T | R | V | D | E | F | - | G | M | V | E | - | - | - | - | - | - | - | - | - | - | - | V | K | H | - | - | - | - | - | A | S | R | Y | K | G | N | E | Y | N | N | I | I | L | A | H | Q | A | H | Q | V | Y | Y | L | S | Y | P | - | - | H | K | S | F | K | T | - | - | - | - | - | - | - | - | - | - | - | - | - | - | W | W | V | A | Y | K | V | N | P | E | V | H | P | Y | - | - | - | - | - | - | R | Y | Q | N | Y | N | L | S | - | - | - | T | N | D | D | D | E | D | D | V | V | F | Q | E | - | V | G | D | Q | A | D | D | S | D | N | D | S | I | V | S | E | G | A | G | L | N | E | L | A | S | L | T | V | E | L | M | E | E | P | I | S | S | N | S | K | R | Q | - | - | - | - | - | - | - | - | - | - | - | - | - | - | - | - | - | - | - | - | - | - | - | - | - | - | - | - | - | - | - | - | - | - | - | - | - | - | - | - | - | - | - | - | - | - | - | - | - | - | - | - | - | - | - | - | - | - | - | - | - | - | - | - | - | - | - | - | - | - | - | - | - | - | - | - | - | - | - | - | - | - | - | - | - | - | - | - | - | - | - | - | - | - | - | - | - | - | - | - | - | - | - | - | - | - | - | - | - | - | - | - | - | - | - | - | - | - | - | - | - | - | - | - | - | - | - | - | - | - | - | - | - | - | - | - | - | - | - | - | - | - | - | - | - | - | - | - | - | - | - | - | - | - | - | - | - | - | - | - | - | - | - | - | - | - | - | - | - | - | - | - | - | - | - | - | - | - | - | - | - | - | - | - | - | - | - | - | - | - | - | - | - | - | - | - | - | - | - | - | - | - | - | - | - | - | - | - | - | - | - | - | - | - | - | - | - | - | - | - | - | - | - | - | - | - | - | - | - | - | - | - | - | - | - | - | - | - | - | - | - | - | - | - | - | - | - | - | - | - | - | - | - | - | - | - | - | - | - | - | - | - | - | - | - | - | - | - | - | - | - | - | - | - | - | - | - | - | - | - | - | - | - | - | - | - | - | - | - | - | - | - | - | - | - | - | - | - | - | - | - | - | - | - | - | - | - | - | - | - | - | - | - | - | - | - | - | - | - | - | - | - | - | - | - | - | - | - | - | - | - | - | - | - | - | - | - | - | - | - | - | - | - | - | - | - | - | - | - | - | - | - | - | - | - | - | - | - | - | - | - | - | - | - | - | - | - | - | - | - | - | - | - | - | - | - | - | - | - | - | - | - | - | - | - | - | - | - | - | - | - | - | - | - | - | - | - | - | - | - | - | - | - | - | - | - | - | - | - | - | - | - | - | - | - | - | - | - | - | - | - | - | - | - | - | - | - | - | - | - | - | - | - | - | - | - | - | - | - | - | - | - | - | - | - | - | - | - | - | - | - | - | - | - | - | - | - | - | - | - | - | - | - | - | - | - | - | - | - | - | - | - | - | - | - | - | - | - | - | - | - | - | - | - | - | - | - | - | - | - | - | - | R | L | E | E | T | V | L | E | T | Q | Q | W | V | E | Q | L | N | T | R | V | A | E | E | D | S | D | A | D | D | F | - | - | - | - | - | - | - | - | - | - | - | - | - | - | - | - | - | - | - | - | - | - | - | - | - | - | - | - | - | - | - | - | - | - | - | - | - | - | - | - | - | - | - | - | - | - | - | - | - | - | - | - | - | - | - | - | - | - | - | - | - | - | - | - | - | - | - | - | - | - | - | - | - | - | - | - | - | - | - | - | - | - | - | - | - | - | - | - | - | - | - | - | - | - | - | - | - | - | - | - | - | - | - | - | - | - | - | - | - | - | - | - | - | - | - | - | - | - | - | - | - | - | - | - | - | - | - | - | - | - | - | - | - | - | - | - | - | - | - | - | - | - | - | - | - | - | - | - | - | - | - | - | - | - | - | - | - | - | - | - | - | - | - | - | - | - | - | - | - | - | - | - | - | - | - | - | - | - | - | - | - | - | - | - | - | - | - | - | - | - | - | - | - | - | - | - | - | - | - | - | - | - | - | - | - | - | - | - | - | - | - | - | - | - | - | - | - | - | - | - | - | - | - | - | - | - | - | - | - | - | - | - | - | - | - | - | - | - | - | - | - | - | - | - | - | - | - | - | - | - | - | - | - | - | - | - | - | - | - | - | - | - | - | - |
|  | Norman | - | - | - | - | - | - | - | - | - | - | - | - | - | - | - | - | M | D | D | N | R | R | R | A | M | Y | D | G | F | N | S | V | T | L | G | H | S | K | E | W | V | R | V | A | K | Q | F | V | D | L | A | F | S | G | G | - | - | - | - | - | - | P | C | V | V | K | C | P | C | T | K | C | R | N | F | K | Y | V | R | K | I | - | - | - | - | E | E | L | E | H | H | L | C | K | N | G | F | M | P | N | Y | L | V | - | - | - | W | R | S | H | - | G | E | - | - | V | E | Q | N | I | T | E | S | E | - | - | - | - | - | - | - | - | - | - | - | - | - | - | - | - | - | - | - | - | - | - | - | - | - | - | - | - | - | - | - | - | - | - | - | - | - | - | - | - | - | - | - | - | - | - | - | - | - | - | - | - | - | - | - | R | I | D | D | E | E | E | D | R | M | D | D | L | V | A | D | I | S | R | E | Y | P | T | L | A | - | - | - | - | - | - | - | - | - | - | - | - | - | - | - | - | - | - | - | - | - | - | - | - | - | - | - | - | - | - | - | - | S | K | Q | A | T | P | - | - | - | - | - | - | - | - | - | - | - | D | E | V | R | E | F | Y | K | L | L | H | A | S | E | E | K | V | H | - | - | - | D | D | T | - | - | T | V | T | V | L | Q | V | V | T | R | F | M | A | M | K | A | K | Y | N | F | S | - | - | - | - | - | N | N | C | Y | N | N | I | I | N | L | I | - | I | D | L | I | P | S | N | H | K | M | P | K | D | L | Y | Q | S | K | K | I | V | P | G | L | G | M | K | Y | K | K | I | D | V | C | E | D | N | C | M | L | F | W | K | E | - | Y | E | A | A | T | N | C | L | K | C | G | K | S | R | Y | P | E | V | L | K | E | D | G | - | - | - | - | - | - | - | - | - | - | - | - | - | - | - | - | - | - | - | - | - | - | - | - | - | - | - | - | - | - | - | - | - | - | - | - | - | A | T | F | A | T | K | V | A | V | K | Q | L | Q | Y | M | P | I | T | P | R | L | K | R | L | F | L | T | P | E | T | A | K | L | M | L | W | H | K | E | G | - | - | - | - | - | - | - | - | - | - | E | R | E | I | Q | D | L | D | I | - | - | - | - | - | M | M | H | P | S | D | G | D | A | W | K | A | L | D | R | F | D | L | E | - | F | A | R | D | A | R | S | V | R | L | G | L | S | T | D | G | F | T | P | Y | D | N | S | S | T | S | Y | S | C | R | P | V | F | I | M | P | Y | N | P | - | P | P | N | K | C | M | K | E | G | F | I | F | L | A | L | I | I | P | G | P | K | H | P | G | K | K | I | - | N | V | F | M | Q | P | L | I | E | E | F | K | E | L | W | - | - | V | G | V | K | A | Y | D | G | H | L | - | V | K | F | T | L | R | A | V | Y | L | W | S | I | H | N | L | P | A | Y | D | I | W | S | G | W | C | V | H | G | R | L | C | - | - | C | P | I | C | M | G | D | T | Q | A | Y | R | L | E | H | G | K | K | V | S | F | F | D | C | H | R | R | F | L | P | S | N | H | L | F | R | - | - | N | D | T | K | S | F | R | R | G | I | K | V | K | D | G | - | P | P | K | R | L | M | - | G | E | D | I | M | - | I | Q | H | R | A | L | Q | R | D | A | - | - | - | - | - | - | - | - | - | - | - | - | - | - | - | - | - | - | - | - | - | - | - | - | - | - | - | - | - | - | - | - | - | - | - | - | - | - | - | - | - | - | - | - | - | E | G | H | E | F | E | G | Y | - | G | Q | D | H | N | W | T | H | I | S | F | L | W | E | - | L | P | Y | A | K | - | - | V | L | I | L | P | H | N | I | D | L | M | H | Q | E | H | N | V | A | E | S | I | I | S | M | C | I | N | I | K | G | K | T | K | D | N | I | N | A | R | K | D | L | A | N | L | C | D | R | P | S | L | E | V | K | L | N | P | - | - | - | - | - | - | - | - | - | - | N | G | K | E | R | T | P | R | A | P | Y | C | L | K | L | E | E | R | K | E | V | F | R | W | L | - | K | M | L | K | F | P | D | R | Y | A | A | N | I | K | R | T | V | N | L | D | T | N | K | L | N | G | - | L | K | A | H | D | Y | H | I | L | M | E | R | L | M | P | V | M | F | R | G | Y | - | - | - | - | - | - | - | L | K | P | - | P | L | W | K | M | I | V | - | - | - | - | - | - | - | - | - | - | - | - | - | - | - | - | - | - | - | - | - | - | - | E | L | S | Y | I | Y | R | H | I | C | A | K | H | I | S | K | K | L | M | I | Q | F | E | K | Q | I | A | A | L | V | C | K | M | E | K | V | F | P | P | G | - | - | - | - | F | M | N | V | M | Q | H | L | L | V | H | L | P | Y | E | A | L | V | G | G | P | V | Q | F | R | W | M | Y | S | Q | E | - | - | - | - | - | - | - | - | - | - | - | - | - | - | - | - | - | - | - | - | - | - | - | - | - | - | - | - | - | R | E | - | - | - | - | - | - | - | - | - | - | - | - | - | - | - | - | - | - | - | - | - | - | - | - | - | - | - | - | - | - | - | - | - | - | - | - | - | - | - | - | - | - | - | - | - | - | - | - | - | - | - | - | - | - | - | - | - | - | - | - | - | - | - | - | - | - | - | - | - | - | - | - | - | - | L | K | K | L | R | A | T | V | R | N | K | A | R | V | E | G | C | I | A | E | A | F | A | A | K | E | I | S | I | - | - | - | - | F | S | S | Q | Y | L | S | - | H | T | N | - | - | N | V | N | A | Q | S | T | R | Y | H | T | E | E | E | G | L | R | - | - | - | - | - | P | T | L | V | F | F | L | W | K | G | K | G | V | G | A | S | T | A | H | Y | V | D | - | - | - | - | I | R | E | R | N | F | T | M | L | Y | L | Y | T | N | M | E | E | L | D | P | - | Y | F | E | M | F | D | - | S | I | Y | L | A | G | H | - | - | - | - | - | - | - | - | - | - | - | - | - | - | - | - | - | - | - | - | - | - | - | - | - | - | - | - | - | - | - | - | - | - | - | - | - | - | - | - | - | - | - | - | - | - | - | - | - | - | - | - | - | - | - | - | - | - | - | - | - | - | - | - | - | - | - | - | - | - | - | - | - | - | - | - | - | - | - | - | - | - | - | - | K | P | T | P | K | E | L | D | N | L | R | M | K | G | - | - | - | - | - | - | M | N | G | G | P | R | F | I | E | W | F | H | E | H | - | - | - | - | - | - | - | - | - | - | - | - | - | - | - | - | - | - | - | - | - | - | - | - | - | - | - | - | - | - | - | - | - | - | - | - | - | - | V | I | I | S | D | D | L | R | H | I | S | H | G | - | Q | L | K | P | R | K | Y | S | R | Y | D | I | N | G | Y | R | I | R | T | S | K | L | E | K | K | I | H | P | Q | A | A | T | T | N | S | G | V | V | A | S | T | T | N | A | D | R | G | T | - | - | - | - | - | - | - | - | - | - | H | D | Y | Y | G | V | L | Q | D | I | T | K | Y | T | L | - | - | - | - | - | G | G | A | K | E | F | M | F | V | L | F | D | - | C | E | W | F | D | - | - | - | - | - | - | - | - | - | - | - | - | P | Q | Q | - | - | - | T | R | E | D | E | F | - | G | I | V | E | - | - | - | - | - | - | - | - | - | - | - | V | K | H | - | - | - | - | - | E | S | R | F | K | G | S | D | Y | S | N | V | V | L | A | H | Q | V | Q | Q | V | Y | Y | L | T | Y | P | - | - | H | E | N | L | K | S | - | - | - | - | - | - | - | - | - | - | - | - | - | - | C | W | V | V | Y | K | I | N | P | E | V | H | P | L | - | - | - | - | - | - | R | Y | D | Y | Y | N | T | N | - | - | - | N | E | D | D | D | Y | V | N | - | I | Y | Q | E | - | E | G | D | Q | S | E | - | - | N | R | E | F | T | I | S | E | D | L | G | L | N | E | V | A | I | L | A | I | D | L | M | A | E | E | P | G | P | S | R | A | R | T | - | - | - | - | - | - | - | - | - | - | - | - | - | - | - | - | - | - | - | - | - | - | - | - | - | - | - | - | - | - | - | - | - | - | - | - | - | - | - | - | - | - | - | - | - | - | - | - | - | - | - | - | - | - | - | - | - | - | - | - | - | - | - | - | - | - | - | - | - | - | - | - | - | - | - | - | - | - | - | - | - | - | - | - | - | - | - | - | - | - | - | - | - | - | - | - | - | - | - | - | - | - | - | - | - | - | - | - | - | - | - | - | - | - | - | - | - | - | - | - | - | - | - | - | - | - | - | - | - | - | - | - | - | - | - | - | - | - | - | - | - | - | - | - | - | - | - | - | - | - | - | - | - | - | - | - | - | - | - | - | - | - | - | - | - | - | - | - | - | - | - | - | - | - | - | - | - | - | - | - | - | - | - | - | - | - | - | - | - | - | - | - | - | - | - | - | - | - | - | - | - | - | - | - | - | - | - | - | - | - | - | - | - | - | - | - | - | - | - | - | - | - | - | - | - | - | - | - | - | - | - | - | - | - | - | - | - | - | - | - | - | - | - | - | - | - | - | - | - | - | - | - | - | - | - | - | - | - | - | - | - | - | - | - | - | - | - | - | - | - | - | - | - | - | - | - | - | - | - | - | - | - | - | - | - | - | - | - | - | - | - | - | - | - | - | - | - | - | - | - | - | - | - | - | - | - | - | - | - | - | - | - | - | - | - | - | - | - | - | - | - | - | - | - | - | - | - | - | - | - | - | - | - | - | - | - | - | - | - | - | - | - | - | - | - | - | - | - | - | - | - | - | - | - | - | - | - | - | - | - | - | - | - | - | - | - | - | - | - | - | - | - | - | - | - | - | - | - | - | - | - | - | - | - | - | - | - | - | - | - | - | - | - | - | - | - | - | - | - | - | - | - | - | - | - | - | - | - | - | - | - | - | - | - | - | - | - | - | - | - | - | - | - | - | - | - | - | - | - | - | - | - | - | - | - | - | - | - | - | - | - | - | - | - | - | - | - | - | - | - | - | - | - | - | - | - | - | - | - | - | - | - | - | - | - | - | - | - | - | - | - | - | - | - | - | - | - | - | - | - | - | - | - | - | - | - | - | - | - | - | - | - | R | R | S | Q | R | I | L | E | K | Q | Q | R | L | E | I | I | E | Q | R | V | A | E | A | D | S | D | A | D | D | F | - | - | - | - | - | - | - | - | - | - | - | - | - | - | - | - | - | - | - | - | - | - | - | - | - | - | - | - | - | - | - | - | - | - | - | - | - | - | - | - | - | - | - | - | - | - | - | - | - | - | - | - | - | - | - | - | - | - | - | - | - | - | - | - | - | - | - | - | - | - | - | - | - | - | - | - | - | - | - | - | - | - | - | - | - | - | - | - | - | - | - | - | - | - | - | - | - | - | - | - | - | - | - | - | - | - | - | - | - | - | - | - | - | - | - | - | - | - | - | - | - | - | - | - | - | - | - | - | - | - | - | - | - | - | - | - | - | - | - | - | - | - | - | - | - | - | - | - | - | - | - | - | - | - | - | - | - | - | - | - | - | - | - | - | - | - | - | - | - | - | - | - | - | - | - | - | - | - | - | - | - | - | - | - | - | - | - | - | - | - | - | - | - | - | - | - | - | - | - | - | - | - | - | - | - | - | - | - | - | - | - | - | - | - | - | - | - | - | - | - | - | - | - | - | - | - | - | - | - | - | - | - | - | - | - | - | - | - | - | - | - | - | - | - | - | - | - | - | - | - | - | - | - | - | - | - | - | - | - | - | - | - | - | - |
|  | Baron | - | - | - | - | - | - | - | - | - | - | - | - | - | - | - | - | - | - | - | - | - | - | - | - | - | - | - | - | - | - | - | - | - | - | - | - | - | - | - | - | - | - | - | - | - | - | - | - | - | - | - | - | - | - | - | - | - | - | - | - | - | - | - | M | I | V | C | P | C | K | D | C | R | N | V | E | R | K | S | - | G | - | - | - | - | S | D | V | V | D | H | L | V | R | R | G | M | D | E | A | Y | K | L | R | A | D | W | Y | H | H | - | G | D | - | - | V | D | S | V | A | D | C | E | S | N | F | S | R | W | N | A | - | - | - | - | - | - | - | - | - | - | - | - | - | - | - | - | - | - | - | - | - | - | - | - | - | - | - | - | - | - | - | - | - | - | - | - | - | - | - | - | - | - | - | - | - | - | - | - | - | - | - | - | - | - | - | - | E | I | L | E | L | Y | Q | A | A | Q | G | F | D | A | D | L | G | E | - | - | - | - | - | - | - | - | - | - | - | - | - | - | - | - | - | - | - | - | - | - | - | - | - | - | - | - | - | - | - | I | A | E | D | E | D | - | - | - | - | - | - | - | - | - | - | - | I | R | E | D | E | F | L | A | K | L | A | D | A | E | I | P | L | Y | - | - | - | P | S | C | L | - | N | H | S | K | L | S | A | I | V | T | L | F | R | I | K | T | K | N | G | W | S | - | - | - | - | - | D | K | S | F | N | E | L | L | G | T | L | - | P | E | M | L | P | A | D | N | V | L | H | T | S | L | Y | E | V | K | K | F | L | R | S | F | D | M | G | Y | E | K | I | H | A | C | V | N | D | C | C | L | F | R | K | R | - | Y | K | N | L | E | N | C | P | K | C | N | A | S | R | W | K | S | N | M | Q | T | - | - | - | - | - | - | - | - | - | - | - | - | - | - | - | - | - | - | - | - | - | - | - | - | - | - | - | - | - | - | - | - | - | - | - | - | - | - | - | G | E | A | K | K | G | V | P | Q | K | V | L | R | Y | F | P | I | I | P | R | F | K | R | M | F | R | S | E | E | M | A | K | D | L | R | W | H | Y | S | N | - | - | - | - | - | - | - | - | - | - | K | S | - | - | - | T | D | G | K | - | - | - | - | - | L | R | H | P | V | D | S | V | T | W | D | K | M | N | D | K | Y | P | E | - | F | A | I | E | E | R | N | L | R | L | G | L | S | T | D | G | F | N | P | F | N | M | K | N | T | R | Y | S | C | W | P | V | L | L | V | N | Y | N | L | - | P | P | D | L | C | M | K | K | E | N | I | M | L | S | L | L | I | P | G | P | Q | Q | P | G | N | S | I | - | D | V | Y | L | E | P | L | I | E | D | L | N | H | L | W | K | - | K | G | E | L | T | Y | D | A | F | S | K | T | T | F | T | L | K | A | M | L | L | W | T | I | S | D | F | P | A | Y | G | N | L | A | G | C | K | V | K | G | K | M | G | - | - | C | P | L | C | G | K | G | T | D | S | M | W | L | K | Y | S | R | K | H | V | F | M | C | - | H | R | K | G | L | A | P | T | H | S | Y | R | - | - | N | K | K | A | W | F | D | G | K | I | E | - | H | G | R | - | K | A | R | I | L | T | - | G | R | E | V | S | - | Q | T | L | K | N | F | K | - | N | D | F | G | N | - | - | - | - | - | - | - | - | - | - | - | - | A | K | E | S | G | R | K | R | K | R | N | - | D | C | I | E | S | V | T | D | S | D | D | E | S | S | E | S | E | E | D | E | E | L | E | V | D | E | - | D | E | L | S | R | W | K | K | R | S | I | F | F | K | - | L | S | Y | W | E | V | N | D | L | P | V | R | H | N | L | D | V | M | H | V | E | R | N | V | G | A | S | I | V | S | T | L | L | H | C | - | G | K | S | K | D | G | L | N | A | R | K | D | L | Q | D | L | G | V | R | K | D | L | H | P | - | - | N | A | Q | - | - | - | - | - | - | - | - | - | G | K | R | T | Y | L | P | A | A | P | W | S | L | S | K | S | E | K | K | T | F | C | K | R | L | - | F | E | F | K | G | P | D | G | Y | C | S | N | I | S | R | G | V | S | L | E | D | C | K | I | M | G | - | L | K | S | H | D | Y | H | V | L | M | Q | Q | L | L | P | V | A | L | M | G | L | - | - | - | - | - | - | - | L | P | K | - | G | P | R | T | A | I | I | - | - | - | - | - | - | - | - | - | - | - | - | - | - | - | - | - | - | - | - | - | - | - | R | L | C | S | F | F | N | H | L | C | Q | R | V | I | D | I | E | V | I | S | V | M | E | A | E | I | V | E | T | L | C | M | F | E | R | F | F | P | P | T | - | - | - | - | F | F | D | I | M | V | H | L | T | V | H | L | G | R | E | A | R | L | G | G | P | V | H | F | R | W | M | Y | P | F | E | - | - | - | - | - | - | - | - | - | - | - | - | - | - | - | - | - | - | - | - | - | - | - | - | - | - | - | - | - | R | Y | - | - | - | - | - | - | - | - | - | - | - | - | - | - | - | - | - | - | - | - | - | - | - | - | - | - | - | - | - | - | - | - | - | - | - | - | - | - | - | - | - | - | - | - | - | - | - | - | - | - | - | - | - | - | - | - | - | - | - | - | - | - | - | - | - | - | - | - | - | - | - | - | - | - | M | K | V | L | K | D | F | V | R | N | P | A | R | P | E | G | C | I | A | E | S | Y | L | A | E | E | C | M | R | - | - | - | - | F | C | S | D | F | L | K | - | K | T | T | - | - | S | V | E | E | K | P | E | R | N | T | E | Y | E | N | N | S | - | - | - | - | - | - | - | - | - | - | - | - | I | L | E | G | R | P | I | S | A | G | T | S | F | M | L | T | - | - | - | - | E | V | D | K | N | I | A | H | L | A | V | I | Q | N | M | A | A | F | D | P | - | Y | V | D | M | - | - | - | - | - | - | - | - | - | - | - | - | - | - | - | - | - | - | - | - | - | - | - | - | - | - | - | - | - | - | - | - | - | - | - | - | - | - | - | - | - | - | - | - | - | - | - | - | - | - | - | - | - | - | - | - | - | - | - | - | - | - | - | - | - | - | - | - | - | - | - | - | - | - | - | - | - | - | - | - | - | - | - | - | - | - | - | - | - | - | - | - | - | - | H | L | Q | Y | L | Q | D | T | N | P | R | C | K | R | D | A | S | F | L | W | N | M | H | A | K | N | F | A | S | W | I | K | E | Q | - | - | - | - | - | - | - | - | - | - | - | - | - | - | - | - | - | - | - | - | - | - | - | - | - | - | - | - | - | - | - | - | - | - | - | - | - | - | - | - | - | A | N | T | I | K | W | L | A | Y | G | P | R | C | S | A | R | S | Y | S | G | Y | I | V | N | G | Q | R | F | H | T | R | S | L | E | - | - | - | - | - | - | R | Q | S | Q | N | S | G | V | Y | Y | E | A | T | A | M | C | R | S | S | A | K | D | T | A | Q | V | V | D | L | V | S | Y | Y | G | T | V | T | D | I | I | L | L | D | Y | - | - | - | - | - | - | - | - | N | V | F | Y | V | P | I | F | R | - | C | Q | W | A | V | - | - | - | - | - | - | - | - | - | - | - | - | - | - | R | G | N | G | V | K | V | E | D | - | - | G | F | T | L | - | - | - | - | - | - | - | - | - | - | - | V | N | L | - | - | - | N | H | S | Q | V | S | - | - | F | L | K | D | P | F | I | L | A | S | Q | A | K | Q | V | F | Y | S | R | V | D | - | - | D | S | S | - | - | - | - | - | - | - | - | - | - | S | - | - | - | - | - | - | W | Y | V | A | M | K | G | S | S | R | R | Y | - | - | - | - | S | - | - | - | K | E | D | A | Q | E | G | N | A | E | - | I | G | P | L | P | A | D | I | D | M | D | A | E | - | - | - | - | - | - | - | - | - | - | - | - | - | - | - | - | - | - | - | - | - | - | - | - | - | - | - | - | - | - | - | - | - | M | D | E | A | E | Y | A | R | S | D | - | - | - | C | E | G | I | Y | V | P | N | V | D | S | Y | S | F | Y | E | S | L | F | Y | A | L | H | A | L | C | L | L | L | L | F | W | F | Q | I | S | F | L | E | V | - | - | - | - | - | - | - | - | - | - | - | - | - | - | - | - | - | - | - | - | - | - | - | - | - | - | - | - | - | - | - | - | - | - | - | - | - | - | - | - | - | - | - | - | - | - | - | - | - | - | - | - | - | - | - | - | - | - | - | - | - | - | - | - | - | - | - | - | - | - | - | - | - | - | - | - | - | - | - | - | - | - | - | - | - | - | - | - | - | - | - | - | - | - | - | - | - | - | - | - | - | - | - | - | - | - | - | - | - | - | - | - | - | - | - | - | - | - | - | - | - | - | - | - | - | - | - | - | - | - | - | - | - | - | - | - | - | - | - | - | - | - | - | - | - | - | - | - | - | - | - | - | - | - | - | - | - | - | - | - | - | - | - | - | - | - | - | - | - | - | - | - | - | - | - | - | - | - | - | - | - | - | - | - | - | - | - | - | - | - | - | - | - | - | - | - | - | - | - | - | - | - | - | - | - | - | - | - | - | - | - | - | - | - | - | - | - | - | - | - | - | - | - | - | - | - | - | - | - | - | - | - | - | - | - | - | - | - | - | - | - | - | - | - | - | - | - | - | - | - | - | - | - | - | - | - | - | - | - | - | - | - | - | - | - | - | - | - | - | - | - | - | - | - | - | - | - | - | - | - | - | - | - | - | - | - | - | - | - | - | - | - | - | - | - | - | - | - | - | - | - | - | - | - | - | - | - | - | - | - | - | - | - | - | - | - | - | - | - | - | - | - | - | - | - | - | - | - | - | - | - | - | - | - | - | - | - | - | - | - | - | - | - | - | - | - | - | - | - | - | - | - | - | - | - | - | - | - | - | - | - | - | - | - | - | - | - | - | - | - | - | - | - | - | - | - | - | - | - | - | - | - | - | - | - | - | - | - | - | - | - | - | - | - | - | - | - | - | - | - | - | - | - | - | - | - | - | - | - | - | - | - | - | - | - | - | - | - | - | - | - | - | - | - | - | - | - | - | - | - | - | - | - | - | - | - | - | - | - | - | - | - | - | - | - | - | - | - | - | - | - | - | - | - | - | - | - | - | - | - | - | - | - | - | - | - | - | - | - | - | - | - | - | - | - | - | - | - | - | - | - | - | - | - | - | - | - | - | - | - | - | - | - | - | - | - | - | - | - | - | - | - | - | - | - | - | - | - | - | - | - | - | - | - | - | - | - | - | - | - | - | - | - | - | - | - | - | - | - | - | - | - | - | - | - | - | - | - | - | - | - | - | - | - | - | - | - | - | - | - | - | - | - | - | - | - | - | - | - | - | - | - | - | - | - | - | - | - | - | - | - | - | - | - | - | - | - | - | - | - | - | - | - | - | - | - | - | - | - | - | - | - | - | - | - | - | - | - | - | - | - | - | - | - | - | - | - | - | - | - | - | - | - | - | - | - | - | - | - | - | - | - | - | - | - | - | - | - | - | - | - | - | - | - | - | - | - | - | - | - | - | - | - | - | - | - | - | - | - | - | - | - | - | - | - | - | - | - | - | - | - | - | - | - | - | - | - | - | - | - | - | - | - | - | - | - | - | - | - | - | - | - | - | - | - | - | - | - | - | - | - | - | - | - | - | - | - | - | - | - | - | - | - | - | - | - | - | - | - | - | - | - | - | - | - | - | - | - | - | - | - | - | - | - | - | - | - | - | - | - | - | - | - | - | - | - | - | - | - | - | - | - | - |
|  | Chester | - | - | - | - | - | - | - | - | - | - | - | - | - | - | - | - | - | - | - | - | - | - | - | - | - | - | - | - | - | - | - | - | - | - | - | - | - | - | - | - | - | - | - | - | - | - | - | - | - | - | - | - | - | - | - | - | - | - | - | - | - | - | - | M | V | I | C | P | C | S | D | C | R | N | V | D | R | H | L | - | G | - | - | - | - | S | V | V | V | D | H | L | V | R | K | G | M | D | E | A | Y | K | K | R | M | D | W | Y | H | H | - | G | E | - | - | I | I | S | G | D | A | E | D | N | T | V | N | E | C | N | E | - | - | - | - | - | - | - | - | - | - | - | - | - | - | - | - | - | - | - | - | - | - | - | - | - | - | - | - | - | - | - | - | - | - | - | - | - | - | - | - | - | - | - | - | - | - | - | - | - | - | - | - | - | - | - | - | E | I | L | E | L | Y | R | A | A | E | C | L | D | D | E | L | A | - | - | - | - | - | - | - | - | - | - | - | - | - | - | - | - | - | - | - | - | - | - | - | - | - | - | - | - | - | D | D | V | E | G | E | A | E | D | - | - | - | - | - | - | - | - | - | - | - | K | K | E | D | E | F | L | A | K | L | A | D | A | E | T | P | L | Y | - | - | - | P | S | C | A | - | N | H | S | K | L | S | A | I | V | S | L | Y | R | I | K | T | Q | N | G | W | S | - | - | - | - | - | D | K | S | F | N | D | L | L | E | T | L | - | P | K | M | L | P | A | D | N | V | L | H | T | S | M | Y | D | V | K | K | F | L | K | S | F | D | M | G | Y | E | K | I | H | A | C | I | N | D | C | C | L | F | R | K | K | - | F | K | K | L | D | A | C | P | K | C | K | A | S | G | W | K | Q | N | M | H | T | - | - | - | - | - | - | - | - | - | - | - | - | - | - | - | - | - | - | - | - | - | - | - | - | - | - | - | - | - | - | - | - | - | - | - | - | - | - | - | G | Q | I | K | R | G | V | P | Q | K | V | L | R | Y | F | P | I | I | P | R | L | K | R | M | F | R | S | E | V | Q | A | K | E | L | R | W | H | S | T | N | - | - | - | - | - | - | - | - | - | - | K | S | - | - | - | S | D | G | K | - | - | - | - | - | L | R | Y | P | V | D | S | V | T | W | E | Q | V | N | A | K | Y | P | A | - | F | A | P | E | E | R | N | I | R | L | G | L | S | T | D | G | F | N | P | F | N | M | K | N | S | T | Y | S | C | W | P | V | L | L | V | N | Y | N | M | - | A | P | D | L | C | M | K | K | E | N | I | M | L | T | L | L | I | P | G | P | Q | Q | P | G | N | S | I | - | D | V | Y | L | E | P | L | I | E | D | L | I | H | L | W | N | - | E | G | E | V | T | Y | D | A | F | S | K | S | T | F | N | L | K | A | M | L | L | W | T | I | S | D | F | P | A | Y | G | N | L | A | G | C | N | V | K | G | K | M | G | - | - | C | P | L | C | G | K | N | T | D | S | M | W | L | P | N | C | R | K | H | V | Y | M | S | - | H | R | K | G | L | P | S | N | H | S | Y | Q | - | - | S | K | K | S | W | F | D | G | K | A | E | - | H | G | R | - | K | G | R | I | L | T | - | G | R | N | I | S | - | I | M | L | R | N | F | K | - | N | D | F | G | N | - | - | - | - | - | - | - | - | - | - | - | - | M | K | E | K | G | K | K | R | V | R | T | G | S | V | I | E | T | S | S | I | S | E | S | E | D | S | E | S | D | E | E | E | E | V | E | L | D | E | - | E | E | L | S | R | W | K | R | R | S | I | F | F | K | - | L | P | Y | W | E | V | - | Y | L | P | V | R | H | N | L | D | V | M | H | V | E | R | N | V | C | A | S | I | V | A | T | L | L | H | C | - | G | K | S | K | D | G | L | K | T | R | K | D | L | Q | L | L | G | I | R | K | G | L | H | P | - | - | T | E | R | - | - | - | - | - | - | - | - | - | G | K | R | I | Y | L | P | P | A | P | W | S | L | S | K | Q | E | K | K | V | F | C | K | R | L | - | Y | D | F | K | G | P | D | G | Y | C | S | N | I | S | T | C | I | S | L | A | E | C | K | V | M | G | - | L | K | S | H | D | Y | H | V | L | M | Q | Q | L | L | Q | V | A | V | R | G | L | - | - | - | - | - | - | - | L | P | K | - | G | P | R | I | A | I | I | - | - | - | - | - | - | - | - | - | - | - | - | - | - | - | - | - | - | - | - | - | - | - | R | L | S | A | F | F | N | K | L | C | Q | R | V | I | D | M | E | Q | I | T | V | L | E | T | E | I | V | E | T | L | C | M | F | E | R | F | F | P | P | S | - | - | - | - | F | F | D | I | M | V | H | L | C | V | H | L | G | R | E | A | R | L | G | G | P | V | H | F | K | W | M | Y | P | F | E | - | - | - | - | - | - | - | - | - | - | - | - | - | - | - | - | - | - | - | - | - | - | - | - | - | - | - | - | - | R | Y | - | - | - | - | - | - | - | - | - | - | - | - | - | - | - | - | - | - | - | - | - | - | - | - | - | - | - | - | - | - | - | - | - | - | - | - | - | - | - | - | - | - | - | - | - | - | - | - | - | - | - | - | - | - | - | - | - | - | - | - | - | - | - | - | - | - | - | - | - | - | - | - | - | - | M | K | V | M | K | D | Y | V | R | N | P | A | R | P | E | G | C | I | A | E | S | Y | L | A | D | E | C | M | E | - | - | - | - | F | C | R | A | F | L | R | - | K | S | T | - | - | S | V | E | E | K | K | V | R | N | I | E | Y | E | N | N | S | - | - | - | - | - | - | - | - | - | - | - | - | I | L | E | G | R | P | I | S | A | G | T | S | L | I | L | T | - | - | - | - | D | S | E | K | K | I | A | H | L | A | V | I | Q | N | T | A | M | F | D | P | - | Y | V | D | M | - | - | - | - | - | - | - | - | - | - | - | - | - | - | - | - | - | - | - | - | - | - | - | - | - | - | - | - | - | - | - | - | - | - | - | - | - | - | - | - | - | - | - | - | - | - | - | - | - | - | - | - | - | - | - | - | - | - | - | - | - | - | - | - | - | - | - | - | - | - | - | - | - | - | - | - | - | - | - | - | - | - | - | - | - | - | - | - | - | - | - | - | - | - | H | L | E | F | L | Q | N | S | N | V | R | C | R | R | D | A | S | V | L | W | S | T | H | T | H | N | F | A | D | W | L | K | K | Q | - | - | - | - | - | - | - | - | - | - | - | - | - | - | - | - | - | - | - | - | - | - | - | - | - | - | - | - | - | - | - | - | - | - | - | - | - | - | - | - | H | E | D | V | L | K | W | L | A | Y | G | P | R | N | I | A | R | S | Y | S | G | Y | I | V | N | G | L | R | F | H | T | D | K | V | D | - | - | - | - | - | - | R | L | S | Q | N | S | G | V | F | Y | E | A | T | A | M | C | R | S | S | V | K | D | T | S | Q | V | A | D | W | V | T | Y | Y | G | R | V | T | D | I | I | L | L | D | Y | - | - | - | - | - | - | - | - | N | I | F | Y | V | P | I | F | R | - | C | Q | W | A | V | - | - | - | - | - | - | - | - | - | - | - | - | - | - | R | G | N | G | V | K | V | E | D | - | - | G | F | T | L | - | - | - | - | - | - | - | - | - | - | - | V | N | L | - | - | - | F | Q | S | Q | V | S | - | - | F | A | R | D | P | F | I | L | A | S | Q | A | K | Q | I | F | Y | S | R | E | D | - | - | D | S | S | - | - | - | - | - | - | - | - | - | - | - | - | - | - | - | - | - | N | F | V | V | M | R | G | S | S | R | R | Y | - | - | - | - | N | - | - | - | E | E | G | E | E | L | G | D | - | - | - | V | G | P | L | P | S | V | V | D | M | T | I | E | - | - | - | - | - | - | - | - | - | - | - | - | - | - | - | - | - | - | - | - | - | - | - | - | - | - | - | - | - | - | - | D | Q | N | D | E | S | Q | N | A | R | S | D | - | - | - | S | E | G | I | Y | V | - | - | - | - | - | - | - | - | - | - | - | - | - | - | - | - | - | - | - | - | - | - | - | - | - | - | - | - | - | - | - | - | - | - | - | - | - | - | - | - | - | - | - | - | - | - | - | - | - | - | - | - | - | - | - | - | - | - | - | - | - | - | - | - | - | - | - | - | - | - | - | - | - | - | - | - | - | - | - | - | - | - | - | - | - | - | - | - | - | - | - | - | - | - | - | - | - | - | - | - | - | - | - | - | - | - | - | - | - | - | - | - | - | - | - | - | - | - | - | - | - | - | - | - | - | - | - | - | - | - | - | - | - | - | - | - | - | - | - | - | - | - | - | - | - | - | - | - | - | - | - | - | - | - | - | - | - | - | - | - | - | - | - | - | - | - | - | - | - | - | - | - | - | - | - | - | - | - | - | - | - | - | - | - | - | - | - | - | - | - | - | - | - | - | - | - | - | - | - | - | - | - | - | - | - | - | - | - | - | - | - | - | - | - | - | - | - | - | - | - | - | - | - | - | - | - | - | - | - | - | - | - | - | - | - | - | - | - | - | - | - | - | - | - | - | - | - | - | - | - | - | - | - | - | - | - | - | - | - | - | - | - | - | - | - | - | - | - | - | - | - | - | - | - | - | - | - | - | - | - | - | - | - | - | - | - | - | - | - | - | - | - | - | - | - | - | - | - | - | - | - | - | - | - | - | - | - | - | - | - | - | - | - | - | - | - | - | - | - | - | - | - | - | - | - | - | - | - | - | - | - | - | - | - | - | - | - | - | - | - | - | - | - | - | - | - | - | - | - | - | - | - | - | - | - | - | - | - | - | - | - | - | - | - | - | - | - | - | - | - | - | - | - | - | - | - | - | - | - | - | - | - | - | - | - | - | - | - | - | - | - | - | - | - | - | - | - | - | - | - | - | - | - | - | - | - | - | - | - | - | - | - | - | - | - | - | - | - | - | - | - | - | - | - | - | - | - | - | - | - | - | - | - | - | - | - | - | - | - | - | - | - | - | - | - | - | - | - | - | - | - | - | - | - | - | - | - | - | - | - | - | - | - | - | - | - | - | - | - | - | - | - | - | - | - | - | - | - | - | - | - | - | - | - | - | - | - | - | - | - | - | - | - | - | - | - | - | - | - | - | - | - | - | - | - | - | - | - | - | - | - | - | - | - | - | - | - | - | - | - | - | - | - | - | - | - | - | - | - | - | - | - | - | - | - | - | - | - | - | - | - | - | - | - | - | - | - | - | - | - | - | - | - | - | - | - | - | - | - | - | - | - | - | - | - | - | - | - | - | - | - | - | - | - | - | - | - | - | - | - | - | - | - | - | - | - | - | - | - | - | - | - | - | - | - | - | - | - | - | - | - | - | - | - | - | - | - | - | - | - | - | - | - | - | - | - | - | - | - | - | - | - | - | - | - | - | - | - | - | - | - | - | - | - | - | - | - | - | - | - | - | - | - | - | - | - | - | - | - | - | - | - | - | - | - | - | - | - | - | - | - | - | - | - | - | - | - | - | - | - | - | - | - | - | - | - | - | - | - | - | - | - | - | - | - | - | - | - | - | - | - | - | - | - | - | - | - | - | - | - | - | - | - | - | - | - | - | - | - | - | - | - | - | - | - | - | - | - | - | - | - | - | - | - | - | - | - | - | - | - | - | - | - | - | - | - | - | - | - | - | - | - | - | - | - | - | - | - | - | - | - | - | - | - | - | - | - | - | - | - | - | - | - | - | - | - | - | - | - | - | - | - | - | - | - | - | - |
|  | Korbin | - | - | - | - | - | - | - | - | - | - | - | - | - | - | - | - | - | - | - | - | - | - | - | - | - | - | - | - | - | - | - | - | - | - | - | - | - | - | - | - | - | - | - | - | - | - | - | - | - | - | - | - | - | - | - | - | - | - | - | - | - | - | - | - | M | F | C | P | C | T | D | C | R | N | L | C | H | Q | P | - | N | - | - | - | - | D | T | V | L | E | H | L | V | I | K | G | M | D | H | K | Y | K | R | N | G | C | W | S | K | H | - | G | E | - | - | I | R | A | D | K | L | E | A | E | P | S | S | E | F | - | - | - | - | - | - | - | - | - | - | - | - | - | - | - | - | - | - | - | - | - | - | - | - | - | - | - | - | - | - | - | - | - | - | - | - | - | - | - | - | - | - | - | - | - | - | - | - | - | - | - | - | - | - | - | - | - | - | G | A | Y | E | L | I | R | T | A | Y | F | D | G | E | D | H | S | D | S | E | N | Q | N | - | - | - | - | - | - | - | - | - | - | - | - | - | - | - | - | - | - | - | - | - | - | - | E | D | D | S | K | E | P | S | T | - | - | - | - | - | - | - | - | - | - | - | K | E | E | S | D | F | R | E | K | L | K | D | V | E | T | P | L | Y | - | - | - | Y | G | C | P | - | K | Y | T | K | V | Y | A | I | M | G | L | Y | R | I | K | V | K | S | G | M | S | - | - | - | - | - | D | N | Y | F | D | Q | L | L | S | L | V | - | H | D | I | L | P | G | E | N | V | L | R | T | S | T | N | E | I | K | K | F | L | K | M | F | G | F | G | Y | D | I | I | H | A | C | P | N | D | C | I | L | Y | R | K | E | - | Y | E | L | R | D | T | C | P | R | C | S | A | S | R | W | E | R | D | K | H | T | - | - | - | - | - | - | - | - | - | - | - | - | - | - | - | - | - | - | - | - | - | - | - | - | - | - | - | - | - | - | - | - | - | - | - | - | - | - | - | G | E | E | K | K | G | I | P | A | K | V | L | R | Y | F | P | I | K | D | R | F | K | R | M | F | R | S | K | K | M | A | E | D | L | R | W | H | F | S | N | - | - | - | - | - | - | - | - | - | - | A | S | - | - | - | D | D | G | T | - | - | - | - | - | M | R | H | P | V | D | S | L | A | W | A | Q | V | N | D | K | W | P | Q | - | F | A | A | E | P | R | N | L | R | L | G | L | S | T | D | G | M | N | P | F | S | I | Q | N | T | K | Y | S | T | W | P | V | L | L | V | N | Y | N | M | - | A | P | T | M | C | M | K | A | E | N | I | M | L | T | L | L | I | P | G | P | T | S | P | S | N | N | I | - | D | V | Y | L | A | P | L | I | D | D | L | K | D | L | W | S | - | D | G | I | Q | V | Y | D | S | F | L | K | E | S | F | T | L | R | A | M | L | L | W | S | I | S | D | Y | P | A | L | G | T | L | A | G | C | K | V | K | G | K | Q | A | - | - | C | N | V | C | G | K | D | T | P | F | R | W | L | K | F | S | R | K | H | V | C | L | S | - | N | R | K | - | R | P | P | G | H | P | Y | R | - | - | R | R | R | G | W | F | D | N | T | V | E | - | E | G | T | - | T | S | R | I | Q | T | - | R | E | E | I | F | - | E | S | L | K | D | F | K | - | N | D | F | G | R | - | - | - | - | - | - | - | - | - | - | - | - | P | L | E | R | N | G | K | R | K | R | S | D | L | C | - | - | - | - | - | - | - | - | - | - | - | - | - | - | - | - | E | D | D | E | Y | G | E | D | - | T | H | Q | W | R | W | K | K | R | S | I | L | F | E | - | L | P | Y | W | K | - | - | D | L | P | V | R | H | N | I | D | V | M | H | V | E | K | N | V | S | N | A | I | V | S | T | L | M | Q | - | S | T | K | S | K | D | G | L | N | A | R | K | D | L | E | D | M | G | I | R | S | N | L | H | T | - | - | Q | L | R | - | - | - | - | - | - | - | - | - | G | K | R | T | Y | L | P | P | A | V | F | W | L | S | K | D | E | K | K | R | F | C | K | R | L | - | A | R | F | R | G | P | D | G | Y | C | T | N | I | S | N | C | V | S | V | D | P | P | V | I | G | G | - | L | K | S | H | D | H | H | V | L | L | Q | N | L | L | P | V | A | L | R | G | L | - | - | - | - | - | - | - | L | P | K | - | G | P | R | V | A | I | S | - | - | - | - | - | - | - | - | - | - | - | - | - | - | - | - | - | - | - | - | - | - | - | R | L | C | H | Y | F | N | R | L | C | Q | R | V | I | D | P | E | K | L | I | S | L | E | S | E | L | V | E | T | M | C | H | M | E | R | Y | F | P | P | S | - | - | - | - | L | F | D | I | M | F | H | L | P | I | H | L | A | R | E | A | R | L | G | G | P | V | H | F | R | W | M | Y | S | F | E | - | - | - | - | - | - | - | - | - | - | - | - | - | - | - | - | - | - | - | - | - | - | - | - | - | - | - | - | - | R | Y | - | - | - | - | - | - | - | - | - | - | - | - | - | - | - | - | - | - | - | - | - | - | - | - | - | - | - | - | - | - | - | - | - | - | - | - | - | - | - | - | - | - | - | - | - | - | - | - | - | - | - | - | - | - | - | - | - | - | - | - | - | - | - | - | - | - | - | - | - | - | - | - | - | - | M | K | T | L | K | A | F | V | K | N | F | A | R | P | E | A | C | M | D | E | G | Y | L | A | G | E | C | M | A | - | - | - | - | F | C | L | E | F | L | Q | - | D | S | L | - | - | S | V | P | E | V | V | N | R | N | E | D | I | D | S | D | S | L | - | - | - | - | - | - | - | - | - | - | - | V | L | E | G | K | P | L | E | K | A | T | Q | V | T | L | T | - | - | - | - | S | K | E | R | D | I | A | H | R | Y | V | L | M | N | M | A | F | M | D | P | - | Y | V | N | L | - | - | - | - | - | - | - | - | - | - | - | - | - | - | - | - | - | - | - | - | - | - | - | - | - | - | - | - | - | - | - | - | - | - | - | - | - | - | - | - | - | - | - | - | - | - | - | - | - | - | - | - | - | - | - | - | - | - | - | - | - | - | - | - | - | - | - | - | - | - | - | - | - | - | - | - | - | - | - | - | - | - | - | - | - | - | - | - | - | - | - | - | - | - | H | L | E | E | L | Q | A | T | D | A | R | C | M | K | N | E | T | F | L | W | K | T | H | A | E | K | F | S | Q | W | V | K | E | K | V | I | I | - | - | - | - | - | - | - | - | - | - | - | - | - | - | - | - | - | - | - | - | - | - | - | - | - | - | - | - | - | S | I | P | T | N | S | K | E | T | S | T | Q | P | R | W | L | A | F | G | P | R | Q | I | A | Q | S | Y | K | G | F | V | I | N | G | H | R | F | H | T | D | - | - | D | M | K | - | - | - | - | K | K | T | Q | N | S | G | V | T | Y | E | A | F | S | M | C | R | S | S | A | K | D | T | R | Q | Q | A | D | I | V | S | Y | F | G | V | I | K | E | I | I | M | L | D | Y | - | - | - | - | - | - | - | - | H | M | F | Q | V | P | I | F | K | - | C | H | W | A | N | - | - | - | - | - | - | - | - | - | - | - | - | - | - | K | G | N | G | V | K | E | E | D | - | - | G | F | T | L | - | - | - | - | - | - | - | - | - | - | - | V | N | L | - | - | - | Y | L | N | Q | T | A | - | - | Y | L | Q | D | P | Y | I | L | A | A | Q | A | K | H | V | Y | Y | S | R | E | D | - | - | D | S | S | - | - | - | - | - | - | - | - | - | - | P | - | - | - | - | - | - | W | Y | V | V | M | K | A | P | P | R | G | Y | - | - | - | - | H | - | - | - | E | L | E | T | K | E | E | F | V | A | A | S | V | L | I | Q | E | N | D | D | I | G | N | E | - | S | - | - | - | - | - | - | - | - | - | - | - | - | - | - | - | - | - | - | - | - | - | - | - | - | - | - | - | - | - | - | S | D | D | E | S | F | C | V | R | N | D | - | - | - | C | E | G | V | L | V | A | D | - | - | - | - | - | - | - | - | - | - | - | - | - | - | - | - | - | - | - | - | - | - | - | - | - | - | - | - | - | - | - | - | - | - | - | - | - | - | - | - | - | - | - | - | - | - | - | - | - | - | - | - | - | - | - | - | - | - | - | - | - | - | - | - | - | - | - | - | - | - | - | - | - | - | - | - | - | - | - | - | - | - | - | - | - | - | - | - | - | - | - | - | - | - | - | - | - | - | - | - | - | - | - | - | - | - | - | - | - | - | - | - | - | - | - | - | - | - | - | - | - | - | - | - | - | - | - | - | - | - | - | - | - | - | - | - | - | - | - | - | - | - | - | - | - | - | - | - | - | - | - | - | - | - | - | - | - | - | - | - | - | - | - | - | - | - | - | - | - | - | - | - | - | - | - | - | - | - | - | - | - | - | - | - | - | - | - | - | - | - | - | - | - | - | - | - | - | - | - | - | - | - | - | - | - | - | - | - | - | - | - | - | - | - | - | - | - | - | - | - | - | - | - | - | - | - | - | - | - | - | - | - | - | - | - | - | - | - | - | - | - | - | - | - | - | - | - | - | - | - | - | - | - | - | - | - | - | - | - | - | - | - | - | - | - | - | - | - | - | - | - | - | - | - | - | - | - | - | - | - | - | - | - | - | - | - | - | - | - | - | - | - | - | - | - | - | - | - | - | - | - | - | - | - | - | - | - | - | - | - | - | - | - | - | - | - | - | - | - | - | - | - | - | - | - | - | - | - | - | - | - | - | - | - | - | - | - | - | - | - | - | - | - | - | - | - | - | - | - | - | - | - | - | - | - | - | - | - | - | - | - | - | - | - | - | - | - | - | - | - | - | - | - | - | - | - | - | - | - | - | - | - | - | - | - | - | - | - | - | - | - | - | - | - | - | - | - | - | - | - | - | - | - | - | - | - | - | - | - | - | - | - | - | - | - | - | - | - | - | - | - | - | - | - | - | - | - | - | - | - | - | - | - | - | - | - | - | - | - | - | - | - | - | - | - | - | - | - | - | - | - | - | - | - | - | - | - | - | - | - | - | - | - | - | - | - | - | - | - | - | - | - | - | - | - | - | - | - | - | - | - | - | - | - | - | - | - | - | - | - | - | - | - | - | - | - | - | - | - | - | - | - | - | - | - | - | - | - | - | - | - | - | - | - | - | - | - | - | - | - | - | - | - | - | - | - | - | - | - | - | - | - | - | - | - | - | - | - | - | - | - | - | - | - | - | - | - | - | - | - | - | - | - | - | - | - | - | - | - | - | - | - | - | - | - | - | - | - | - | - | - | - | - | - | - | - | - | - | - | - | - | - | - | - | - | - | - | - | - | - | - | - | - | - | - | - | - | - | - | - | - | - | - | - | - | - | - | - | - | - | - | - | - | - | - | - | - | - | - | - | - | - | - | - | - | - | - | - | - | - | - | - | - | - | - | - | - | - | - | - | - | - | - | - | - | - | - | - | - | - | - | - | - | - | - | - | - | - | - | - | - | - | - | - | - | - | - | - | - | - | - | - | - | - | - | - | - | - | - | - | - | - | - | - | - | - | - | - | - | - | - | - | - | - | - | - | - | - | - | - | - | - | - | - | - | - | - | - | - | - | - | - | - | - | - | - | - | - | - | - | - | - | - | - | - | - | - | - | - | - | - | - | - | - | - | - | - | - | - | - | - | - | - | - | - | - | - | - | - | - | - | - | - | - | - | - | - | - | - | - | - | - | - | - | - | - | - | - | - | - | - | - | - | - | - |
|  | EnSpm12\_Fves | - | - | - | - | - | M | - | - | - | - | - | - | - | - | - | - | - | - | - | D | K | - | S | W | I | T | - | - | - | - | - | - | L | D | K | N | S | D | E | Y | R | E | G | V | K | Q | F | V | L | N | S | K | R | Y | A | - | - | - | R | N | - | P | E | M | I | I | C | P | C | A | I | C | R | N | L | S | P | Q | T | - | D | - | - | - | - | D | D | L | E | M | H | L | M | R | Y | D | V | D | P | D | Y | D | I | - | - | - | W | C | A | H | - | G | E | - | - | D | A | G | P | S | I | Q | V | V | D | N | E | S | - | - | - | - | - | - | - | - | - | - | - | - | - | - | - | - | - | - | - | - | - | - | - | - | - | - | - | - | - | - | - | - | - | - | - | - | - | - | - | - | - | - | - | - | S | D | H | - | - | - | - | I | D | V | D | Y | E | L | P | E | V | L | Q | M | Y | K | D | A | H | Y | P | F | S | - | - | - | - | - | - | - | - | - | - | - | - | - | - | - | - | - | - | - | - | - | - | - | - | - | - | - | - | - | - | - | - | D | G | V | G | S | S | R | T | A | - | - | - | - | - | - | - | - | - | - | - | S | I | E | E | E | Y | K | K | K | V | E | E | A | E | V | P | L | Y | - | - | - | P | G | C | K | K | R | Y | T | K | L | S | A | T | L | I | L | Y | K | F | K | A | D | N | G | L | X | - | - | - | - | - | T | E | S | F | D | E | L | L | E | V | F | - | K | D | M | L | P | D | V | N | T | F | P | E | S | N | Y | I | I | K | K | F | L | K | E | F | D | L | G | Y | E | K | I | E | A | C | I | N | D | R | C | L | F | R | G | E | K | K | K | N | M | Q | N | C | P | E | C | N | A | S | R | W | Y | V | N | P | R | T | - | - | - | - | - | - | - | - | - | - | - | - | - | - | - | - | - | - | - | - | - | - | - | - | - | - | - | - | - | - | - | - | - | - | - | - | - | - | - | N | E | I | N | K | G | V | P | A | K | V | L | R | Y | F | P | I | I | P | R | I | Q | R | M | F | L | S | A | S | N | S | E | L | L | T | X | X | S | T | H | - | - | - | - | - | - | - | - | - | - | H | S | - | - | - | Q | D | X | M | - | - | - | - | - | M | R | H | P | V | D | S | I | Q | W | X | T | V | D | Q | K | W | P | S | - | F | A | S | E | P | R | N | L | R | F | G | L | A | T | D | G | F | N | P | Y | K | N | L | S | S | T | H | S | I | W | P | V | I | L | V | I | Y | N | L | - | P | P | N | V | C | M | S | Q | E | N | L | M | L | S | L | L | V | P | G | P | K | Q | P | G | N | D | I | - | D | V | Y | L | E | P | L | I | D | D | L | K | E | L | W | S | - | N | G | V | E | M | Y | D | A | Y | K | R | A | M | F | N | L | K | A | I | L | L | R | T | I | N | D | F | P | A | Y | G | N | L | A | G | L | T | T | K | G | S | X | H | V | L | C | V | V | L | R | H | F | L | N | G | Y | S | L | Q | - | - | - | - | - | - | - | - | - | - | I | R | L | F | I | S | - | - | - | - | - | - | - | - | - | - | - | - | - | - | - | - | - | - | - | - | - | - | - | - | C | P | K | P | R | - | - | - | - | - | - | - | - | - | - | - | - | - | - | - | - | - | - | - | - | - | - | - | - | - | - | - | - | - | - | - | - | - | - | - | - | - | - | - | - | - | - | - | - | - | - | - | - | - | - | - | - | - | - | - | - | - | - | - | - | - | - | - | - | - | - | - | - | - | - | - | - | - | - | - | - | - | - | - | - | - | - | - | - | - | - | - | F | P | L | - | - | - | - | V | L | P | L | R | H | N | L | D | V | M | H | I | E | K | N | V | C | E | S | V | L | A | T | I | L | D | V | N | G | K | S | K | I | D | N | E | S | R | R | D | L | E | L | L | E | L | M | E | D | V | P | N | - | - | D | E | K | - | - | - | - | - | - | - | - | - | R | D | K | L | E | L | P | S | A | P | Y | T | L | T | K | V | K | K | P | K | F | C | G | K | L | - | Y | F | Q | R | F | P | D | G | Y | C | S | N | I | A | N | C | V | K | L | Q | Q | C | K | I | Q | G | - | L | K | S | H | D | H | H | V | L | M | Q | Q | L | L | S | V | A | L | K | G | L | - | - | - | - | - | - | - | L | P | E | - | G | L | R | K | A | I | W | - | - | - | - | - | - | - | - | - | - | - | - | - | - | - | - | - | - | - | - | - | - | - | R | L | S | S | F | F | N | E | L | C | Q | R | V | L | D | K | K | R | L | E | E | L | E | D | E | I | V | L | T | L | C | L | L | E | R | Y | F | P | P | S | - | - | - | - | F | F | D | I | M | I | H | L | T | I | H | I | G | R | E | A | K | L | C | G | P | V | H | F | R | W | M | Y | P | F | E | - | - | - | - | - | - | - | - | - | - | - | - | - | - | - | - | - | - | - | - | - | - | - | - | - | - | - | - | - | R | - | - | - | - | - | - | - | - | - | - | - | - | - | - | - | - | - | - | - | - | - | - | - | - | - | - | - | - | - | - | - | - | - | - | - | - | - | - | - | - | - | - | - | - | - | - | - | - | - | - | - | - | - | - | - | - | - | - | - | - | - | - | - | - | - | - | - | - | - | - | - | - | - | - | - | - | - | - | - | - | - | - | - | - | - | - | - | - | - | - | - | - | - | - | - | - | - | - | - | - | - | - | - | - | - | - | - | - | - | - | - | - | - | - | - | - | - | - | - | - | - | - | - | - | - | - | - | - | - | - | - | - | - | - | - | - | - | - | - | - | - | - | - | - | - | - | - | - | - | - | - | - | - | - | - | - | - | - | - | - | - | - | - | - | - | - | - | - | - | - | - | - | - | - | - | - | - | - | - | - | - | - | - | - | - | - | - | - | - | - | - | - | - | - | - | - | - | - | - | - | - | - | - | - | - | - | - | - | - | - | - | - | - | - | - | - | - | - | - | - | - | - | - | - | - | - | - | - | - | - | - | - | - | - | - | - | - | - | - | - | - | - | - | - | - | - | - | - | - | - | - | - | - | - | - | - | - | - | - | - | - | - | - | - | - | - | - | - | - | - | - | - | - | - | - | - | - | - | - | - | - | - | - | - | - | - | - | - | - | - | - | - | - | - | - | - | - | - | - | - | - | - | - | - | - | - | - | - | - | - | - | - | - | - | - | - | - | - | - | - | - | - | - | - | - | - | - | - | - | - | - | - | - | - | - | - | - | - | - | - | - | - | - | - | - | - | - | - | - | - | - | - | - | - | - | - | - | - | - | - | - | - | - | - | - | - | - | - | - | - | - | - | - | - | - | - | - | - | - | - | - | - | - | - | - | - | - | - | - | - | - | - | - | - | - | - | - | - | - | - | - | - | - | - | - | - | - | - | - | - | - | - | - | - | - | - | - | - | - | - | - | - | - | - | - | - | - | - | - | - | - | - | - | - | - | - | - | - | - | - | - | - | - | - | - | - | - | - | - | - | - | - | - | - | - | - | - | - | - | - | - | - | - | - | - | - | - | - | - | - | - | - | - | - | - | - | - | - | - | - | - | - | - | - | - | - | - | - | - | - | - | - | - | - | - | - | - | - | - | - | - | - | - | - | - | - | - | - | - | - | - | - | - | - | - | - | - | - | - | - | - | - | - | - | - | - | - | - | - | - | - | - | - | - | - | - | - | - | - | - | - | - | - | - | - | - | - | - | - | - | - | - | - | - | - | - | - | - | - | - | - | - | - | - | - | - | - | - | - | - | - | - | - | - | - | - | - | - | - | - | - | - | - | - | - | - | - | - | - | - | - | - | - | - | - | - | - | - | - | - | - | - | - | - | - | - | - | - | - | - | - | - | - | - | - | - | - | - | - | - | - | - | - | - | - | - | - | - | - | - | - | - | - | - | - | - | - | - | - | - | - | - | - | - | - | - | - | - | - | - | - | - | - | - | - | - | - | - | - | - | - | - | - | - | - | - | - | - | - | - | - | - | - | - | - | - | - | - | - | - | - | - | - | - | - | - | - | - | - | - | - | - | - | - | - | - | - | - | - | - | - | - | - | - | - | - | - | - | - | - | - | - | - | - | - | - | - | - | - | - | - | - | - | - | - | - | - | - | - | - | - | - | - | - | - | - | - | - | - | - | - | - | - | - | - | - | - | - | - | - | - | - | - | - | - | - | - | - | - | - | - | - | - | - | - | - | - | - | - | - | - | - | - | - | - | - | - | - | - | - | - | - | - | - | - | - | - | - | - | - | - | - | - | - | - | - | - | - | - | - | - | - | - | - | - | - | - | - | - | - | - | - | - | - | - | - | - | - | - | - | - | - | - | - | - | - | - | - | - | - | - | - | - | - | - | - | - | - | - | - | - | - | - | - | - | - | - | - | - | - | - | - | - | - | - | - | - | - | - | - | - | - | - | - | - | - | - | - | - | - | - | - | - | - | - | - | - | - | - | - | - | - | - | - | - | - | - | - | - | - | - | - | - | - | - | - | - | - | - | - | - | - | - | - | - | - | - | - | - | - | - | - | - | - | - | - | - | - | - | - | - | - | - | - | - | - | - | - | - | - | - | - | - | - | - | - | - | - | - | - | - | - | - | - | - | - | - | - | - | - | - | - | - | - | - | - | - | - | - | - | - | - | - | - | - | - | - | - | - | - | - | - | - | - | - | - | - | - | - | - | - | - | - | - | - | - | - | - | - | - | - | - | - | - | - | - | - | - | - | - | - | - | - | - | - | - | - | - | - | - | - | - | - | - | - | - | - | - | - | - | - | - | - | - | - | - | - | - | - | - | - | - | - | - | - | - | - | - | - | - | - | - | - | - | - | - | - | - | - | - | - | - | - | - | - | - | - | - | - | - | - | - | - | - | - | - | - | - | - | - | - | - | - | - | - | - | - | - | - | - | - | - | - | - | - | - | - | - | - | - | - | - | - | - | - | - | - | - | - | - | - | - | - | - | - | - | - | - | - | - | - | - | - | - | - | - | - | - | - | - | - | - | - | - | - | - | - | - | - | - | - | - | - | - | - | - | - | - | - | - | - | - | - | - | - | - | - | - | - | - | - | - | - | - | - | - | - | - | - | - | - | - | - | - | - | - | - | - | - | - | - | - | - | - | - | - | - | - | - | - | - | - | - | - | - | - | - | - | - | - | - | - | - | - | - | - | - | - | - | - | - | - | - | - | - | - | - | - | - | - | - | - | - | - | - | - | - | - | - | - | - | - | - | - | - | - | - | - | - | - | - | - | - | - | - | - | - | - | - | - | - | - | - | - | - | - | - | - | - | - | - | - | - | - | - | - | - | - | - | - | - | - | - | - | - | - | - | - | - | - | - | - | - | - | - | - | - | - | - | - | - | - | - | - | - | - | - | - | - | - | - | - | - | - | - | - | - | - | - | - | - | - | - | - | - | - | - | - | - | - | - | - | - | - | - | - | - | - | - | - | - | - | - | - | - | - | - | - | - | - | - | - | - | - | - | - | - | - | - | - | - | - | - | - | - | - | - | - | - | - | - | - | - | - | - | - | - | - | - | - | - | - | - | - | - | - | - | - | - | - | - | - | - | - | - | - | - | - | - | - | - | - | - | - | - | - | - | - | - | - | - | - | - | - | - | - | - | - | - | - | - | - | - | - | - | - | - | - | - | - | - | - | - | - | - | - | - | - | - | - | - | - | - | - | - | - | - | - | - | - | - | - | - | - | - | - | - | - | - | - |
|  | A | - | - | - | - | - | - | - | - | - | - | - | - | - | - | - | - | - | - | - | - | - | - | - | - | M | Y | A | - | - | - | - | - | K | E | R | I | N | A | R | W | I | T | E | W | T | V | F | F | G | V | A | K | G | D | M | E | R | - | K | G | - | L | L | M | M | R | C | P | C | R | K | C | G | N | T | C | M | M | K | - | P | - | - | - | - | E | D | V | Q | M | H | V | L | A | S | G | F | V | E | G | Y | S | R | - | - | - | W | T | C | H | - | G | E | - | - | D | A | V | D | V | G | S | G | S | E | D | D | D | V | L | R | Y | D | L | A | N | D | S | E | E | E | T | R | V | D | E | E | A | N | V | - | - | - | - | - | - | - | - | - | - | - | - | - | - | - | - | - | - | - | - | - | - | - | - | - | - | - | - | - | E | G | E | G | A | P | R | G | R | I | A | E | M | L | D | D | P | Y | L | R | - | - | - | - | - | - | - | - | - | - | - | - | - | - | - | - | - | - | - | - | - | - | - | - | - | - | - | - | - | - | - | - | - | - | D | Q | L | E | D | P | E | D | E | - | - | - | - | - | - | - | - | - | - | - | A | E | S | A | Q | F | K | K | L | L | E | D | A | N | T | P | L | Y | - | - | - | D | G | A | G | E | E | N | N | V | L | E | V | T | L | E | L | L | R | L | K | A | A | S | C | W | S | - | - | - | - | - | D | K | G | F | T | D | L | L | S | Y | L | - | A | S | V | F | P | Q | P | N | K | L | P | K | S | T | Y | E | A | K | K | I | T | C | P | L | G | L | D | C | V | K | H | H | S | C | P | N | D | C | M | V | Y | I | G | E | - | Y | E | N | H | E | S | C | H | I | C | G | A | S | R | Y | K | K | K | N | A | R | A | G | E | D | D | G | - | - | - | - | - | - | - | - | - | - | - | - | - | - | - | - | - | - | - | - | - | - | - | - | - | - | - | - | - | - | - | - | - | E | E | V | M | K | G | R | P | A | K | T | V | W | Y | L | T | A | G | G | R | V | E | R | W | M | Q | N | V | K | D | A | R | Y | L | V | H | H | D | P | A | Q | A | A | F | D | P | D | G | H | Y | R | V | - | - | E | E | P | G | V | - | - | - | - | - | L | R | H | P | A | D | G | T | Q | W | R | N | F | D | T | A | F | P | D | - | F | G | A | E | P | R | N | L | R | L | G | L | S | T | D | G | I | N | P | F | G | N | M | N | N | K | H | S | T | W | P | V | I | L | F | V | Y | N | L | - | P | P | W | L | I | M | K | R | K | Y | I | H | M | C | M | L | I | Q | G | P | K | Q | P | G | A | D | L | - | N | V | Y | L | Q | L | V | K | D | E | L | K | Q | L | W | N | - | P | G | R | V | V | W | D | A | N | R | R | E | Y | F | T | M | R | A | A | L | L | T | C | V | H | D | Y | P | A | N | G | N | T | S | C | Q | V | T | H | G | Y | K | A | - | - | C | T | K | C | G | E | N | T | T | S | E | K | L | P | A | S | S | K | I | V | Y | M | G | - | H | R | K | W | L | D | A | K | D | P | W | R | - | - | D | D | K | E | R | F | N | G | K | T | E | - | H | G | T | - | A | P | K | E | K | S | - | G | M | Q | I | L | - | E | I | V | N | D | L | E | - | V | V | P | G | K | - | - | - | - | - | - | - | - | - | - | - | - | - | - | - | - | - | - | - | - | - | - | - | - | - | - | - | - | - | - | - | - | - | - | - | - | - | - | - | - | - | I | A | A | - | K | K | Y | K | E | - | P | E | G | V | V | W | K | R | R | S | V | F | W | D | - | L | E | Y | W | A | - | - | H | L | E | C | R | H | S | I | D | V | M | H | V | E | K | N | V | C | D | S | V | L | G | L | L | M | N | I | P | E | K | T | K | D | G | P | N | A | R | K | D | L | E | L | M | S | I | R | K | E | L | W | G | K | D | Q | V | S | - | A | A | D | K | N | G | F | V | T | V | T | T | R | C | R | L | A | C | Y | N | L | Y | K | D | E | L | H | R | M | C | Q | C | L | - | Q | G | I | K | V | P | S | N | Y | S | S | S | I | K | H | L | V | D | M | K | S | H | K | L | S | G | - | M | K | S | H | D | N | H | V | I | L | T | Q | L | L | P | V | A | I | R | G | I | - | - | - | - | - | - | - | M | E | P | - | H | V | R | E | T | I | M | - | - | - | - | - | - | - | - | - | - | - | - | - | - | - | - | - | - | - | - | - | - | - | K | L | S | D | F | F | D | S | I | S | Q | K | S | I | T | V | R | R | C | Q | I | L | K | D | S | M | I | Q | I | L | C | E | L | E | M | F | F | P | P | S | - | - | - | - | F | F | D | I | M | V | H | L | M | I | H | I | C | D | E | I | L | S | L | G | P | S | F | L | H | N | M | Y | G | P | E | - | - | - | - | - | - | - | - | - | - | - | - | - | - | - | - | - | - | - | - | - | - | - | - | - | - | - | - | - | R | Y | - | - | - | - | - | - | - | - | - | - | - | - | - | - | - | - | - | - | - | - | - | - | - | - | - | - | - | - | - | - | - | - | - | - | - | - | - | - | - | - | - | - | - | - | - | - | - | - | - | - | - | - | - | - | - | - | - | - | - | - | - | - | - | - | - | - | - | - | - | - | - | - | - | - | N | G | V | L | K | R | Y | V | R | N | R | S | R | P | E | G | S | I | V | E | G | F | R | A | E | E | C | I | E | - | - | - | - | F | C | T | D | W | L | A | - | D | Q | K | - | - | S | I | G | V | P | E | S | R | H | K | G | - | - | - | - | - | - | - | - | - | - | - | - | - | - | - | - | - | K | L | D | G | E | G | G | L | G | H | T | Y | L | N | V | Y | G | R | G | R | E | A | D | F | E | R | A | H | L | V | V | L | Q | T | V | D | C | C | R | P | - | Y | Q | E | M | - | - | - | - | - | - | - | - | - | - | - | - | - | - | - | - | - | - | - | - | - | - | - | - | - | - | - | - | - | - | - | - | - | - | - | - | - | - | - | - | - | - | - | - | - | - | - | - | - | - | - | - | - | - | - | - | - | - | - | - | - | - | - | - | - | - | - | - | - | - | - | - | - | - | - | - | - | - | - | - | - | - | - | - | - | - | - | - | - | - | - | - | - | - | H | M | E | Q | L | R | N | E | N | P | K | R | G | E | - | - | V | W | I | H | W | E | H | N | K | T | F | A | A | W | L | K | N | Y | W | Y | G | - | - | - | - | - | - | - | - | - | - | - | - | - | - | - | - | - | - | - | - | - | - | - | - | - | - | - | - | - | - | - | R | E | T | T | N | V | S | E | E | T | V | A | C | L | S | R | E | P | A | Y | H | V | S | T | W | Q | S | Y | A | I | N | G | Y | N | F | Y | T | A | S | Q | D | R | K | - | - | - | - | S | T | Y | Q | N | S | G | V | V | M | T | S | E | T | A | T | D | D | N | S | K | T | - | - | - | - | - | - | - | K | A | F | F | G | V | I | E | E | I | W | E | L | E | Y | - | - | - | - | - | - | - | - | S | I | T | K | I | P | M | F | R | - | V | R | W | T | K | - | - | - | - | - | - | - | - | - | - | - | - | - | - | - | - | - | G | D | K | E | E | S | H | - | R | F | T | T | M | V | L | P | P | E | I | P | R | E | Q | V | D | V | - | - | - | - | - | R | K | I | P | - | - | A | R | E | E | P | W | V | F | A | K | Q | C | K | Q | V | F | Y | I | D | D | P | - | - | A | N | K | - | - | - | - | - | - | - | - | - | - | G | - | - | - | - | - | - | R | V | V | V | R | R | G | K | R | - | - | - | - | - | - | - | - | - | - | - | S | I | V | G | V | D | G | V | - | - | - | T | S | Q | E | - | - | D | Y | E | G | F | H | D | - | P | T | T | A | A | D | D | E | E | A | E | R | T | I | L | T | R | K | R | R | G | K | R | R | S | D | G | S | E | S | E | K | I | P | Q | K | P | Y | L | R | S | S | S | A | R | S | Q | V | M | T | Y | R | R | K | E | K | H | - | - | - | - | - | - | - | - | - | - | - | - | - | - | - | - | - | - | - | - | - | - | - | - | - | - | - | - | - | - | - | - | - | - | - | - | - | - | - | - | - | - | - | - | - | - | - | - | - | - | - | - | - | - | - | - | - | - | - | - | - | - | - | - | - | - | - | - | - | - | - | - | - | - | - | - | - | - | - | - | - | - | - | - | - | - | - | - | - | - | - | - | - | - | - | - | - | - | - | - | - | - | - | - | - | - | - | - | - | - | - | - | - | - | - | - | - | - | - | - | - | - | - | - | - | - | - | - | - | - | - | - | - | - | - | - | - | - | - | - | - | - | - | - | - | - | - | - | - | - | - | - | - | - | - | - | - | - | - | - | - | - | - | - | - | - | - | - | - | - | - | - | - | - | - | - | - | - | - | - | - | - | - | - | - | - | - | - | - | - | - | - | - | - | - | - | - | - | - | - | - | - | - | - | - | - | - | - | - | - | - | - | - | - | - | - | - | - | - | - | - | - | - | - | - | - | - | - | - | - | - | - | - | - | - | - | - | - | - | - | - | - | - | - | - | - | - | - | - | - | - | - | - | - | - | - | - | - | - | - | - | - | - | - | - | - | - | - | - | - | - | - | - | - | - | - | - | - | - | - | - | - | - | - | - | - | - | - | - | - | - | - | - | - | - | - | - | - | - | - | - | - | - | - | - | - | - | - | - | - | - | - | - | - | - | - | - | - | - | - | - | - | - | - | - | - | - | - | - | - | - | - | - | - | - | - | - | - | - | - | - | - | - | - | - | - | - | - | - | - | - | - | - | - | - | - | - | - | - | - | - | - | - | - | - | - | - | - | - | - | - | - | - | - | - | - | - | - | - | - | - | - | - | - | - | - | - | - | - | - | - | - | - | - | - | - | - | - | - | - | - | - | - | - | - | - | - | - | - | - | - | - | - | - | - | - | - | - | - | - | - | - | - | - | - | - | - | - | - | - | - | - | - | - | - | - | - | - | - | - | - | - | - | - | - | - | - | - | - | - | - | - | - | - | - | - | - | - | - | - | - | - | - | - | - | - | - | - | - | - | - | - | - | - | - | - | - | - | - | - | - | - | - | - | - | - | - | - | - | - | - | - | - | - | - | - | - | - | - | - | - | - | - | - | - | - | - | - | - | - | - | - | - | - | - | - | - | - | - | - | - | - | - | - | - | - | - | - | - | - | - | - | - | - | - | - | - | - | - | - | - | - | - | - | - | - | - | - | - | - | - | - | - | - | - | - | - | - | - | - | - | - | - | - | - | - | - | - | - | - | - | - | - | - | - | - | - | - | - | - | - | - | - | - | - | - | - | - | - | - | - | - | - | - | - | - | - | - | - | - | - | - | - | - | - | - | - | - | - | - | - | - | - | - | - | - | - | - | - | - | - | - | - | - | - | - | - | - | - | - | - | - | - | - | - | - | - | - | - | - | - | - | - | - | - | - | - | - | - | - | - | - | - | - | - | - | - | - | - | - | - | - | - | - | - | - | - | - | - | - | - | - | - | - | - | - | - | - | - | - | - | - | - | - | - | - | - | - | - | - | - | - | - | - | - | - | - | - | - | - | - | - | - | - | - | - | - | - | - | - | - | - | - | - | - | - | - | - | - | - | - | - | - | - | - | - | - | - | - | - | - | - | - | - | - | - | - | - | - | - | - | - | - | - | - | - | - | - | - | - | - | - | - | - | - | - | - | - | - | - | - | - | - | - | - | - | - | - | - | - | - |
|  | D | - | - | - | - | - | - | - | - | - | - | - | - | - | - | - | - | - | - | - | - | - | - | - | - | M | Y | - | - | - | - | - | - | E | E | R | M | S | A | S | W | V | A | K | W | A | T | F | Y | D | F | A | V | A | D | M | K | S | - | K | G | - | N | V | K | M | C | C | P | C | D | R | C | R | N | T | V | M | H | L | - | P | - | - | - | - | V | D | V | E | M | H | V | M | A | N | G | F | V | E | G | Y | F | C | - | - | - | W | T | S | H | - | G | E | - | - | V | A | V | E | V | G | S | G | F | E | E | E | H | I | E | F | D | Q | S | G | E | E | Y | R | G | D | D | A | M | D | E | E | T | - | - | - | - | - | - | - | - | - | - | - | - | - | - | - | - | - | - | - | - | - | - | - | - | - | - | - | - | - | - | - | P | V | D | E | A | P | R | G | R | M | G | E | M | L | D | D | V | H | L | Q | - | - | - | - | - | - | - | - | - | - | - | - | - | - | - | - | - | - | - | - | - | - | - | - | - | - | - | - | - | - | - | - | - | - | N | Q | L | K | D | P | D | N | A | - | - | - | - | - | - | - | - | - | - | - | T | D | E | K | K | F | Q | K | L | V | E | D | A | N | T | P | V | Y | - | - | - | D | N | A | G | I | N | N | N | V | L | E | V | T | L | E | L | L | R | I | K | A | K | S | G | L | S | - | - | - | - | - | D | K | G | F | T | E | M | L | S | Y | L | - | A | T | V | F | P | E | G | N | K | L | P | K | S | T | Y | E | A | K | K | I | T | C | P | L | G | L | D | V | V | K | Y | H | A | C | P | L | D | C | I | V | Y | T | G | P | - | H | K | N | K | D | K | C | P | I | C | G | T | S | R | Y | K | K | K | D | G | N | H | D | E | V | D | E | - | - | - | - | - | - | - | - | - | - | - | - | - | - | - | - | - | - | - | - | - | - | - | - | - | - | - | - | - | - | - | - | - | E | E | V | R | K | G | R | P | A | K | I | V | W | Y | L | P | M | G | S | R | F | E | R | W | F | Q | N | L | K | D | A | V | R | L | V | Y | H | D | P | R | E | K | M | Y | D | P | D | G | E | Y | R | T | D | N | T | E | A | G | I | - | - | - | - | - | L | K | H | P | A | D | A | A | Q | W | R | T | L | D | A | E | F | P | D | - | F | A | S | D | P | R | N | L | R | L | G | L | S | T | D | G | I | N | P | F | G | N | M | S | S | T | H | S | T | W | P | V | I | I | F | V | Y | N | L | - | P | P | W | L | I | M | K | K | K | Y | I | H | L | C | M | L | I | Q | G | P | K | Q | P | G | A | D | L | - | N | V | Y | L | Q | L | V | K | D | E | L | A | E | L | W | N | - | T | G | R | K | V | W | D | A | R | R | E | E | Y | F | T | L | R | A | A | L | L | T | C | V | H | D | Y | P | A | N | G | N | T | S | C | Q | C | T | K | G | Y | K | A | - | - | C | T | K | C | A | E | N | T | I | S | E | M | L | P | T | S | R | K | I | V | Y | M | G | - | H | R | R | W | L | H | P | K | D | P | W | R | - | - | L | D | A | E | S | F | N | G | K | E | E | - | H | E | G | - | K | P | R | E | K | S | - | G | F | E | I | Y | - | D | I | V | K | D | L | E | - | V | R | P | G | K | - | - | - | - | - | - | - | - | - | - | - | - | - | - | - | - | - | - | - | - | - | - | - | - | - | - | - | - | - | - | - | - | - | - | - | - | - | - | - | - | - | A | E | N | V | K | D | K | D | G | - | P | D | T | S | V | W | K | R | R | S | V | F | W | G | - | L | P | Y | W | P | - | - | H | L | R | C | R | H | S | I | D | V | M | H | V | E | K | N | V | C | D | S | V | L | G | L | L | M | N | I | P | D | K | T | K | D | G | P | K | A | R | K | D | L | E | I | L | K | I | R | K | E | L | W | A | E | E | E | E | S | - | P | E | D | K | N | G | F | V | T | V | T | T | R | F | N | P | A | C | Y | N | L | S | K | D | E | L | H | R | V | C | E | C | F | - | R | G | I | K | V | P | S | N | Y | S | S | N | I | K | R | L | V | D | M | K | S | H | K | L | V | G | - | M | K | S | H | D | C | H | V | I | I | T | Q | L | L | P | V | A | I | R | G | A | - | - | - | - | - | - | - | M | E | P | - | G | V | R | E | T | V | M | - | - | - | - | - | - | - | - | - | - | - | - | - | - | - | - | - | - | - | - | - | - | - | K | L | C | D | F | F | D | T | I | S | Q | K | S | I | T | V | K | R | C | L | L | L | Q | P | M | M | V | Q | I | L | C | E | F | E | K | Y | F | P | P | T | - | - | - | - | F | F | D | V | M | V | H | L | M | V | H | I | V | D | E | I | L | C | L | G | P | S | F | L | H | N | M | Y | G | P | E | - | - | - | - | - | - | - | - | - | - | - | - | - | - | - | - | - | - | - | - | - | - | - | - | - | - | - | - | - | R | Y | - | - | - | - | - | - | - | - | - | - | - | - | - | - | - | - | - | - | - | - | - | - | - | - | - | - | - | - | - | - | - | - | - | - | - | - | - | - | - | - | - | - | - | - | - | - | - | - | - | - | - | - | - | - | - | - | - | - | - | - | - | - | - | - | - | - | - | - | - | - | - | - | - | - | N | G | V | L | K | R | Y | V | R | N | R | G | R | P | E | G | S | I | L | Q | G | Y | L | V | E | E | C | V | E | - | - | - | - | F | C | T | D | W | L | A | - | D | Q | K | - | - | A | I | G | V | P | E | S | R | H | K | G | - | - | - | - | - | - | - | - | - | - | - | - | - | - | - | - | - | K | L | A | G | Q | G | L | F | G | Q | K | Y | I | E | V | H | S | K | R | R | A | A | D | F | N | R | A | H | L | V | V | L | Q | H | I | D | A | V | R | P | - | Y | Q | E | M | - | - | - | - | - | - | - | - | - | - | - | - | - | - | - | - | - | - | - | - | - | - | - | - | - | - | - | - | - | - | - | - | - | - | - | - | - | - | - | - | - | - | - | - | - | - | - | - | - | - | - | - | - | - | - | - | - | - | - | - | - | - | - | - | - | - | - | - | - | - | - | - | - | - | - | - | - | - | - | - | - | - | - | - | - | - | - | - | - | - | - | - | - | - | H | M | Q | E | L | Q | N | N | N | P | T | R | G | Q | - | - | I | W | I | H | R | E | H | N | K | H | F | A | E | W | L | K | E | Y | W | Y | E | - | - | - | - | - | - | - | - | - | - | - | - | - | - | - | - | - | - | - | - | - | - | - | - | - | - | - | - | - | - | - | R | E | P | V | D | E | N | E | K | T | V | L | R | L | S | R | E | P | A | Y | N | V | A | T | W | Q | A | Y | A | I | N | G | Y | T | Y | Y | T | K | S | Q | D | C | K | - | - | - | - | S | Q | Y | Q | N | S | G | V | V | L | V | S | E | T | A | T | D | S | Q | A | K | T | - | - | - | - | - | - | - | K | A | F | Y | G | V | I | E | E | I | W | D | L | D | Y | - | - | - | - | - | - | - | - | S | I | T | R | I | P | L | F | R | - | V | R | W | A | T | - | - | - | - | - | - | - | - | - | - | - | - | - | - | - | - | - | N | V | K | P | D | G | H | - | R | F | T | T | M | M | L | P | P | E | I | P | R | D | Q | V | N | V | - | - | - | - | - | E | A | I | P | - | - | A | T | Q | E | P | W | V | F | A | N | Q | C | K | Q | V | F | F | I | E | D | P | - | - | S | N | K | - | - | - | - | - | - | - | - | - | - | S | - | - | - | - | - | - | R | V | V | V | R | R | G | K | R | - | - | - | - | - | - | - | - | - | - | - | S | I | V | G | V | D | G | V | - | - | - | T | S | Q | E | - | - | D | Y | D | G | F | H | D | - | P | T | T | A | A | D | D | E | E | A | E | R | T | I | E | T | R | K | R | A | R | K | S | T | G | N | E | - | - | - | - | K | A | T | T | R | A | Y | V | R | K | T | - | - | H | R | E | K | L | T | Y | R | T | R | V | K | K | K | S | R | K | D | D | - | - | - | - | - | - | - | - | - | - | - | - | - | - | - | - | - | - | - | - | - | - | - | - | - | - | - | - | - | - | - | - | - | - | - | - | - | - | - | - | - | - | - | - | - | - | - | - | - | - | - | - | - | - | - | - | - | - | - | - | - | - | - | - | - | - | - | - | - | - | - | - | - | - | - | - | - | - | - | - | - | - | - | - | - | - | - | - | - | - | - | - | - | - | - | - | - | - | - | - | - | - | - | - | - | - | - | - | - | - | - | - | - | - | - | - | - | - | - | - | - | - | - | - | - | - | - | - | - | - | - | - | - | - | - | - | - | - | - | - | - | - | - | - | - | - | - | - | - | - | - | - | - | - | - | - | - | - | - | - | - | - | - | - | - | - | - | - | - | - | - | - | - | - | - | - | - | - | - | - | - | - | - | - | - | - | - | - | - | - | - | - | - | - | - | - | - | - | - | - | - | - | - | - | - | - | - | - | - | - | - | - | - | - | - | - | - | - | - | - | - | - | - | - | - | - | - | - | - | - | - | - | - | - | - | - | - | - | - | - | - | - | - | - | - | - | - | - | - | - | - | - | - | - | - | - | - | - | - | - | - | - | - | - | - | - | - | - | - | - | - | - | - | - | - | - | - | - | - | - | - | - | - | - | - | - | - | - | - | - | - | - | - | - | - | - | - | - | - | - | - | - | - | - | - | - | - | - | - | - | - | - | - | - | - | - | - | - | - | - | - | - | - | - | - | - | - | - | - | - | - | - | - | - | - | - | - | - | - | - | - | - | - | - | - | - | - | - | - | - | - | - | - | - | - | - | - | - | - | - | - | - | - | - | - | - | - | - | - | - | - | - | - | - | - | - | - | - | - | - | - | - | - | - | - | - | - | - | - | - | - | - | - | - | - | - | - | - | - | - | - | - | - | - | - | - | - | - | - | - | - | - | - | - | - | - | - | - | - | - | - | - | - | - | - | - | - | - | - | - | - | - | - | - | - | - | - | - | - | - | - | - | - | - | - | - | - | - | - | - | - | - | - | - | - | - | - | - | - | - | - | - | - | - | - | - | - | - | - | - | - | - | - | - | - | - | - | - | - | - | - | - | - | - | - | - | - | - | - | - | - | - | - | - | - | - | - | - | - | - | - | - | - | - | - | - | - | - | - | - | - | - | - | - | - | - | - | - | - | - | - | - | - | - | - | - | - | - | - | - | - | - | - | - | - | - | - | - | - | - | - | - | - | - | - | - | - | - | - | - | - | - | - | - | - | - | - | - | - | - | - | - | - | - | - | - | - | - | - | - | - | - | - | - | - | - | - | - | - | - | - | - | - | - | - | - | - | - | - | - | - | - | - | - | - | - | - | - | - | - | - | - | - | - | - | - | - | - | - | - | - | - | - | - | - | - | - | - | - | - | - | - | - | - | - | - | - | - | - | - | - | - | - | - | - | - | - | - | - | - | - | - | - | - | - | - | - | - | - | - | - | - | - | - | - | - | - | - | - | - | - | - | - | - | - | - | - | - | - | - | - | - | - | - | - | - | - | - | - | - | - | - | - | - | - | - | - | - | - | - | - | - | - | - | - | - | - | - | - | - | - | - | - | - | - | - | - | - | - | - | - | - | - | - | - | - | - | - | - | - | - | - | - | - | - | - | - | - | - | - | - | - | - | - | - | - | - | - | - | - | - | - | - | - | - | - | - | - | - | - | - | - | - | - | - | - | - | - | - | - | - | - | - | - | - |
|  | B | - | - | - | - | - | - | - | - | - | - | - | - | - | - | - | - | - | - | - | - | - | - | - | - | M | Y | - | - | - | - | - | - | E | D | R | L | R | V | E | W | I | E | R | L | K | T | F | V | D | A | G | V | E | D | M | H | R | - | K | G | - | G | D | K | M | C | C | P | C | V | R | C | R | N | S | K | L | Y | E | - | A | - | - | - | - | E | D | V | E | M | H | L | L | M | R | G | F | V | S | G | Y | S | R | - | - | - | W | T | S | H | - | G | E | - | - | D | G | I | E | V | D | G | G | I | E | M | E | N | M | E | S | D | D | Q | P | G | E | E | A | Y | R | G | V | D | - | - | - | - | - | - | - | - | - | - | - | - | - | - | - | - | - | - | - | - | - | - | - | - | - | - | - | - | - | - | - | - | - | - | - | P | V | Q | D | V | P | T | G | R | M | V | E | M | L | D | D | Q | H | L | Q | - | - | - | - | - | - | - | - | - | - | - | - | - | - | - | - | - | - | - | - | - | - | - | - | - | - | - | - | - | - | - | - | - | - | N | Q | L | D | D | P | E | D | E | - | - | - | - | - | - | - | - | - | - | - | R | V | S | R | K | F | E | K | L | R | E | D | A | E | T | P | L | Y | - | - | - | E | N | A | G | V | D | K | S | V | L | E | V | T | L | E | L | L | R | I | K | A | K | Y | N | I | V | - | - | - | - | - | D | S | G | F | T | E | I | L | S | Y | L | - | R | T | V | L | P | P | G | N | K | L | P | K | S | T | Y | E | A | K | K | V | T | C | P | L | G | L | E | V | I | R | Y | H | A | C | P | L | D | C | I | I | Y | K | G | D | - | Y | K | D | M | H | S | C | P | V | C | K | T | S | R | Y | R | K | K | D | P | N | P | D | G | G | E | E | - | - | - | - | - | - | - | - | - | - | - | - | - | - | - | - | - | - | - | - | - | - | - | - | - | - | - | - | - | - | - | - | - | E | E | L | K | R | G | A | A | A | K | T | V | W | Y | L | P | C | G | T | R | L | E | R | W | F | Q | N | E | K | E | A | R | W | F | I | Y | H | D | A | T | Q | A | D | I | D | P | E | G | V | Y | R | N | - | - | - | D | D | G | V | - | - | - | - | - | L | R | H | P | A | D | A | A | Q | W | R | T | L | D | D | E | F | P | E | - | F | G | A | E | P | R | N | I | R | F | G | M | S | T | D | G | I | N | P | F | G | N | L | S | S | K | H | S | T | W | P | V | I | L | F | I | Y | N | L | - | P | P | W | L | V | M | K | R | K | Y | I | H | L | S | M | L | I | Q | G | P | K | Q | P | G | A | D | L | - | N | V | Y | L | E | L | L | K | D | E | L | K | E | L | W | E | - | K | G | R | K | V | W | D | A | H | K | K | E | E | F | T | L | R | A | A | L | L | T | C | V | H | D | Y | P | A | N | G | N | S | S | C | Q | S | T | H | G | Y | K | A | - | - | C | T | K | C | G | D | E | T | D | G | L | F | L | P | E | S | K | K | I | V | Y | M | G | - | H | R | K | W | L | E | M | K | D | P | W | R | - | - | D | D | K | K | N | F | N | G | R | A | E | - | R | R | H | - | K | P | R | E | L | T | - | G | H | E | V | Y | - | E | I | V | R | D | L | E | - | V | V | A | G | K | - | - | - | - | - | - | - | - | - | - | - | - | - | - | - | - | - | - | - | - | - | - | - | - | - | - | - | - | - | - | - | - | - | - | - | - | - | - | - | - | - | A | Q | P | A | A | G | P | - | - | - | D | G | G | L | T | Y | K | R | R | S | V | F | W | D | - | L | P | Y | W | R | - | - | F | L | Q | S | R | H | T | I | D | V | M | H | V | E | K | N | V | C | D | S | L | L | G | L | M | M | H | H | A | D | K | S | K | D | G | P | K | A | R | K | D | L | Q | W | M | G | I | R | E | K | L | W | P | I | E | E | E | A | P | Q | E | N | K | D | G | E | R | T | I | Y | T | R | C | K | P | A | C | Y | S | L | S | K | A | E | L | H | R | M | C | E | C | L | - | H | G | I | K | V | P | S | N | Y | S | S | S | I | K | K | L | V | D | M | K | S | H | K | L | V | G | - | M | K | S | H | D | C | H | V | I | L | T | Q | L | L | P | V | A | I | R | G | C | - | - | - | - | - | - | - | M | E | P | - | W | V | R | E | T | V | M | - | - | - | - | - | - | - | - | - | - | - | - | - | - | - | - | - | - | - | - | - | - | - | K | L | C | D | F | F | D | T | I | G | Q | K | S | I | T | V | D | R | C | L | Q | L | R | D | A | M | I | Q | I | L | C | E | C | E | M | F | F | P | P | T | - | - | - | - | F | F | D | I | M | V | H | L | M | V | H | V | A | D | E | I | L | L | L | G | P | S | F | L | H | N | M | F | G | P | E | - | - | - | - | - | - | - | - | - | - | - | - | - | - | - | - | - | - | - | - | - | - | - | - | - | - | - | - | - | R | Y | - | - | - | - | - | - | - | - | - | - | - | - | - | - | - | - | - | - | - | - | - | - | - | - | - | - | - | - | - | - | - | - | - | - | - | - | - | - | - | - | - | - | - | - | - | - | - | - | - | - | - | - | - | - | - | - | - | - | - | - | - | - | - | - | - | - | - | - | - | - | - | - | - | - | N | G | V | L | K | R | Y | V | R | N | R | G | R | P | E | G | S | I | M | E | G | Y | H | A | E | E | C | V | E | - | - | - | - | F | C | T | D | W | L | A | - | D | R | K | - | - | P | I | G | V | P | E | S | R | H | K | G | - | - | - | - | - | - | - | - | - | - | - | - | - | - | - | - | - | K | L | E | G | E | G | G | L | G | R | K | E | L | D | V | H | A | R | Q | R | M | D | D | F | I | R | A | H | T | M | V | L | Q | L | T | P | E | V | E | P | - | F | I | D | M | - | - | - | - | - | - | - | - | - | - | - | - | - | - | - | - | - | - | - | - | - | - | - | - | - | - | - | - | - | - | - | - | - | - | - | - | - | - | - | - | - | - | - | - | - | - | - | - | - | - | - | - | - | - | - | - | - | - | - | - | - | - | - | - | - | - | - | - | - | - | - | - | - | - | - | - | - | - | - | - | - | - | - | - | - | - | - | - | - | - | - | - | - | - | H | I | E | E | L | R | N | L | N | P | N | R | N | D | - | - | D | W | I | Q | R | K | H | N | S | T | F | A | M | W | L | K | D | L | W | Y | P | - | - | - | - | - | - | - | - | - | - | - | - | - | - | - | - | - | - | - | - | - | - | - | - | - | - | - | - | - | - | - | R | A | A | A | N | E | D | E | K | I | V | Q | K | L | S | R | L | P | D | R | N | V | V | T | Y | Q | S | Y | A | M | N | G | Y | T | Y | Y | T | K | A | Q | D | R | K | - | - | - | - | S | T | Y | Q | N | S | G | I | V | L | V | A | E | T | G | T | D | S | E | G | Q | T | - | - | - | - | - | - | - | E | A | Y | Y | G | V | V | E | E | I | W | E | L | D | Y | - | - | - | - | - | - | - | - | T | F | T | T | I | A | M | F | R | - | I | R | W | A | R | - | - | - | - | - | - | - | - | - | - | - | - | - | - | - | - | - | D | V | R | H | E | D | H | - | R | F | T | T | M | I | L | P | K | E | I | P | R | H | Q | V | N | V | - | - | - | - | - | K | R | I | S | - | - | A | L | H | E | P | W | Q | F | A | S | K | C | K | Q | V | F | F | V | E | D | P | - | - | I | A | K | - | - | - | - | - | - | - | - | - | - | N | - | - | - | - | - | - | R | V | V | V | R | R | G | K | R | - | - | - | - | - | - | - | - | - | - | - | S | I | V | G | V | D | G | V | - | - | - | T | S | Q | Q | - | - | D | Y | E | G | F | P | D | - | P | T | T | R | Q | E | E | E | V | A | R | R | T | I | Q | T | R | K | K | S | K | L | - | - | - | - | - | - | - | - | - | P | T | Q | D | G | A | Y | A | R | S | S | - | - | H | D | E | V | Q | T | Y | K | R | K | E | K | T | T | K | K | K | D | K | I | V | Q | - | - | - | - | - | - | - | - | - | - | - | - | - | - | - | - | - | - | - | - | - | - | - | - | - | - | - | - | - | - | - | - | - | - | - | - | - | - | - | - | - | - | - | - | - | - | - | - | - | - | - | - | - | - | - | - | - | - | - | - | - | - | - | - | - | - | - | - | - | - | - | - | - | - | - | - | - | - | - | - | - | - | - | - | - | - | - | - | - | - | - | - | - | - | - | - | - | - | - | - | - | - | - | - | - | - | - | - | - | - | - | - | - | - | - | - | - | - | - | - | - | - | - | - | - | - | - | - | - | - | - | - | - | - | - | - | - | - | - | - | - | - | - | - | - | - | - | - | - | - | - | - | - | - | - | - | - | - | - | - | - | - | - | - | - | - | - | - | - | - | - | - | - | - | - | - | - | - | - | - | - | - | - | - | - | - | - | - | - | - | - | - | - | - | - | - | - | - | - | - | - | - | - | - | - | - | - | - | - | - | - | - | - | - | - | - | - | - | - | - | - | - | - | - | - | - | - | - | - | - | - | - | - | - | - | - | - | - | - | - | - | - | - | - | - | - | - | - | - | - | - | - | - | - | - | - | - | - | - | - | - | - | - | - | - | - | - | - | - | - | - | - | - | - | - | - | - | - | - | - | - | - | - | - | - | - | - | - | - | - | - | - | - | - | - | - | - | - | - | - | - | - | - | - | - | - | - | - | - | - | - | - | - | - | - | - | - | - | - | - | - | - | - | - | - | - | - | - | - | - | - | - | - | - | - | - | - | - | - | - | - | - | - | - | - | - | - | - | - | - | - | - | - | - | - | - | - | - | - | - | - | - | - | - | - | - | - | - | - | - | - | - | - | - | - | - | - | - | - | - | - | - | - | - | - | - | - | - | - | - | - | - | - | - | - | - | - | - | - | - | - | - | - | - | - | - | - | - | - | - | - | - | - | - | - | - | - | - | - | - | - | - | - | - | - | - | - | - | - | - | - | - | - | - | - | - | - | - | - | - | - | - | - | - | - | - | - | - | - | - | - | - | - | - | - | - | - | - | - | - | - | - | - | - | - | - | - | - | - | - | - | - | - | - | - | - | - | - | - | - | - | - | - | - | - | - | - | - | - | - | - | - | - | - | - | - | - | - | - | - | - | - | - | - | - | - | - | - | - | - | - | - | - | - | - | - | - | - | - | - | - | - | - | - | - | - | - | - | - | - | - | - | - | - | - | - | - | - | - | - | - | - | - | - | - | - | - | - | - | - | - | - | - | - | - | - | - | - | - | - | - | - | - | - | - | - | - | - | - | - | - | - | - | - | - | - | - | - | - | - | - | - | - | - | - | - | - | - | - | - | - | - | - | - | - | - | - | - | - | - | - | - | - | - | - | - | - | - | - | - | - | - | - | - | - | - | - | - | - | - | - | - | - | - | - | - | - | - | - | - | - | - | - | - | - | - | - | - | - | - | - | - | - | - | - | - | - | - | - | - | - | - | - | - | - | - | - | - | - | - | - | - | - | - | - | - | - | - | - | - | - | - | - | - | - | - | - | - | - | - | - | - | - | - | - | - | - | - | - | - | - | - | - | - | - | - | - | - | - | - | - | - | - | - | - | - | - | - | - | - | - | - | - | - | - | - | - | - | - | - | - | - | - | - | - | - | - | - | - | - | - | - | - | - | - | - | - | - | - | - | - | - | - | - | - | - | - | - | - | - | - | - | - | - | - | - | - | - | - | - | - | - |
|  | E | - | - | - | - | - | - | - | - | - | - | - | - | - | - | - | - | - | - | - | - | - | - | - | - | M | Y | E | - | - | - | - | - | E | T | R | N | C | E | E | W | I | R | K | L | K | D | F | T | D | V | A | T | E | H | M | L | R | - | K | G | - | D | D | M | T | C | C | P | C | S | N | C | R | N | T | M | M | L | L | - | P | - | - | - | - | S | D | V | E | L | H | L | L | L | H | G | F | V | L | G | Y | S | R | - | - | - | W | T | C | H | - | G | E | - | - | D | A | I | E | V | E | S | D | P | E | M | E | N | M | E | H | D | D | P | P | - | D | E | P | C | R | G | - | - | - | - | - | - | - | - | - | - | - | - | - | - | - | - | - | - | - | - | - | - | - | - | - | - | - | - | - | - | - | - | - | - | - | - | - | E | D | Q | E | V | P | Q | G | R | V | A | E | M | L | D | D | V | H | L | R | - | - | - | - | - | - | - | - | - | - | - | - | - | - | - | - | - | - | - | - | - | - | - | - | - | - | - | - | - | - | - | - | - | - | K | Q | L | D | D | A | D | D | E | - | - | - | - | - | - | - | - | - | - | - | L | V | S | R | K | F | Q | K | L | R | E | D | A | E | T | P | L | Y | - | - | - | E | N | A | G | L | D | K | S | V | L | E | V | T | L | E | L | L | R | M | K | A | K | Y | N | I | V | - | - | - | - | - | D | S | G | F | T | D | I | L | S | Y | L | - | S | T | V | L | P | A | G | N | M | L | P | K | S | T | Y | E | A | K | K | V | A | C | P | L | G | L | E | V | V | R | Y | H | A | C | P | L | D | C | I | I | Y | K | G | D | - | Y | K | D | M | H | S | C | P | V | C | K | T | S | R | Y | R | K | K | D | P | N | P | E | G | L | P | E | - | - | - | - | - | - | - | - | - | - | - | - | - | - | - | - | - | - | - | - | - | - | - | - | - | - | - | - | - | - | - | - | - | E | E | V | T | R | G | P | A | A | K | T | V | W | Y | L | P | C | G | S | R | L | E | R | W | F | Q | N | E | K | E | A | R | W | F | I | F | H | D | A | T | Q | K | D | I | D | P | D | G | V | Y | R | N | - | - | - | D | D | G | V | - | - | - | - | - | L | R | H | P | A | D | A | A | Q | W | R | T | L | D | E | E | F | P | D | - | F | G | A | E | P | R | N | I | R | F | G | M | S | T | D | G | I | N | P | F | G | N | M | S | S | K | H | S | T | W | P | V | I | L | F | I | Y | N | L | - | P | P | W | L | I | M | K | R | K | Y | I | H | L | S | M | L | I | Q | G | P | K | Q | P | G | A | D | L | - | N | V | Y | L | E | L | L | K | D | E | L | A | E | L | W | S | - | T | G | R | K | V | W | D | A | H | K | K | E | E | F | T | L | R | A | A | L | L | T | C | V | H | D | Y | P | A | N | G | N | S | S | C | Q | R | T | H | G | Y | K | A | - | - | C | T | K | C | G | D | E | T | N | S | Q | R | L | P | V | S | Q | K | I | V | Y | M | G | - | H | R | K | W | L | Q | R | D | D | P | W | R | - | - | K | D | K | K | N | F | N | G | R | T | E | - | P | R | P | - | K | P | R | E | K | T | - | G | H | E | I | Y | - | E | I | V | R | N | L | E | - | V | V | A | G | K | - | - | - | - | - | - | - | - | - | - | - | - | - | - | - | - | - | - | - | - | - | - | - | - | - | - | - | - | - | - | - | - | - | - | - | - | - | - | - | - | - | A | N | P | Q | A | K | E | K | D | - | G | D | G | P | V | W | K | R | R | S | V | F | W | D | - | L | P | Y | W | K | - | - | F | L | Q | S | R | H | T | I | D | V | M | H | V | E | K | N | V | C | D | S | L | L | G | L | L | M | N | N | A | D | K | T | K | D | G | P | K | A | R | K | D | L | E | F | L | G | I | R | K | K | L | W | A | V | E | E | E | S | - | P | E | D | H | N | G | E | V | T | V | T | T | R | C | K | P | A | C | Y | T | L | S | K | A | E | L | H | R | M | C | Q | C | L | - | H | S | I | K | V | P | S | N | Y | S | S | S | I | K | R | L | V | D | M | K | S | H | K | L | V | G | - | M | K | S | H | D | C | H | V | I | I | T | Q | L | L | P | V | A | I | R | G | A | - | - | - | - | - | - | - | M | E | P | - | W | V | R | E | T | V | M | - | - | - | - | - | - | - | - | - | - | - | - | - | - | - | - | - | - | - | - | - | - | - | K | L | C | D | F | F | D | T | I | G | Q | K | S | I | T | V | E | R | C | L | Q | L | K | D | S | M | I | Q | I | L | C | E | C | E | M | F | F | P | P | T | - | - | - | - | F | F | D | I | M | V | H | L | M | V | H | I | T | D | E | I | I | L | L | G | P | S | F | L | H | N | M | Y | G | P | E | - | - | - | - | - | - | - | - | - | - | - | - | - | - | - | - | - | - | - | - | - | - | - | - | - | - | - | - | - | R | Y | - | - | - | - | - | - | - | - | - | - | - | - | - | - | - | - | - | - | - | - | - | - | - | - | - | - | - | - | - | - | - | - | - | - | - | - | - | - | - | - | - | - | - | - | - | - | - | - | - | - | - | - | - | - | - | - | - | - | - | - | - | - | - | - | - | - | - | - | - | - | - | - | - | - | N | G | V | L | K | R | Y | V | R | N | R | G | H | P | E | G | S | I | M | Q | G | Y | H | A | E | E | C | V | E | - | - | - | - | Y | A | T | D | W | L | A | - | D | R | K | - | - | P | I | G | V | P | E | S | R | H | K | G | - | - | - | - | - | - | - | - | - | - | - | - | - | - | - | - | - | K | L | E | G | E | G | G | L | G | R | K | V | L | D | V | Y | S | S | G | R | E | D | D | Y | M | R | A | H | T | M | V | L | Q | H | M | Y | E | V | Q | P | - | F | I | D | M | - | - | - | - | - | - | - | - | - | - | - | - | - | - | - | - | - | - | - | - | - | - | - | - | - | - | - | - | - | - | - | - | - | - | - | - | - | - | - | - | - | - | - | - | - | - | - | - | - | - | - | - | - | - | - | - | - | - | - | - | - | - | - | - | - | - | - | - | - | - | - | - | - | - | - | - | - | - | - | - | - | - | - | - | - | - | - | - | - | - | - | - | - | - | H | M | E | E | L | E | N | R | Y | P | N | R | S | A | - | - | D | W | I | H | K | T | H | N | A | T | F | A | M | W | L | K | S | F | W | Y | A | - | - | - | - | - | - | - | - | - | - | - | - | - | - | - | - | - | - | - | - | - | - | - | - | - | - | - | - | - | - | - | R | V | A | A | N | E | E | E | R | T | A | Q | K | F | S | R | L | P | D | G | C | V | V | T | Y | Q | S | Y | A | M | N | G | Y | T | Y | Y | T | K | D | Q | D | R | K | - | - | - | - | S | S | Y | Q | N | S | G | I | V | L | V | A | E | T | S | S | D | N | Q | G | T | T | - | - | - | - | - | - | - | E | A | Y | Y | G | V | I | E | E | I | W | E | L | D | Y | - | - | - | - | - | - | - | - | T | F | T | T | I | H | N | N | D | P | A | S | W | G | S | - | - | - | - | - | - | - | - | - | - | - | - | - | - | - | - | - | S | S | K | - | - | - | - | - | - | - | - | - | - | - | - | - | - | - | - | - | - | - | - | V | D | V | - | - | - | - | - | K | K | I | P | - | - | A | R | W | E | P | W | Q | F | A | T | K | C | K | Q | V | F | F | I | D | D | P | - | - | T | S | K | - | - | - | - | - | - | - | - | - | - | N | - | - | - | - | - | - | R | V | V | V | R | R | G | K | R | - | - | - | - | - | - | - | - | - | - | - | S | I | V | G | V | D | G | V | - | - | - | T | S | Q | Q | - | - | D | Y | E | G | F | P | D | - | P | T | S | K | Q | E | E | E | E | A | S | R | T | I | Q | T | R | K | K | S | R | P | - | - | - | - | - | - | - | - | - | E | A | R | E | E | A | Y | A | R | S | G | - | - | H | S | E | T | L | T | Y | K | R | K | E | K | T | R | K | K | K | K | D | K | I | V | E | - | - | - | - | - | - | - | - | - | - | - | - | - | - | - | - | - | - | - | - | - | - | - | - | - | - | - | - | - | - | - | - | - | - | - | - | - | - | - | - | - | - | - | - | - | - | - | - | - | - | - | - | - | - | - | - | - | - | - | - | - | - | - | - | - | - | - | - | - | - | - | - | - | - | - | - | - | - | - | - | - | - | - | - | - | - | - | - | - | - | - | - | - | - | - | - | - | - | - | - | - | - | - | - | - | - | - | - | - | - | - | - | - | - | - | - | - | - | - | - | - | - | - | - | - | - | - | - | - | - | - | - | - | - | - | - | - | - | - | - | - | - | - | - | - | - | - | - | - | - | - | - | - | - | - | - | - | - | - | - | - | - | - | - | - | - | - | - | - | - | - | - | - | - | - | - | - | - | - | - | - | - | - | - | - | - | - | - | - | - | - | - | - | - | - | - | - | - | - | - | - | - | - | - | - | - | - | - | - | - | - | - | - | - | - | - | - | - | - | - | - | - | - | - | - | - | - | - | - | - | - | - | - | - | - | - | - | - | - | - | - | - | - | - | - | - | - | - | - | - | - | - | - | - | - | - | - | - | - | - | - | - | - | - | - | - | - | - | - | - | - | - | - | - | - | - | - | - | - | - | - | - | - | - | - | - | - | - | - | - | - | - | - | - | - | - | - | - | - | - | - | - | - | - | - | - | - | - | - | - | - | - | - | - | - | - | - | - | - | - | - | - | - | - | - | - | - | - | - | - | - | - | - | - | - | - | - | - | - | - | - | - | - | - | - | - | - | - | - | - | - | - | - | - | - | - | - | - | - | - | - | - | - | - | - | - | - | - | - | - | - | - | - | - | - | - | - | - | - | - | - | - | - | - | - | - | - | - | - | - | - | - | - | - | - | - | - | - | - | - | - | - | - | - | - | - | - | - | - | - | - | - | - | - | - | - | - | - | - | - | - | - | - | - | - | - | - | - | - | - | - | - | - | - | - | - | - | - | - | - | - | - | - | - | - | - | - | - | - | - | - | - | - | - | - | - | - | - | - | - | - | - | - | - | - | - | - | - | - | - | - | - | - | - | - | - | - | - | - | - | - | - | - | - | - | - | - | - | - | - | - | - | - | - | - | - | - | - | - | - | - | - | - | - | - | - | - | - | - | - | - | - | - | - | - | - | - | - | - | - | - | - | - | - | - | - | - | - | - | - | - | - | - | - | - | - | - | - | - | - | - | - | - | - | - | - | - | - | - | - | - | - | - | - | - | - | - | - | - | - | - | - | - | - | - | - | - | - | - | - | - | - | - | - | - | - | - | - | - | - | - | - | - | - | - | - | - | - | - | - | - | - | - | - | - | - | - | - | - | - | - | - | - | - | - | - | - | - | - | - | - | - | - | - | - | - | - | - | - | - | - | - | - | - | - | - | - | - | - | - | - | - | - | - | - | - | - | - | - | - | - | - | - | - | - | - | - | - | - | - | - | - | - | - | - | - | - | - | - | - | - | - | - | - | - | - | - | - | - | - | - | - | - | - | - | - | - | - | - | - | - | - | - | - | - | - | - | - | - | - | - | - | - | - | - | - | - | - | - | - | - | - | - | - | - | - | - | - | - | - | - | - | - | - | - | - | - | - | - | - | - | - | - | - | - | - | - | - | - | - | - | - | - | - | - | - | - | - | - | - | - | - | - | - | - | - | - | - | - | - | - | - | - | - | - | - | - | - | - | - | - |
|  | Calvin | - | - | - | - | - | M | - | - | - | - | - | - | - | - | - | - | - | - | - | D | R | - | Q | W | M | Y | - | - | - | - | - | - | A | D | R | R | S | K | E | F | I | D | G | V | H | Y | F | L | R | V | A | E | A | N | R | - | - | - | Q | - | - | R | G | F | I | C | C | P | C | N | K | C | K | N | Q | K | E | Y | S | A | S | - | - | - | - | R | T | I | H | F | H | L | F | E | S | G | F | M | P | S | Y | N | C | - | - | - | W | T | S | H | - | G | E | - | - | Q | G | V | E | M | E | E | D | E | V | E | D | D | N | I | P | D | F | - | - | - | - | - | - | - | - | A | Q | Y | V | G | F | E | G | N | Q | T | G | E | E | E | I | A | - | - | - | - | - | - | - | - | - | - | - | - | - | - | - | - | - | - | - | - | - | A | D | G | N | D | V | A | D | D | L | G | Q | M | L | Q | D | A | R | - | - | - | - | - | - | - | - | - | - | - | - | - | - | - | - | - | - | - | - | - | - | - | - | - | - | - | - | - | - | - | - | - | - | - | - | - | - | - | E | D | C | E | S | E | - | - | - | - | - | - | - | - | - | - | - | K | E | A | H | K | L | D | K | M | L | E | D | H | R | T | S | L | Y | - | - | - | P | G | C | E | Q | G | H | K | K | L | D | T | T | L | E | L | L | Q | W | K | A | K | N | G | V | S | - | - | - | - | - | D | K | A | F | G | D | L | L | K | L | V | - | K | N | I | L | P | G | G | N | K | L | P | E | T | T | Y | E | A | K | K | I | V | C | P | L | G | L | E | V | H | K | I | H | A | C | P | N | D | C | I | L | Y | R | G | E | E | Y | E | N | L | E | A | C | P | V | C | K | A | L | R | Y | K | I | R | R | D | D | P | G | E | V | D | G | - | - | - | - | - | - | - | - | - | - | - | - | - | - | - | - | - | - | - | - | - | - | - | - | - | - | - | - | - | - | - | - | - | Q | L | T | K | K | R | I | P | A | K | V | M | W | Y | F | P | I | I | P | R | L | R | R | L | F | R | N | K | G | N | A | R | M | L | R | W | H | A | E | E | - | - | - | - | - | - | - | - | - | - | R | Q | - | - | - | Q | D | G | M | - | - | - | - | - | L | R | H | P | A | D | G | S | Q | W | R | N | I | D | R | K | F | K | E | - | F | G | K | D | A | R | N | I | R | F | G | L | S | T | D | G | M | N | P | F | G | E | M | S | S | G | H | S | T | W | P | V | T | M | C | I | Y | N | L | - | P | P | W | L | C | M | K | R | K | Y | I | M | M | P | I | I | I | Q | G | P | K | Q | P | G | N | D | I | - | D | V | Y | L | R | P | L | V | E | D | L | K | Q | L | W | K | K | E | G | V | P | V | W | D | E | D | K | Q | E | E | F | N | L | R | A | L | L | F | V | T | I | N | D | W | P | A | L | S | N | L | S | G | Q | S | N | K | G | Y | K | A | - | - | C | T | H | C | M | D | E | T | E | S | T | Y | L | K | H | C | R | K | V | V | Y | M | G | - | H | R | R | F | L | A | A | N | H | P | V | R | - | - | K | K | G | K | H | F | E | H | K | A | D | - | H | R | T | - | K | P | K | H | R | S | - | G | K | T | V | F | - | A | M | V | K | D | L | K | - | V | V | F | G | K | - | - | - | - | - | - | - | - | - | - | - | - | G | P | G | - | - | - | - | - | - | - | - | - | - | - | - | - | - | - | - | - | - | - | - | - | - | - | - | - | - | S | Q | H | I | - | E | S | E | D | - | G | H | A | A | M | W | K | K | N | S | I | F | W | E | - | L | P | Y | W | E | - | - | F | L | D | V | R | H | A | I | D | V | M | H | L | T | K | N | L | C | V | N | L | L | G | - | F | L | G | V | Y | G | K | S | K | D | T | L | E | A | R | N | D | L | K | H | M | E | Q | R | G | D | L | H | P | E | - | P | K | E | - | - | - | - | - | - | - | - | - | K | G | S | H | Y | L | S | P | A | S | Y | T | L | S | K | A | E | K | E | S | M | F | E | C | L | - | E | S | I | K | V | P | S | G | Y | S | T | N | I | K | R | I | I | S | T | K | E | K | K | F | T | N | - | L | K | S | H | D | C | H | V | L | M | T | Q | L | L | P | V | V | I | R | G | I | - | - | - | - | - | - | - | L | P | D | - | N | V | R | A | T | I | T | - | - | - | - | - | - | - | - | - | - | - | - | - | - | - | - | - | - | - | - | - | - | - | K | L | C | A | F | M | N | A | I | S | Q | K | V | I | D | P | D | R | L | E | A | L | Q | N | E | V | V | Q | C | L | V | S | F | E | L | I | F | P | P | S | - | - | - | - | F | F | N | I | M | T | H | L | L | C | H | L | V | K | E | I | R | I | L | G | P | M | Y | L | H | N | M | F | P | F | E | - | - | - | - | - | - | - | - | - | - | - | - | - | - | - | - | - | - | - | - | - | - | - | - | - | - | - | - | - | R | Y | - | - | - | - | - | - | - | - | - | - | - | - | - | - | - | - | - | - | - | - | - | - | - | - | - | - | - | - | - | - | - | - | - | - | - | - | - | - | - | - | - | - | - | - | - | - | - | - | - | - | - | - | - | - | - | - | - | - | - | - | - | - | - | - | - | - | - | - | - | - | - | - | - | - | M | G | V | L | K | K | Y | V | R | N | R | A | R | P | E | A | S | I | A | K | G | Y | G | T | E | E | V | I | E | - | - | - | - | F | C | V | E | F | I | E | - | D | L | R | - | - | P | I | G | V | P | E | S | R | H | E | G | - | - | - | - | - | - | - | - | - | - | - | - | - | - | - | - | - | R | L | R | G | K | G | T | L | G | R | K | A | I | M | T | V | - | - | - | D | N | N | L | F | R | K | A | H | F | T | V | L | Q | H | S | S | L | V | A | P | - | Y | I | E | E | - | - | - | - | - | - | - | - | - | - | - | - | - | - | - | - | - | - | - | - | - | - | - | - | - | - | - | - | - | - | - | - | - | - | - | - | - | - | - | - | - | - | - | - | - | - | - | - | - | - | - | - | - | - | - | - | - | - | - | - | - | - | - | - | - | - | - | - | - | - | - | - | - | - | - | - | - | - | - | - | - | - | - | - | - | - | - | - | - | - | - | - | - | - | H | L | A | L | V | R | A | R | N | I | G | K | S | D | - | - | A | W | I | T | R | H | H | I | D | T | F | P | A | W | L | R | Q | H | L | M | G | - | - | - | - | - | - | - | - | - | - | - | - | - | - | - | - | - | - | - | - | - | - | - | - | - | - | - | - | - | - | - | - | - | - | N | E | S | I | N | Q | Q | L | A | F | L | A | R | G | P | S | G | S | I | A | T | F | Q | G | Y | E | I | N | G | Y | T | F | Y | T | R | A | Q | D | M | K | - | - | - | - | S | T | N | Q | N | S | A | V | R | V | D | A | M | G | H | D | G | T | T | - | - | - | - | - | - | - | - | - | - | A | T | Y | Y | G | A | I | E | D | I | W | E | L | D | Y | - | - | - | - | - | - | - | - | G | P | L | K | V | P | L | F | R | - | C | Q | W | V | R | - | - | - | - | - | - | - | - | - | - | - | - | - | L | T | G | G | G | V | M | I | D | D | S | - | G | M | T | T | - | - | - | - | - | - | - | - | - | - | - | V | D | L | - | - | - | - | - | N | K | V | G | - | - | Y | S | D | E | P | F | V | L | A | N | D | V | T | Q | V | F | F | V | K | D | M | - | - | S | S | K | G | K | K | G | - | - | - | - | - | - | R | G | P | D | E | P | K | R | Q | V | V | L | P | G | K | R | - | - | - | - | - | - | - | - | - | - | - | K | I | V | G | V | E | D | K | - | - | - | T | D | E | - | - | - | D | Y | D | Q | L | D | G | - | Q | P | P | F | T | V | T | I | D | P | S | I | L | L | - | - | - | - | - | - | - | - | - | - | - | - | - | - | - | - | - | S | N | E | D | T | P | Y | S | R | S | D | - | - | H | K | E | G | T | I | V | R | R | K | Y | V | R | S | T | V | T | A | D | V | M | P | - | - | - | - | - | - | - | - | - | - | - | - | - | - | - | - | - | - | - | - | - | - | - | - | - | - | - | - | - | - | - | - | - | - | - | - | - | - | - | - | - | - | - | - | - | - | - | - | - | - | - | - | - | - | - | - | - | - | - | - | - | - | - | - | - | - | - | - | - | - | - | - | - | - | - | - | - | - | - | - | - | - | - | - | - | - | - | - | - | - | - | - | - | - | - | - | - | - | - | - | - | - | - | - | - | - | - | - | - | - | - | - | - | - | - | - | - | - | - | - | - | - | - | - | - | - | - | - | - | - | - | - | - | - | - | - | - | - | - | - | - | - | - | - | - | - | - | - | - | - | - | - | - | - | - | - | - | - | - | - | - | - | - | - | - | - | - | - | - | - | - | - | - | - | - | - | - | - | - | - | - | - | - | - | - | - | - | - | - | - | - | - | - | - | - | - | - | - | - | - | - | - | - | - | - | - | - | - | - | - | - | - | - | - | - | - | - | - | - | - | - | - | - | - | - | - | - | - | - | - | - | - | - | - | - | - | - | - | - | - | - | - | - | - | - | - | - | - | - | - | - | - | - | - | - | - | - | - | - | - | - | - | - | - | - | - | - | - | - | - | - | - | - | - | - | - | - | - | - | - | - | - | - | - | - | - | - | - | - | - | - | - | - | - | - | - | - | - | - | - | - | - | - | - | - | - | - | - | - | - | - | - | - | - | - | - | - | - | - | - | - | - | - | - | - | - | - | - | - | - | - | - | - | - | - | - | - | - | - | - | - | - | - | - | - | - | - | - | - | - | - | - | - | - | - | - | - | - | - | - | - | - | - | - | - | - | - | - | - | - | - | - | - | - | - | - | - | - | - | - | - | - | - | - | - | - | - | - | - | - | - | - | - | - | - | - | - | - | - | - | - | - | - | - | - | - | - | - | - | - | - | - | - | - | - | - | - | - | - | - | - | - | - | - | - | - | - | - | - | - | - | - | - | - | - | - | - | - | - | - | - | - | - | - | - | - | - | - | - | - | - | - | - | - | - | - | - | - | - | - | - | - | - | - | - | - | - | - | - | - | - | - | - | - | - | - | - | - | - | - | - | - | - | - | - | - | - | - | - | - | - | - | - | - | - | - | - | - | - | - | - | - | - | - | - | - | - | - | - | - | - | - | - | - | - | - | - | - | - | - | - | - | - | - | - | - | - | - | - | - | - | - | - | - | - | - | - | - | - | - | - | - | - | - | - | - | - | - | - | - | - | - | - | - | - | - | - | - | - | - | - | - | - | - | - | - | - | - | - | - | - | - | - | - | - | - | - | - | - | - | - | - | - | - | - | - | - | - | - | - | - | - | - | - | - | - | - | - | - | - | - | - | - | - | - | - | - | - | - | - | - | - | - | - | - | - | - | - | - | - | - | - | - | - | - | - | - | - | - | - | - | - | - | - | - | - | - | - | - | - | - | - | - | - | - | - | - | - | - | - | - | - | - | - | - | - | - | - | - | - | - | - | - | - | - | - | - | - | - | - | - | - | - | - | - | - | - | - | - | - | - | - | - | - | - | - | - | - | - | - | - | - | - | - | - | - | - | - | - | - | - | - | - | - | - | - | - | - | - | - | - | - | - | - | - | - | - | - | - | - | - | - | - | - | - | - | - | - | - | - | - | - | - | - | - | - | - | - | - | - | - | - | - | - | - | - | - | - | - | - | - | - | - | - | - | - | - | - | - | - | - | - |
|  | Caspar | - | - | - | - | - | M | - | - | - | - | - | - | - | - | - | - | - | - | - | S | H | - | P | W | M | Y | - | - | - | - | - | - | G | D | R | R | T | T | A | Y | R | E | G | V | H | S | F | R | D | A | A | D | A | N | K | - | - | - | H | G | - | G | G | Y | M | F | C | P | C | V | E | C | R | N | E | K | D | Y | T | S | S | - | - | - | - | R | V | I | Q | S | H | L | L | R | S | G | F | M | S | G | Y | N | V | - | - | - | W | T | K | H | - | G | E | - | - | R | G | V | M | M | E | D | D | D | E | E | E | E | N | D | D | D | N | Y | R | S | M | - | F | P | E | Y | A | D | T | A | M | E | D | N | E | E | E | D | Q | - | - | - | - | - | - | - | - | - | - | - | - | - | - | - | - | - | D | E | E | - | - | - | - | R | E | P | D | E | P | A | D | D | L | G | R | V | I | S | D | A | R | - | - | - | - | - | - | - | - | - | - | - | - | - | - | - | - | - | - | - | - | - | - | - | - | - | - | - | - | - | - | - | - | - | - | - | - | - | - | - | R | G | C | D | T | E | - | - | - | - | - | - | - | - | - | - | - | K | E | R | L | Q | F | E | Q | M | L | Q | D | H | N | K | L | L | Y | - | - | - | P | T | C | E | D | G | Q | K | K | L | G | S | T | L | E | L | L | K | W | K | A | E | T | G | V | T | - | - | - | - | - | D | S | S | F | E | K | L | L | V | L | M | - | K | K | M | L | P | R | K | N | E | L | P | A | S | T | Y | E | A | K | K | L | V | C | P | L | G | L | D | V | Q | K | I | H | A | C | P | N | D | C | I | L | Y | R | G | E | K | Y | E | N | M | D | K | C | P | V | C | T | A | L | R | Y | K | I | R | K | D | D | P | G | D | I | E | G | - | - | - | - | - | - | - | - | - | - | - | - | - | - | - | - | - | - | - | - | - | - | - | - | - | - | - | - | - | - | - | - | - | E | P | P | R | K | R | V | P | A | K | V | M | W | Y | A | P | I | I | P | R | L | K | R | L | F | R | N | K | D | H | A | K | L | L | R | W | H | M | E | D | - | - | - | - | - | - | - | - | - | - | R | M | - | - | - | K | D | D | K | - | - | - | - | - | L | R | H | T | A | D | G | L | Q | W | R | K | I | E | T | E | F | P | R | - | F | A | A | D | A | R | N | L | W | L | G | L | S | T | D | G | M | N | P | F | G | E | Q | S | C | S | H | S | T | W | P | V | T | L | C | I | Y | N | L | - | P | P | W | L | C | M | K | R | K | F | I | M | M | P | V | L | I | Q | G | P | K | Q | P | G | N | D | I | - | D | V | Y | L | R | P | L | V | D | E | L | L | Q | L | W | A | E | P | G | V | R | V | W | D | E | H | K | Q | E | E | F | D | L | R | A | L | L | F | V | T | I | N | D | W | P | A | L | S | N | I | S | G | Q | S | N | K | G | Y | N | A | - | - | C | T | H | C | L | D | Q | T | E | S | I | Y | L | D | K | C | R | K | N | V | Y | P | Y | - | N | R | R | F | L | P | P | K | H | P | L | K | - | - | K | K | G | K | H | F | N | G | K | A | E | - | P | R | G | - | K | P | V | I | R | T | - | G | A | E | V | F | - | D | M | V | K | D | L | K | - | V | I | F | G | K | - | - | - | - | - | - | - | - | - | - | - | - | G | P | G | - | - | - | - | - | - | - | - | - | - | - | - | - | - | - | - | - | - | - | - | - | - | - | - | - | - | S | Q | P | V | P | N | G | P | D | - | K | R | V | P | M | W | K | K | K | S | I | F | W | E | - | L | P | Y | W | E | - | - | V | H | E | V | R | S | V | I | D | V | M | H | L | T | K | N | L | C | V | N | I | L | G | - | F | L | G | L | Y | G | K | S | K | D | T | P | E | A | R | E | D | Q | E | R | H | K | G | R | D | G | M | H | P | - | - | - | - | - | - | - | - | - | - | - | - | - | - | - | - | G | Q | F | Q | G | R | A | S | Y | A | L | T | K | E | E | K | E | I | F | F | E | V | L | - | F | S | I | K | V | P | T | G | F | S | S | N | I | K | G | I | V | N | M | K | D | K | K | F | Q | N | - | L | K | S | H | D | C | H | V | L | M | T | Q | L | L | P | V | A | L | R | G | I | - | - | - | - | - | - | - | L | P | E | - | N | V | R | L | A | I | V | - | - | - | - | - | - | - | - | - | - | - | - | - | - | - | - | - | - | - | - | - | - | - | K | V | C | A | F | L | N | A | I | S | Q | K | V | I | D | R | E | S | L | S | G | L | Q | I | D | V | V | Q | C | L | V | S | F | E | L | L | F | P | P | S | - | - | - | - | F | F | N | I | M | T | H | L | L | V | H | L | V | E | E | I | R | I | L | G | P | V | F | L | H | N | M | F | P | F | E | - | - | - | - | - | - | - | - | - | - | - | - | - | - | - | - | - | - | - | - | - | - | - | - | - | - | - | - | - | R | F | - | - | - | - | - | - | - | - | - | - | - | - | - | - | - | - | - | - | - | - | - | - | - | - | - | - | - | - | - | - | - | - | - | - | - | - | - | - | - | - | - | - | - | - | - | - | - | - | - | - | - | - | - | - | - | - | - | - | - | - | - | - | - | - | - | - | - | - | - | - | - | - | - | - | M | G | V | L | K | K | Y | V | R | N | R | A | R | P | E | G | S | I | S | K | G | Y | G | T | E | D | V | I | E | - | - | - | - | F | C | V | D | F | L | P | - | D | L | K | - | - | P | I | G | V | P | E | S | R | Y | E | G | - | - | - | - | - | - | - | - | - | - | - | - | - | - | - | - | - | R | L | T | G | K | G | T | L | G | R | K | A | K | V | C | M | - | - | - | D | G | H | S | F | S | Q | A | H | Y | T | V | L | H | N | S | T | V | V | A | P | - | Y | I | V | R | - | - | - | - | - | - | - | - | - | - | - | - | - | - | - | - | - | - | - | - | - | - | - | - | - | - | - | - | - | - | - | - | - | - | - | - | - | - | - | - | - | - | - | - | - | - | - | - | - | - | - | - | - | - | - | - | - | - | - | - | - | - | - | - | - | - | - | - | - | - | - | - | - | - | - | - | - | - | - | - | - | - | - | - | - | - | - | - | - | - | - | - | - | - | H | K | N | I | L | R | S | E | N | P | G | K | A | D | - | - | S | W | I | K | G | E | H | E | K | T | F | G | S | W | L | Q | T | H | L | M | N | - | - | - | - | - | - | - | - | - | - | - | - | - | - | - | - | - | - | - | - | - | - | - | - | - | - | - | - | - | - | - | - | - | - | D | D | T | V | G | D | E | L | Y | C | L | A | R | P | P | S | S | T | I | C | T | F | Q | G | Y | E | I | N | G | N | T | F | Y | T | V | A | Q | D | K | K | - | - | - | - | S | T | N | Q | N | S | G | V | R | F | D | A | T | D | E | N | G | H | C | L | - | - | - | - | - | - | - | - | - | E | T | Y | Y | G | Y | I | E | E | I | W | E | L | D | Y | - | - | - | - | - | - | - | G | P | T | F | K | I | P | L | F | R | - | C | K | W | V | K | - | - | - | - | - | - | - | - | - | - | - | - | - | L | T | G | G | G | V | V | V | D | Q | K | Y | G | M | T | T | - | - | - | - | - | - | - | - | - | - | - | V | D | L | - | - | - | - | - | N | N | L | A | - | - | Y | M | D | E | P | F | V | L | A | N | D | V | A | Q | V | F | Y | V | K | D | M | - | - | S | T | K | T | R | K | R | - | N | Q | Q | K | K | I | S | S | D | E | P | K | R | H | I | V | L | S | G | K | R | - | - | - | - | - | - | - | - | - | - | - | N | I | V | G | V | D | D | K | - | - | - | T | D | M | S | E | - | D | Y | N | K | F | H | E | - | I | P | P | F | K | V | K | T | D | P | S | I | L | L | - | - | - | - | - | - | - | - | - | - | - | - | - | - | - | - | - | N | D | E | D | S | P | W | L | R | P | R | R | K | Q | K | - | - | - | - | - | - | - | - | - | - | - | - | - | - | - | - | - | - | - | - | - | - | - | - | - | - | - | - | - | - | - | - | - | - | - | - | - | - | - | - | - | - | - | - | - | - | - | - | - | - | - | - | - | - | - | - | - | - | - | - | - | - | - | - | - | - | - | - | - | - | - | - | - | - | - | - | - | - | - | - | - | - | - | - | - | - | - | - | - | - | - | - | - | - | - | - | - | - | - | - | - | - | - | - | - | - | - | - | - | - | - | - | - | - | - | - | - | - | - | - | - | - | - | - | - | - | - | - | - | - | - | - | - | - | - | - | - | - | - | - | - | - | - | - | - | - | - | - | - | - | - | - | - | - | - | - | - | - | - | - | - | - | - | - | - | - | - | - | - | - | - | - | - | - | - | - | - | - | - | - | - | - | - | - | - | - | - | - | - | - | - | - | - | - | - | - | - | - | - | - | - | - | - | - | - | - | - | - | - | - | - | - | - | - | - | - | - | - | - | - | - | - | - | - | - | - | - | - | - | - | - | - | - | - | - | - | - | - | - | - | - | - | - | - | - | - | - | - | - | - | - | - | - | - | - | - | - | - | - | - | - | - | - | - | - | - | - | - | - | - | - | - | - | - | - | - | - | - | - | - | - | - | - | - | - | - | - | - | - | - | - | - | - | - | - | - | - | - | - | - | - | - | - | - | - | - | - | - | - | - | - | - | - | - | - | - | - | - | - | - | - | - | - | - | - | - | - | - | - | - | - | - | - | - | - | - | - | - | - | - | - | - | - | - | - | - | - | - | - | - | - | - | - | - | - | - | - | - | - | - | - | - | - | - | - | - | - | - | - | - | - | - | - | - | - | - | - | - | - | - | - | - | - | - | - | - | - | - | - | - | - | - | - | - | - | - | - | - | - | - | - | - | - | - | - | - | - | - | - | - | - | - | - | - | - | - | - | - | - | - | - | - | - | - | - | - | - | - | - | - | - | - | - | - | - | - | - | - | - | - | - | - | - | - | - | - | - | - | - | - | - | - | - | - | - | - | - | - | - | - | - | - | - | - | - | - | - | - | - | - | - | - | - | - | - | - | - | - | - | - | - | - | - | - | - | - | - | - | - | - | - | - | - | - | - | - | - | - | - | - | - | - | - | - | - | - | - | - | - | - | - | - | - | - | - | - | - | - | - | - | - | - | - | - | - | - | - | - | - | - | - | - | - | - | - | - | - | - | - | - | - | - | - | - | - | - | - | - | - | - | - | - | - | - | - | - | - | - | - | - | - | - | - | - | - | - | - | - | - | - | - | - | - | - | - | - | - | - | - | - | - | - | - | - | - | - | - | - | - | - | - | - | - | - | - | - | - | - | - | - | - | - | - | - | - | - | - | - | - | - | - | - | - | - | - | - | - | - | - | - | - | - | - | - | - | - | - | - | - | - | - | - | - | - | - | - | - | - | - | - | - | - | - | - | - | - | - | - | - | - | - | - | - | - | - | - | - | - | - | - | - | - | - | - | - | - | - | - | - | - | - | - | - | - | - | - | - | - | - | - | - | - | - | - | - | - | - | - | - | - | - | - | - | - | - | - | - | - | - | - | - | - | - | - | - | - | - | - | - | - | - | - | - | - | - | - | - | - | - | - | - | - | - | - | - | - | - | - | - | - | - | - | - | - | - | - | - | - | - | - | - | - | - | - | - | - | - | - | - | - | - | - | - | - | - | - | - | - | - | - | - | - | - | - | - | - | - | - | - | - | - | - | - | - | - | - | - | - | - | - | - | - |
|  | Conan | - | - | - | - | - | M | - | - | - | - | - | - | - | - | - | - | - | - | - | S | R | - | Q | W | M | Y | - | - | - | - | - | - | D | D | R | C | S | P | Q | F | V | E | G | V | H | T | F | L | L | A | A | E | A | N | K | - | - | - | R | A | - | D | G | F | M | P | C | P | C | A | R | C | K | N | G | H | N | Y | S | T | S | - | - | - | - | R | T | I | H | V | H | L | F | K | S | G | F | M | P | H | Y | N | V | - | - | - | W | T | K | H | - | G | E | - | - | R | G | V | M | M | E | D | N | E | E | E | E | - | - | D | D | D | S | Y | P | G | H | G | F | P | E | Y | D | D | T | T | M | G | - | - | - | - | - | - | E | E | A | E | P | V | M | R | E | E | A | E | P | A | M | R | E | E | A | E | E | - | - | - | - | E | A | S | D | E | P | V | D | D | L | G | R | A | I | A | D | A | K | - | - | - | - | - | - | - | - | - | - | - | - | - | - | - | - | - | - | - | - | - | - | - | - | - | - | - | - | - | - | - | - | - | - | - | - | - | - | - | R | N | C | A | S | D | - | - | - | - | - | - | - | - | - | - | - | L | E | K | K | K | L | Q | R | M | L | E | D | H | K | K | L | L | Y | - | - | - | P | N | C | V | G | D | K | K | K | L | G | T | T | L | E | L | L | Q | W | K | A | E | N | G | V | S | - | - | - | - | - | D | K | G | F | G | K | L | L | V | M | I | - | K | D | M | L | P | K | D | N | E | L | P | E | S | T | Y | E | A | K | K | V | V | C | P | L | G | L | E | V | Q | K | I | H | A | C | P | N | D | C | I | L | Y | R | G | E | - | Y | E | D | L | N | A | C | P | V | C | G | A | L | R | Y | K | I | S | R | D | D | P | G | D | V | E | G | - | - | - | - | - | - | - | - | - | - | - | - | - | - | - | - | - | - | - | - | - | - | - | - | - | - | - | - | - | - | - | - | - | R | R | P | R | K | K | I | P | A | K | V | M | W | Y | A | P | I | I | P | R | L | K | R | L | F | Q | N | K | E | H | A | K | A | M | R | W | H | R | E | D | - | - | - | - | - | - | - | - | - | - | R | K | - | - | - | K | D | G | K | - | - | - | - | - | L | R | V | P | A | D | G | S | Q | W | R | K | I | E | R | K | Y | G | K | E | F | A | D | D | A | R | S | V | W | F | G | L | S | A | D | G | I | N | P | F | G | E | Q | S | S | N | H | S | T | W | P | V | T | L | C | L | Y | N | L | - | P | P | W | L | C | M | K | R | K | F | I | M | M | P | V | L | I | Q | G | P | K | Q | P | G | N | D | I | - | D | V | Y | L | R | P | L | V | E | E | L | L | Q | L | W | N | G | T | G | V | R | A | W | D | E | H | M | G | E | E | F | D | L | K | A | L | L | F | V | T | I | N | D | W | P | A | L | S | N | L | S | G | Q | T | N | K | G | Y | R | A | - | - | C | T | H | C | L | D | D | T | D | S | I | Y | L | A | N | C | K | K | N | V | Y | L | G | - | H | R | R | F | L | P | S | R | H | P | V | R | - | - | K | K | G | K | H | F | K | G | E | A | D | - | H | R | T | - | K | P | R | H | R | T | - | G | A | D | V | H | - | D | M | V | K | D | L | K | - | V | V | F | G | K | - | - | - | - | - | - | - | - | - | - | - | - | G | P | G | - | - | - | - | - | - | - | - | - | - | - | - | - | - | - | - | - | - | - | - | - | - | - | - | - | - | G | Q | P | V | P | N | D | A | D | - | G | R | A | P | M | W | K | K | K | S | I | F | W | D | - | L | P | Y | W | K | - | - | D | L | E | V | R | S | A | I | D | V | M | H | V | T | K | N | L | C | V | T | L | L | G | - | F | L | G | V | Y | G | K | T | K | D | T | P | E | A | R | E | D | Q | Q | C | M | H | G | K | D | G | I | H | Q | - | - | - | - | - | - | - | - | - | - | - | - | - | - | - | - | - | - | - | - | G | H | A | S | Y | A | L | T | K | E | E | K | E | I | F | F | E | C | L | - | L | S | I | K | V | P | S | G | F | S | S | N | I | K | G | I | I | N | M | T | E | K | K | F | Q | N | - | L | K | S | H | D | C | H | V | I | M | T | Q | L | L | P | V | A | L | R | G | L | - | - | - | - | - | - | - | L | P | E | - | N | V | R | L | A | I | V | - | - | - | - | - | - | - | - | - | - | - | - | - | - | - | - | - | - | - | - | - | - | - | K | L | C | A | F | L | N | A | I | S | Q | K | V | I | D | P | E | I | I | P | R | L | Q | N | D | L | V | Q | C | L | V | S | F | E | L | V | F | P | P | S | - | - | - | - | F | F | N | I | M | T | H | V | L | V | H | L | C | E | E | I | N | V | L | G | P | V | F | L | H | N | M | F | P | F | E | - | - | - | - | - | - | - | - | - | - | - | - | - | - | - | - | - | - | - | - | - | - | - | - | - | - | - | - | - | R | F | - | - | - | - | - | - | - | - | - | - | - | - | - | - | - | - | - | - | - | - | - | - | - | - | - | - | - | - | - | - | - | - | - | - | - | - | - | - | - | - | - | - | - | - | - | - | - | - | - | - | - | - | - | - | - | - | - | - | - | - | - | - | - | - | - | - | - | - | - | - | - | - | - | - | M | G | V | L | K | K | Y | V | H | N | R | A | R | P | E | G | S | I | S | K | G | R | Q | N | E | E | V | I | E | - | - | - | - | F | C | I | D | F | I | P | - | D | L | K | - | - | P | I | G | V | P | E | S | R | H | K | G | - | - | - | - | - | - | - | - | - | - | - | - | - | - | - | - | - | R | L | D | G | K | G | T | L | G | G | E | Q | I | I | C | M | - | - | - | D | G | H | S | L | T | E | A | H | Y | T | V | L | Q | N | S | A | L | V | A | P | - | Y | M | D | E | - | - | - | - | - | - | - | - | - | - | - | - | - | - | - | - | - | - | - | - | - | - | - | - | - | - | - | - | - | - | - | - | - | - | - | - | - | - | - | - | - | - | - | - | - | - | - | - | - | - | - | - | - | - | - | - | - | - | - | - | - | - | - | - | - | - | - | - | - | - | - | - | - | - | - | - | - | - | - | - | - | - | - | - | - | - | - | - | - | - | - | - | - | - | H | K | N | L | L | R | S | K | H | P | E | R | S | D | - | - | D | W | I | T | R | E | Q | T | R | S | F | A | S | W | L | Q | A | R | T | M | H | - | - | - | - | - | - | - | - | - | - | - | - | - | - | - | - | - | - | - | - | - | - | - | - | - | - | - | - | - | - | - | - | - | - | D | T | S | I | E | D | D | L | Y | L | L | S | Q | L | P | S | S | N | I | M | T | F | K | G | Y | E | I | N | G | N | T | F | Y | T | I | A | Q | D | K | K | - | - | - | - | S | T | N | Q | N | S | G | V | R | F | D | A | E | T | K | T | G | K | - | - | - | - | - | - | - | - | - | - | - | E | T | Y | Y | G | Y | I | Q | D | I | W | E | L | D | Y | - | - | - | - | - | - | - | R | R | G | L | K | V | P | L | F | R | - | C | K | W | V | N | - | - | - | - | - | - | - | - | - | - | - | - | - | M | T | R | G | G | V | T | E | D | P | Q | Y | G | M | T | T | - | - | - | - | - | - | - | - | - | - | - | V | D | L | - | - | - | - | - | N | N | L | A | - | - | Y | A | D | E | P | F | V | L | A | N | D | V | A | Q | V | F | Y | V | K | D | M | - | - | S | T | K | P | R | K | R | K | D | K | E | A | N | A | S | Y | D | E | P | K | W | H | I | V | L | S | G | K | R | - | - | - | - | - | - | - | - | - | - | - | N | I | V | G | V | D | D | K | - | - | - | T | D | K | S | E | - | D | Y | E | K | F | D | E | - | I | S | P | F | T | V | N | I | D | P | S | I | P | L | - | - | - | - | - | - | - | - | - | - | - | - | - | - | - | - | - | N | D | E | D | F | P | W | L | R | R | K | G | T | H | Q | E | K | V | S | H | P | K | I | W | D | V | I | G | L | L | S | S | L | S | S | V | F | H | T | Q | R | E | S | L | - | - | - | - | - | - | - | - | - | - | - | - | - | - | - | - | - | - | - | - | - | - | - | - | - | - | - | - | - | - | - | - | - | - | - | - | - | - | - | - | - | - | - | - | - | - | - | - | - | - | - | - | - | - | - | - | - | - | - | - | - | - | - | - | - | - | - | - | - | - | - | - | - | - | - | - | - | - | - | - | - | - | - | - | - | - | - | - | - | - | - | - | - | - | - | - | - | - | - | - | - | - | - | - | - | - | - | - | - | - | - | - | - | - | - | - | - | - | - | - | - | - | - | - | - | - | - | - | - | - | - | - | - | - | - | - | - | - | - | - | - | - | - | - | - | - | - | - | - | - | - | - | - | - | - | - | - | - | - | - | - | - | - | - | - | - | - | - | - | - | - | - | - | - | - | - | - | - | - | - | - | - | - | - | - | - | - | - | - | - | - | - | - | - | - | - | - | - | - | - | - | - | - | - | - | - | - | - | - | - | - | - | - | - | - | - | - | - | - | - | - | - | - | - | - | - | - | - | - | - | - | - | - | - | - | - | - | - | - | - | - | - | - | - | - | - | - | - | - | - | - | - | - | - | - | - | - | - | - | - | - | - | - | - | - | - | - | - | - | - | - | - | - | - | - | - | - | - | - | - | - | - | - | - | - | - | - | - | - | - | - | - | - | - | - | - | - | - | - | - | - | - | - | - | - | - | - | - | - | - | - | - | - | - | - | - | - | - | - | - | - | - | - | - | - | - | - | - | - | - | - | - | - | - | - | - | - | - | - | - | - | - | - | - | - | - | - | - | - | - | - | - | - | - | - | - | - | - | - | - | - | - | - | - | - | - | - | - | - | - | - | - | - | - | - | - | - | - | - | - | - | - | - | - | - | - | - | - | - | - | - | - | - | - | - | - | - | - | - | - | - | - | - | - | - | - | - | - | - | - | - | - | - | - | - | - | - | - | - | - | - | - | - | - | - | - | - | - | - | - | - | - | - | - | - | - | - | - | - | - | - | - | - | - | - | - | - | - | - | - | - | - | - | - | - | - | - | - | - | - | - | - | - | - | - | - | - | - | - | - | - | - | - | - | - | - | - | - | - | - | - | - | - | - | - | - | - | - | - | - | - | - | - | - | - | - | - | - | - | - | - | - | - | - | - | - | - | - | - | - | - | - | - | - | - | - | - | - | - | - | - | - | - | - | - | - | - | - | - | - | - | - | - | - | - | - | - | - | - | - | - | - | - | - | - | - | - | - | - | - | - | - | - | - | - | - | - | - | - | - | - | - | - | - | - | - | - | - | - | - | - | - | - | - | - | - | - | - | - | - | - | - | - | - | - | - | - | - | - | - | - | - | - | - | - | - | - | - | - | - | - | - | - | - | - | - | - | - | - | - | - | - | - | - | - | - | - | - | - | - | - | - | - | - | - | - | - | - | - | - | - | - | - | - | - | - | - | - | - | - | - | - | - | - | - | - | - | - | - | - | - | - | - | - | - | - | - | - | - | - | - | - | - | - | - | - | - | - | - | - | - | - | - | - | - | - | - | - | - | - | - | - | - | - | - | - | - | - | - | - | - | - | - | - | - | - | - | - | - | - | - | - | - | - | - | - | - | - | - | - | - | - | - | - | - | - | - | - | - | - | - | - | - | - | - | - | - | - | - | - | - | - | - | - | - | - | - | - | - | - | - | - | - | - | - | - | - | - | - | - | - | - | - |
|  | EnSpm21\_Sbic | - | - | - | - | - | - | - | - | - | - | - | - | - | - | - | - | - | - | - | - | - | - | - | - | - | - | - | - | - | - | - | - | - | - | - | - | - | - | - | - | - | - | - | - | - | - | - | - | - | - | - | - | - | - | - | - | - | - | - | - | - | - | - | - | - | - | - | - | - | - | - | - | - | - | - | - | - | - | - | - | - | - | - | - | - | - | - | - | - | - | - | - | - | - | - | - | - | - | - | - | - | - | - | - | - | - | - | - | - | - | - | - | - | - | - | - | - | - | - | - | - | - | - | - | - | - | - | - | - | - | - | - | - | - | - | - | - | - | - | - | - | - | - | - | - | - | - | - | - | - | - | - | - | - | - | - | - | - | - | - | - | - | - | - | - | - | - | - | - | - | - | - | - | - | - | - | - | - | - | - | - | - | - | - | - | - | - | - | - | - | - | - | - | - | - | - | - | - | - | - | - | - | - | - | - | - | - | - | - | - | - | - | - | - | - | - | - | - | - | - | - | - | - | - | - | - | - | - | - | - | - | - | - | - | - | - | - | - | - | - | - | - | - | - | - | - | - | - | - | - | - | - | - | - | - | - | - | - | - | - | - | - | - | - | - | - | - | - | - | - | - | - | - | - | - | - | - | - | - | - | - | - | - | - | - | - | - | - | - | - | - | - | - | - | - | - | - | - | - | - | - | - | - | - | - | - | - | - | - | - | - | - | - | - | - | - | - | - | - | M | L | P | K | P | N | E | L | P | T | T | T | Y | E | A | K | Q | I | V | C | P | L | G | L | E | I | K | K | I | H | A | C | P | N | D | C | I | L | Y | R | G | K | D | Y | E | N | L | D | E | C | P | V | C | K | A | S | R | Y | K | I | R | R | D | D | P | G | D | V | E | G | E | - | - | - | - | - | - | - | - | - | - | - | - | - | - | - | - | - | - | - | - | - | - | - | - | - | - | - | - | - | - | - | - | E | R | P | R | K | K | I | P | A | K | V | M | W | Y | A | P | I | I | P | R | L | K | R | L | F | R | N | K | D | H | A | K | L | L | R | W | H | K | E | D | - | - | - | - | - | - | - | - | - | - | R | K | - | - | - | V | D | N | M | - | - | - | - | - | L | R | H | P | A | D | G | S | Q | W | R | A | I | D | R | E | F | P | E | - | F | A | K | D | A | R | N | L | R | F | A | L | S | T | D | G | M | N | P | F | G | E | Q | S | S | S | H | S | T | W | P | V | T | L | C | I | Y | N | L | - | P | P | W | L | C | M | K | R | K | F | I | M | M | P | V | L | I | Q | G | P | K | Q | P | G | N | D | I | - | D | V | Y | L | R | P | L | I | E | E | L | L | L | L | W | S | E | T | G | V | R | V | W | D | E | Y | K | Q | E | H | F | D | L | R | A | L | L | F | V | T | I | N | D | W | P | A | L | S | N | L | S | G | Q | T | N | K | G | Y | N | A | - | - | C | T | H | C | F | D | D | L | D | S | I | Y | L | K | K | C | R | K | V | V | Y | L | G | - | Y | R | R | F | L | S | M | N | H | P | V | R | - | - | K | K | G | K | H | F | K | G | K | A | D | - | H | R | C | - | K | P | R | N | R | T | - | G | E | D | V | F | - | E | M | V | K | D | V | K | - | V | V | F | G | K | - | - | - | - | - | - | - | - | - | - | - | - | G | Q | G | - | - | - | - | - | - | - | - | - | - | - | - | - | - | - | - | - | - | - | - | - | - | - | - | - | - | S | Q | P | V | P | K | D | A | A | - | G | H | A | P | M | W | K | K | K | S | I | F | W | E | - | L | P | Y | W | Q | - | - | V | L | E | V | R | N | A | I | D | V | M | H | L | T | K | N | L | C | V | N | L | L | G | - | F | M | G | V | Y | G | K | P | K | D | S | L | E | A | R | Q | D | L | Q | R | M | E | E | R | D | N | L | H | P | E | - | K | T | D | - | - | - | - | - | - | - | - | - | D | G | R | Q | Y | L | R | P | A | S | Y | T | L | S | N | E | E | K | E | I | M | F | E | C | L | - | S | S | I | K | V | P | S | G | F | S | S | N | I | K | G | I | I | N | V | P | E | K | K | F | L | N | - | L | K | S | H | D | C | H | V | L | M | T | Q | L | L | P | V | A | L | R | G | I | - | - | - | - | - | - | - | L | P | P | - | H | V | R | L | A | T | V | - | - | - | - | - | - | - | - | - | - | - | - | - | - | - | - | - | - | - | - | - | - | - | K | L | C | A | F | L | N | A | I | S | Q | K | A | I | N | P | L | E | L | A | A | L | Q | N | D | V | V | Q | C | L | V | S | F | E | L | V | F | P | P | S | - | - | - | - | F | F | D | I | M | T | H | L | L | V | H | L | V | K | E | I | N | I | L | G | P | V | F | L | H | N | M | F | P | F | E | - | - | - | - | - | - | - | - | - | - | - | - | - | - | - | - | - | - | - | - | - | - | - | - | - | - | - | - | - | R | F | - | - | - | - | - | - | - | - | - | - | - | - | - | - | - | - | - | - | - | - | - | - | - | - | - | - | - | - | - | - | - | - | - | - | - | - | - | - | - | - | - | - | - | - | - | - | - | - | - | - | - | - | - | - | - | - | - | - | - | - | - | - | - | - | - | - | - | - | - | - | - | - | - | - | M | G | V | L | K | K | Y | V | H | Q | R | A | R | P | E | G | S | I | A | E | G | Y | G | T | E | E | V | I | E | - | - | - | - | F | C | V | E | F | I | P | - | E | L | D | - | - | P | I | G | V | P | E | S | R | H | E | G | - | - | - | - | - | - | - | - | - | - | - | - | - | - | - | - | - | R | L | S | G | K | G | T | L | G | K | K | T | Y | I | G | T | - | - | - | G | D | D | S | F | N | K | A | H | Y | T | V | L | Q | N | S | S | V | V | E | P | - | Y | V | T | K | - | - | - | - | - | - | - | - | - | - | - | - | - | - | - | - | - | - | - | - | - | - | - | - | - | - | - | - | - | - | - | - | - | - | - | - | - | - | - | - | - | - | - | - | - | - | - | - | - | - | - | - | - | - | - | - | - | - | - | - | - | - | - | - | - | - | - | - | - | - | - | - | - | - | - | - | - | - | - | - | - | - | - | - | - | - | - | - | - | - | - | - | - | - | H | K | D | F | L | R | S | Q | F | P | E | K | N | E | - | - | A | W | F | M | R | Q | H | I | D | T | F | S | D | W | L | R | K | E | C | Q | G | - | - | - | - | - | - | - | - | - | - | - | - | - | - | - | - | - | - | - | - | - | - | - | - | - | - | - | - | - | - | - | - | - | - | N | D | Q | I | D | E | Q | L | Y | L | L | A | R | Q | P | S | W | H | I | L | T | Y | K | G | Y | E | I | N | G | N | T | F | Y | T | L | G | Q | D | K | R | - | - | - | - | S | T | N | Q | N | S | G | V | R | V | D | A | I | D | P | N | G | N | R | - | - | - | - | - | - | - | - | - | - | Q | T | Y | Y | G | R | I | E | E | I | W | E | L | D | Y | - | - | - | - | - | - | - | A | P | N | F | K | I | P | L | F | R | - | C | Q | W | V | K | - | - | - | - | - | - | - | - | - | - | - | - | - | V | T | G | G | G | V | T | V | D | K | D | Y | G | M | T | T | - | - | - | - | - | - | - | - | - | - | - | V | D | L | - | - | - | - | - | N | N | V | G | - | - | Y | K | D | E | P | F | V | L | A | A | D | V | N | Q | V | F | Y | V | K | D | M | - | - | S | T | K | Q | K | R | - | - | G | K | N | D | N | K | S | T | N | E | P | K | R | H | I | V | L | S | G | K | R | - | - | - | - | - | - | - | - | - | - | - | N | I | V | G | I | E | D | K | - | - | - | S | D | I | S | E | - | D | Y | E | R | D | D | R | - | I | T | P | F | N | V | T | K | D | P | S | I | L | I | - | - | - | - | - | - | - | - | - | - | - | - | - | - | - | - | - | N | A | E | D | T | P | W | L | R | Q | D | - | - | H | D | Q | G | T | Y | V | K | K | K | I | T | I | V | P | A | - | - | - | - | - | - | - | - | - | - | - | - | - | - | - | - | - | - | - | - | - | - | - | - | - | - | - | - | - | - | - | - | - | - | - | - | - | - | - | - | - | - | - | - | - | - | - | - | - | - | - | - | - | - | - | - | - | - | - | - | - | - | - | - | - | - | - | - | - | - | - | - | - | - | - | - | - | - | - | - | - | - | - | - | - | - | - | - | - | - | - | - | - | - | - | - | - | - | - | - | - | - | - | - | - | - | - | - | - | - | - | - | - | - | - | - | - | - | - | - | - | - | - | - | - | - | - | - | - | - | - | - | - | - | - | - | - | - | - | - | - | - | - | - | - | - | - | - | - | - | - | - | - | - | - | - | - | - | - | - | - | - | - | - | - | - | - | - | - | - | - | - | - | - | - | - | - | - | - | - | - | - | - | - | - | - | - | - | - | - | - | - | - | - | - | - | - | - | - | - | - | - | - | - | - | - | - | - | - | - | - | - | - | - | - | - | - | - | - | - | - | - | - | - | - | - | - | - | - | - | - | - | - | - | - | - | - | - | - | - | - | - | - | - | - | - | - | - | - | - | - | - | - | - | - | - | - | - | - | - | - | - | - | - | - | - | - | - | - | - | - | - | - | - | - | - | - | - | - | - | - | - | - | - | - | - | - | - | - | - | - | - | - | - | - | - | - | - | - | - | - | - | - | - | - | - | - | - | - | - | - | - | - | - | - | - | - | - | - | - | - | - | - | - | - | - | - | - | - | - | - | - | - | - | - | - | - | - | - | - | - | - | - | - | - | - | - | - | - | - | - | - | - | - | - | - | - | - | - | - | - | - | - | - | - | - | - | - | - | - | - | - | - | - | - | - | - | - | - | - | - | - | - | - | - | - | - | - | - | - | - | - | - | - | - | - | - | - | - | - | - | - | - | - | - | - | - | - | - | - | - | - | - | - | - | - | - | - | - | - | - | - | - | - | - | - | - | - | - | - | - | - | - | - | - | - | - | - | - | - | - | - | - | - | - | - | - | - | - | - | - | - | - | - | - | - | - | - | - | - | - | - | - | - | - | - | - | - | - | - | - | - | - | - | - | - | - | - | - | - | - | - | - | - | - | - | - | - | - | - | - | - | - | - | - | - | - | - | - | - | - | - | - | - | - | - | - | - | - | - | - | - | - | - | - | - | - | - | - | - | - | - | - | - | - | - | - | - | - | - | - | - | - | - | - | - | - | - | - | - | - | - | - | - | - | - | - | - | - | - | - | - | - | - | - | - | - | - | - | - | - | - | - | - | - | - | - | - | - | - | - | - | - | - | - | - | - | - | - | - | - | - | - | - | - | - | - | - | - | - | - | - | - | - | - | - | - | - | - | - | - | - | - | - | - | - | - | - | - | - | - | - | - | - | - | - | - | - | - | - | - | - | - | - | - | - | - | - | - | - | - | - | - | - | - | - | - | - | - | - | - | - | - | - | - | - | - | - | - | - | - | - | - | - | - | - | - | - | - | - | - | - | - | - | - | - | - | - | - | - | - | - | - | - | - | - | - | - | - | - | - | - | - | - | - | - | - | - | - | - | - | - | - | - | - | - | - | - | - | - | - | - | - | - | - | - | - | - | - | - | - | - | - | - | - | - | - | - | - | - | - | - | - | - | - | - | - | - | - | - | - | - | - | - | - | - | - | - | - | - | - | - | - | - | - | - | - | - | - | - | - | - | - | - | - | - | - | - | - | - | - | - | - | - | - | - | - | - |
|  | Clifford | - | - | - | - | - | M | - | - | - | - | - | - | - | - | - | - | - | - | - | N | R | - | Q | W | M | Y | - | - | - | - | - | - | G | D | R | H | T | R | E | Y | I | K | G | V | H | E | F | L | D | A | A | E | A | N | K | - | - | - | Q | - | - | N | G | F | M | C | C | P | C | T | E | C | G | N | T | R | S | Y | S | N | R | - | - | - | - | K | V | L | H | S | H | L | L | Y | K | G | F | M | P | H | Y | N | V | - | - | - | W | T | R | H | - | R | E | - | - | I | G | V | M | M | E | D | G | E | E | D | Y | - | - | D | D | N | Y | V | P | - | - | - | - | P | E | Y | G | D | A | A | T | G | G | A | G | E | D | E | E | E | P | D | D | V | P | N | D | A | A | T | G | E | A | A | E | D | Q | E | E | - | - | - | - | P | T | M | C | P | D | D | D | D | L | R | R | V | I | V | D | A | R | - | - | - | - | - | - | - | - | - | - | - | - | - | - | - | - | - | - | - | - | - | - | - | - | - | - | - | - | - | - | - | - | - | - | - | - | - | - | - | T | Q | C | E | S | Q | - | - | - | - | - | - | - | - | - | - | - | K | E | K | L | K | F | D | H | M | L | E | D | H | K | K | R | V | Y | - | - | - | P | N | C | E | D | G | N | T | K | L | G | T | V | L | E | L | L | Q | - | K | A | E | N | A | V | P | - | - | - | - | - | D | K | G | F | E | K | L | L | K | I | L | - | K | K | K | L | S | K | D | N | E | L | P | D | S | T | Y | A | T | K | K | V | V | C | P | L | G | L | E | V | E | K | I | H | T | C | P | N | D | C | N | L | Y | R | G | A | - | Y | K | D | L | N | A | C | P | V | C | G | A | L | R | Y | K | I | R | R | D | D | P | G | D | V | D | D | - | - | - | - | - | - | - | - | - | - | - | - | - | - | - | - | - | - | - | - | - | - | - | - | - | - | - | - | - | - | - | - | - | E | P | P | R | K | R | V | P | A | K | V | M | W | Y | A | P | I | I | P | R | L | K | R | L | F | R | N | E | E | H | A | K | L | M | R | W | H | S | E | N | - | - | - | - | - | - | - | - | - | - | R | K | - | - | - | K | D | G | K | - | - | - | - | - | L | R | A | P | A | D | G | S | Q | W | R | K | I | E | R | K | Y | W | D | E | F | A | E | D | P | R | N | V | W | F | A | L | S | A | D | G | I | N | P | F | G | E | Q | S | S | N | H | S | T | W | P | V | T | L | C | M | Y | N | L | - | P | P | W | M | C | M | K | R | K | F | I | M | M | P | V | L | I | Q | G | P | K | Q | P | G | N | E | I | - | D | V | Y | L | R | P | L | V | E | E | L | L | Q | L | W | N | E | N | G | V | R | T | W | D | E | H | R | Q | E | E | F | N | L | K | S | L | L | F | V | T | I | N | D | W | P | A | L | S | N | L | S | G | Q | T | N | K | G | Y | H | A | - | - | C | T | H | C | L | D | D | T | E | S | I | Y | L | D | K | C | R | K | N | V | Y | L | G | - | H | R | R | F | L | P | T | N | H | Q | C | - | - | - | E | K | G | K | H | F | K | G | E | A | D | - | H | R | K | - | K | P | A | M | R | T | - | G | D | D | V | L | - | A | M | V | N | D | L | H | Y | V | I | F | G | K | - | - | - | - | - | - | - | - | - | - | - | - | G | P | G | - | - | - | - | - | - | - | - | - | - | - | - | - | - | - | - | - | - | - | - | - | - | - | - | - | - | G | L | A | V | P | N | D | A | E | - | G | H | A | P | M | W | K | K | K | S | I | F | W | D | - | L | P | Y | W | K | - | - | D | L | E | V | R | S | S | I | D | V | M | H | V | T | K | N | L | C | V | N | L | L | G | - | F | L | G | V | Y | G | K | T | K | D | T | P | E | A | R | E | D | L | Q | R | L | H | E | K | D | G | M | P | P | - | - | - | - | - | - | - | - | - | - | - | - | - | - | - | - | K | Q | Y | E | G | P | A | S | Y | A | L | T | K | E | E | K | E | I | F | F | E | C | L | - | L | S | M | K | V | T | T | G | F | S | S | N | I | K | G | I | I | N | M | P | E | K | K | F | Q | N | - | L | K | S | H | D | C | H | V | I | M | T | Q | L | L | P | V | A | L | R | G | L | - | - | - | - | - | - | - | L | P | E | - | N | V | R | L | A | I | V | - | - | - | - | - | - | - | - | - | - | - | - | - | - | - | - | - | - | - | - | - | - | - | K | L | C | A | F | L | N | A | I | S | Q | K | V | I | D | P | E | I | I | P | R | L | R | S | D | V | A | Q | C | L | V | S | F | E | L | V | F | P | P | S | - | - | - | - | F | F | N | I | M | T | H | V | L | V | H | L | V | D | E | I | S | S | W | - | P | V | F | L | H | N | M | F | P | F | E | - | - | - | - | - | - | - | - | - | - | - | - | - | - | - | - | - | - | - | - | - | - | - | - | - | - | - | - | - | R | F | - | - | - | - | - | - | - | - | - | - | - | - | - | - | - | - | - | - | - | - | - | - | - | - | - | - | - | - | - | - | - | - | - | - | - | - | - | - | - | - | - | - | - | - | - | - | - | - | - | - | - | - | - | - | - | - | - | - | - | - | - | - | - | - | - | - | - | - | - | - | - | - | - | - | M | G | V | L | K | K | Y | V | R | N | R | A | R | P | E | G | S | I | Y | - | - | - | - | - | - | - | - | - | - | - | - | - | - | - | - | - | - | - | - | - | - | - | - | - | - | - | - | - | - | L | P | K | S | R | Y | E | G | - | - | - | - | - | - | - | - | - | - | - | - | - | - | - | - | - | R | L | T | G | K | G | T | L | G | R | D | S | I | I | C | R | - | - | - | D | G | Y | S | W | S | Q | A | H | Y | T | V | L | Q | N | S | T | L | V | T | P | - | Y | V | D | E | - | - | - | - | - | - | - | - | - | - | - | - | - | - | - | - | - | - | - | - | - | - | - | - | - | - | - | - | - | - | - | - | - | - | - | - | - | - | - | - | - | - | - | - | - | - | - | - | - | - | - | - | - | - | - | - | - | - | - | - | - | - | - | - | - | - | - | - | - | - | - | - | - | - | - | - | - | - | - | - | - | - | - | - | - | - | - | - | - | - | - | - | - | - | H | K | N | S | L | R | S | K | H | P | E | Q | C | D | - | - | D | W | I | T | C | E | H | I | R | T | F | S | S | W | L | E | T | R | L | R | G | - | - | - | - | - | - | - | - | - | - | - | - | - | - | - | - | - | - | - | - | - | - | - | - | - | - | - | - | - | - | - | - | - | - | D | N | T | V | C | D | E | L | Y | L | L | S | R | G | P | S | L | T | V | L | T | Y | K | G | Y | E | I | N | G | N | T | F | Y | T | I | P | - | D | Q | K | - | - | - | - | S | T | N | Q | N | S | G | V | R | F | D | A | A | T | E | S | G | K | - | - | - | - | - | - | - | - | - | - | - | D | T | Y | Y | G | Y | I | V | D | I | W | E | L | D | Y | - | - | - | - | - | - | - | G | P | D | F | K | V | P | L | F | K | - | C | K | W | V | N | - | - | - | - | - | - | - | - | - | - | - | - | - | L | L | G | G | G | V | Q | V | D | P | Q | Y | G | M | T | T | - | - | - | - | - | - | - | - | - | - | - | V | D | L | - | - | - | - | - | K | N | L | G | - | - | Y | T | D | E | P | F | V | L | A | N | D | V | A | Q | V | I | Y | V | K | D | M | - | - | S | T | K | P | R | K | E | - | D | K | E | A | N | T | S | Y | D | E | P | K | R | H | I | V | L | S | G | K | R | - | - | - | - | - | - | - | - | - | - | - | D | I | L | G | V | E | A | R | - | - | - | Q | T | C | L | K | - | I | M | K | S | F | M | K | - | F | L | P | S | M | S | R | L | T | Q | A | S | - | - | - | - | - | - | - | - | - | - | - | - | - | - | - | - | - | - | - | - | - | - | - | - | - | - | - | - | - | - | - | - | - | - | - | - | - | - | - | - | - | - | - | - | - | - | - | - | - | - | - | - | - | - | - | - | - | - | - | - | - | - | - | - | - | - | - | - | - | - | - | - | - | - | - | - | - | - | - | - | - | - | - | - | - | - | - | - | - | - | - | - | - | - | - | - | - | - | - | - | - | - | - | - | - | - | - | - | - | - | - | - | - | - | - | - | - | - | - | - | - | - | - | - | - | - | - | - | - | - | - | - | - | - | - | - | - | - | - | - | - | - | - | - | - | - | - | - | - | - | - | - | - | - | - | - | - | - | - | - | - | - | - | - | - | - | - | - | - | - | - | - | - | - | - | - | - | - | - | - | - | - | - | - | - | - | - | - | - | - | - | - | - | - | - | - | - | - | - | - | - | - | - | - | - | - | - | - | - | - | - | - | - | - | - | - | - | - | - | - | - | - | - | - | - | - | - | - | - | - | - | - | - | - | - | - | - | - | - | - | - | - | - | - | - | - | - | - | - | - | - | - | - | - | - | - | - | - | - | - | - | - | - | - | - | - | - | - | - | - | - | - | - | - | - | - | - | - | - | - | - | - | - | - | - | - | - | - | - | - | - | - | - | - | - | - | - | - | - | - | - | - | - | - | - | - | - | - | - | - | - | - | - | - | - | - | - | - | - | - | - | - | - | - | - | - | - | - | - | - | - | - | - | - | - | - | - | - | - | - | - | - | - | - | - | - | - | - | - | - | - | - | - | - | - | - | - | - | - | - | - | - | - | - | - | - | - | - | - | - | - | - | - | - | - | - | - | - | - | - | - | - | - | - | - | - | - | - | - | - | - | - | - | - | - | - | - | - | - | - | - | - | - | - | - | - | - | - | - | - | - | - | - | - | - | - | - | - | - | - | - | - | - | - | - | - | - | - | - | - | - | - | - | - | - | - | - | - | - | - | - | - | - | - | - | - | - | - | - | - | - | - | - | - | - | - | - | - | - | - | - | - | - | - | - | - | - | - | - | - | - | - | - | - | - | - | - | - | - | - | - | - | - | - | - | - | - | - | - | - | - | - | - | - | - | - | - | - | - | - | - | - | - | - | - | - | - | - | - | - | - | - | - | - | - | - | - | - | - | - | - | - | - | - | - | - | - | - | - | - | - | - | - | - | - | - | - | - | - | - | - | - | - | - | - | - | - | - | - | - | - | - | - | - | - | - | - | - | - | - | - | - | - | - | - | - | - | - | - | - | - | - | - | - | - | - | - | - | - | - | - | - | - | - | - | - | - | - | - | - | - | - | - | - | - | - | - | - | - | - | - | - | - | - | - | - | - | - | - | - | - | - | - | - | - | - | - | - | - | - | - | - | - | - | - | - | - | - | - | - | - | - | - | - | - | - | - | - | - | - | - | - | - | - | - | - | - | - | - | - | - | - | - | - | - | - | - | - | - | - | - | - | - | - | - | - | - | - | - | - | - | - | - | - | - | - | - | - | - | - | - | - | - | - | - | - | - | - | - | - | - | - | - | - | - | - | - | - | - | - | - | - | - | - | - | - | - | - | - | - | - | - | - | - | - | - | - | - | - | - | - | - | - | - | - | - | - | - | - | - | - | - | - | - | - | - | - | - | - | - | - | - | - | - | - | - | - | - | - | - | - | - | - | - | - | - | - | - | - | - | - | - | - | - | - | - | - | - | - | - | - | - | - | - | - | - | - | - | - | - | - | - | - | - | - | - | - | - | - | - | - | - | - | - | - | - | - | - | - | - | - | - | - | - | - | - | - | - | - | - | - | - | - | - | - | - |
|  | Eric | - | - | - | - | - | - | - | - | - | - | - | - | - | - | - | - | - | - | - | - | - | - | - | - | - | - | - | - | - | - | - | - | - | - | - | - | - | - | - | - | - | - | - | - | - | - | - | M | N | A | A | E | E | D | R | I | R | - | R | N | - | S | D | Y | M | C | C | P | C | A | D | C | K | N | E | N | M | F | D | S | G | - | - | - | - | E | D | V | H | G | H | L | I | Q | R | G | F | M | E | G | Y | T | C | - | - | - | W | V | K | H | - | G | E | - | - | Q | E | S | G | S | G | A | A | A | D | R | S | G | A | H | N | Q | E | D | E | D | - | - | - | - | E | H | D | M | F | I | P | S | P | - | - | - | - | - | - | - | - | - | - | - | - | - | - | - | - | - | - | - | - | - | - | - | L | G | G | - | - | - | - | E | M | V | D | V | D | H | D | L | L | Q | D | M | L | R | D | V | E | - | - | - | - | - | - | - | - | - | - | - | - | - | - | - | - | - | - | - | - | - | - | - | - | - | - | - | - | - | - | - | - | - | - | - | - | - | - | - | D | P | A | Q | N | E | - | - | - | - | - | - | - | - | - | - | - | R | D | G | M | K | F | S | R | L | V | S | D | S | E | T | P | L | Y | - | - | - | A | G | C | K | A | K | H | T | K | L | S | V | T | L | D | L | M | K | L | K | A | S | S | G | W | T | - | - | - | - | - | D | K | S | F | T | D | L | L | G | I | L | - | K | A | M | L | P | V | E | N | T | L | P | E | T | T | Y | E | A | K | Q | V | L | C | P | L | G | L | E | V | R | R | I | H | A | C | P | N | D | C | I | L | Y | H | K | Q | - | Y | A | D | L | D | A | C | P | V | C | K | A | S | R | Y | K | R | K | K | S | A | D | E | G | - | - | - | - | - | - | - | - | - | - | - | - | - | - | - | - | - | - | - | - | - | - | - | - | - | - | - | - | - | - | - | - | - | - | - | - | N | K | S | K | R | G | G | P | A | K | V | V | W | Y | L | P | I | I | D | R | F | K | R | I | F | A | N | P | N | E | A | K | L | V | R | W | H | A | T | E | - | - | - | - | - | - | - | - | - | - | R | R | - | - | - | N | D | G | M | - | - | - | - | - | L | R | H | P | A | D | S | I | E | W | R | N | I | D | R | K | H | K | D | - | F | A | A | D | P | R | N | M | R | I | C | L | C | T | D | G | M | N | P | F | G | D | M | S | S | T | H | S | T | W | P | V | L | I | A | N | Y | N | L | - | P | P | W | L | C | F | K | R | K | Y | I | M | L | C | L | L | I | Q | G | P | R | Q | P | G | N | D | I | - | D | V | F | L | E | P | V | I | D | D | L | E | I | L | W | K | - | E | G | V | E | T | W | D | A | Y | G | Q | E | N | F | K | L | R | V | L | L | F | C | T | I | N | D | Y | P | A | L | G | N | L | S | G | Q | T | I | K | G | K | K | A | - | - | C | S | D | C | K | E | H | T | R | S | R | W | L | K | K | S | R | K | M | V | Y | M | G | - | H | R | R | W | L | P | L | R | H | A | F | R | - | - | R | K | K | K | I | F | N | G | K | R | E | - | L | Q | P | - | A | P | K | D | L | S | - | G | D | E | V | H | - | N | M | V | K | D | I | S | - | N | E | F | G | K | - | - | - | - | - | - | - | - | - | - | - | - | K | R | K | - | - | - | - | - | - | - | - | - | - | - | - | - | - | - | - | - | - | - | - | - | - | - | - | - | - | - | - | - | - | - | R | S | K | T | - | K | E | K | G | M | W | K | K | K | S | I | F | W | R | - | L | P | Y | W | K | - | - | D | L | D | V | R | H | C | I | D | L | M | H | V | E | K | N | V | C | E | S | L | V | G | L | M | L | N | I | P | G | K | T | K | D | G | L | N | A | R | L | D | L | Q | D | M | N | I | R | S | E | L | Q | P | I | R | D | A | E | - | - | - | - | - | - | - | - | - | T | G | K | V | Y | L | P | P | A | C | H | T | L | S | K | D | E | K | I | A | M | L | S | C | L | - | K | D | I | K | V | P | S | G | Y | S | A | R | I | S | K | Y | V | K | L | D | D | L | K | L | V | G | - | M | K | S | H | D | C | H | V | L | I | T | Q | I | L | P | V | A | I | R | G | I | - | - | - | - | - | - | - | L | P | P | - | K | V | R | H | T | I | Q | - | - | - | - | - | - | - | - | - | - | - | - | - | - | - | - | - | - | - | - | - | - | - | R | L | C | A | F | F | N | A | I | G | Q | K | V | I | D | P | E | D | L | D | G | L | Q | T | D | I | V | N | T | L | C | H | L | E | M | F | F | P | L | S | - | - | - | - | F | F | D | I | M | V | H | L | P | V | H | L | V | K | Q | T | K | L | C | G | P | A | F | L | R | E | M | W | P | F | E | - | - | - | - | - | - | - | - | - | - | - | - | - | - | - | - | - | - | - | - | - | - | - | - | - | - | - | - | - | R | Y | - | - | - | - | - | - | - | - | - | - | - | - | - | - | - | - | - | - | - | - | - | - | - | - | - | - | - | - | - | - | - | - | - | - | - | - | - | - | - | - | - | - | - | - | - | - | - | - | - | - | - | - | - | - | - | - | - | - | - | - | - | - | - | - | - | - | - | - | - | - | - | - | - | - | M | G | V | L | K | S | Y | V | R | N | R | A | K | P | E | G | S | I | I | E | G | Y | T | T | E | E | A | I | E | - | - | - | - | F | C | V | N | Y | M | S | - | D | A | D | - | - | P | I | G | V | P | A | S | R | H | E | G | - | - | - | - | - | - | - | - | - | - | - | - | - | - | - | - | - | R | L | S | G | V | G | T | I | G | R | K | R | I | R | P | D | - | - | - | - | Q | A | S | Y | A | Q | A | H | Y | A | V | L | Q | H | M | A | E | V | G | P | - | Y | F | E | E | - | - | - | - | - | - | - | - | - | - | - | - | - | - | - | - | - | - | - | - | - | - | - | - | - | - | - | - | - | - | - | - | - | - | - | - | - | - | - | - | - | - | - | - | - | - | - | - | - | - | - | - | - | - | - | - | - | - | - | - | - | - | - | - | - | - | - | - | - | - | - | - | - | - | - | - | - | - | - | - | - | - | - | - | - | - | - | - | - | - | - | - | - | - | H | L | A | K | I | R | D | E | N | L | G | R | S | D | - | - | A | W | I | N | R | E | H | N | S | R | F | N | E | W | F | K | N | R | V | T | M | - | - | - | - | - | - | - | - | - | - | - | - | - | - | - | - | - | - | - | - | - | - | - | - | - | - | - | - | - | - | - | - | - | S | T | D | V | P | N | E | T | V | Q | L | L | G | M | G | P | S | W | T | V | D | T | W | Q | G | Y | D | I | N | G | Y | T | F | Y | T | V | K | Q | D | D | K | - | - | - | - | S | T | V | Q | N | S | G | V | R | I | D | A | F | Q | D | Q | V | G | S | - | - | - | - | - | - | - | - | - | - | N | T | Y | Y | G | R | I | E | E | I | W | E | L | N | Y | - | - | - | - | - | - | - | - | V | K | F | K | V | P | L | F | R | - | C | R | W | V | N | - | - | - | - | - | - | - | - | - | - | - | - | - | - | L | R | T | G | V | K | A | D | K | E | - | G | F | T | L | - | - | - | - | - | - | - | - | - | - | - | V | D | L | - | - | - | - | - | S | K | V | G | - | - | Y | A | D | E | P | F | V | L | A | K | Q | V | E | Q | I | F | Y | I | K | D | P | - | - | S | N | K | - | - | - | - | - | - | - | - | - | - | K | - | - | - | - | - | - | M | H | I | V | R | D | G | K | R | - | - | - | - | - | - | - | - | - | - | - | R | I | V | G | V | D | N | V | - | - | - | V | D | E | E | - | - | E | Y | N | H | N | L | H | - | V | R | P | H | I | D | L | D | D | D | - | - | - | - | - | - | - | - | - | - | - | - | - | - | - | - | - | - | - | - | - | P | Q | E | P | V | A | Y | A | R | S | D | - | - | H | T | E | G | I | T | L | - | - | - | - | - | - | - | - | - | - | - | - | - | - | - | - | - | - | - | - | - | - | - | - | - | - | - | - | - | - | - | - | - | - | - | - | - | - | - | - | - | - | - | - | - | - | - | - | - | - | - | - | - | - | - | - | - | - | - | - | - | - | - | - | - | - | - | - | - | - | - | - | - | - | - | - | - | - | - | - | - | - | - | - | - | - | - | - | - | - | - | - | - | - | - | - | - | - | - | - | - | - | - | - | - | - | - | - | - | - | - | - | - | - | - | - | - | - | - | - | - | - | - | - | - | - | - | - | - | - | - | - | - | - | - | - | - | - | - | - | - | - | - | - | - | - | - | - | - | - | - | - | - | - | - | - | - | - | - | - | - | - | - | - | - | - | - | - | - | - | - | - | - | - | - | - | - | - | - | - | - | - | - | - | - | - | - | - | - | - | - | - | - | - | - | - | - | - | - | - | - | - | - | - | - | - | - | - | - | - | - | - | - | - | - | - | - | - | - | - | - | - | - | - | - | - | - | - | - | - | - | - | - | - | - | - | - | - | - | - | - | - | - | - | - | - | - | - | - | - | - | - | - | - | - | - | - | - | - | - | - | - | - | - | - | - | - | - | - | - | - | - | - | - | - | - | - | - | - | - | - | - | - | - | - | - | - | - | - | - | - | - | - | - | - | - | - | - | - | - | - | - | - | - | - | - | - | - | - | - | - | - | - | - | - | - | - | - | - | - | - | - | - | - | - | - | - | - | - | - | - | - | - | - | - | - | - | - | - | - | - | - | - | - | - | - | - | - | - | - | - | - | - | - | - | - | - | - | - | - | - | - | - | - | - | - | - | - | - | - | - | - | - | - | - | - | - | - | - | - | - | - | - | - | - | - | - | - | - | - | - | - | - | - | - | - | - | - | - | - | - | - | - | - | - | - | - | - | - | - | - | - | - | - | - | - | - | - | - | - | - | - | - | - | - | - | - | - | - | - | - | - | - | - | - | - | - | - | - | - | - | - | - | - | - | - | - | - | - | - | - | - | - | - | - | - | - | - | - | - | - | - | - | - | - | - | - | - | - | - | - | - | - | - | - | - | - | - | - | - | - | - | - | - | - | - | - | - | - | - | - | - | - | - | - | - | - | - | - | - | - | - | - | - | - | - | - | - | - | - | - | - | - | - | - | - | - | - | - | - | - | - | - | - | - | - | - | - | - | - | - | - | - | - | - | - | - | - | - | - | - | - | - | - | - | - | - | - | - | - | - | - | - | - | - | - | - | - | - | - | - | - | - | - | - | - | - | - | - | - | - | - | - | - | - | - | - | - | - | - | - | - | - | - | - | - | - | - | - | - | - | - | - | - | - | - | - | - | - | - | - | - | - | - | - | - | - | - | - | - | - | - | - | - | - | - | - | - | - | - | - | - | - | - | - | - | - | - | - | - | - | - | - | - | - | - | - | - | - | - | - | - | - | - | - | - | - | - | - | - | - | - | - | - | - | - | - | - | - | - | - | - | - | - | - | - | - | - | - | - | - | - | - | - | - | - | - | - | - | - | - | - | - | - | - | - | - | - | - | - | - | - | - | - | - | - | - | - | - | - | - | - | - | - | - | - | - | - | - | - | - | - | - | - | - | - | - | - | - | - | - | - | - | - | - | - | - | - | - | - | - | - | - | - | - | - | - | - | - | - | - | - | - | - | - | - | - | - | - | - | - | - | - | - | - | - | - | - | - | - | - | - | - | - | - | - | - | - | - | - | - | - | - | - | - | - | - |
|  | Grover | - | - | - | - | - | M | - | - | - | - | - | - | - | - | - | - | - | - | - | D | R | - | R | W | M | Y | Y | - | - | - | - | - | A | H | R | S | S | T | E | Y | R | E | G | V | T | E | F | V | T | F | A | D | N | D | R | K | S | - | R | M | - | S | M | H | M | L | C | P | C | R | D | C | K | N | E | Q | M | I | E | D | K | - | - | - | - | D | E | V | H | A | H | L | I | M | N | G | F | M | K | K | Y | T | C | - | - | - | W | T | K | H | - | G | E | - | - | Q | E | A | P | D | V | A | A | E | E | - | - | - | V | L | D | Q | D | V | E | N | T | A | A | A | R | E | G | M | F | V | P | S | P | - | - | - | - | - | - | - | - | - | - | - | - | - | - | - | - | - | - | - | - | - | - | - | L | G | G | - | - | - | - | E | T | I | D | L | D | T | Q | C | L | S | T | M | L | H | D | I | E | - | - | - | - | - | - | - | - | - | - | - | - | - | - | - | - | - | - | - | - | - | - | - | - | - | - | - | - | - | - | - | - | - | - | - | - | - | - | - | D | A | E | D | N | D | - | - | - | - | - | - | - | - | - | - | - | R | D | Y | E | K | F | S | K | L | V | E | D | C | Q | M | P | L | Y | - | - | - | D | G | C | K | S | K | H | S | K | L | S | C | V | L | E | L | M | K | L | K | A | S | N | G | W | S | - | - | - | - | - | D | K | S | F | T | E | L | L | E | L | L | - | K | D | L | L | P | E | G | N | N | L | P | Q | T | T | Y | E | A | K | Q | V | L | C | P | L | G | L | E | V | R | R | I | H | A | C | P | N | D | C | I | L | Y | Y | K | E | - | Y | A | D | L | D | V | C | P | I | C | G | A | S | R | Y | K | R | A | K | S | E | G | E | G | - | - | - | - | - | - | - | - | - | - | - | - | - | - | - | - | - | - | - | - | - | - | - | - | - | - | - | - | - | - | - | - | - | - | - | - | S | K | S | K | R | G | G | P | A | K | V | V | W | Y | L | P | I | A | E | R | M | K | R | M | F | A | N | K | E | Q | A | K | L | V | R | W | H | A | E | E | - | - | - | - | - | - | - | - | - | - | R | K | - | - | - | V | D | T | M | - | - | - | - | - | L | R | H | P | A | D | S | V | Q | W | R | T | I | D | R | I | Y | Q | E | - | F | S | N | D | P | R | N | M | R | F | A | M | C | T | D | G | I | N | P | F | G | D | L | S | S | R | H | S | T | W | P | V | L | L | V | N | Y | N | L | - | P | P | W | L | C | F | K | R | K | Y | I | M | L | A | M | L | I | Q | G | P | R | Q | P | G | N | D | I | - | D | V | F | L | E | P | I | I | D | D | F | E | R | L | W | N | - | E | G | T | R | T | W | D | A | Y | A | Q | E | Y | F | N | L | H | A | M | L | F | C | T | I | N | D | Y | P | A | L | G | N | L | S | G | Q | T | V | K | G | K | W | A | - | - | C | S | E | C | M | E | E | T | R | S | K | W | L | K | H | S | H | K | T | V | Y | M | G | - | H | R | R | F | L | P | R | Y | H | P | Y | R | - | - | N | M | R | K | N | F | N | G | H | R | D | - | T | A | G | - | P | P | T | E | L | T | - | G | T | E | V | H | - | N | L | V | M | G | I | T | - | N | E | F | G | K | - | - | - | - | - | - | - | - | - | - | - | - | K | R | K | V | G | K | R | K | E | K | S | - | - | - | - | - | - | - | - | - | - | - | - | T | S | K | E | K | T | E | E | H | V | E | K | Q | K | T | - | K | E | R | S | M | W | K | K | K | S | I | F | W | R | - | L | P | Y | W | K | - | - | D | L | E | V | R | H | C | I | D | L | M | H | V | E | K | N | V | C | E | S | L | M | G | L | L | L | N | - | P | G | T | T | K | D | G | L | N | A | R | R | D | L | E | D | M | G | V | R | S | E | L | H | P | I | - | T | T | E | - | - | - | - | - | - | - | - | - | S | G | R | V | Y | L | P | P | A | C | Y | T | L | S | K | E | E | K | I | D | L | L | T | C | L | - | S | G | I | K | V | P | S | G | Y | S | S | R | I | S | R | L | V | S | L | Q | D | L | K | L | V | G | - | M | K | S | H | D | C | H | V | L | I | T | Q | L | L | P | V | A | I | R | N | I | - | - | - | - | - | - | - | L | P | P | - | K | V | R | H | T | I | Q | - | - | - | - | - | - | - | - | - | - | - | - | - | - | - | - | - | - | - | - | - | - | - | R | L | C | S | F | F | H | A | I | G | Q | K | I | I | D | P | E | G | L | D | E | L | Q | A | E | L | V | R | T | L | C | H | L | E | M | Y | F | P | P | T | - | - | - | - | F | F | D | I | M | E | H | L | P | V | H | L | V | R | Q | T | K | C | C | G | P | A | F | M | T | Q | M | Y | P | C | E | - | - | - | - | - | - | - | - | - | - | - | - | - | - | - | - | - | - | - | - | - | - | - | - | - | - | - | - | - | R | Y | - | - | - | - | - | - | - | - | - | - | - | - | - | - | - | - | - | - | - | - | - | - | - | - | - | - | - | - | - | - | - | - | - | - | - | - | - | - | - | - | - | - | - | - | - | - | - | - | - | - | - | - | - | - | - | - | - | - | - | - | - | - | - | - | - | - | - | - | - | - | - | - | - | - | L | G | I | L | K | G | Y | V | R | N | R | S | H | P | E | G | S | I | I | E | S | Y | T | T | E | E | A | I | E | - | - | - | - | F | C | V | D | Y | M | S | - | E | T | S | - | - | S | I | G | L | P | R | S | H | H | E | G | - | - | - | - | - | - | - | - | - | - | - | - | - | - | - | - | - | R | L | D | G | V | G | T | V | G | R | K | T | I | R | L | D | - | - | - | - | R | K | V | Y | D | K | A | H | F | T | V | L | Q | H | M | T | E | V | V | P | - | Y | V | D | E | - | - | - | - | - | - | - | - | - | - | - | - | - | - | - | - | - | - | - | - | - | - | - | - | - | - | - | - | - | - | - | - | - | - | - | - | - | - | - | - | - | - | - | - | - | - | - | - | - | - | - | - | - | - | - | - | - | - | - | - | - | - | - | - | - | - | - | - | - | - | - | - | - | - | - | - | - | - | - | - | - | - | - | - | - | - | - | - | - | - | - | - | - | - | H | L | A | V | I | R | Q | E | N | P | G | R | S | E | - | - | S | W | V | R | N | K | H | M | S | S | F | N | E | W | L | K | N | R | I | A | R | - | - | - | - | - | - | - | - | - | - | - | - | - | - | - | - | - | - | - | - | - | - | - | - | - | - | - | - | - | - | - | - | - | L | Q | N | L | P | S | E | T | L | Q | W | L | S | Q | G | P | E | W | S | A | T | T | W | Q | G | Y | D | I | N | G | Y | T | F | H | T | V | K | Q | D | S | K | - | - | - | - | C | T | V | Q | N | S | G | L | R | I | E | A | A | S | D | G | G | R | R | - | - | - | - | - | - | - | - | - | - | D | Q | Y | Y | G | R | V | E | Q | I | L | E | L | D | Y | - | - | - | - | - | - | - | - | L | K | F | K | V | P | L | F | R | - | C | R | W | V | D | - | - | - | - | - | - | - | - | - | - | - | - | - | - | - | L | R | N | V | K | V | D | N | E | - | A | F | T | T | - | - | - | - | - | - | - | - | - | - | - | V | N | L | - | - | - | - | - | A | N | N | A | - | - | Y | K | D | E | P | F | V | L | A | K | Q | V | V | Q | V | F | Y | I | V | D | P | - | - | C | N | K | - | - | - | - | - | - | - | - | - | - | K | - | - | - | - | - | - | L | H | V | V | R | E | G | K | R | - | - | - | - | - | - | - | - | - | - | - | R | I | V | G | L | D | N | I | - | - | - | A | D | E | D | - | - | D | Y | N | Q | H | V | H | G | I | G | Q | E | I | P | L | E | E | E | E | - | - | - | - | - | - | - | - | - | - | - | - | - | - | - | - | - | - | - | - | E | E | D | E | V | Q | Y | A | R | V | D | - | - | H | E | E | G | - | - | - | - | - | - | - | - | - | - | - | - | - | - | - | - | - | - | - | - | - | - | - | - | - | - | - | - | - | - | - | - | - | - | - | - | - | - | - | - | - | - | - | - | - | - | - | - | - | - | - | - | - | - | - | - | - | - | - | - | - | - | - | - | - | - | - | - | - | - | - | - | - | - | - | - | - | - | - | - | - | - | - | - | - | - | - | - | - | - | - | - | - | - | - | - | - | - | - | - | - | - | - | - | - | - | - | - | - | - | - | - | - | - | - | - | - | - | - | - | - | - | - | - | - | - | - | - | - | - | - | - | - | - | - | - | - | - | - | - | - | - | - | - | - | - | - | - | - | - | - | - | - | - | - | - | - | - | - | - | - | - | - | - | - | - | - | - | - | - | - | - | - | - | - | - | - | - | - | - | - | - | - | - | - | - | - | - | - | - | - | - | - | - | - | - | - | - | - | - | - | - | - | - | - | - | - | - | - | - | - | - | - | - | - | - | - | - | - | - | - | - | - | - | - | - | - | - | - | - | - | - | - | - | - | - | - | - | - | - | - | - | - | - | - | - | - | - | - | - | - | - | - | - | - | - | - | - | - | - | - | - | - | - | - | - | - | - | - | - | - | - | - | - | - | - | - | - | - | - | - | - | - | - | - | - | - | - | - | - | - | - | - | - | - | - | - | - | - | - | - | - | - | - | - | - | - | - | - | - | - | - | - | - | - | - | - | - | - | - | - | - | - | - | - | - | - | - | - | - | - | - | - | - | - | - | - | - | - | - | - | - | - | - | - | - | - | - | - | - | - | - | - | - | - | - | - | - | - | - | - | - | - | - | - | - | - | - | - | - | - | - | - | - | - | - | - | - | - | - | - | - | - | - | - | - | - | - | - | - | - | - | - | - | - | - | - | - | - | - | - | - | - | - | - | - | - | - | - | - | - | - | - | - | - | - | - | - | - | - | - | - | - | - | - | - | - | - | - | - | - | - | - | - | - | - | - | - | - | - | - | - | - | - | - | - | - | - | - | - | - | - | - | - | - | - | - | - | - | - | - | - | - | - | - | - | - | - | - | - | - | - | - | - | - | - | - | - | - | - | - | - | - | - | - | - | - | - | - | - | - | - | - | - | - | - | - | - | - | - | - | - | - | - | - | - | - | - | - | - | - | - | - | - | - | - | - | - | - | - | - | - | - | - | - | - | - | - | - | - | - | - | - | - | - | - | - | - | - | - | - | - | - | - | - | - | - | - | - | - | - | - | - | - | - | - | - | - | - | - | - | - | - | - | - | - | - | - | - | - | - | - | - | - | - | - | - | - | - | - | - | - | - | - | - | - | - | - | - | - | - | - | - | - | - | - | - | - | - | - | - | - | - | - | - | - | - | - | - | - | - | - | - | - | - | - | - | - | - | - | - | - | - | - | - | - | - | - | - | - | - | - | - | - | - | - | - | - | - | - | - | - | - | - | - | - | - | - | - | - | - | - | - | - | - | - | - | - | - | - | - | - | - | - | - | - | - | - | - | - | - | - | - | - | - | - | - | - | - | - | - | - | - | - | - | - | - | - | - | - | - | - | - | - | - | - | - | - | - | - | - | - | - | - | - | - | - | - | - | - | - | - | - | - | - | - | - | - | - | - | - | - | - | - | - | - | - | - | - | - | - | - | - | - | - | - | - | - | - | - | - | - | - | - | - | - | - | - | - | - | - | - | - | - | - | - | - | - | - | - | - | - | - | - | - | - | - | - | - | - | - | - | - | - | - | - | - | - | - | - |
[truncated: 298,594 more chars]
