# Supplementary figures and images for: Analysis of CACTA transposases reveals intron loss as major factor influencing their exon/intron structure in monocotyledonous and eudicotyledonous hosts
Source: Mob DNA. 2014 Sep 1;5:24. doi: 10.1186/1759-8753-5-24 (PMC4158355; doi:10.1186/1759-8753-5-24)

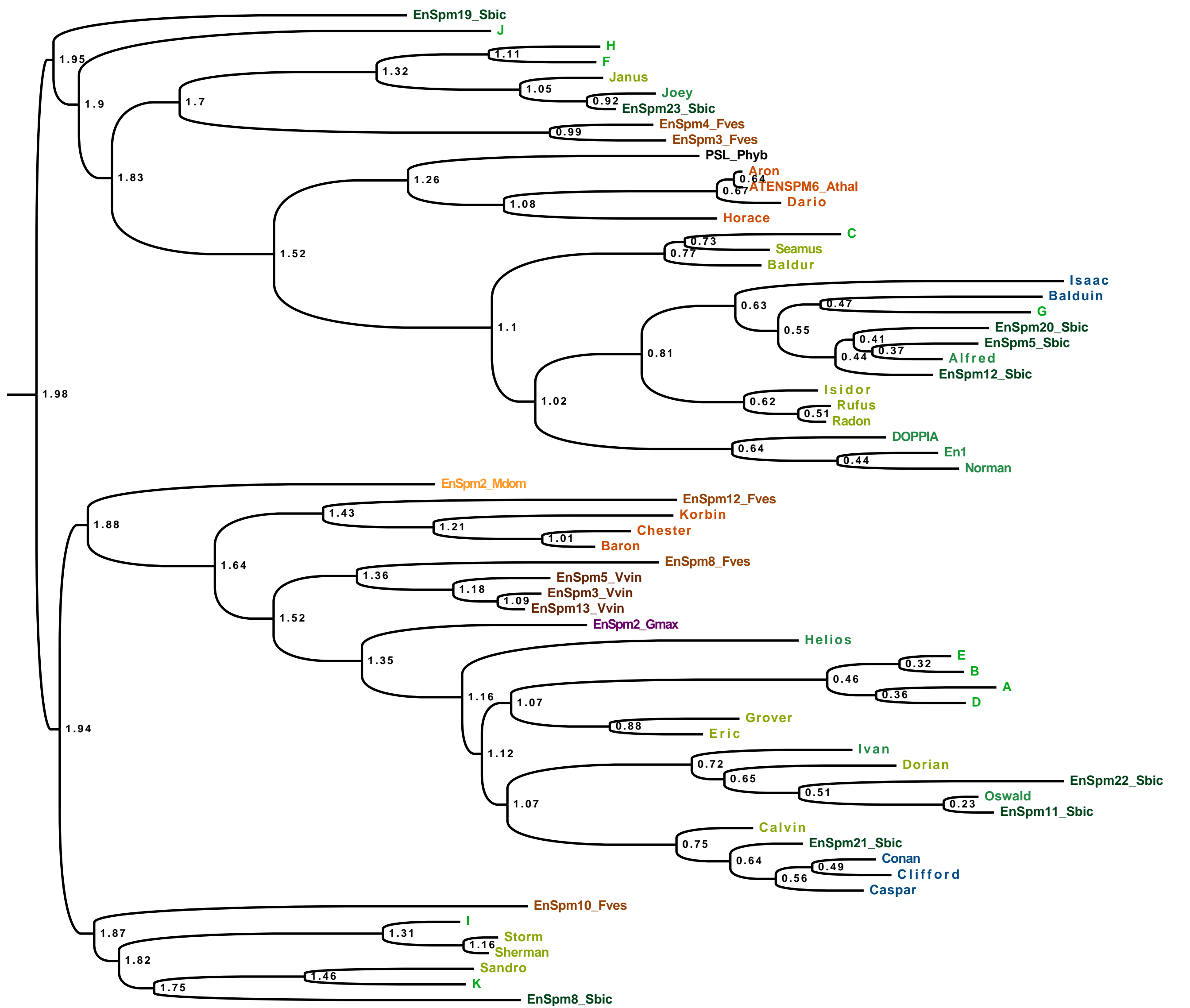

Supplement: Additional file 3 — Best maximum likelihood tree for the 57 analyzed CACTA transposases. Describe s the best maximum likelihood tree out of 200 distinct, randomized, maximum parsimony trees for the 64 analyzed CACTA transposases. The tree has been mid-point rooted due to the lack of an available outgroup. Contains the 12 maximum likelihood trees in the Newick format which were used to check the robustness of the initial maximum likelihood tree. It can be opened using most modern phylogenetic programs. [file 1759-8753-5-24-S3.zip › Additional_File3.pdf]
